# MetaboSERV—a platform for selecting, exchanging, and visualizing metabolomics data with controlled data access

**DOI:** 10.1093/gigascience/giaf075

**Published:** 2025-08-01

**Authors:** Tim Tucholski, Angela Maennel, Yacoub Abelard Njipouombe Nsangou, Sven Schuchardt, Matthias Gruber, Fabian Kellermeier, Katja Dettmer, Peter J Oefner, Wolfram Gronwald, Michael Altenbuchinger, Jürgen Dönitz, Helena U Zacharias

**Affiliations:** Department of Medical Bioinformatics, University Medical Center Göttingen, 37077 Göttingen, Germany; Peter L. Reichertz Institute for Medical Informatics of TU Braunschweig and Hannover Medical School, Hannover Medical School, 30625 Hannover, Germany; Department of Medical Bioinformatics, University Medical Center Göttingen, 37077 Göttingen, Germany; Institute of Computational Biology, Helmholtz Center Munich, 85764 Neuherberg, Germany; Department of Bio- and Environmental Analytics, Fraunhofer Institute for Toxicology and Experimental Medicine ITEM, 30625 Hannover, Germany; Institute of Functional Genomics, University of Regensburg, 93053 Regensburg, Germany; Institute of Functional Genomics, University of Regensburg, 93053 Regensburg, Germany; Institute of Functional Genomics, University of Regensburg, 93053 Regensburg, Germany; Institute of Functional Genomics, University of Regensburg, 93053 Regensburg, Germany; Institute of Functional Genomics, University of Regensburg, 93053 Regensburg, Germany; Department of Medical Bioinformatics, University Medical Center Göttingen, 37077 Göttingen, Germany; Department of Medical Bioinformatics, University Medical Center Göttingen, 37077 Göttingen, Germany; Institute of Computational Biology, Helmholtz Center Munich, 85764 Neuherberg, Germany; Campus Institute Data Science (CIDAS) Göttingen, 37077 Göttingen, Germany; Peter L. Reichertz Institute for Medical Informatics of TU Braunschweig and Hannover Medical School, Hannover Medical School, 30625 Hannover, Germany

**Keywords:** (privacy-preserving) data sharing, metabolomics, nuclear magnetic resonance spectroscopy, mass spectrometry, collaborative research

## Abstract

**Background:**

The growing number of metabolomics studies, based on high-dimensional data measured by hyphenated mass spectrometry (MS) and/or nuclear magnetic resonance (NMR) spectroscopy, has sparked the creation of several public metabolomics data repositories. Each repository emphasizes different aspects regarding data selection and representation, but most offer only limited options for privacy-preserving data sharing.

**Results:**

We present MetaboSERV, an open-source, browser-based metabolomics platform dedicated to the selection, integration, and sharing of quantitative metabolomics data and metadata with controlled data access. MetaboSERV aims to aid researchers in analyzing their results by facilitating means to browse, visualize, and compare data across available datasets. It provides different access control functionalities, creating an environment in which data can be shared safely in a privacy-preserving manner to support collaborative and interdisciplinary research. Furthermore, it is designed to be extensible and adaptable to existing data management infrastructures through the creation of self-managed MetaboSERV instances, for which we provide the source code and a set of configurable Docker images.

**Conclusions:**

The public MetaboSERV instance is available at https://metaboserv.ckdn.app, and the source code can be found at https://gitlab.gwdg.de/MedBioinf/metabolomics/metaboserv. The Research Resource Identifier (RRID) for MetaboSERV is *SCR_025496*.

## Background

Metabolomics is the comprehensive study and quantitative analysis of all metabolites that are detectable in a biological specimen. It has found a wide range of applications in the medical field, including the identification of biomarkers and elucidation of molecular pathomechanisms in precision medicine [1–3]. Nuclear magnetic resonance (NMR) spectroscopy and hyphenated mass spectrometry (MS) are the 2 most widely used analytical methods in metabolomics and are suitable for large-scale studies [4, 5]. In response to the fast increase in the number of metabolomics studies published and the corresponding generation of vast amounts of research data, different metabolomics data repositories such as MetaboLights [6], Metabolonote [7], Metabolomic Repository Bordeaux (MeRy-B) [8], the Metabolomics Workbench [9], and more recent platforms such as MetHoS [10] and COMETS Analytics [11] have been created. MetaboLights, Metabolomics Workbench, MeRy-B, and Metabolonote primarily focus on fully open public sharing of metabolomics data. MeRy-B solely accommodates NMR-based metabolomics plant data, and Metabolonote solely accommodates metabolomics metadata, respectively. COMETS Analytics and MetHoS enable comprehensive data analysis of stored experimental data, the former being specifically designed for meta-analyses and the latter with a particular focus on untargeted MS data. The repositories are constantly evolving to fit the needs of the research community [12], enabling researchers to make their experimental data findable, accessible, interoperable, and reusable as defined by the FAIR principles for scientific data management and stewardship [13, 14].

**Figure 1:**
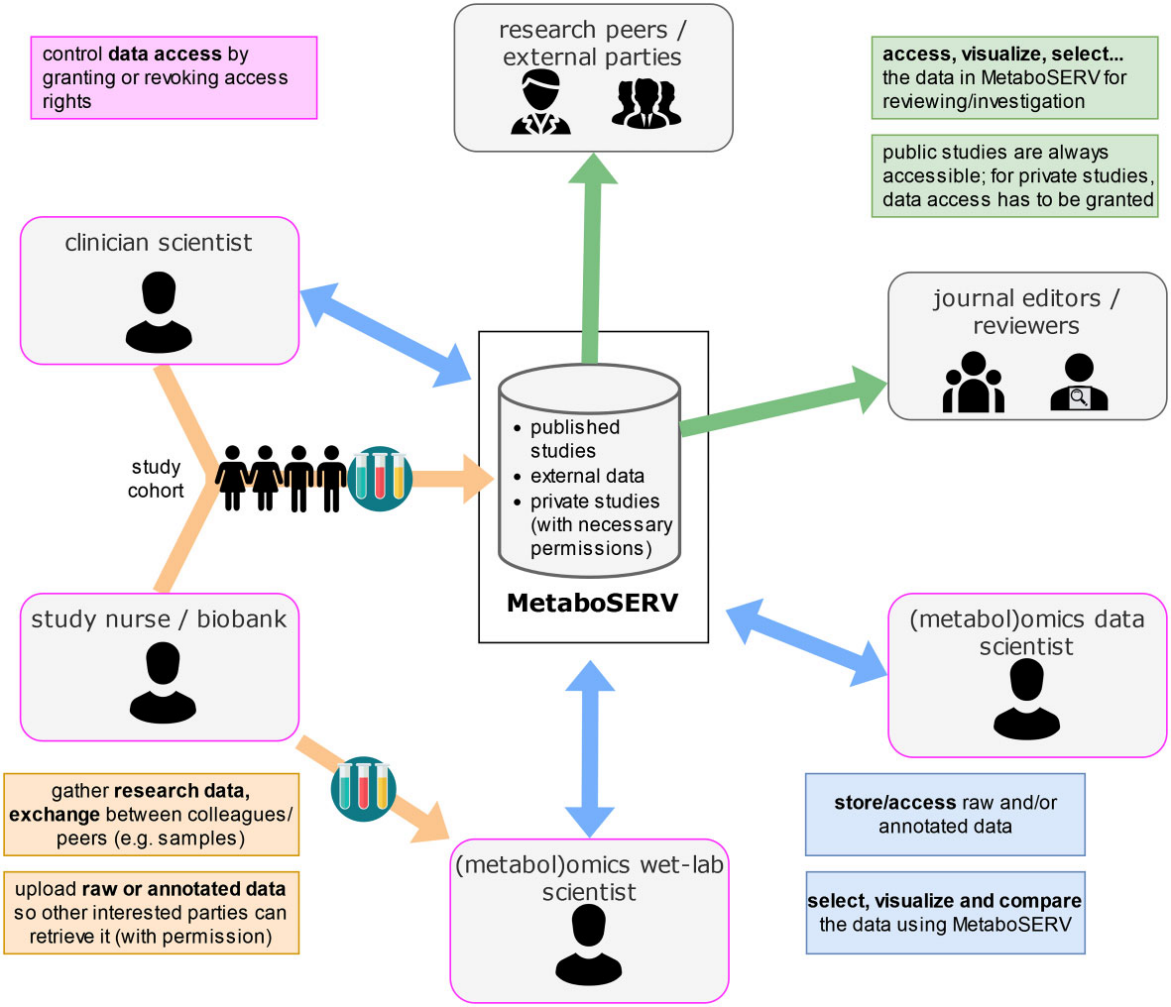
An exemplary schema of how the MetaboSERV platform can connect interdisciplinary collaborators of a research project as well as external parties: clinician scientists and study nurses gather phenotypic information and biofluid specimens from the study cohort, which are further measured by metabolomics wet-lab scientists. All 3 collaboration partners can store their collected raw and processed data on the MetaboSERV platform by either employing the public MetaboSERV instance or a local MetaboSERV instance, autonomously set up at one of the collaborating institutions. (Metabol)omics data scientists can access the data in MetaboSERV, analyze them, and upload further results to MetaboSERV. Additional access can be granted to research peers who are interested in the data, as well as to journal editors and reviewers to facilitate peer review. Credit: Icons in figure 1 were taken from svgrepo.com and fall under the Creative Commons (CC0) license.

Existing repositories, in particular MetaboLights [6] and Metabolomics Workbench [9], focus on fully open public sharing of experimental data and/or metadata upon publication of study results. They provide only limited options for controlled access sharing of metabolomics data within a specific group of researchers. However, the latter is the typical scenario in an interdisciplinary collaboration, where clinicians, metabolomics experimentalists, and (metabolomics) data scientists/bioinformaticians perform dedicated tasks in a joint metabolomics research project (Fig. 1). To foster collaborative and interdisciplinary research, all partners, irrespective of their (potentially highly diverse) programming skills, should be able to browse and visualize the metabolomics data as well as generate summary statistics and carry out different data analysis tasks within a data privacy–preserving environment. Especially biomedical metabolomics data from human studies require specific attention to data security. Just recently, a call for controlled access models for metabolomics data sharing repositories, as a potential requirement due to patient consent statements, personal data regulations such as the European Union General Data Protection Regulation (GDPR), or other relevant legislation, has been issued [15]. This call demonstrates the urgent need of providing metabolomics data repositories with controllable data access.

We present MetaboSERV, an open-source browser-based platform for controlled access sharing of NMR and MS metabolomics data, metadata, and research results. MetaboSERV offers rich and intuitive data selection, browsing, and visualization functionalities and aims to facilitate controlled data accessibility within research collaborations, particularly prior to publication of research findings. The MetaboSERV platform can be employed through either the public MetaboSERV instance [16] or through fully autonomous, self-managed, local MetaboSERV instances that can be set up and operated by end users utilizing our configurable Docker images and detailed user guides.

## Methods

### Implementation

#### Data storage

Metabolite concentration data and associated metadata (such as phenotype data) are stored in 2 different databases: an Elasticsearch [17] instance running on version 8.3 and a MariaDB instance running on version 10.11, with a Python interface [18], respectively. Other experimental data (such as raw spectral data) that can be uploaded to MetaboSERV are stored on the server MetaboSERV is hosted on. MariaDB contains numerical data, such as metabolite concentrations and reference values retrieved from the Human Metabolome Database (HMDB), version 5.0 [19]. Elasticsearch contains user account data, phenotype data, and study metadata. The schemaless data storage provided by Elasticsearch enables MetaboSERV to be flexible with regard to metadata that the user uploads for a study. The Python Elasticsearch Client [20] acts as a wrapper around Elasticsearch, providing basic database querying functions that are then translated into Elasticsearch Query Domain Specific Language (DSL) queries.

#### User interaction

The MetaboSERV web interface is tailored to facilitate seamless user interaction. It is based on the VueJS3 [21] framework and mainly implemented in TypeScript [22, 23]. In addition, it makes use of the client-side-store capabilities provided by Pinia [24] and the AG Data Grid [25] package for table creation.

#### Supplementary web services

MetaboSERV utilizes 2 web services to (i) offer an application programming interface (API) and (ii) process raw metabolomics NMR data. The first web service is implemented in Python [26] and uses the package flask [27] to provide the API for supplying data to the web application. Nonnative packages used for the service include numpy [28], pandas [29], matplotlib [30], seaborn [31], and xmltodict [32]. Flask-related add-ons include flask-cors [33], flask-swagger-ui [34], flask-jwt-extended [35], and werkzeug [36]. The package gunicorn [37] is used as a production WSGI server on top of flask. The package python-magic [38] helps with file validation. The second web service for processing raw metabolomics data, including raw NMR spectra, is R-based [39] and makes use of the packages restRserve [40] and mrbin [41]. For a complete list of used TypeScript, Python, and R packages and their respective versions, please refer to Supplementary Table S1.

#### Data privacy and security

Following the principles of personal data minimization in the GDPR, only required personal data are collected and stored in MetaboSERV. New users need to provide an e-mail address upon registration, which is automatically validated and can be used to recover lost passwords, which are saved securely encrypted in the database (salted and hashed). Communication between all modules is handled using Hypertext Transfer Protocol (HTTP) or preferably HTTP-secure (HTTPS) requests and responses. For all noninternal communication, HTTPS is enforced. More details are provided in the Supplementary Section “User Authentication and Password Storage.”

### Metabolomics metadata

Metabolite metadata consisting of reference concentration ranges for healthy humans for the most common human biofluids (urine, plasma, serum, feces, and cerebral spinal fluid) and synonym lists were retrieved from the HMDB, version 5.0 [19].

### Metabolomics use case data

NMR data from a previous study on 106 patients undergoing cardiac surgery [42] served as an exemplary dataset for MetaboSERV and were used to guide the implementation process. Thirty-four of the 106 patients had been diagnosed with postoperative acute kidney injury (AKI) [42]. It includes 1D $^1$H NMR spectra from urine specimens collected from all study participants 24 hours after cardiac surgery with cardiopulmonary bypass (CPB) use. These spectra were acquired using a 600 MHz Bruker Avance III spectrometer (Bruker BioSpin GmbH). Additionally, 1D $^1$H NMR spectra from 85 plasma specimens of the same study participants were collected, measured, and absolutely quantified as described in [43].

A second use case dataset includes absolute concentrations of 630 metabolites measured in 9 aliquots of the NIST frozen human plasma Standard Reference Material 1950 (SRM 1950). Data were acquired on an AB Sciex 6500+ triple quadrupole mass spectrometer (AB Sciex Germany GmbH) coupled to an ExionLC 30AD (AB Sciex Germany GmbH) ultra-high-performance liquid chromatography (UHPLC) system employing the MxP Quant 500 kit (Biocrates Life Sciences).

A third use case dataset consists of 1,228 unique metabolites semi-quantitatively measured on the Metabolon H4 platform in 1,002 human blood plasma specimens [44]. Data were acquired on a Thermo Scientific Q-Exactive high-resolution/accurate mass spectrometer interfaced with a heated electrospray ionization (HESI-II) source and utilizing a Waters ACQUITY ultra-performance liquid chromatography (UPLC) system. A methanol extraction was performed for protein precipitation, and the resulting extract of each specimen was divided into 5 fractions: 2 fractions were used for analysis by 2 separate reversed-phase (RP)/UPLC-MS/MS methods with positive ion mode electrospray ionization (ESI), 1 fraction was used for analysis by RP/UPLC-MS/MS with negative ion mode ESI, 1 fraction was used for analysis by hydrophilic interaction liquid chromatography (HILIC)/UPLC-MS/MS with negative ion mode ESI, and 1 aliquot was reserved for backup. Data on the original scale (i.e., values normalized in terms of raw area counts without missing value imputation as provided by Metabolon) were downloaded from the Metabolomics Workbench [45], Project ID PR001762.

## Results

### MetaboSERV architecture

**Figure 2:**
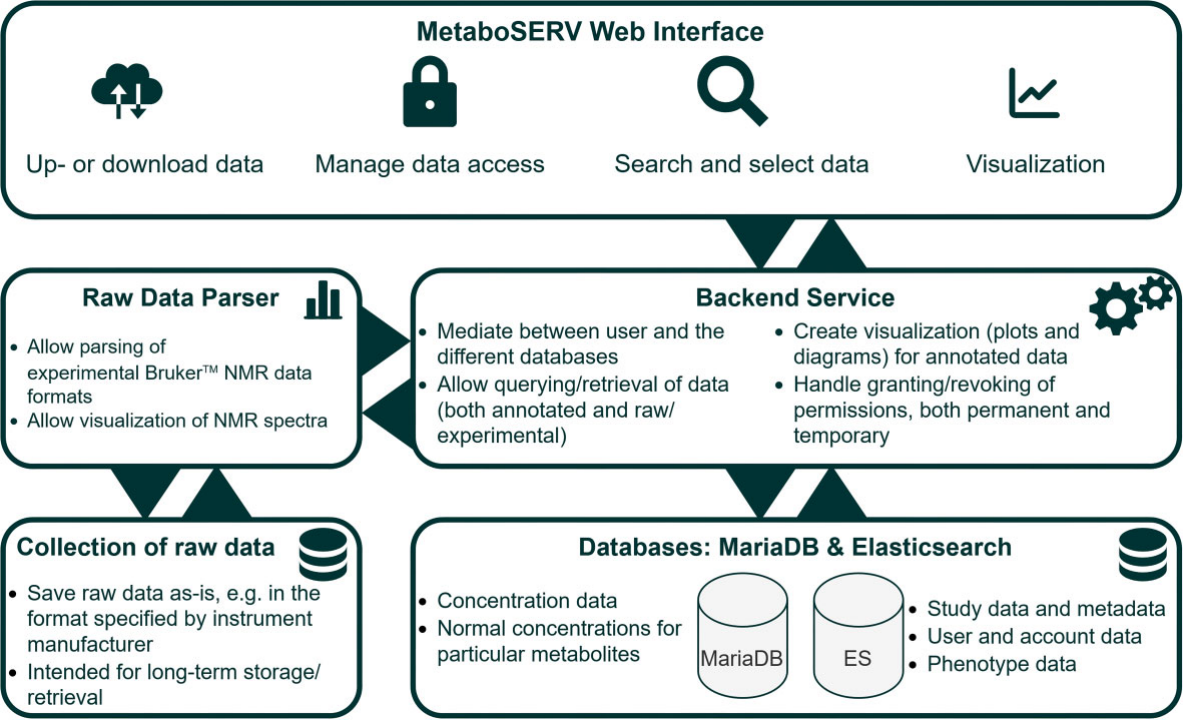
The 4 modules of MetaboSERV. Users interact solely with the *MetaboSERV Web Interface*, while the *Backend Service* mediates between the user and the *Databases* as well as the *Raw Data Parser* for parsing Bruker NMR spectra.

MetaboSERV is a web-based, open-source metabolomics platform, specifically designed for controlled user access and cross-comparison between studies. It includes 4 interconnected modules: the *MetaboSERV Web Interface*, the *Backend Service*, the *Databases*, and the *Raw Data Parser*, as presented in Fig. 2.

The *MetaboSERV Web Interface* serves as an interface to all functionalities and data contained in MetaboSERV, facilitating seamless interaction with the user. Raw experimental data (e.g., spectra derived from NMR or MS experiments) can also be stored in MetaboSERV. However, raw experimental data are saved as-is on the server without any additional processing. MetaboSERV further includes an R-based *Raw Data Parser*, which is capable of parsing, processing, and visualizing NMR raw frequency domain data provided in the Bruker format. Finally, to encapsulate “create, read, update, and delete (CRUD)” operations to the databases and to add logic and visualization options, MetaboSERV includes an extensible web service, referred to as the *Backend Service*, that mediates between the web interface and the other modules. The *Backend Service* also handles user authentication and authorization as well as file validation measures.

### MetaboSERV server environment

The public MetaboSERV instance [16] is hosted at the computing center of the Hannover Medical School, Germany (MHH). MHH’s regulations for access control to the server, security updates, backup, and monitoring are implemented following the ISO 27001 and the standards of the German Federal Office for Information Security (Bundesamt für Sicherheit in der Informationstechnik, BSI). In particular, the public MetaboSERV server at MHH and the data stored there can only be accessed by authorized administrators. Server access is continuously logged and regularly inspected. The virtual machine is equipped with the latest security updates, and regular backups are being taken every 6 hours.

Local MetaboSERV instances can be set up by cloning our repositories [46] and creating, configuring, and running the respective Docker images. Detailed user guides are provided in Supplementary File S6: “Detailed Installation and User Guide for the Set-up of Local MetaboSERV Instances,” as well as at the “Guide” section of our public MetaboSERV instance and in our GitLab repositories. MetaboSERV is generally resource-friendly; all core components can be set up on a dual-core machine with 6 GB of random access memory (RAM) and 200 GB of hard disk space for MariaDB and Elasticsearch. Furthermore, a sufficient amount of hard disk space is necessary to store raw metabolomics data. Query speed is heavily dependent on the resources attributed to the underlying Elasticsearch and MariaDB instances. Therefore, it is recommended to set up MetaboSERV on a machine with at least 4 cores and 16 GB of RAM and to take advantage of Elasticsearch’s *sharding* mechanism [47]. By default, MetaboSERV makes no assumptions about the Elasticsearch environment to avoid structural and capacity-related issues. These self-managed MetaboSERV instances run isolated from the public MetaboSERV instance and allow hosting and managing data on self-governed servers, removing any further data privacy concerns. They can also be altered and configured to fit different research environments and data formats, as well as ensure a degree of system portability due to the nature of container virtualization. More information on user-specific configuration settings are provided in Supplementary File S6.

### Data upload and processing

**Figure 3:**
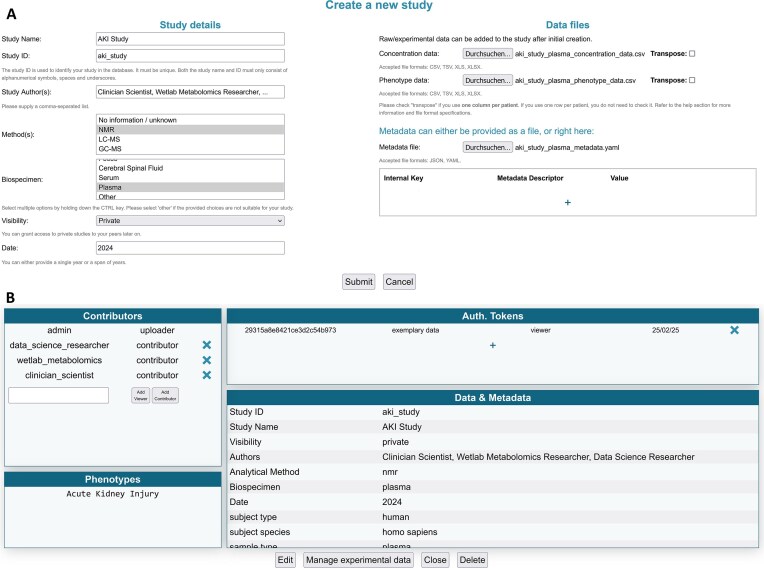
(A) MetaboSERV platform data upload menu. Several files and metadata attributes can be provided for a study. Concentration and phenotype data can be added (concentration data are required). Metadata can be provided by means of a JSON/YAML file or directly in the application. (B) MetaboSERV study management menu. Access can be granted to other users, either “read-only” access or (limited) “write” access (top left). Access can be revoked, though the study uploader privileges can neither be removed nor altered at any time. Available phenotypic information of the stored study is listed under “Phenotypes” (bottom left). Authorization tokens for a particular study can be added/removed, if desired, with specific expiration dates, comments, and additional studies that are covered by the token (top right). A summary of study data and metadata is provided under “Metadata” (bottom right). By clicking on “Edit,” the user can edit the study, while clicking on “Manage experimental data” allows the user to up- or download experimental data.

In the MetaboSERV platform, a *study* encapsulates the uploaded experimental data, annotations (such as phenotypes), and metadata (e.g., the study owner and collaborators). The study creation process is represented in Fig. 3A. The user can upload raw experimental data, absolutely quantified metabolite concentration data, or both, and the study must contain mandatory metadata (study authors, at least 1 biospecimen, at least 1 analytical method, and a year or range of years associated with the study). Raw experimental data of any format, bundled with a common tool like gzip or zip, can be uploaded to and retrieved from MetaboSERV. Concentration data, represented as any of the common file formats TSV, CSV (e.g., bucket tables), XLS, or XLSX, are accepted. It is also possible to add an additional file with phenotype data. The concentration and phenotype data files, however, have to adhere to the layout specified by MetaboSERV, as outlined in Supplementary File S3. A validation procedure, which verifies that data fit the requirements by checking the file structure, is performed instantaneously before the data are further processed and added to MetaboSERV. Finally, additional arbitrary metadata can be added by uploading a JSON/YAML metadata file, as detailed in Supplementary File S3. It is also possible to specify metadata directly, which will overwrite any uploaded metadata with conflicting entries.

### Data access control and management

The MetaboSERV platform is built around the notion of collaborative research work [48] and aims to simplify the exchange of experimental data and metadata, irrespective of whether the data are meant to be published or to remain private. Each study can be managed separately, as shown in Fig. 3B. In addition to showcasing phenotypes and any study metadata, it allows to modify the metadata.

In order to guarantee data privacy, 2 different access control mechanisms are implemented for both public and any local MetaboSERV instances. They can be used independently of or in combination with each other. Each MetaboSERV user, who wants to upload data to the MetaboSERV platform, first needs to create an individual user account. Users are required to provide a username, e-mail address, and password, which is stored encrypted (salted and hashed, please refer to Supplementary Section S2 “User Authentication and Password Storage” for more details), to register, and neither their account names nor their personal data are revealed to other users. Upon registration, an e-mail containing a verification code is sent to the e-mail address that was used to register the account. This unique code, which consists of 10 random characters, must be entered once after logging in to unlock any permissions associated with the account, which includes viewing studies shared with the account or uploading studies. The user accounts are associated with access rights for specific studies. For each uploaded dataset, 2 different levels of access rights can be granted to other user accounts—either “read-only permissions” or “full data editing and management authority”—by the original uploader or users with “full data editing and management authority” accounts. The account responsible for the initial creation of the study possesses “full data editing and management authority” at any time and can never be removed as a contributor by any other user. Authentication during the log-in process is handled through JSON web tokens [49] (JWTs), which are associated with each user and newly generated on each log-in. Unauthorized access attempts are continuously logged in the Elasticsearch database. The number of unauthorized access attempts, which occurred since the last authorized log-in, is reported to the user upon every log-in. In the public MetaboSERV instance, an alert e-mail is sent to the user’s e-mail address linked to the account in case the number of unauthorized access attempts exceeds 5 attempts, and the account is locked for 10 minutes after a total of 10 tracked unauthorized access attempts. On a successful log-in attempt, this counter is reset to zero. More information and customization details for local MetaboSERV instances are provided in Supplementary File S2.

Additionally, all users with “full data editing and management authority” have the ability to create authorization tokens. These, in contrast to JWTs, are independent of user accounts. Each token can be associated with an expiration date, a set of permissions for 1 or more datasets, and a comment. Logging in with an authorization token grants the set of permissions specified by the token creator without having to create a user account, as long as the token is valid and not yet expired. This feature is intended to enable short-term sharing of data, such as providing project results to a peer or journal editor/reviewer for validation, by minimizing the effort required to access the data: simply sharing the token will allow the recipient to view—or even edit, if allowed—the selected data.

### Data search and selection

**Figure 4:**
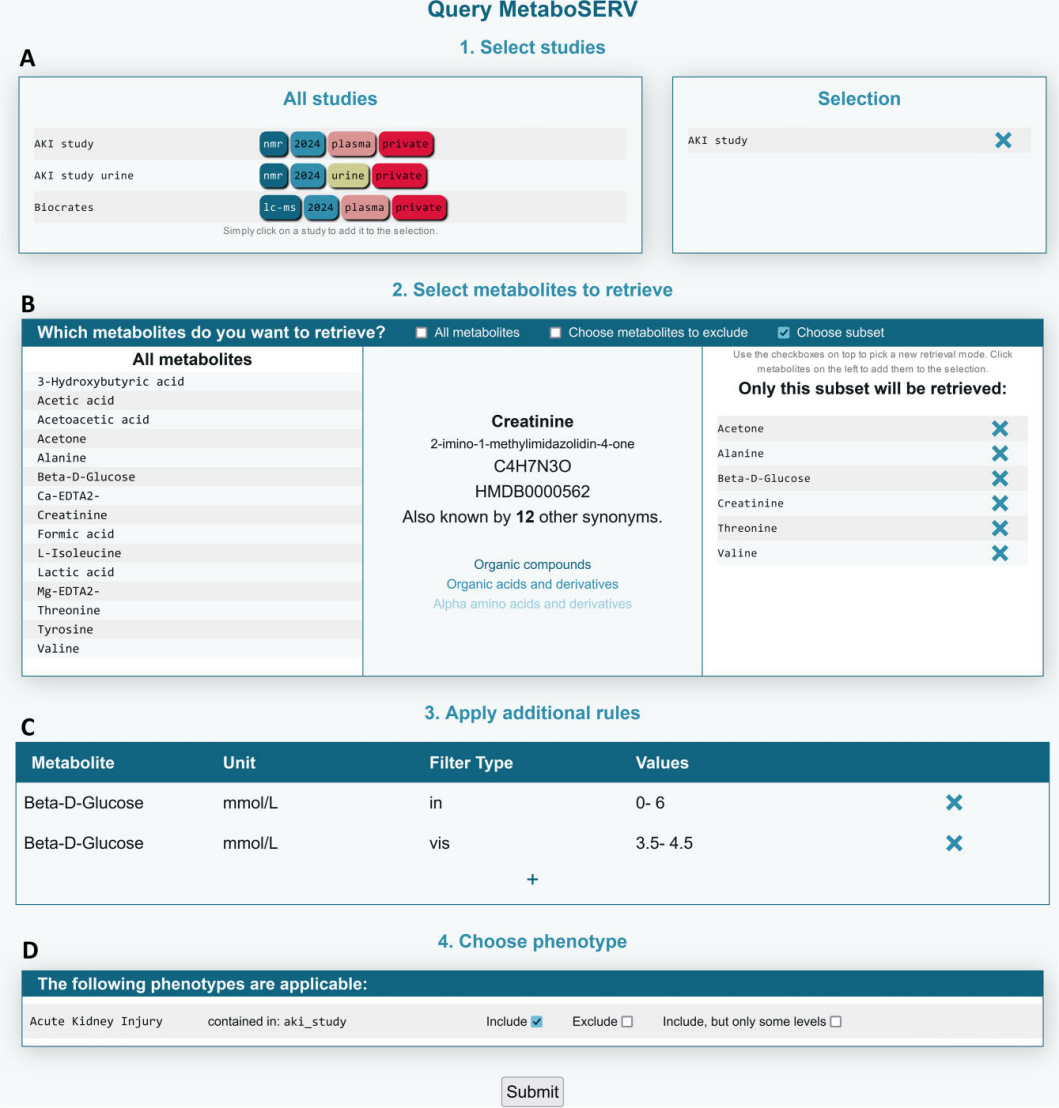
An example query to the public MetaboSERV database. (A) Study selection menu. All accessible studies are shown on the left and can be selected for a query. In this case, only the *AKI study* was selected, consisting of absolutely quantified concentration values of plasma metabolites measured by NMR spectroscopy. (B) Metabolite selection menu. By default, all metabolites are retrieved. If desired, only a particular subset will be retrieved or excluded, respectively. Metabolite information, such as for creatinine, is shown if the user hovers over a metabolite entry. (C) Metabolite filter menu. Specific filtering rules, such as specifying that the concentration level of a particular metabolite has to be included in or excluded from a certain range, can optionally be added here. Metabolite concentration ranges for healthy individuals provided by the HMDB may be used as filter options, too. Alternatively, only a selected concentration range can be visualized without altering the set of results. In this example, all entries with β-D-glucose levels above 6 mmol/L are ignored, while entries with a β-D-glucose level between 3.5 and 4.5 mmol/L will be highlighted. (D) Phenotype selection menu. By default, phenotype data are retrieved completely. A subset of phenotypes or phenotype levels can also be selected.

The core feature of the MetaboSERV platform is a many-faceted, intuitive data selection system that allows for simple and complex queries alike. By configuring search parameters in the web interface, users can specify exactly the data or studies suitable for their research task or use case, thereby filtering out superfluous data. Data can be selected according to studies, metabolites, and phenotypes (Fig. 4), and subsequently either be analyzed further in MetaboSERV or downloaded for other purposes. This also facilitates straightforward integration of different studies or research projects, as data from several datasets can be combined arbitrarily, as long as the user has acquired permission to view the respective data.

Data selection revolves around finding data that fit the physiological and phenotypical criteria outlined by the user. In a first step, users can select all studies they want to include in their query (Fig. 4A). Any number of studies can be combined. Next, metabolites can be specified (e.g., to represent the target metabolic profile) (Fig. 4B). By default, all metabolite concentration levels are retrieved from the selected studies. It is also possible to exclude a subset of metabolites (Fig. 4B). In an optional next step, users can add constraints by specifying individual concentration ranges for the inclusion or exclusion of metabolite levels (Fig. 4C). Additionally, it is possible to supply a range only for visualization purposes (without affecting the query results) by choosing the filter type “vis.” The corresponding metabolite concentration ranges can either be entered by the user or selected from a predefined collection of different reference concentrations from the HMDB [19].

**Figure 5:**
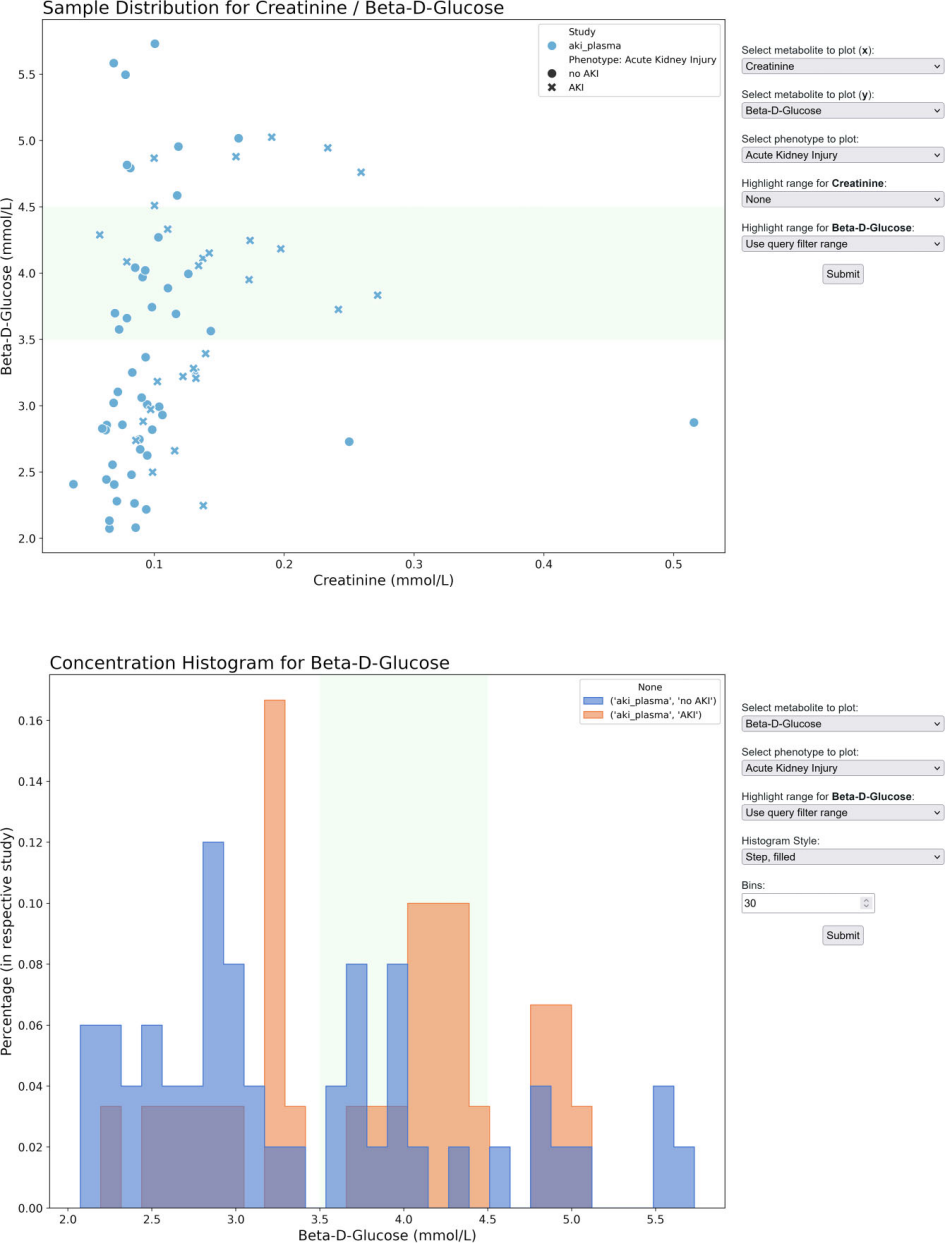
Results of the query pictured in Fig. 4, shown in the form of a sample distribution scatterplot and a concentration histogram, respectively. The metabolites and phenotypes to be plotted can be selected by the user. Specimens with β-D-glucose levels between 3.5 and 4.5 mmol/L are highlighted, as specified in the query in Fig. 4C and by using the “Use query filter range” option in the “Highlight range” field, as seen in the menu on the right.

**Figure 6:**
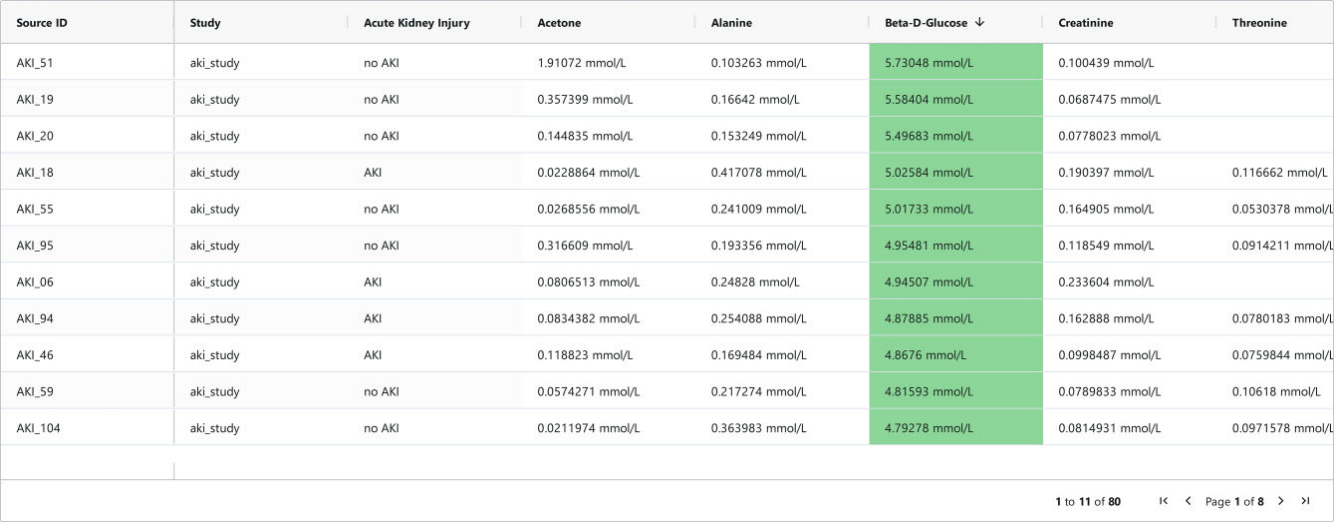
Results of the query pictured in Fig. 4, shown as a table that is sorted according to β-D-glucose levels in descending order. Concentration values that passed the filtering rules set by the user are highlighted in green. As the filtering rules removed patients with β-D-glucose levels above 6 mmol/L, the entire β-D-glucose column is highlighted in green. Phenotypes, such as *Acute Kidney Injury* and the according levels, are also shown. Missing values are represented as empty cells.

In a final step, users can choose to include phenotype data (or only particular levels of a phenotype, such as “healthy”), if such data are available for any of the selected datasets (Fig. 4D). Phenotype data do not directly affect the data selection process but can be used for subsequent data analysis and visualization purposes. Query results can be displayed and browsed in a table (see Fig. 6) or visual representation (as shown in Fig. 5). It is also possible to inspect the underlying experimental data, given that it matches a supported format. Currently, raw NMR free induction decays (FIDs) as well as spectra in the frequency domain in the Bruker file format are supported.

Datasets uploaded to the public MetaboSERV instance, as well as combinations or subsets thereof, can be browsed and visualized in the public MetaboSERV web interface, provided the user may access the respective data. Local MetaboSERV instances are initially set up without any public use case datasets, which are, however, available for download at the public MetaboSERV instance [16]. Histograms, scatterplots, and heatmaps depicting the Pearson correlation between different metabolites (an example is provided in Supplementary Fig. S2) are created automatically. Different subgroups, such as those defined by phenotypes, are also taken into account (see Fig. 5). They can be configured and created on demand for different combinations of nominal phenotypes or metabolites. Concentration values can be displayed in a table, which highlights whether each value is contained in a selected range or not and additionally displays selected phenotype data for each entry (Fig. 6). NMR raw frequency data in the Bruker file format can also be displayed. Finally, MetaboSERV facilitates the automatic creation of quality control plots (see example provided in Supplementary File S4: use case 2 and Supplementary Fig. S3), allowing researchers to quickly gauge the amount of missing values, that is, concentration values below the limit of detection (LOD). Here, the user can create further plots showing the distribution of missing values per metabolite across all measured samples and toggle between showing values over or under the LOD for both types of plots.

### Data retrieval

Research data, which include both public data and private data given the required permissions, can also be retrieved from the MetaboSERV platform. This applies to retrieving any raw data as well as generating new documents containing quantified metabolite concentration data for any study or subsets thereof. Currently, JSON, CSV, and XLSX formats are supported. Created plots are also available for download in PNG format.

### Use case

We demonstrate the capabilities of the MetaboSERV platform in an exemplary use case:

A consortium of clinician scientists, wet-lab metabolomics, and data science researchers carries out a metabolomics study on patients undergoing cardiac surgery. The clinician scientists have sent blood plasma specimens to the metabolomics wet lab for measurement by NMR spectroscopy. Furthermore, the clinician scientists have created a new study on the public MetaboSERV instance, named it “AKI study” (Fig. 3A), and uploaded the corresponding phenotype data and study metadata. They further add the wet-lab metabolomics and data science researchers as “contributors” to the study (Fig. 3B). The former upload the measured experimental data, in this case, both raw and absolute concentration data to this MetaboSERV study. The goal of this metabolomics study is to identify possible associations between postoperative acute kidney injury (AKI) and particularly creatinine as well as β-D-glucose, but also additional metabolites. The statistical analyses are carried out by the data science researchers, who select the “AKI study” as well as the metabolites creatinine and β-D-glucose, as well as acetone, alanine, threonine, and valine in the MetaboSERV query interface (Fig. 4). As the researchers are not interested in effects of extremely high β-D-glucose levels, they filter out any plasma specimen with a β-D-glucose level above 6 mmol/L. They also highlight plasma specimens with β-D-glucose levels between 3.5 and 4.5 mmol/L using a filter of type “vis” (Fig. 4C). Finally, the phenotype “Acute Kidney Injury” is selected. To graphically explore the hypothesized association between AKI and β-D-glucose, the researchers generate 2 different visualizations: a histogram of the β-D-glucose distribution stratified according to AKI diagnosis and a scatterplot depicting the relation between β-D-glucose and creatinine, as elevated blood creatinine levels are a strong indicator for impaired renal function and thus a marker for AKI [50] (Fig. 5). A clear association between higher β-D-glucose levels and AKI diagnosis can be seen in both visualizations. Finally, the wet-lab metabolomics researchers want to show the metabolite concentration data to external collaboration partners. As they only want to provide them with a temporary data access for 7 days without any editing permissions, they generate the corresponding authentication token “exemplary data” (see Fig. 3B) and share them with their collaboration partners. Two additional exemplary use cases, the first demonstrating a quality assessment of mass spectrometry data using MetaboSERV and the second illustrating MetaboSERV’s capabilities of handling a large-scale, multimodal, untargeted mass spectrometry dataset, are provided in Supplementary Sections “3.4  File S4: Use case 2: Quality assessment of mass spectrometry data” and “3.5 File S5: Use case 3: Large-scale, multimodal, untargeted mass spectrometry data.”

## Discussion

MetaboSERV is a browser-based, extensible metabolomics platform with a focus on (absolutely) quantified metabolomics research data from NMR and LC-MS measurements. A major goal of MetaboSERV is to provide researchers with the means to autonomously control access to their metabolomics data. The MetaboSERV platform differentiates between public and private data and offers 2 distinct and independent ways to grant access to private datasets: account- and token-based authentication. Data access can be limited to a customized time frame or revoked at any time by the owner (i.e., the user who originally uploaded the dataset). Thus, MetaboSERV provides the research community with a privacy-preserving platform for exchanging metabolomics data and research findings. It is particularly designed for interdisciplinary collaborations between metabolomics experts, biological or medical scientists, and data analysts in different research settings, such as contract work of a metabolomics core facility, large third-party funded collaboration projects (e.g., within [transregional] collaborative research centers), or institutional, national, and international research collaborations. MetaboSERV supports common data and metadata exchange formats (Bruker, CSV/TSV, XLSX, and YAML, respectively) and is flexible with regard to data formatting, particularly for metadata and phenotypical data. The platform is completely open-source and also offers configurable and portable Docker containers for the purpose of hosting self-managed and self-maintained local MetaboSERV instances in addition to the centralized, public MetaboSERV instance, hosted at MHH. These characteristics allow a seamless integration of MetaboSERV into local (biomedical) data exchange infrastructures. The MetaboSERV platform is efficient in terms of speed and flexible in terms of memory usage thanks to Elasticsearch and MariaDB. It is also extensible to suit the needs of different research institutions: both the functionality and the accepted data formats are designed to be adaptable, and MetaboSERV does not require a specific environment apart from a system containing Docker.

A second major feature of the MetaboSERV platform is to allow users to store and find data suitable for their research projects. In the public MetaboSERV instance, public datasets, such as the AKI study or reference concentration values for metabolites, can be retrieved or combined with available data, enabling complex queries. The MetaboSERV platform also provides visualization options for both raw NMR spectra and summary statistics of absolute metabolite concentrations. While MetaboSERV includes reference concentration ranges for a large number of metabolites from different biofluids provided by the HMDB, arbitrary concentration ranges can likewise be selected. Metadata and phenotypical data for studies and research projects can be kept alongside concentration data, and the latter is also available for database queries.

MetaboSERV fills an important gap in the already established metabolomics data repository landscape: it enables interdisciplinary research collaborations to share their metabolomics experimental, phenotypic, and metadata within a user-friendly platform with fully controlled data access and advanced data browsing and visualization options prior to publication of study results. It provides a user-controlled permission system for uploaded data, which can selectively allow access (both read-only and editorial) to particular datasets for different institutions, researchers, and affiliated peers. Self-managed, local MetaboSERV instances can be created by any user employing the fully configurable Docker images, guaranteeing full autonomy as well as data privacy. These features set MetaboSERV apart from the largest and most widely used metabolomics data repositories, MetaboLights, the Metabolomics Workbench, Metabolonote, and MeRy-B, which are designed to share experimental metabolomics data and/or metadata with completely open access [6–9]. Similar to MetaboLights and the Metabolomics Workbench, MetaboSERV can accommodate experimental data, regardless of species, sample, or analytical method, as well as metabolomics metadata. In contrast, Metabolonote is designed to exclusively hold metabolomics metadata [7], and MeRy-B focuses solely on NMR-based metabolomics plant data [8].

Another difference between MetaboSERV and the metabolomics data repositories discussed above is the rich data selection, visualization, and querying functionality for absolutely quantified data. This selection process allows the combination of different studies and the integration of already published studies into new research projects. COMETS Analytics offers similar selection features and advanced data analysis tools across different studies, but it is designed specifically for standardized meta-analyses of multiple metabolomics studies rather than serving as a metabolomics study repository [11]. Likewise, MetHos focuses on large-scale processing, storage, and analysis of mass spectrometry data, without elaborate, privacy-preserving data-sharing options as provided by MetaboSERV [10]. Furthermore, MetaboSERV allows the recording of methodological metadata without any format and/or content restrictions. To facilitate a low-threshold user experience of MetaboSERV, we implemented a very flexible metadata upload by deliberately not enforcing mandatory metadata specifications. In contrast, established metabolomics data repositories such as MetaboLights and the Metabolomics Workbench face the users with rather strict mandatory metadata as well as experimental data upload requirements.

Besides metabolomics-focused data repositories, a large number of workspaces for data sharing and collaboration with options for privacy preservation have been released in the past decades, including commercial applications like Google Workspace and Nextcloud, as well as a multitude of freely available solutions, including Figshare or Synapse/NF Data Portal [51]. Additionally, academic institutions worldwide have started building their own data-sharing repositories (e.g., the Academic Cloud service for Lower Saxony, or RepoMed, the institutional repository of Hannover Medical School). However, none of these workspaces and solutions are designed to the specific needs of metabolomics data repositories, but rather provide “data-type agnostic” data lakes for the storage and retrieval of individual datasets [51]. In comparison to MetaboSERV, they do not provide smart search functions for metabolites across several, independent studies, have no data analysis or visualization options, and, more importantly, do not support the setup of self-administered, configurable instances, which are completely independent of the providers. The freely available software FAIRDOM-SEEK [52, 53] can be, similar to MetaboSERV, also deployed locally, but it is designed particularly for data spanning multiple omics types or interconnecting datasets and systems biology models [52] and not for metabolomics data. Thus, uploaded datasets cannot be systematically queried or analyzed with respect to individual metabolites and/or across studies.

The demands for privacy-preserving data sharing will further increase with technical advances in metabolic fingerprinting of human individuals, on the one hand, and ongoing, large-scale roll-out of artificial intelligence (AI) for metabolomics data analysis, on the other hand. Analytical sensitivity and specificity of metabolic fingerprints will further increase due to technical progress, potentially facilitating patient reidentification [15]. AI, in particular, demands large, highly standardized (metabolomics) datasets to ensure maximum performance. MetaboSERV can build the basis for data scientists to access and select multiple metabolomics datasets measured at different metabolomics wet labs for subsequent AI-based analysis within a privacy-preserving environment. Its open-source architecture allows full user control, adaptability, and, moreover, seamless integration into already existing research data infrastructures and AI analysis platforms.

## Availability of Source Code and Requirements

- **Project name:** MetaboSERV
- **Project homepage:**  https://gitlab.gwdg.de/MedBioinf/metabolomics/metaboserv
- **Operating system(s):** Platform independent
- **Programming language:** Python, R, TypeScript
- **Other requirements:** Docker, Elasticsearch, MariaDB
- **License:** MIT License
- RRID: SCR_025496

**Minimum installation requirements (recommended):**

- 2 cores
- 100 GB of hard-drive space (SSD recommended)
- 6 GB of RAM

## Supplementary Material

giaf075_Supplemental_File

giaf075_Authors_Response_To_Reviewer_Comments_Original_Submission

giaf075_Authors_Response_To_Reviewer_Comments_Revision_1

giaf075_Authors_Response_To_Reviewer_Comments_Revision_2

giaf075_GIGA-D-24-00275_Original_Submission

giaf075_GIGA-D-24-00275_Revision_1

giaf075_GIGA-D-24-00275_Revision_2

giaf075_GIGA-D-24-00275_Revision_3

giaf075_Reviewer_1_Report_Original_Submission Kevin Murray, Ph.D. -- 10/19/2024

giaf075_Reviewer_1_Report_Revision_1 Kevin Murray, Ph.D. -- 3/24/2025

giaf075_Reviewer_2_Report_Original_Submission javascript:popupReviewerInfo(18892,%207763) -- 11/7/2024

## Data Availability

All data used in this project are available in the *GigaScience* database, GigaDB [54]. The metabolomic data generated in this study are available from the Metabolomics Workbench (study ST003477) [55]. The metabolomic data reused in this study are available from Metabolomics Workbench (study ST002820) [45], MetaboLights (study MTBLS24) [56], and supplementary data of [57]. A version of record snapshot of the GitHub repository has been archived in the Software Heritage Library with the following PIDs: swh:1:snp:02cafba52fe533dddcb9797d58f1645dcb7a284c [58], swh:1:snp:068ba01910b810c5f4628c7858cebc2da1a4ee94 [59], swh:1:snp:33b98aa470f0efc9a464b64f0fe54050de6471ca [60]. A tutorial video for the installation of MetaboSERV is available via YouTube [61], and a copy has been archived in mkv format to the GigaDB dataset [54].

## Additional Files

**Supplementary Fig. S1**. Data model (database schema) of the MetaboSERV Elasticsearch and MariaDB databases.

**Supplementary Fig. S2**. Exemplary heatmap showing pairwise Pearson’s correlation coefficients between different metabolites in the AKI plasma study.

**Supplementary Fig. S3**. Exemplary quality control plot for the Biocrates test data discussed in use case 2.

**Supplementary Fig. S4**. Exemplary query to the public MetaboSERV database discussed in use case 3.

**Supplementary Fig. S5**. Part of the query results of Use Case 3.

**Supplementary Fig. S6**. Scatterplots of Use Case 3.

**Supplementary Table S1**. List of all programming languages and packages used to implement MetaboSERV, as well as their respective versions.

**Supplementary File S1**. Detailed description of MetaboSERV data model.

**Supplementary File S2**. Details on user authentication and password storage.

**Supplementary File S3**. MetaboSERV file specifications.

**Supplementary File S4**. Use case 2: Quality assessment of mass spectrometry data.

**Supplementary File S5**. Use case 3: Large-scale, multimodal, untargeted mass spectrometry data.

**Supplementary File S6**. Detailed installation and user guide for the setup of local MetaboSERV instances.

## Abbreviations

AI: artificial intelligence; AKI: acute kidney injury; API: application programming interface; CPB: cardiopulmonary bypass; CRUD: create, read, update, delete; DSL: domain-specific language; FAIR: findable, accessible, interoperable, reusable; FID: free induction decay; GDPR: General Data Protection Regulation; HILIC: hydrophilic interaction liquid chromatography; HMDB: Human Metabolome Database; JWT: JSON web token; LC-MS: liquid chromatography–mass spectrometry; LOD: limit of detection; MeRy-B: Metabolomics Repository Bordeaux; MHH: Hannover Medical School; NMR: nuclear magnetic resonance; RRID: Research Resource Identifier.
