# Supplementary material for: MetaboSERV—a platform for selecting, exchanging, and visualizing metabolomics data with controlled data access
Source: Gigascience. 2025 Aug 1;14:giaf075. doi: 10.1093/gigascience/giaf075 (PMC12315529; doi:10.1093/gigascience/giaf075)
Supplement: giaf075_Supplemental_File [file giaf075_supplemental_file.pdf]

Supplementary material

MetaboSERV - a platform for selecting, exchanging, and visualizing metabolomics data with controlled data access

Tim Tucholski, Angela Maennel, Yacoub Abelard Njipouombe Nsangou, Sven Schuchardt, Matthias Gruber, Fabian Kellermeier, Katja Dettmer, Peter J. Oefner, Wolfram Gronwald, Michael Altenbuchinger, Jürgen Dönitz, Helena U. Zacharias

1 Supplementary figures

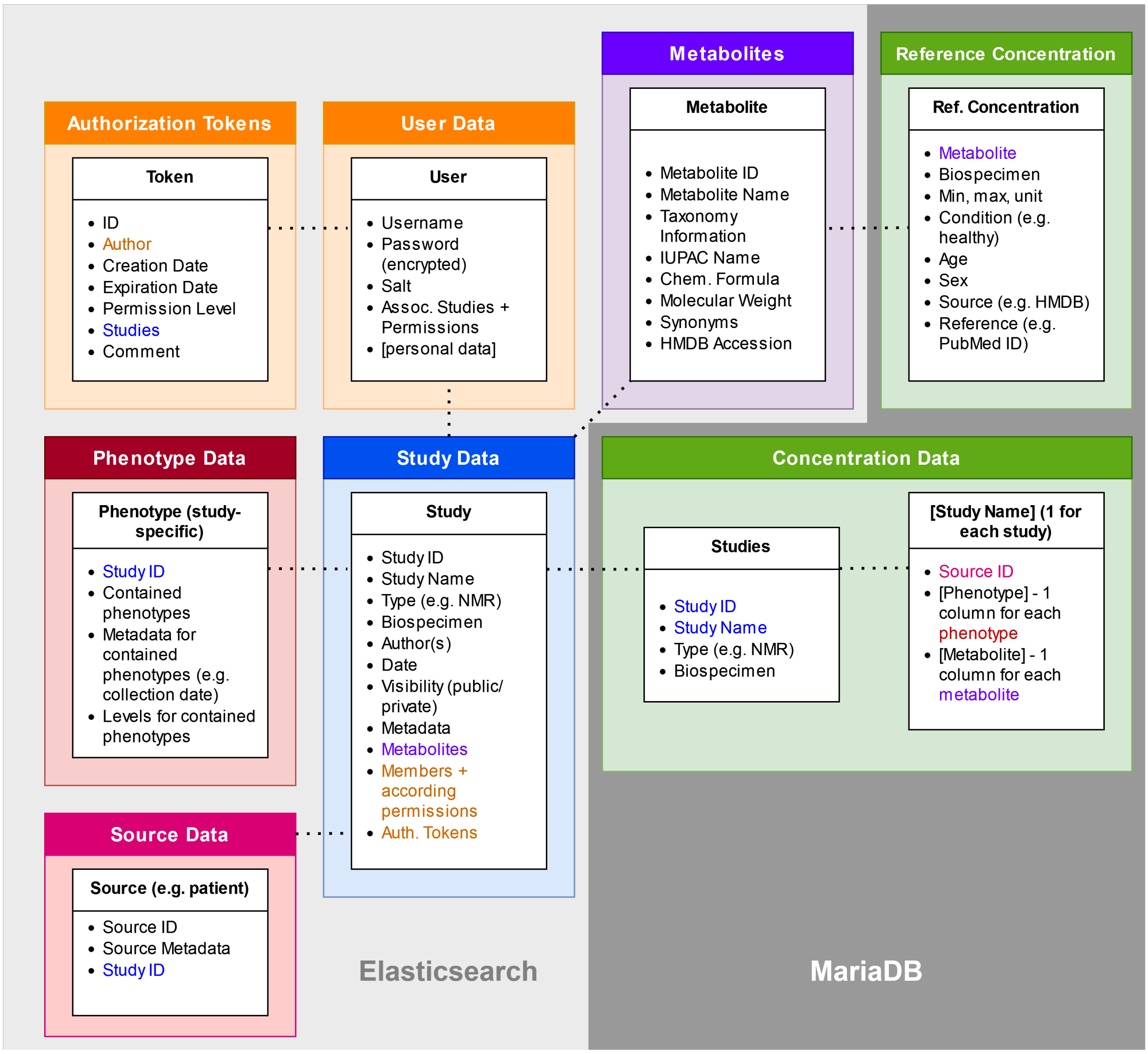

Figure S1: Data model (database schema) of the MetaboSERV Elasticsearch and MariaDB databases. The “Elasticsearch” side displays the six different indices and the associations between them. Each block on the “Elasticsearch” side represents an *Elasticsearch index*. Each index is only populated with one type of document, as described in the corresponding white boxes. Colored blocks on the “MariaDB” side are only conceptional. Each white box on this side represents a table (as MariaDB is a relational database solution) and the described variables represent one row in that table. Colored variables represent “links” or overlaps between tables/indices. Raw experimental data are not stored in either of the databases, but rather directly on the server MetaboSERV is hosted on.

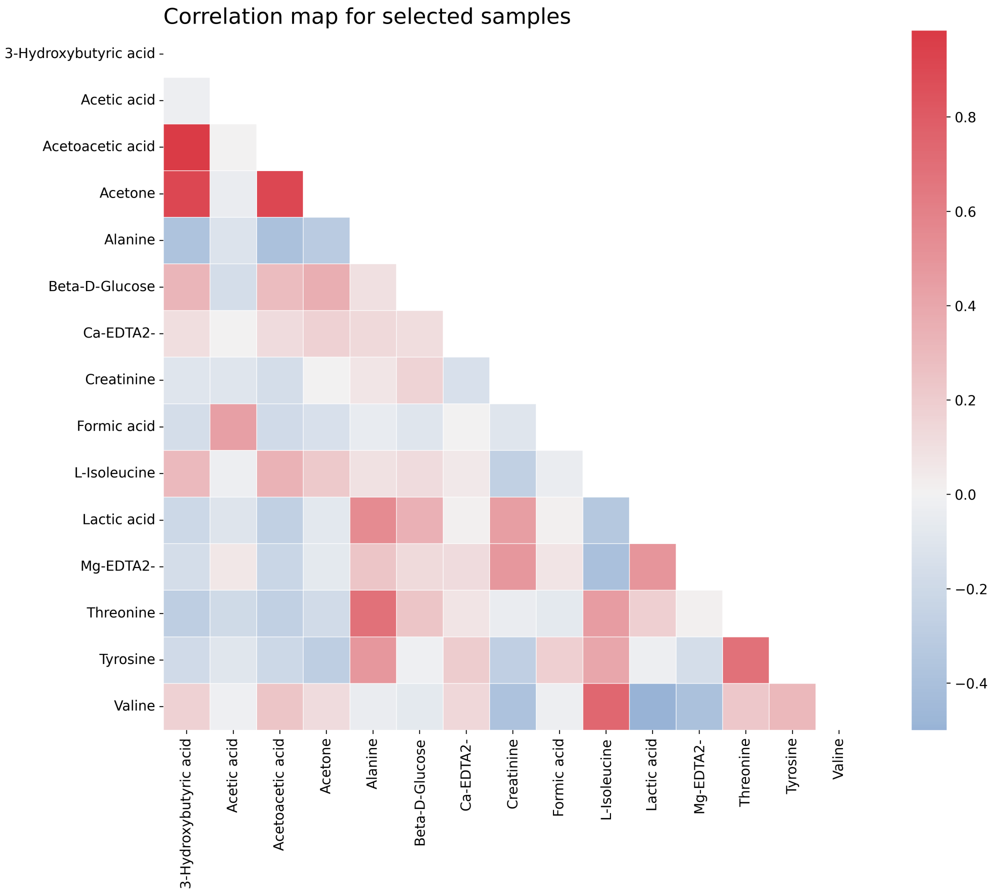

Figure S2: Exemplary heatmap showing pairwise Pearson's correlation coefficients between different metabolites in the AKI plasma study. The color bar on the right indicates the color coding of the strength of the Pearson's correlation coefficients.

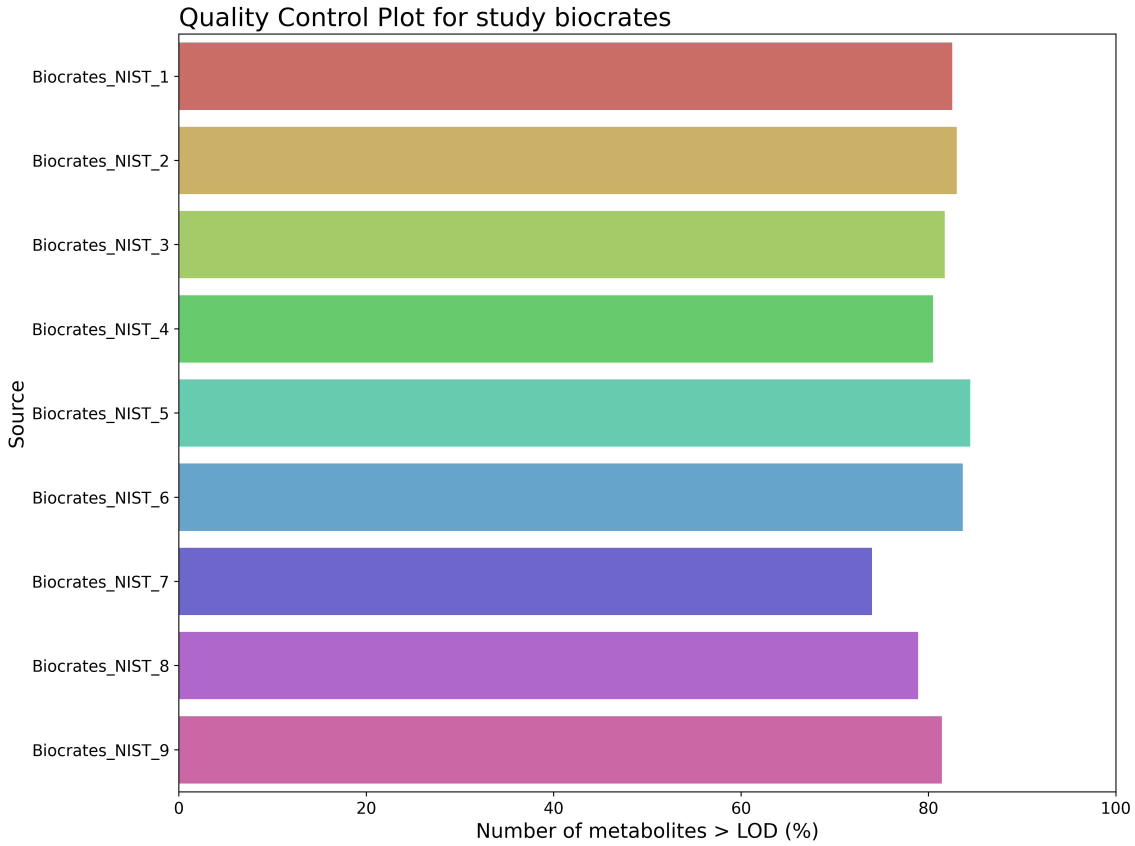

Figure S3: Exemplary quality control plot for the Biocrates Test data discussed in Use Case 2. The overall number of metabolites without missing values equivalent to the number of metabolites with values above the limit of detection (LOD) across the different NIST specimens are shown.

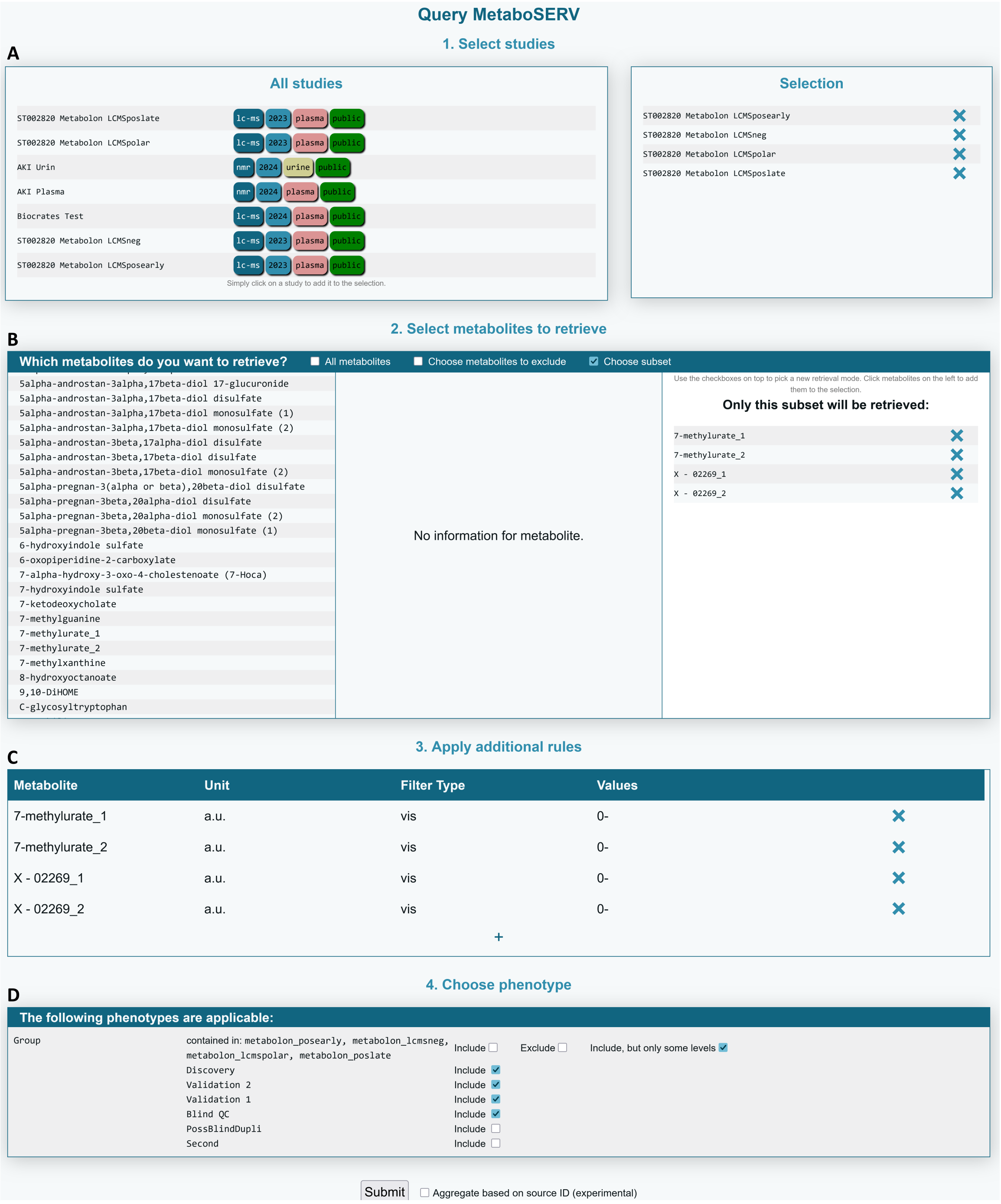

Figure S4: Exemplary query to the public MetaboSERV database discussed in Use Case 3. **A** Study selection menu. The studies “ST002820 Metabolon LCMSneg”, “ST002820 Metabolon LCMSposlate”,“ST002820 Metabolon LCMSposearly”, and “ST002820 Metabolon LCMSpolar”, consisting of untargeted LC/MS data of 1,228 unique metabolites semi-quantitatively measured by Metabolon in 1,002 human blood plasma specimens are retrieved. **B** Metabolite selection menu. A subset of four metabolites, namely ‘7-methylurate_1’, ‘7-methylurate_2’, ‘X - 02269_1’, and ‘X - 02269_2’ are selected. **C** Metabolite filter menu. For all four metabolites, measured in arbitrary units (a.u.), the Filter Type ‘vis’ is applied. **D** Phenotype selection menu. Only the phenotype levels ‘Discovery’, ‘Validation 1’, ‘Validation 2’, and ‘Blind QC’ are selected.

Source ID	Study	7-methylurate_1	7-methylurate_2	X - 02269_1	X - 02269_2	Group ↑
META-96843	metabolon_posearly		63361.797 a.u.		6569484.5 a.u.	Discovery
META-96845	metabolon_posearly				723525.5 a.u.	Discovery
META-96846	metabolon_posearly				1073004.625 a.u.	Discovery
META-96848	metabolon_posearly		66113.367 a.u.		1207107.375 a.u.	Discovery
META-96849	metabolon_posearly		177703.328 a.u.		2262345.75 a.u.	Discovery
META-96850	metabolon_posearly		190429.125 a.u.		4509315 a.u.	Discovery
META-95838	metabolon_lcmsneg			14756424 a.u.		Discovery
META-95842	metabolon_lcmsneg			13849085 a.u.		Discovery
META-95843	metabolon_lcmsneg			3702497 a.u.		Discovery
META-95844	metabolon_lcmsneg	1046539.25 a.u.		693650.688 a.u.		Discovery
META-95848	metabolon_lcmsneg	79322.922 a.u.		5532982.5 a.u.		Discovery

551 to **561** of **2,000** Page **51** of **182**

Figure S5: Part of the Query Results of Use Case 3. The table is sorted according to the phenotype variable 'Group' and metabolites within one respective study, for which at least one value is contained in the filter selection specified in Supplementary Figure S4 are highlighted in green. Metabolites within one respective study, for which no value is contained in this filter selection, are highlighted in red. Overall, metabolites with values below the limit of detection/missing values are represented as empty cells.

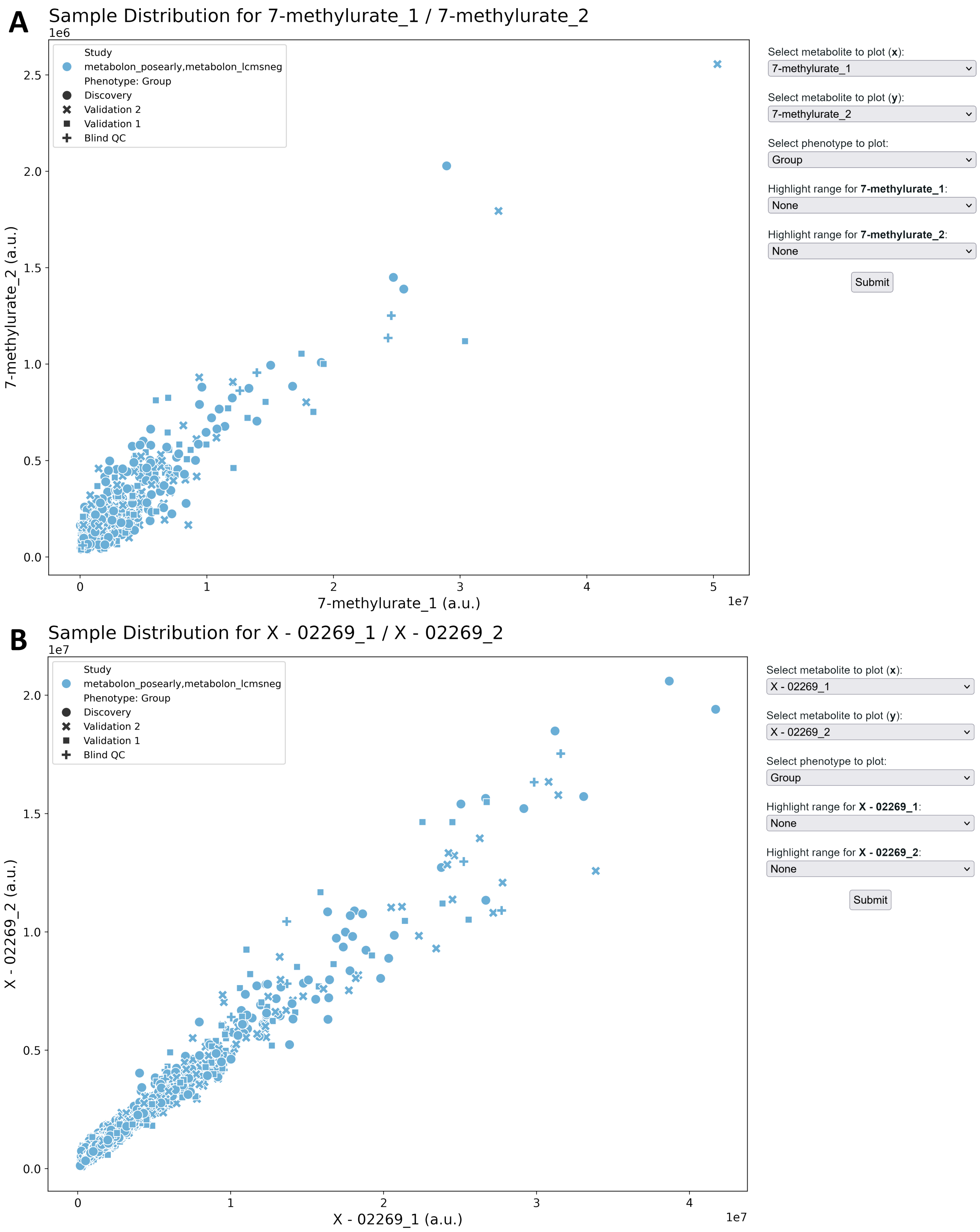

Figure S6: Scatter plots of Use Case 3. **A** Scatter plot of the metabolites '7-methylurate_1' and '7-methylurate_2' in arbitrary units (a.u.). **B** Scatter plot of the metabolites 'X - 02269_1' and 'X - 02269_2' in arbitrary units (a.u.).

2 Supplementary tables

Package	Version used	Package	Version used
R [1]	4.2.1	flask-cors [2]	3.0.10
mrbin [3]	1.7.2	flask-jwt-extended [4]	4.4.4
restRserve [5]	1.2.1	flask-swagger-ui [6]	3.36.0
TypeScript [7]	4.5.5	numpy [8]	1.23.5
VueJS [9]	3.2.13	matplotlib [10]	3.6.2
Pinia [11]	2.1.7	seaborn [12]	0.11.2
AG Data Grid [13]	27.4.0	pandas [14]	1.5.2
Python [15]	3.9.15	xmltodict [16]	0.12.0
flask [17]	2.0.2	werkzeug [18]	2.0.3
xlrd [19]	2.0.1	pytest [20]	8.1.1
email-validator [21]	2.2.0	elasticsearch [22][1]	7.13.3
magic [23]	0.4.27	mariadb [24][2]	1.1.9
dropzonejs [25]	6.0.0	gunicorn [26]	23.0.0

Table S1: List of all programming languages and packages used to implement MetaboSERV, as well as their respective versions. [1]Refers to the Python Elasticsearch client. [2]Refers to the Python MariaDB connector.

3 Supplementary files

3.1 File S1: MetaboSERV data model

Fig. S1 shows the data model and database schema, respectively, of the data contained in the MetaboSERV platform. MetaboSERV leverages schemaless data storage provided by Elasticsearch, which partitions data into *indices*. There are six Elasticsearch indices in total, each corresponding to a different category of data. Furthermore, a number of tables exist in the MariaDB instance: One for reference concentrations, one internal mapping table, and one additional table for each study that was uploaded to the MetaboSERV platform.

3.1.1 Studies and sources

MetaboSERV is set up to maintain a collection of **studies** (marked in blue). Each study object - such as the AKI study - is associated with a particular set of experiments and relevant metadata attributes such as the study authors, one or more biospecimens, the publication date or relevant filenames, as well as a unique study ID. The user provides a unique study ID, which is automatically compared to all existing study identifiers of the used MetaboSERV instance which are stored in both the MariaDB and Elasticsearch database. In the case a new study ID is already stored in the databases, a conflict message is shown and the user is asked to provide a new study ID. A study can be seen as a unique research project, or as one part of a larger research project involving several studies. The entities that were investigated in a study are referred to as **sources**. A typical source would be a patient in a medical study, but the term is intentionally avoided as metabolomics studies can also deal with other specimen sources such as plants, foods, cell lines, animals or specific organs. The source index is marked in magenta in Fig. S1.

3.1.2 Concentration data

Both metabolite concentration levels uploaded by a user, as well as reference concentrations from the HMDB are stored in MariaDB, highlighted in green. There is one table for reference concentrations, one table that serves as a list of existing studies, as well as one table per study which contains the actual concentration levels.

3.1.3 Phenotypical data

Phenotypical data are stored in an Elasticsearch index, shown in red in Fig. S1. Each phenotype object (which stores the levels and phenotype data for all sources) is study-specific, but if a phenotype occurs in several studies they can still be linked using an internal phenotype identifier. Experimental metadata, i.e. data on the assessment of the phenotype, is also stored in the same index.

General metabolite data, such as taxonomy information, IUPAC name or synonyms are stored in a separate index, highlighted in purple.

3.1.4 User data and access tokens

Finally, there are two indices for **user data** and **access tokens** (depicted in orange). Each user object is associated with a username, e-mail address, password (stored encrypted), salt (used for checking against the password) and a set of permissions for specific studies. Metadata that can be provided when an account is registered, such as the affiliated institution, are stored as well.

Access tokens are associated with an author, a set of permissions the token provides when attached to the URL, the studies the permissions are set up for, an expiration date and optionally a comment.

3.2 File S2: User Authentication and Password Storage

When a user account is created, the password that the user chooses is safely sent to the respective MetaboSERV instance over HTTP-Secure (HTTPS). There, a random 64 byte salt (interpreted as a 128-character hexadecimal character string) is prepended to the password and both are encrypted using 10000 iterations of the Password-Based Key Derivation Function 2 (PBKDF2), using SHA-256 as a hashing algorithm. This makes it computationally infeasible to retrieve the original password from the encrypted string, even if access to the database (which is not accessible to external clients) should

be obtained. Only the encrypted password and the salt are stored in the MetaboSERV database for authentication purposes. Apart from the salt and encrypted password, only a small amount of user data, comprising the account name, e-mail address and, optionally, the institution, is stored.

In order to fully activate an account, the MetaboSERV platform uses an e-mail verification system, which can optionally be disabled for self-managed MetaboSERV instances. Upon registration, an e-mail containing a verification code is sent to the e-mail address that was used to register the account. This unique code, which consists of ten random characters, must be entered once after logging in to unlock any permissions associated with the account, which includes viewing studies shared with the account or uploading studies. Each code is valid for 24 hours or until a new code is generated. Failed login attempts are tracked and displayed to the user when they successfully log in. After a default number of five failed attempts, an e-mail reporting potentially suspicious activities is sent to the e-mail address associated with the account. After a default number of ten failed attempts, the account is temporarily locked for ten minutes. The number of attempts for both of those events can be configured for self-managed instances. A password recovery service allows the recovery of lost passwords associated with an account by employing the associated e-mail address. The e-mails are sent using SMTP by a function e-mail account, which has to be provided by administrators of self-managed MetaboSERV instances, should they choose to use this feature.

3.3 File S3: MetaboSERV file specifications

The following section describes the layout that uploaded concentration and phenotype data files in a table format must adhere to in order to be accepted by the MetaboSERV platform. MetaboSERV assumes each row of a file to represent a source/patient and each column of a file to represent data associated with the source (such as a metabolite name/identity or a patient identifier) by default. The following specifications are also stated with this standard in mind. For convenience, MetaboSERV offers a *transpose* feature upon study creation that inverts the parsing of rows and columns - meaning each column would represent one source and each row would represent data associated with the source instead. Templates for 'Concentration data', 'Phenotype data', as well as 'metadata'-YAML-files are provided for download on the public MetaboSERV landing page at `https://metaboserv.ckdn.app/`. Furthermore, complete example data files for the three use cases are provided on the same webpage.

3.3.1 Concentration data

MetaboSERV supports concentration data provided in CSV, TSV, XLS or XLSX table file format. A concentration data file must contain one column per metabolite (but at least one) and may contain an additional column for source/patient identifiers. A header row with metabolite names - or the term `source_id` must therefore be provided. Up to 1,016 metabolites can be provided per study. The unit of measurement for each metabolite can be indicated by adding an extra `unit` column and providing the unit for each metabolite in that column. To avoid the creation of misleading plots, MetaboSERV, however, does not allow the creation of multi-study-plots featuring the same metabolite measured in different units. To facilitate such analyses, the user needs to match the respective concentration units by converting them appropriately prior to data upload to MetaboSERV. If no unit is indicated, MetaboSERV assumes mmol/L by default. If no source identifier column is provided, ascending numerical values will instead be used to identify each source. Metabolite identifiers are treated as case-insensitive and checked against a list of synonyms obtained from the Human Metabolome Database (HMDB). Note that this does not exclude any metabolite from being added to the database, even if the corresponding HMDB information is not available; any unknown metabolite will be added to the database the first time it is encountered.

The public MetaboSERV platform does not enforce the use of chemical identifiers for metabolite names as not to impede the upload of metabolomics experiments containing unknown/unidentified metabolites. However, to further ease seamless matching of metabolite names across different studies, MetaboSERV offers the optional, semi-automatic mapping of metabolite names to HMDB identifiers directly on the public MetaboSERV instance website prior to data upload. For this mapping process, we use data provided by the HMDB as well as The Chemical Translation Service (CTS) by G. Wohlgemuth et al. [27]. An enforcement of HMDB identifiers can optionally be used for self-managed, local MetaboSERV instances and can be toggled on or off at any point in time without retroactively affecting already uploaded studies. Enforcing HMDB identifiers will require users to either provide

HMDB identifiers instead of metabolite names, or optionally have them mapped to the respective HMDB identifiers during the study creation process.

Each row represents one source and should contain the according metabolite concentration levels for each metabolite, as well as (optionally) a unique identifier for the source. There are only three types of permissible values for concentration levels: Firstly, a numerical value, indicating the metabolite concentration for the source in mmol/L or the unit of measurement supplied in the header row. Floating point numbers (such as 0.5) must be separated by a period symbol. Secondly, a field can be empty, which would indicate a missing value. Thirdly, the special string $<LOD$ is permitted, which will be treated as a missing value, as well.

The column separator for CSV files must be one of the four accepted choices, namely the tabulator, semicolon, comma or dollar symbol, and must be correctly indicated to MetaboSERV during study creation (see Fig. 3A). If an XLS or XLSX file is provided, only one sheet should be used as MetaboSERV currently does not support the use of several sheets and will consequently ignore any sheet beyond the first one.

3.3.2 Phenotype data

Phenotype data, similar to metabolite concentration data, can be provided in CSV, TSV, XLS and XLSX table formats. There are two mandatory columns: One denoted as `id` or `source_id`, which should contain source identifiers, and at least one phenotype column, which should contain the levels of that phenotype (e.g., *healthy* and *diseased*). Phenotype columns have to be prefixed with the string *PHENOTYPE_*, followed by a descriptor for the phenotype, such as *Acute Kidney Injury*, in order to be treated as such.

All other columns are treated as metadata regarding the collection of the particular phenotypes (such as sample collection dates) and will be stored in a separate index (see Fig. S1).

3.3.3 Study metadata

Arbitrary metadata for the study can be provided in the form of a YAML file. It is recommended to set the mandatory metadata attributes such as study authors, specimen type, etc. (refer to Fig. 3A), directly. Any additional metadata can be given in the form of YAML objects and will be stored in the MetaboSERV database, and shown in the MetaboSERV interface. Each object must have a unique name, such as *instrument*, and contain one `name` and one `value` property. The `name` property specifies a descriptor for the `value` property - for example, a YAML object describing the spectrometer used in an experiment could look like this:

```yaml
instrument:
  name: Spectrometer
  value: 600 MHz Bruker Avance III
```

3.3.4 Raw experimental data

As mentioned in the section "Data upload and processing", MetaboSERV makes no assumptions about the contents of provided raw experimental data. However, it expects one of the following file archive formats: ZIP, GZ, TAR/TAR.GZ, 7Z or RAR. Files that do not match any of the aforementioned formats will not be accepted.

3.4 File S4: Use case 2: Quality assessment of mass spectrometry data

In a second use case, we demonstrate the capabilities of MetaboSERV for metabolomics data quality assessment by an interdisciplinary research consortium:

A wet-lab metabolomics and a data science group conduct a data quality assessment study to examine the performance variation of the triple quadrupole mass spectrometer that they plan to use for a large-scale human plasma metabolomics study employing the MxP Quant 500 kit. To this end, the wet-lab metabolomics group prepares and measures 9 different MxP Quant 500 well-plates, each including one NIST SRM 1950 plasma specimen from the same NIST SRM 1950 aliquot. The group creates a new study on the public MetaboSERV instance, names it "Biocrates" (see Fig. 4A) and

uploads the absolutely quantified metabolomics data. They add the data science group as "contributors" to the study, who will analyse the performance variation in MetaboSERV as follows: For each NIST plasma specimen 630 different metabolites have been measured and absolutely quantified, whereas the data also includes a certain number of missing values across metabolites and samples alike due to biological and/or technical reasons common in mass spectrometry metabolomics data [28]. A non-uniform distribution of the total number of missing values across the 9 different NIST plasma specimens would be an indicator for a performance variation of the sample preparation procedure or the mass spectrometer measurements across the 9 different MxP Quant 500 well-plates. The data science group selects the Biocrates study in the MetaboSERV Browse menu. In a next step, the data scientists select the 'Quality Control Plot (by source)' procedure for all metabolites '>LOD' and click on 'Execute'. They obtain the distribution of metabolites without missing values, equivalent to the number of metabolites with values above the limit of detection (LOD), in percent across the different well-plates displayed as a bar plot shown in Supplementary Fig. S3. The data set seems to have a rather uniform distribution of metabolites without missing values around 80% (corresponding to approx. 20% missing values) across all NIST plasma specimens, except for NIST plasma specimen 7, which exhibits a slightly lower number of metabolites without missing values (corresponding to a higher number of metabolites with missing values). The data scientists download the analysis results and send them to their wet-lab collaboration partners to take further action.

3.5 File S5: Use case 3: Large-scale, multi-modal, untargeted mass spectrometry data

In a third use case, we demonstrate the application of MetaboSERV on a large-scale, multi-modal, untargeted mass spectrometry data set, a typical scenario for major epidemiological studies:

An epidemiological research group would like to share a data set consisting of 1,228 unique metabolites semi-quantitatively measured by Metabolon in 1,002 human blood plasma specimens as well as the corresponding phenotype data on discovery/validation as well as quality control sample cohorts with two international collaboration partners. As these metabolites had been measured by four different LC/MS methods, they divide the data set into four subsets: (1) the LCMSneg subset, comprising 692 metabolites analyzed using basic negative ion optimized conditions, (2) the LCMSposearly subset, comprising 284 metabolites analyzed using acidic positive ion conditions, chromatographically optimized for more hydrophilic compounds, (3) the LCMSposlate subset with 173 metabolites analyzed using acidic positive ion conditions, chromatographically optimized for more hydrophobic compounds, and (4) the LCMSpolar subset consisting of 79 metabolites analyzed via negative ionization following elution from a HILIC column [29]. For each data subset, they have created separate YAML files comprising individual experimental metadata. As the metabolites have only been semi-quantitatively measured, the epidemiological researchers specify the unit as "a.u.", i.e., arbitrary unit, in the concentration files. They upload each concentration and corresponding YAML file separately, which takes between 20 to 90 seconds depending on the amount of contained metabolites. They add their collaboration partners as "contributors" to the study.

The collaboration partners log into MetaboSERV and select all four studies, "ST002820 Metabolon LCMSneg", "ST002820 Metabolon LCMSposlate","ST002820 Metabolon LCMSposearly", and "ST002820 Metabolon LCMSpolar" in the 'Query' browser. They scroll through the list of metabolites, alphabetically ordered across all four studies, and realize that the unidentified metabolite 'X - 02269' appears twice in the list, one time with the name 'X - 02269_1' and one time with the name 'X - 02269_2'. The same holds true for the metabolite '7-methylurate', which appears twice in the list. As the collaborators would like to understand why these two metabolites each appear twice in the list, they retrieve only these four metabolites in the 'Metabolite Selection' menu by selecting 'Choose subset', shown in Supplementary Figure S4. Furthermore, they choose the 'Filter Type' 'vis' for all four metabolites and for the phenotype information, only include the levels "Discovery", "Validation 1", "Validation 2", and "Blind QC". They hit the 'Submit' button without aggregation. In the 'Query Results' table, they order the results according to the provided phenotype variable, scroll through the individual Source IDs and realize that '7-methylurate_1' and 'X - 02269_1' are only available in the "LCMSneg" study, and '7-methylurate_2' and 'X - 02269_2' are only available in the "LCMSposearly" study (Supplementary Figure S5). As they are curious about the relationships between the respective metabolites, the collaboration partners first visualize the association between '7-methylurate_1' and '7-methylurate_2' in a scatterplot by selecting these two metabolites (Supplementary Figure S6A). In a second step, they perform the same analysis for the metabolites 'X - 02269_1' and 'X - 02269_2'

(Supplementary Figure S6B). In both scatterplots, a high correlation between the respective metabolites can be detected, but it becomes also apparent, that the intensity scales seem to differ between the two LC/MS methods. The collaboration partners contact the epidemiology research group and point out their observations in order to discuss further data preprocessing steps.

3.6 File S6: Detailed installation and user guide for the set-up of local MetaboSERV instances

This guide is provided on the MetaboSERV GitLab repository, located at `https://gitlab.gwdg.de/MedBioinf/metabolomics/metaboserv`, where it is included both in the backend and frontend projects, as well as on the public MetaboSERV instance website `https://metaboserv.ckdn.app/`.

3.6.1 Installation

This installation guide covers both the MetaboSERV frontend, which can be downloaded from `https://gitlab.gwdg.de/MedBioinf/metabolomics/metaboserv/frontend`, and the MetaboSERV backend, which can be downloaded from `https://gitlab.gwdg.de/MedBioinf/metabolomics/metaboserv/backend`. The frontend can not adequately be used without the backend.

MetaboSERV is recommended to be used with Docker or Podman. You can also install everything natively, but this will also not be covered here. We recommend a system with *at least* two cores and 6GB of RAM (but ideally more) to install MetaboSERV.
First, create a directory for MetaboSERV and download the required additional files `https://owncloud.gwdg.de/index.php/s/oZEH5eFMLrUXv4M`. Put the compose file (*docker-compose.yml*) and the SQL script (*create_database.sql*) into the newly created directory, which will be referred to as the **"project root directory"** further on.

Compose-file

The compose-file, *docker-compose.yml*, controls different parameters associated with the MetaboSERV application. Unless your environment demands it, it is recommended to leave ports, service names, volume names and network names unchanged.

The following file paths are **mandatory** to adjust. On Windows, remember to double each backslash symbol, e.g. `C:\\some\\host\\path`.

- In the `metaboserv-backend:volumes` section, there are two host paths that need to be adapted. These host paths must be readable and writeable directories on your host system, which are then mounted into the MetaboSERV backend Docker container.
- The first one is mapped to the internal container path */app/files* . This resembles the directory where all experimental data uploaded to MetaboSERV will be saved. Set a sensible path on your host system here, such as */mnt/metaboserv* for external network storage (recommended, but local paths on the server will work fine).
- The second one is mapped to the internal container path */app/data*. It is intended for additional non-experimental data that MetaboSERV can use to provide extra functionality, for example HMDB reference concentrations. Set an according path on your host system here.
- In the `metaboserv-db:volumes` section, replace */your/project/root/create_database.sql* with the path to the *create_database.sql* file you downloaded earlier, which should be in your project root directory. The reason this is necessary is that Docker prefers absolute host paths to relative ones.

The following parameters can **optionally** be adjusted:

- In the `metaboserv-es` section: `ES_JAVA_OPTS` for Elasticsearch - indicates how much RAM Elasticsearch will use. The default is 4GB, i.e. 4096MB.

- The MariaDB login information can be changed in the section `metaboserv-db:environment`. Make sure to adapt any changes in the configuration for the backend service later on.

- If you wish to use the Bruker NMR visualization service based on R, uncomment the `metaboserv-raw` section. You may notice there is another volume here, mapped to the internal path */app/files*. Please replace this directory by the same directory you already used for */app/files* in the `metaboserv-backend:volumes` section.

Frontend

1. In your terminal, navigate to the project root directory and clone the frontend git project located at `https://gitlab.gwdg.de/MedBioinf/metabolomics/metaboserv/frontend`. You should now have a folder called *frontend* in your project root directory, navigate into it.

2. The frontend folder contains a file called `.env.template`. Copy this file and rename the copy to `.env`. This local copy will not be overwritten by updates in the repository. Edit the *.env* file and set the URL where you want/expect the backend service to be. By default, it is set to `http://localhost:9201`, so if the backend will run on the same machine as the frontend and you want to keep the default backend port (9201), you can skip this step - otherwise adapt the host and port according to your system. Note that you will have to use a public URL that can be resolved by a browser here in production.

3. Build the frontend container (from the according *frontend* directory; please make sure that Docker is running before executing the command):

   ```
   docker build -t metaboserv-frontend .
   ```

   If you ever wish to change the backend URL value in the *.env* file, you will have to rebuild the Docker image using the same command.

4. You can also change the name of the frontend container, make sure to also change it in the provided *docker-compose.yml*.

Backend

1. Navigate to the project root directory, clone the backend git project located at `https://gitlab.gwdg.de/MedBioinf/metabolomics/metaboserv/backend` and then navigate into the new *backend* folder.

2. The backend folder contains a file called `metaboserv_conf_docker_template.yml`. Copy this file and rename the copy to `metaboserv_conf_docker.yml`. This local copy will not be overwritten by updates in the repository. Configure *metaboserv_conf_docker.yml* to your needs. Here is a quick rundown of your configuration options (anything not mentioned is recommended to be left unchanged):

   - In the `flask` section: It is recommended to leave the top four values unchanged. Definitely change `jwt_key` to a long character string of your liking, as this is the string of characters used to generate JSON web tokens. If someone knows this key, they can generate login tokens for your MetaboSERV instance. In production, set `jwt_https` to true in order to only accept JWTs over HTTPS. You can change the JWT expiration time (`jwt_expiration`), which is given in hours, if you wish.

   - In the `backend` section, you should change the `admin_password`. This is the password for the *admin* account on MetaboSERVs web interface, which is created automatically and has special permissions. The parameter `enforce_hmdb` dictates whether or not HMDB identifiers will be enforced for your MetaboSERV instance. The other three parameters in this section should not be changed unless you wish to change the container-internal filepaths.

   - In the `mariadb` section, you have to change the `password` to the same password that you set in *docker-compose.yml* for the MariaDB login information. There, it can be found in the section `metaboserv-db:environment` as the environment variable `MYSQL_PASSWORD`.

- The section `smtp` deals with mail verification. If you do not wish to use mail verification, simply set `verification` to *False* (the other SMTP parameters do not matter in this case). Otherwise, pass a valid function `email` address, `password`, SMTP `server` and SMTP server `port`. Independent of the verification system, you can also set the amount of failed login attempts to trigger an email notification (`notify_attempts`) or to trigger a temporary account lock (`lock_attempts`). If you do not wish to use a feature, simply set it to 0. Note that the `notify_attempts` feature does require the SMTP parameters, i.e. a function mail account, to be set.

3. Once you are satisfied with the configuration file, build the backend container (from the according *backend* folder):

```
docker build -t metaboserv-backend .
```

You can change the parameters in *metaboserv_conf_docker.yml* at any point, you will however need to rebuild the Docker image using the same command.

4. If you wish to use the Bruker NMR visualization service (and have already adapted *docker-compose.yml* accordingly), navigate back to the project root directory and clone the according GitLab project (*nmr-parser*) from `https://gitlab.gwdg.de/MedBioinf/metabolomics/metaboserv/nmr-parser`. Navigate into the new directory *nmr-parser* and build the service:

```
docker build -t metaboserv-raw .
```

5. Start up MetaboSERV by running

```
docker compose up -d
```

or

```
docker-compose up -d
```

if you are running an older version of Docker. You can omit the `-d` flag if you don't want the containers to run in the background. This command will pull some Docker images (which requires internet access) if they are not already present. If your machine does not have internet access, consider the use of

```
docker save -o archived_image.tar [IMAGE-NAME]
```

on a server with internet and (on the server where MetaboSERV should later run, after moving the archived image there)

```
docker load < archived_image.tar
```

Refer to the Docker documentation (`https://docs.docker.com/reference/cli/docker/image/save/`) for more information.

6. Verify that all four (five if you chose to install the *nmr-parser*) containers are running using

```
docker ps
```

7. Now, some final configuration steps have to be performed. In your browser, navigate to the SwaggerUI/OpenAPI instance that comes with MetaboSERV. By default, this can be found at `http://localhost:9201/metaboserv-api/doc`. Scroll down to the `login` endpoint in the section "Authentication and Authorization", open it and click *Try it out*. Where it says `string`, enter the username "admin" and the password you set in the backend configuration, respectively. Click *Execute*. Scroll down to the Response body, there should be a line like `"access_token": "XXXXX"`. Copy the long line of characters (without quotation marks).

8. Scroll all the way up. On the right side of the screen, there is an *Authorize* button. Click it, paste the character string you copied in here and click *Authorize* again. You are now acting as the admin account and have necessary permissions to perform administrative tasks.

9. The first endpoint, at the very top, is called `/ESDatabaseInitialize`. Click on it, then click *Try it out* and click *Execute*. This will initialize the Elasticsearch database.

10. MetaboSERV is now set up for use! You can also add HMDB reference concentrations and synonyms, which come in XML files you can download from their FTP servers at `https://hmdb.ca/downloads`. Here is an example for the *serum_metabolites.xml* file, which for this purpose has to be put in the directory you specified during the configuration of the compose-file (the one mapped to */app/data*). Once the HMDB file is in the correct folder, scroll down to the `CompoundAddFromHMDB` endpoint, which you can find in the "Compounds" section. Click it, then click *Try it out* and type the name of the HMDB file (e.g. *serum_metabolites.xml*) into the `filename` text field. *Click Execute*. This may take a while, but should eventually return a "Success" response.

Uninstalling MetaboSERV To uninstall MetaboSERV, you need to remove the Docker containers and volumes as well as any files associated with MetaboSERV stored on the host system. Bring up Docker images using the command

```
docker ps
```

which should show the four to five MetaboSERV images (depending on whether the NMR Parser was installed or not). Remove each image using the command

```
docker rmi [IMAGE-ID]
```

where `IMAGE-ID` is the identifier the image was saved under. Once all images are removed, use the command

```
docker system prune
```

to remove the volumes. Delete the directory where you installed MetaboSERV (making sure to back up any files you want to keep beforehand) to complete the process.

3.6.2 Using MetaboSERV

This usage guide assumes that you either have access to a running MetaboSERV instance, or that you have successfully installed and set up both the MetaboSERV backend and the MetaboSERV frontend as well as the required databases yourself.

Starting MetaboSERV

1. In the MetaboSERV project root directory, run

   ```
   docker compose up -d
   ```

   or

   ```
   docker-compose up -d
   ```

   (based on your Docker version) to start up the backend and frontend. Optionally omit the `-d` flag in order to not have the Docker containers running in the background. Use

   ```
   docker compose down
   ```

   to shut down MetaboSERV, and

   ```
   docker compose restart
   ```

   to restart it.

2. Navigate to the according URL in your browser to access the MetaboSERV frontend. By default, this is `http://localhost:9202`. For production, i.e., to make your MetaboSERV instance accessible on the internet, you should use HTTPS and a reverse proxy.

Logging in and out Click on the button on the top right to log in (or log out, if you are already logged in). You can either use a MetaboSERV account, which you can register by clicking the link on the bottom of the login menu, or an authorization token that has been passed to you by a third party.

If verification is turned on in the configuration options, you will need to verify your account before you unlock most permissions. A verification mail will be sent to you in this case, which will contain a code you need to enter once in the “Account” section after registering your account.

Both variants of authentication (user accounts and authorization tokens) will allow you to browse, download and in some cases edit a set of studies which were uploaded to MetaboSERV. If you use a MetaboSERV account, you may upload new studies, as well.

You can change your password in the “Account” section as well. If you have ever forgotten your password, you can use the according option in the login interface to receive a new temporary password through your e-mail address.

Creating/deleting/editing studies Navigate to the “Studies” tab and click the plus. Fill in the mandatory metadata values on the left. On the right side, you can add annotated files, which must adhere to the format specifications stated below. On the bottom right, you can also manually add some metadata entries.

You can tick the *transpose* option in order to swap rows and columns (refer to the format specifications).

You can edit studies at any time by clicking on a study in the “Studies” tab and clicking on the “Edit” button. You can also delete studies here if you wish.

Uploading/downloading experimental data In the “Studies” tab, click on a study and click on “Manage experimental data”. To upload files, you can either click on the dropbox on the left to browse your machine for according files, or drag files into the box using drag-and-drop. Uploaded files appear on the right, where you can download or delete them (given the necessary permissions). Note that the files you used to create the study (such as the concentration or phenotype data files) are automatically uploaded here.
Uploaded files can be used to alter the concentration and phenotype data or the study metadata at any time. Note that concentration data and phenotype data are always completely replaced if you use these options, while metadata you upload through a file is merged with existing metadata (with metadata from the file receiving priority in case of duplicate keys).

Managing study permissions Click on an existing study in the “Studies” tab. If you have the necessary permissions, you will be able to add new contributors, remove contributors (top left) and manage authorization tokens (top right) for the study. Otherwise, you will only be able to browse the contained phenotypes and metadata.

Queries You can query your studies by navigating to the “Query” tab. There are four distinct sections here, three of which open up after you selected at least one study in the **first section** (“Study Selection”). Any study you select here (indicated by being listed on the right) is included in your query.

In the **second section**, you can optionally limit the amount of metabolites to consider in your query. By default, all metabolites are used. On the very top of the window, you can choose to only retrieve a selected subset of metabolites, or to retrieve all metabolites but the selected subset. Select metabolites by clicking on them in the list on the left.

In the **third section**, you can optionally further limit which entries to consider by imposing filter rules on metabolites. There are generally three different filter rules. The first will cause your query to only include entries where the concentration level of the specified metabolite falls inside the range you provide. The second filter type does the same, but only considers entries where the concentration level falls outside of the provided range. The last only serves to visualize a particular range and will not change the query set.

If reference concentrations are contained in the MetaboSERV instance, you can also select them here wherever possible instead of providing a numerical range by yourself.

In the **fourth section**, you can choose which phenotypes should be included in your query. You can also limit the query set by the levels of each nominal phenotype, such as only considering healthy patients.

Creating quality control plots and heatmaps, visualizing Bruker NMR spectra MetaboSERV facilitates easy creation of quality control plots and Pearson correlation heatmaps for your uploaded concentration data. Navigate to “Browse” and select a study you want to create a quality control plot. Then select your desired plot, choose whether you want to create a plot from a `<LOD` or `>LOD` perspective (for QC plots) and click on Submit. Bruker NMR spectra can be visualized here, as well. Simply select a study, then select the according archive from the dropdown list and click on “Execute”. Note that the archive has to be uploaded using the “Manage experimental metadata” functionality of the study beforehand.

Mapping metabolites to their HMDB identifiers Navigate to the MetaboSERV landing page. The bottom line of the first section (right above “Citation”) has a link that will take you to the mapping utility. Here, you can either map a list of metabolites to their HMDB equivalents or provide a file that follows the concentration data format specifications outlined below. Click on the “Map” button. For each metabolite (either taken from your provided list or provided file), the MetaboSERV database will be searched (employing a “fuzzy matching” approach, i.e. metabolite names do not have to match exactly to be considered) for a fitting HMDB identifier.

All matches will have to be acknowledged and accepted by you, which you can do by clicking on the green tick button. Exact matches are accepted automatically. If you are dissatisfied with a particular mapping, you can browse through different matches for each metabolite by clicking on the “`<`” and “`>`” buttons. We use The Chemical Translation Service (CTS) located at `https://cts.fiehnlab.ucdavis.edu/`, provided by the Fiehn Lab at UC Davis West Coast Metabolomics Center [27], to try and resolve metabolites that were not matched at all in the MetaboSERV database. In case no match can be found using the MetaboSERV database or the CTS, which is indicated by the word *Failed*, you can still manually search for and provide a correct identifier by clicking on the “`+`” button. Note that this feature mostly exists for instances where HMDB identifiers are enforced - you can also simply accept failed matches or remove matches where you are unsatisfied with any of the suggestions (by clicking on the red “`X`”), which will result in the original metabolite name remaining and not being replaced by a HMDB identifier. The resulting metabolite mapping can be downloaded in JSON or CSV format. If you provided a concentration data file, you can also download the adapted version with all metabolites being replaced with their HMDB accession numbers (as long as you accepted the mappings).

3.6.3 Format specifications

For any data you upload, a particular format has to be adhered to in order for MetaboSERV to parse your files. The following sections describe the allowed formats in detail for each particular type of file, of which there are:

- Concentration data files, containing concentration levels of particular sources (e.g. patients) in your study
- Metadata files, containing general metadata for your study
- Phenotype files, containing nominal phenotype data for particular sources (e.g. patients) in your study
- Raw experimental data. This should be compressed/archived to save storage space, but otherwise no restrictions are placed on it.

Templates can be downloaded from the public MetaboSERV instance’s landing page.

Concentration data files Allowed file extensions are: CSV, TSV, XLS, XLSX. Allowed separators are tabs (`\t`) and semicolons (`;`). Even in CSV files, commas are currently not allowed in order not to interfere with the parsing of numerical data. The standard layout for concentration data files is to have one column per metabolite, and one column containing according patient IDs. Each source, e.g. a patient, is then reflected in one row of the file.

Please provide a header row. The column containing patient IDs should contain a header called `id` or `source_id`. All other columns are considered as metabolites, and whatever you pass in the header row is considered the metabolite name. In order to make the most out of MetaboSERV's querying features, such as combining different studies containing similar metabolites in a single query, it is optimal to provide HMDB accession numbers instead of metabolite names as those are guaranteed to be matched. However, any metabolite names are accepted unless HMDB identifiers are enforced in the relevant MetaboSERV instance.
In addition to rows for each source, you may also pass a special row that indicates the unit used for each metabolite. Insert `unit` in the `source_id` column of the file for this row, and then provide the according unit for each metabolite in the respective columns. MetaboSERV places no restrictions on unit identifiers.

Refer to this exemplary table or to the provided exemplary files for clarification:

source_id	metabolite 1	metabolite 2	...
unit	uL	mmol/L	...
patient_1	5471	<LOD	...
patient_2	6901	279	...
...	...	...	...

Each cell may contain either nothing, the special string `<LOD` (both treated as NAs) or a numerical value indicating the concentration value of the according source x for metabolite y.
You can also swap rows and columns, in which case you need to tick the *transpose* option for the concentration file when creating your study.

Metadata files Allowed file extensions: YML, JSON. You can provide a simple YML file to add some additional metadata for your study. This is particularly useful if you have large amounts of metadata. Otherwise, it is recommended to simply set it using the frontend GUI during study creation.
Each metadata entry is associated with a unique ID, a name and a value, passed in the format

```
id:
  name: Name
  value: Value
```

Example:

```
instrument:
  name: Spectrometer
  value: 600 MHz Bruker Avance III
another_md:
  name: Another attribute
  value: Another value
```

Or the JSON equivalent:

```
{
    "instrument": {
        "name": "Spectrometer",
        "value": "600 MHz Bruker Avance III"
    },
    "another_md": {
```

```
        "name": "Another attribute",
        "value": "Another value"
    }
}
```

You can pass an arbitrary amount of entries this way.

Phenotype data files Allowed file extensions: CSV, TSV, XLS, XLSX. Allowed separators are tabs (`\t`) and semicolons (`;`). Even in CSV files, commas are currently not allowed in order not to interfere with the parsing of numerical data. Generally, phenotype files are set up similarly to concentration data files. There are three types of possible columns:

- ID column (mandatory), which should be indicated by setting `id` or `source_id` in the header row. Add all source IDs, e.g. patient IDs, here.
- Phenotype columns (at least one is mandatory). Indicate phenotype columns by adding `PHENOTYPE_` before the phenotype description in the header row, e.g. `PHENOTYPE_Acute Kidney Injury`. Each cell in this column should contain the phenotype level that the source (e.g. patient) in each row is associated with.
- Metadata columns (optional). All columns which are not starting with `PHENOTYPE_` or are marked as the ID column are treated as metadata. You can add arbitrary values into these columns.

Refer to this exemplary table or the provided exemplary files for clarification:

source_id	PHENOTYPE_some disease	PHENOTYPE_something else	collection time	...
patient_1	healthy	level 1	17:37pm	...
patient_2	sick	level 1	11:17am	...
patient_3	healthy	level 2		...
...	...	...	...	...

Raw experimental data MetaboSERV sets no limitations about the data you choose to upload to a study, i.e. all types of files are allowed. It is up to the user to make sure no illegal content is uploaded. However, we recommend compressing larger files in order to reduce the storage space allocated to your study.

References

[1] R Core Team, *R: A Language and Environment for Statistical Computing*, R Foundation for Statistical Computing, Vienna, Austria, 2022. [Online]. Available: https://www.R-project.org/

[2] Flask-Cors: A Flask extension adding a decorator for CORS support. [Online]. Available: https://github.com/corydolphin/flask-cors

[3] M. S. Klein, "Affine Transformation of Negative Values for NMR Metabolomics Using the mrbin R Package," *Journal of Proteome Research*, vol. 20, no. 2, pp. 1397–1404, Feb. 2021. [Online]. Available: https://doi.org/10.1021/acs.jproteome.0c00684

[4] Flask-JWT-Extended: Extended JWT integration with Flask. [Online]. Available: https://github.com/vimalloc/flask-jwt-extended

[5] RestRserve: a Framework for Building HTTP API. [Online]. Available: https://restrserve.org/

[6] flask-swagger-ui: Swagger UI blueprint for Flask. [Online]. Available: https://github.com/sveint/flask-swagger-ui

[7] G. Bierman, M. Abadi, and M. Torgersen, "Understanding TypeScript," in *ECOOP 2014 – Object-Oriented Programming*, ser. Lecture Notes in Computer Science, R. Jones, Ed. Berlin, Heidelberg: Springer, 2014, pp. 257–281.

[8] C. R. Harris, K. J. Millman, S. J. van der Walt, R. Gommers, P. Virtanen, D. Cournapeau, E. Wieser, J. Taylor, S. Berg, N. J. Smith, R. Kern, M. Picus, S. Hoyer, M. H. van Kerkwijk, M. Brett, A. Haldane, J. F. del Río, M. Wiebe, P. Peterson, P. Gérard-Marchant, K. Sheppard, T. Reddy, W. Weckesser, H. Abbasi, C. Gohlke, and T. E. Oliphant, "Array programming with NumPy," *Nature*, vol. 585, no. 7825, pp. 357–362, Sep. 2020, number: 7825 Publisher: Nature Publishing Group. [Online]. Available: https://www.nature.com/articles/s41586-020-2649-2

[9] Vue.js - The Progressive JavaScript Framework | Vue.js. [Online]. Available: https://vuejs.org/

[10] J. D. Hunter, "Matplotlib: A 2D Graphics Environment," *Computing in Science & Engineering*, vol. 9, no. 3, pp. 90–95, May 2007, conference Name: Computing in Science & Engineering.

[11] Pinia | The intuitive store for Vue.js. [Online]. Available: https://pinia.vuejs.org

[12] M. L. Waskom, "seaborn: statistical data visualization," *Journal of Open Source Software*, vol. 6, no. 60, p. 3021, Apr. 2021. [Online]. Available: https://joss.theoj.org/papers/10.21105/joss.03021

[13] Angular Data Grid: Documentation. [Online]. Available: https://www.ag-grid.com/angular-data-grid/

[14] W. McKinney, "Data Structures for Statistical Computing in Python," Austin, Texas, 2010, pp. 56–61. [Online]. Available: https://conference.scipy.org/proceedings/scipy2010/mckinney.html

[15] (2023, Mar.) The official home of the python programming language. [Online]. Available: https://www.python.org/

[16] xmltodict: Makes working with XML feel like you are working with JSON. [Online]. Available: https://github.com/martinblech/xmltodict

[17] M. Grinberg, *Flask Web Development: Developing Web Applications with Python*, 1st ed. O'Reilly Media, Inc., 2014.

[18] Werkzeug: The comprehensive WSGI web application library. [Online]. Available: https://palletsprojects.com/p/werkzeug/

[19] xlrd: Library for developers to extract data from microsoft excel (tm) .xls spreadsheet files. [Online]. Available: http://www.python-excel.org/

[20] pytest documentation. [Online]. Available: https://docs.pytest.org/en/stable/

[21] email-validator: A robust email address syntax and deliverability validation library. [Online]. Available: https://github.com/JoshData/python-email-validator

[22] elasticsearch: Python client for Elasticsearch. [Online]. Available: https://github.com/elastic/elasticsearch-py

[23] A. Hupp. (2023, Apr.) python-magic. [Online]. Available: https://github.com/ahupp/python-magic

[24] (2020, Jun.) Python to MariaDB Connector. [Online]. Available: https://mariadb.com/resources/blog/how-to-connect-python-programs-to-mariadb/

[25] Dropzone.js. [Online]. Available: https://www.dropzone.dev/

[26] Gunicorn - wsgi server. [Online]. Available: https://docs.gunicorn.org/en/stable/

[27] G. Wohlgemuth, P. K. Haldiya, E. Willighagen, T. Kind, and O. Fiehn, "The chemical translation service—a web-based tool to improve standardization of metabolomic reports," *Bioinformatics*, vol. 26, no. 20, pp. 2647–2648, 2010. [Online]. Available: https://doi.org/10.1093/bioinformatics/btq476

[28] O. Hrydziuszko and M. R. Viant, “Missing values in mass spectrometry based metabolomics: an undervalued step in the data processing pipeline,” *Metabolomics*, vol. 8, pp. 161–174, 2012.

[29] N. F. Fino, O. M. Adingwupu, J. Coresh, T. Greene, B. Haaland, M. G. Shlipak, V. T. C. e Silva, R. Kalil, A. L. Mindikoglu, S. L. Furth *et al.*, “Evaluation of novel candidate filtration markers from a global metabolomic discovery for glomerular filtration rate estimation,” *Kidney international*, vol. 105, no. 3, pp. 582–592, 2024.
