# Supplementary material for: MetaboSERV—a platform for selecting, exchanging, and visualizing metabolomics data with controlled data access
Source: Gigascience. 2025 Aug 1;14:giaf075. doi: 10.1093/gigascience/giaf075 (PMC12315529; doi:10.1093/gigascience/giaf075)
Supplement: giaf075_GIGA-D-24-00275_Revision_1 [file giaf075_giga-d-24-00275_revision_1.pdf]

GigaScience

MetaboSERV - a platform for selecting, exchanging, and visualizing metabolomics data with controlled data access

--Manuscript Draft--

Manuscript Number:	GIGA-D-24-00275R1
Full Title:	MetaboSERV - a platform for selecting, exchanging, and visualizing metabolomics data with controlled data access
Article Type:	Technical Note
Funding Information:	Bundesministerium für Bildung und Forschung (01ZX1912A) — Ms. Helena U. Zacharias<br>Bundesministerium für Bildung und Forschung (01ZX1912C) — Mr. Michael Altenbuchinger<br>Bundesministerium für Bildung und Forschung (01ZX1912D) — Mr. Juergen Doenitz
Abstract:	Background<br>The growing number of metabolomics studies, based on high-dimensional data measured by hyphenated mass spectrometry (MS) and/or nuclear magnetic resonance (NMR) spectroscopy, has sparked the creation of several public metabolomics data repositories. Each repository emphasizes different aspects regarding data selection and representation, but most offer only limited options for privacy-preserving data sharing.<br>Results<br><br>We present MetaboSERV, an open-source, browser-based metabolomics platform dedicated to the selection, integration and sharing of quantitative metabolomics data and metadata with controlled data access. MetaboSERV aims to aid researchers in analyzing their results by facilitating means to browse, visualize and compare data across available data sets. It provides different access control functionalities, creating an environment in which data can be shared safely in a privacy-preserving manner to support collaborative and interdisciplinary research. Furthermore, it is designed to be extensible and adaptable to existing data management infrastructures through the creation of self-managed MetaboSERV instances, for which we provide the source code and a set of configurable Docker images.<br>Conclusions<br><br>The public MetaboSERV instance is available at https://metaboserv.ckdn.app, and the source code can be found at https://gitlab.gwdg.de/MedBioinf/metabolomics/metaboserv. The Research Resource Identifier (RRID) for MetaboSERV is SCR_025496.
Corresponding Author:	Tim Tucholski, M.Sc.<br>University Medical Center Göttingen: Universitatsmedizin Gottingen<br>Goettingen, GERMANY
Corresponding Author Secondary Information:	
Corresponding Author's Institution:	University Medical Center Göttingen: Universitatsmedizin Gottingen
Corresponding Author's Secondary Institution:	
First Author:	Tim Tucholski, M.Sc.
First Author Secondary Information:	
Order of Authors:	Tim Tucholski, M.Sc.
	Angela Maennel

Powered by Editorial Manager® and ProduXion Manager® from Aries Systems Corporation

	Yacoub Abelard Njipouombe Nsangou, M.Sc.
	Sven Schuchardt, Dr.
	Matthias Gruber
	Fabian Kellermeier, M.Sc.
	Katja Dettmer, Dr.
	Peter J. Oefner, Prof. Dr.
	Wolfram Gronwald, Prof. Dr.
	Michael Altenbuchinger, Prof. Dr.
	Juergen Doenitz, Dr.
	Helena U. Zacharias, Prof. Dr.
**Order of Authors Secondary Information:**	

Response to Reviewers:

Dear Dr. Zauner,

we are very thankful for the opportunity to submit a revised version of our manuscript "MetaboSERV - a platform for selecting, exchanging, and visualizing metabolomics data with controlled data access" (GIGA-D-24-00275) to GigaScience. In particular, we would like to thank you and the reviewers for the elaborate assessment of our manuscript and the fruitful comments, which we carefully address in a point-by-point reply below.

As you have particularly highlighted the reviewers' comments on data privacy, we would like to briefly elaborate on our efforts in this regards:

We fully agree with you and the reviewers that secure data handling is an important point which affects the project in different aspects, in particular (1) the operation of the public MetaboSERV instance, (2) the general architectural design and implementation of the MetaboSERV platform, and (3) the operation of local MetaboSERV instances.

The public instance of MetaboSERV, as described in the manuscript, is hosted in the computing center of the Hannover Medical School, Germany (MHH). MHH's regulations for access control to the server, security updates, backup and monitoring are implemented following the ISO 27001 and the standards of the German Federal Office for Information Security (Bundesamt für Sicherheit in der Informationstechnik, BSI), are focussed on handling sensitive clinical data, and therefore overfulfill the requirements for MetaboSERV. In particular, the public MetaboSERV server at MHH and the data stored there can only be accessed by authorized administrators. Server access is continuously logged and regularly inspected. The virtual machine is equipped with the latest security updates and regular backups are being taken every six hours.

Following GDPR regulations, the amount of personal data stored in MetaboSERV is minimized. As explicitly stated in the "Data privacy" statement on the public MetaboSERV instance, data uploaded to MetaboSERV should always be pseudonymized and contain no information to identify study participants. To create and manage MetaboSERV accounts, a minimum amount of personal information (username, e-mail address, and optionally institution) is requested and the necessity for their storage is explained in the "Data privacy" statement. Except for the e-mail address, no provided information is verified, as it mainly has the purpose to facilitate the permission structure of MetaboSERV. The user is free to choose a username with no link to their real-world identity. Upon account creation, the accounts are initially verified by an automated e-mail verification system and unauthorized access attempts are logged and reported back to the user (for more details on e-mail verification as well as access control, see our response to reviewer 1, comment 4 and reviewer 2, comment Q1). Passwords are only stored in an encrypted manner (hashed and salted). The risk of data loss is reduced by organisational measures and the effect of a data loss is minimized by encryption of the passwords.

For local installations and operations of MetaboSERV instances, the user remains fully

responsible for the security of the installation, e.g., that server access is controlled or data is not forwarded to third-parties. We have now created a video tutorial on the installation and set-up of local MetaboSERV instances, available at https://www.youtube.com/watch?v=vbqY2qJcgLk, where we also explicitly highlight steps during the installation set-up addressing vital data privacy aspects.

In the manuscript, we added a new subsection "Data privacy and security" at the end of the "Methods: Implementation" section, describing the GDPR-conform data handling in MetaboSERV. In the subsection "Results: MetaboSERV server environment", the secure hosting of the public MetaboSERV instance is described. Extended descriptions are provided in Supplementary File S2, "User Authentication and Password Storage".

You will find our complete point-by-point reply to the reviewer comments below.

There are no actual or potential conflicts of interest for any of the authors involved in this study. The results of this manuscript have not been published elsewhere, nor are they under consideration at any other journal. The manuscript has been read and approved by all authors. All the persons listed as co-authors have qualified for authorship and have approved the manuscript for submission.

We would like to thank you once again for your time reviewing this submission. Please contact us (Prof. Dr. Helena Zacharias: Zacharias.Helena@mh-hannover.de; Dr. Juergen Doenitz: juergen.doenitz@bioinf.med.uni-goettingen.de) in case any questions might arise.

We are looking forward to hearing from you.

Best regards,
Tim Tucholski, Helena Zacharias, and Juergen Doenitz

--- Reviewer reports ---

--- Reviewer 1 ---

The manuscript "MetaboSERV - platform for selecting, exchanging, and visualizing metabolomics data with controlled data access" presents an ambitious resource for metabolomics research which aims to provide a collaborative workspace tool designed for team-driven research efforts. The technical note emphasizes the privacy-preserving functionality of MetaboSERV and offers selective curation tools to evaluate multiple experimental outcomes simultaneously. The platform presented in this paper has the potential to be a valuable tool to research teams, but this reviewer was confused on what role the proposed platform serves in a broader scientific context. Below is a list of issues that should be addressed before this manuscript is suitable for publication:

Response by authors:

We are very grateful to the reviewer for his/her appreciation as well as detailed comments, and we have carefully tried to address the reviewer's concerns as outlined below.

-- Major Issues --

1. MetaboSERV is regularly compared to existing public metabolomics data repositories, but it was unclear if it is a public repository itself. The public instance of MetaboSERV only contains the example results outlined in this manuscript and none of the raw data from those experiments was available for download from the public instance. Additionally, this reviewer was unable to upload raw experimental data to the public instance on their own account. Is the public instance of MetaboSERV intended for end users or just a demo of a potential private instance? The manuscript is so focused on privacy and team collaboration, it is entirely unclear if this platform serves any functionality as a public data repository. As the introduction and discussion sections of this manuscript heavily compare MetaboSERV to existing public data repositories, the authors need to disambiguate this potential source of confusion.

Response by authors:

We are very thankful to the reviewer for pointing out this potential confusion. The public MetaboSERV instance is indeed not only a demo, but is intended as a fully functioning platform freely available to end-users. During our first submission, the data upload was disabled. We have now removed these restrictions and both data upload and download is now enabled for registered users. In order to disambiguate the intended role of the public MetaboSERV instance, we have carefully rephrased the complete manuscript and supplement: we now persistently differentiate between the "public MetaboSERV instance", hosted at MHH, and "local MetaboSERV instances", which are autonomously set-up at the end-user's institutions. Furthermore, we consistently use the term "MetaboSERV platform" (or simply "MetaboSERV") in case we describe general functionalities, which are valid for both the public and any local MetaboSERV instances. Furthermore, we completely revised the following section:

"Results: MetaboSERV server environment
The public MetaboSERV instance, available at https://metaboserv.ckdn.app, is hosted at the computing center of the Hannover Medical School, Germany (MHH). MHH's regulations for access control to the server, security updates, backup and monitoring are implemented following the ISO 27001 and the standards of the German Federal Office for Information Security (Bundesamt für Sicherheit in der Informationstechnik, BSI). In particular, the public MetaboSERV server at MHH and the data stored there can only be accessed by authorized administrators. Server access is continuously logged and regularly inspected. The virtual machine is equipped with the latest security updates and regular backups are being taken every six hours.

Local MetaboSERV instances can be set up by cloning our repositories at https://gitlab.gwdg.de/MedBioinf/metabolomics/metaboserv and creating, configuring, and running the respective Docker images. Detailed user guides are provided in Supplementary File S6: "Detailed installation and user guide for the set-up of local MetaboSERV instances" as well as at https://metaboserv.ckdn.app/guide and in our GitLab repositories. MetaboSERV is generally resource-friendly, all core components can be set up on a dual-core machine with 6 GB of random access memory (RAM) and 200 GB of hard disk space for MariaDB and Elasticsearch. Furthermore, a sufficient amount of hard disk space is necessary to store raw metabolomics data. Query speed is heavily dependent on the resources attributed to the underlying Elasticsearch and MariaDB instances. Therefore, it is recommended to set up MetaboSERV on a machine with at least four cores and 16 GB of RAM and to take advantage of Elasticsearch's sharding mechanism [44]. By default, MetaboSERV makes no assumptions about the Elasticsearch environment to avoid structural and capacity-related issues. These self-managed MetaboSERV instances run isolated from the public MetaboSERV instance and allow hosting and managing data on self-governed servers, removing any further data privacy concerns. They can also be altered and configured to fit different research environments and data formats, and assure a degree of system portability due to the nature of container virtualization. More information on user-specific configuration settings are provided in Supplementary File S6."

2. The authors make no mention of any existing privacy-focused workspaces for data sharing and collaboration. There are multiple commercial tools, such as Google Workspace, that enable sharing of experimental raw data and meta data. Many of these tools provide privacy-preserving functionality and are marketed to be in-compliance with protected health data security policy. This reviewer felt only comparing MetaboSERV to public metabolomics data repositories misrepresented available options for secure collaborative research. The authors should mention the state of existing commercial and open-source tools for secure data sharing and collaboration, and what their platform offers relative to these tools to better represent the impact of their platform.

Response by authors:

We are very thankful to the reviewer for pointing out a missing discussion on existing privacy-focused workspaces for data sharing and collaboration beyond metabolomics repositories. We have therefore extended our discussion on this topic and have added

the following paragraph to the main manuscript:

"Besides metabolomics-focussed data repositories, a large number of workspaces for data sharing and collaboration with options for privacy preservation have been released in the last decades, including commercial applications like Google Workspace and Nextcloud, as well as a multitude of freely available solutions including Figshare or Synapse/NF Data Portal [48]. Additionally, academic institutions worldwide start building up their own data sharing repositories, e.g., the Academic Cloud service for Lower Saxony, or RepoMed, the institutional repository of Hannover Medical School. However, none of these workspaces and solutions are designed to the specific needs of metabolomics data repositories, but rather provide "data-type agnostic" data lakes for the storage and retrieval of individual data sets [48]. In comparison to MetaboSERV, they do not provide smart search functions for metabolites across several, independent studies, no data analysis or visualization options, and, more importantly, do not support the set-up of self-administered, configurable instances, which are completely independent of the providers. The freely available software FAIRDOM-SEEK [49, 50] can be, similar to MetaboSERV, also deployed locally, however, it is designed particularly for data spanning multiple omics types or interconnecting datasets and systems biology models [49], and not for metabolomics data. Thus, uploaded data sets cannot be systematically queried or analysed with respect to individual metabolites and/or across studies."

-- Moderate Issues --

3. This reviewer observed some issues with metabolite metadata interactions when using MetaboSERV. The authors mention their tool will search for known synonyms of metabolites to match measures between studies that use different identifiers for the same molecule. However, this reviewer noted the metabolites in the 'AKI Study' and 'Biocrates Test' experiments could not be selectively queried together due to different synonym inputs. For example, 'L-Isoleucine' in the AKI Study did not match the 'Ile' metabolite abbreviation in the Biocrates Test experiment. This easily encountered conflict is highly concerning and suggests conflicting names will severely limit the cross-experiment selection functionality of MetaboSERV. Could the authors enforce uploads using chemical identifiers, such as HMDB, CAS, InChiKey, to enhance integration of multiple independent data sources?

Response by authors:

We thank the reviewer for his/her suggestion to integrate common chemical identifiers into MetaboSERV. We have decided not to enforce the use of chemical identifiers for our public MetaboSERV instance as not to impede the upload of metabolomics experiments containing unknown/unidentified metabolites. Nevertheless, we have now implemented the optional feature of mapping metabolite names to HMDB identifiers directly on the MetaboSERV website prior to data upload, which helps resolving metabolite synonyms. For this mapping process, we use data provided by the HMDB as well as The Chemical Translation Service (CTS) by G. Wohlgemuth et al. (http://cts.fiehnlab.ucdavis.edu). An enforcement of HMDB identifiers can optionally be used for self-managed MetaboSERV instances and can be toggled on or off at any point in time without retroactively affecting already uploaded studies. Enforcing HMDB identifiers will require users to either provide HMDB identifiers instead of metabolite names, or optionally have them mapped to the respective HMDB identifiers during the study creation process. We have thus updated section 3.3.1 in Supplementary File S3: MetaboSERV file specifications:

"The public MetaboSERV platform does not enforce the use of chemical identifiers for metabolite names as not to impede the upload of metabolomics experiments containing unknown/unidentified metabolites. However, to further ease seamless matching of metabolite names across different studies, MetaboSERV offers the optional, semi-automatic mapping of metabolite names to HMDB identifiers directly on the public MetaboSERV instance website prior to data upload. For this mapping process, we use data provided by the HMDB as well as The Chemical Translation Service (CTS) by G. Wohlgemuth et al. [27]. An enforcement of HMDB identifiers can optionally be used for self-managed, local MetaboSERV instances and can be toggled on or off at any point in time without retroactively affecting already uploaded studies.

Enforcing HMDB identifiers will require users to either provide HMDB identifiers instead of metabolite names, or optionally have them mapped to the respective HMDB identifiers during the study creation process."

Please note that, however, if metabolites are measured in different units across studies, we have now implemented a functionality that stops the user from creating multi-study-plots to avoid confusion and prevent the creation of misleading plots.

4. The manuscript mentions the cyber security efforts for ensuring the privacy of user created accounts and passwords. This reviewer observed that newly registered accounts did not require email validation, thus it would be very easy to impersonate any researcher by creating an account using a known email address.

Response by authors:

We are very thankful to the reviewer for pointing this out. To address this important comment, we have now introduced an e-mail verification system: Upon account creation, an e-mail containing a randomly generated, unique verification code is sent to the e-mail address specified by the new user. This code - which is valid for 24 hours - has to be entered once per account to verify it. Unverified accounts are treated as accounts with no special permissions and can only view or browse public studies. The e-mails are sent using SMTP by a function e-mail account, which has to be provided by administrators of self-managed MetaboSERV instances, should they choose to use this feature. While this e-mail verification step can be toggled off for self-managed MetaboSERV instances (which will cause all accounts to be auto-verified), it is mandatory for the public MetaboSERV instance. We provide detailed information on this e-mail verification system in the main manuscript (section "Data access control and management"):

"Upon registration, an e-mail containing a verification code is sent to the e-mail address that was used to register the account. This unique code, which consists of ten random characters, must be entered once after logging in to unlock any permissions associated with the account, which includes viewing studies shared with the account or uploading studies. The user accounts are associated with access rights for specific studies. For each uploaded data set, two different levels of access rights can be granted to other user accounts - either "read-only permissions" or "full data editing and management authority" - by the original uploader or users with "full data editing and management authority" accounts. The account responsible for the initial creation of the study possesses "full data editing and management authority" at any time and can never be removed as a contributor by any other user."

as well as in Supplementary File S2: User Authentication and Password Storage:

"In order to fully activate an account, the MetaboSERV platform uses an e-mail verification system, which can optionally be disabled for self-managed MetaboSERV instances. Upon registration, an e-mail containing a verification code is sent to the e-mail address that was used to register the account. This unique code, which consists of ten random characters, must be entered once after logging in to unlock any permissions associated with the account, which includes viewing studies shared with the account or uploading studies. Each code is valid for 24 hours or until a new code is generated. Failed login attempts are tracked and displayed to the user when they successfully log in. After a default number of five failed attempts, an e-mail reporting potentially suspicious activities is sent to the e-mail address associated with the account. After a default number of ten failed attempts, the account is temporarily locked for ten minutes. The number of attempts for both of those events can be configured for self-managed instances. A password recovery service allows the recovery of lost passwords associated with an account by employing the associated e-mail address. The e-mails are sent using SMTP by a function e-mail account, which has to be provided by administrators of self-managed MetaboSERV instances, should they choose to use this feature."

-- Minor Issues --

5. It would be very helpful to provide example upload templates for the 'Concentration

	data' and 'Phenotype data' study sections on MetaboSERV to ease the new user experience of this platform.<br><br>Response by authors:<br><br>We thank the reviewer for this thoughtful comment and now provide templates for the 'Concentration data', 'Phenotype data', as well as 'metadata'-YAML-files for download on the public MetaboSERV landing page. Additionally, we provide complete example data files for the three use cases on the MetaboSERV landing page. We now point out this information to the reader in Supplementary File S3: MetaboSERV file specifications:<br><br>"Templates for 'Concentration data', 'Phenotype data', as well as 'metadata'-YAML-files are provided for download on the public MetaboSERV landing page at https://metaboserv.ckdn.app/. Furthermore, complete example data files for the three use cases are provided on the same webpage."<br><br>6. The liquid chromatography platform model is missing from the description of the Biocrates MxP Quant 500 kit experiment in the methods section.<br><br>Response by authors:<br><br>We thank the reviewer for pointing out this missing information, which we now added to the following section in the main manuscript:<br><br>"Data was acquired on an AB Sciex 6500+ triple quadrupole mass spectrometer (AB Sciex Germany GmbH, Darmstadt, Germany) coupled to an ExionLC 30AD (AB Sciex Germany GmbH, Darmstadt, Germany) ultra-high performance liquid chromatography (UHPLC) system employing the MxP Quant 500 kit (Biocrates life sciences, Innsbruck, Austria)."<br><br>7. It appears that studies cannot be edited after creation when using the public instance. Is this the intended functionality or a bug? The manuscript alludes to that multiple researchers could upload the raw data, meta data, and phenotypes, but it is unclear how this could be accomplished if studies are not editable after creation.<br><br>Response by authors:<br><br>We apologize that during our first submission, the studies were not yet editable. We have now reactivated this functionality and all studies are editable by the study owners and users with the respective "full data editing and management authority" permissions.<br><br>8. How are unique study IDs enforced? Would private studies require unique study IDs from other private studies? How would users know what IDs are unavailable for use?<br><br>Response by authors:<br><br>We are very thankful to the reviewer for his/her inquiry about unique study IDs. We have now included the following paragraph in the Supplementary File S1, section 3.1.1:<br><br>"Each study object - such as the AKI study - is associated with a particular set of experiments and relevant metadata attributes such as the study authors, one or more biospecimens, the publication date or relevant filenames, as well as a unique study ID. The user provides a unique study ID, which is automatically compared to all existing study identifiers of the used MetaboSERV instance which are stored in both the MariaDB and Elasticsearch database. In the case a new study ID is already stored in the databases, a conflict message is shown and the user is asked to provide a new study ID."<br><br>9. When a user's study uses multiple methods, there is nowhere to specify which multiple methods where used. For example, a typical Biocrates MxP Quant 500 experiment includes an LC and flow injection component, but the example data only

Powered by Editorial Manager® and ProduXion Manager® from Aries Systems Corporation

notes the LC method. This issue is also observed in the Biospecimen category as well.

Response by authors:

We are very thankful to the reviewer for pointing this out. We now revised the implementation of both the "Analytical Method" and "Biospecimen" selection tabs on the "Create a new study" site to allow the selection of multiple options. On the corresponding "Data & Metadata" project site, these multiple methods and/or biospecimen types are then listed individually. These edits affect Figure 3A in the main manuscript, which has been updated accordingly.

Furthermore, we would like to point out that a data set comprising metabolites measured, for example, by different hyphenated MS modalities can be separated into multiple studies, each comprising metabolites measured by one specific modality. This allows the detailed description of the different methodological metadata associated with each modality. We describe this scenario in the newly added use case 3 in Supplementary File S5.

10. Does MetaboSERV also record methodological meta data, such as chromatography, MS, or NMR parameters? These are critical components to existing public metabolomics data repositories mentioned throughout this manuscript but are undiscussed in the context of this manuscript.

Response by authors:

MetaboSERV allows the recording of methodological metadata without any format and/or content restrictions. These methodological metadata can be provided by JSON/YAML metadata files and the deposited metadata is displayed on the corresponding "Data & Metadata" project site. To facilitate a low-threshold user experience of MetaboSERV, we have implemented a very flexible metadata upload by deliberately not enforcing mandatory metadata specifications. To illustrate this flexibility, we now specifically provide metadata of various resolution for the different use cases on the MetaboSERV public instance. Example JSON/YAML metadata files of all use cases as well as template files are further provided for download both on the public MetaboSERV landing page as well as the MetaboSERV GitLab repository (see also our response to comment 5 of reviewer 1). We now contrast MetaboSERV's flexibility with respect to methodological metadata in comparison to existing metabolomics data repositories in the discussion:

"Furthermore, MetaboSERV allows the recording of methodological metadata without any format and/or content restrictions. To facilitate a low-threshold user experience of MetaboSERV, we implemented a very flexible metadata upload by deliberately not enforcing mandatory metadata specifications. In contrast, established metabolomics data repositories such as MetaboLights and the Metabolomics Workbench face the users with rather strict mandatory metadata as well as experimental data upload requirements."

--- Reviewer 2 ---

The manuscript presents MetaboSERV, an open-source platform for metabolomics data selection, integration, and controlled access sharing. The manuscript is well-structured and clearly explains the functionalities and use cases of MetaboSERV. However, a few areas where additional detail and clarification could enhance the manuscript's clarity and impact.

Response by authors:

We thank the reviewer for his/her positive feedback and appreciation and have carefully tried to address his/her concerns as outlined below.

Q1: The authors highlight MetaboSERV's potential in maintaining data privacy in compliance with GDPR and similar regulations. Can MetaboSERV detect and log unauthorized access attempts, and if so, how are these managed? A deeper discussion on MetaboSERV's robustness in data security would better inform readers

of its suitability for handling sensitive biomedical data.

Response by authors:

We thank the reviewer for his/her suggestion to log unauthorized access attempts, which we have now implemented. The number of unauthorized attempts, which occurred since their last successful login, is now displayed to the user whenever they sign in. Also, an e-mail is sent to the e-mail address linked with the MetaboSERV account after a configurable number of tracked unauthorized access attempts, and the account is locked (i.e. unable for login) for ten minutes after another configurable number of tracked unauthorized access attempts. On the public instance, these are set to five and ten attempts, respectively. Moreover, we have implemented a password recovery service which allows the recovery of lost passwords associated with an account by employing the associated e-mail address.

We have included this information in the main manuscript, section "Data access control and management":

"Unauthorized access attempts are continuously logged in the Elasticsearch database. The number of unauthorized access attempts, which occurred since the last authorized log-in, is reported to the user upon every log-in. In the public MetaboSERV instance, an alert e-mail is sent to the user's e-mail address linked to the account in case the number of unauthorized access attempts exceeds five attempts, and the account is locked for ten minutes after a total of ten tracked unauthorized access attempts. On a successful log-in attempt, this counter is reset to zero. More information and customization details for local MetaboSERV instances are provided in Supplementary File S2."

and provide detailed information in Supplementary File S2: User Authentication and Password Storage:

"In order to fully activate an account, the MetaboSERV platform uses an e-mail verification system, which can optionally be disabled for self-managed MetaboSERV instances. Upon registration, an e-mail containing a verification code is sent to the e-mail address that was used to register the account. This unique code, which consists of ten random characters, must be entered once after logging in to unlock any permissions associated with the account, which includes viewing studies shared with the account or uploading studies. Each code is valid for 24 hours or until a new code is generated. Failed login attempts are tracked and displayed to the user when they successfully log in. After a default number of five failed attempts, an e-mail reporting potentially suspicious activities is sent to the e-mail address associated with the account. After a default number of ten failed attempts, the account is temporarily locked for ten minutes. The number of attempts for both of those events can be configured for self-managed instances. A password recovery service allows the recovery of lost passwords associated with an account by employing the associated e-mail address. The e-mails are sent using SMTP by a function e-mail account, which has to be provided by administrators of self-managed MetaboSERV instances, should they choose to use this feature."

Furthermore, we now elaborately discuss the server environment structure and organizational security measures of the public MetaboSERV instance hosted at MHH in the section "MetaboSERV server environment":

"The public MetaboSERV instance, available at https://metaboserv.ckdn.app, is hosted at the computing center of the Hannover Medical School, Germany (MHH). MHH's regulations for access control to the server, security updates, backup and monitoring are implemented following the ISO 27001 and the standards of the German Federal Office for Information Security (Bundesamt für Sicherheit in der Informationstechnik, BSI). In particular, the public MetaboSERV server at MHH and the data stored there can only be accessed by authorized administrators. Server access is continuously logged and regularly inspected. The virtual machine is equipped with the latest security updates and regular backups are being taken every six hours."

Q2: How does MetaboSERV scale with larger datasets, such as those often

encountered in multi-site studies or large biobanks? The examples provided as test data are relatively limited in size. Clinical metabolomics datasets can reach thousands of samples, so this aspect of scalability warrants further clarification.

Response by authors:

To demonstrate the scalability of MetaboSERV, we have added a third use case in Supplementary Section S5: "Use case 3: Large-scale, multi-modal, untargeted mass spectrometry data", employing a publicly available dataset from a study provided by Lesley A. Inker at Tufts Medical Center, University of Minnesota, including 1,002 samples x 1,228 metabolites. The dataset was split into four different MetaboSERV studies - one for each of the four different LC/MS modalities used for data collection - to comply with MetaboSERV's limit of 1,016 metabolites per uploaded study. By conducting this data subsetting, we also intend to show a possible representation of multiple LC/MS methods as suggested by comment 9 of reviewer 1. All four subsets contain data on the same 1,002 samples. The four LC/MS modalities and resulting dataset dimensionalities are: "LC/MS Neg" (692 metabolites), "LC/MS Polar" (79 metabolites), "LC/MS Pos Late" (173 metabolites), and "LC/MS Pos Early" (284 metabolites). More details on the different LC/MS modalities are provided in the main manuscript as well as in the Supplementary Material. Metabolite concentrations are given in arbitrary units ("a.u.").

To aid in displaying data from a study partitioned in this manner, we have also implemented an optional functionality that aggregates data based on the sample identifiers. As the same 1,002 samples are contained in all four studies, a joint query on all four studies can be performed in order to display the results as if they were retrieved from a single study. This feature can also be used to subset the data - for example, a joint query on only three out of the four studies could be performed in order to only retrieve data of three of the four LC/MS modalities.

We timed the upload and querying processes on a system that does not meet our minimum hardware recommendations - at least 2 CPU cores and 6GB of RAM - in order to determine MetaboSERV's performance on large datasets and to obtain a lower bound for the expected waiting time when dealing with large-scale metabolomics experiments using MetaboSERV. The system we used runs on 4GB of RAM and 2 cores.
Uploading times during study creation for each of the four studies ranged between 20 and 90 seconds, with the LC/MS Neg dataset taking the longest time due to the largest number of metabolites. The most performance-heavy query possible - the aforementioned joint query on all four study partitions, with the aggregation feature turned on - took 37 seconds to process on this setup.
Quality control plots for each of the partitions are generated in less than 30 seconds. Quality control plots based on the samples (as opposed to the metabolites), however, are generally generated much faster.

We describe use case 3 as follows in Supplementary Section S5:

"File S5: Use case 3: Large-scale, multi-modal, untargeted mass spectrometry data

In a third use case, we demonstrate the application of MetaboSERV on a large-scale, multi-modal, untargeted mass spectrometry data set, a typical scenario for major epidemiological studies:

An epidemiological research group would like to share a data set consisting of 1,228 unique metabolites semi-quantitatively measured by Metabolon in 1,002 human blood plasma specimens as well as the corresponding phenotype data on discovery/validation as well as quality control sample cohorts with two international collaboration partners. As these metabolites had been measured by four different LC/MS methods, they divide the data set into four subsets: (1) the LCMSneg subset, comprising 692 metabolites analyzed using basic negative ion optimized conditions, (2) the LCMSposearly subset, comprising 284 metabolites analyzed using acidic positive ion conditions, chromatographically optimized for more hydrophilic compounds, (3) the LCMSposlate subset with 173 metabolites analyzed using acidic positive ion conditions, chromatographically optimized for more hydrophobic compounds, and (4)

Powered by Editorial Manager® and ProduXion Manager® from Aries Systems Corporation

the LCMSpolar subset consisting of 79 metabolites analyzed via negative ionization following elution from a HILIC column [28]. For each data subset, they have created separate YAML files comprising individual experimental metadata. As the metabolites have only been semi-quantitatively measured, the epidemiological researchers specify the unit as "a.u.", i.e., arbitrary unit, in the concentration files. They upload each concentration and corresponding YAML file separately, which takes between 20 to 90 seconds depending on the amount of contained metabolites. They add their collaboration partners as "contributors" to the study.

The collaboration partners log into MetaboSERV and select all four studies, "ST002820 Metabolon LCMSneg", "ST002820 Metabolon LCMSposlate", "ST002820 Metabolon LCMSposearly", and "ST002820 Metabolon LCMSpolar" in the 'Query' browser. They scroll through the list of metabolites, alphabetically ordered across all four studies, and realize that the unidentified metabolite 'X - 02269' appears twice in the list, one time with the name 'X - 02269_1' and one time with the name 'X - 02269_2'. The same holds true for the metabolite '7-methylurate', which appears twice in the list. As the collaborators would like to understand why these two metabolites each appear twice in the list, they retrieve only these four metabolites in the 'Metabolite Selection' menu by selecting 'Choose subset', shown in Supplementary Figure S4. Furthermore, they choose the 'Filter Type' 'vis' for all four metabolites and for the phenotype information, only include the levels "Discovery", "Validation 1", "Validation 2", and "Blind QC". They hit the 'Submit' button without aggregation. In the 'Query Results' table, they order the results according to the provided phenotype variable, scroll through the individual Source IDs and realize that '7-methylurate_1' and 'X - 02269_1' are only available in the "LCMSneg" study, and '7-methylurate_2' and 'X - 02269_2' are only available in the "LCMSposearly" study (Supplementary Figure S5). As they are curious about the relationships between the respective metabolites, the collaboration partners first visualize the association between '7-methylurate_1' and '7-methylurate_2' in a scatterplot by selecting these two metabolites (Supplementary Figure S6A). In a second step, they perform the same analysis for the metabolites 'X - 02269_1' and 'X - 02269_2' (Supplementary Figure S6B). In both scatterplots, a high correlation between the respective metabolites can be detected, but it becomes also apparent, that the intensity scales seem to differ between the two LC/MS methods. The collaboration partners contact the epidemiology research group and point out their observations in order to discuss further data preprocessing steps."

Q3: The manuscript mentions the availability of Docker images for self-managed instances of MetaboSERV. Are these images easy to configure for users with limited technical backgrounds? In my lab, I use multiple software tools for processing raw data (e.g., MZmine, MS-DIAL, XCMS), structure elucidation (e.g., MS-FINDER), and in-house Python-based programs for data curation—all of which do not require extensive IT skills to set up. MetaboSERV seems more challenging to install, so a detailed manual would be beneficial.

Response by authors:

We provide a detailed installation and user guide in Supplementary File S6, section 3.6 "File S6: Detailed installation and user guide for the set-up of local MetaboSERV instances", which is also available at https://metaboserv.ckdn.app/guide on the public MetaboSERV instance as well as on the Gitlab repository at https://gitlab.gwdg.de/metaboserv2/backend/-/blob/main/README.md. Furthermore, we have created a video tutorial on the installation and management of local MetaboSERV instances, which is freely available at https://www.youtube.com/watch?v=vbqY2qJcgLk. We anticipate that these detailed manuals enable users without extensive IT skills to independently set-up and manage local MetaboSERV instances. For further support, we have added contact details for technical support on the landing page of the public MetaboSERV instance.

Q4: Is technical support available for installation and setup, or are there resources like a detailed user guide? Addressing this would reassure researchers of varying technical skill levels about the feasibility of independently setting up MetaboSERV.

Response by authors:

	A detailed installation manual and user guide can be found on the "Guide"-tab of the MetaboSERV public instance, to which we provide a link on the corresponding landing page, as well as on the MetaboSERV GitLab page. Additionally, we have created a video tutorial on the installation and management of local MetaboSERV instances, which is freely available at https://www.youtube.com/watch?v=vbqY2qJcgLk. Moreover, we have now included the detailed user guide in the Supplement as Supplementary File S6: "Detailed installation and user guide for the set-up of local MetaboSERV instances", section 3.6.2 "Using MetaboSERV". Likewise, we have added contact details for technical support on the landing page of the MetaboSERV public instance.<br><br>Q5: The authors limit the application of MetaboSERV to projects where metabolite concentrations are reported in molar units. This could exclude a substantial portion of metabolomics studies starting with a discovery cohort typically acquired using untargeted LC-MS or GC-MS-based metabolomics, often reported as peak areas or intensities in arbitrary units. Broadening this scope may enhance the platform's usability across a wider range of studies.<br><br>Response by authors:<br><br>MetaboSERV supports completely arbitrary units, as it simply uses what is provided by the user. However, if metabolites are measured in different units across studies, we have implemented a functionality that stops the user from creating multi-study-plots to avoid confusion and prevent the creation of misleading plots. To point out the flexibility of MetaboSERV with respect to concentration units to the reader, we now explicitly mention this in Supplementary File S3: MetaboSERV file specifications, section 3.3.1:<br><br>"The unit of measurement for each metabolite can be indicated by adding an extra 'unit' column and providing the unit for each metabolite in that column. To avoid the creation of misleading plots, MetaboSERV, however, does not allow the creation of multi-study-plots featuring the same metabolite measured in different units. To facilitate such analyses, the user needs to match the respective concentration units by converting them appropriately prior to data upload to MetaboSERV."<br><br>Moreover, we have now added a third use case with untargeted LC-MS data provided in arbitrary units, as detailed in our response to comment Q2 of reviewer 2.<br><br>Q6: How does MetaboSERV handle uploading raw LC-MS or GC-MS data? Here, raw data refer to the files generated directly from mass spectrometers (e.g., Agilent .d, Thermo .raw, Sciex .wiff, .wiff2). While MetaboSERV supports the upload and visualization of NMR spectra, extending this support to MS data would be beneficial, as LC-MS is a cornerstone in metabolomics.<br><br>Response by authors:<br><br>MetaboSERV allows the upload of raw (hyphenated) MS data of any data format, as long as it is bundled into a zip-file. In contrast to NMR metabolomics, which, in most cases, employs a Bruker NMR spectrometer and therefore, most of the NMR raw data available is provided in the Bruker file format, MS utilizes a large variety of different instrument providers, which all use their own data formats (as also reflected by the list of MS file formats provided by the reviewer). Since freely available software for the visualization of raw MS data are typically limited to specific raw data file formats, we have not implemented visualization options for raw (hyphenated) MS data. Moreover, MetaboSERV is designed for interdisciplinary collaborations involving researchers with varying background knowledge on MS. Since the profound judgment of the data quality of any raw metabolomics experiment by visual inspection is, at least to our knowledge, only possible by wet-lab metabolomics experts, we believe that the use of data visualizations of raw MS data within MetaboSERV is rather limited to wet-lab metabolomics experts. The latter, however, are typically able to easily visualize the raw data in the corresponding MS vendor software. In case the wet-lab metabolomics experts want to share these visualizations, they still have the option to share any visualization files, generated outside of MetaboSERV, within the zip-file bundle of the raw experimental data uploaded to MetaboSERV. To stress that the visualization option in the MetaboSERV platform is restricted to raw NMR data in the Bruker format,

	we have modified Figure 2 in the main manuscript accordingly.<br><br>Q7: Using the https://metaboserv.ckdn.app/ website, I attempted to replicate Figure 4. However, no response was received after submitting, so I could not test the functionalities associated with Figures 5 and 6.<br><br>Response by authors:<br><br>We are very sorry about this inconvenience and have carefully rechecked all functionalities prior to resubmission.
Additional Information:	
**Question**	**Response**
Are you submitting this manuscript to a special series or article collection?	No
**Experimental design and statistics**<br><br>Full details of the experimental design and statistical methods used should be given in the Methods section, as detailed in our Minimum Standards Reporting Checklist. Information essential to interpreting the data presented should be made available in the figure legends.<br><br>Have you included all the information requested in your manuscript?	Yes
**Resources**<br><br>A description of all resources used, including antibodies, cell lines, animals and software tools, with enough information to allow them to be uniquely identified, should be included in the Methods section. Authors are strongly encouraged to cite Research Resource Identifiers (RRIDs) for antibodies, model organisms and tools, where possible.<br><br>Have you included the information requested as detailed in our Minimum Standards Reporting Checklist?	Yes
**Availability of data and materials**<br><br>All datasets and code on which the	Yes

conclusions of the paper rely must be either included in your submission or deposited in publicly available repositories (where available and ethically appropriate), referencing such data using a unique identifier in the references and in the "Availability of Data and Materials" section of your manuscript.<br><br>Have you have met the above requirement as detailed in our Minimum Standards Reporting Checklist?	

Powered by Editorial Manager® and ProduXion Manager® from Aries Systems Corporation

Click here to access/download;LaTeX - Main Document (TeX file);main.tex

```
This is pdfTeX, Version 3.141592653-2.6-1.40.26 (TeX Live 2024)
(preloaded format=pdflatex 2024.8.2)  18 MAR 2025 12:12
entering extended mode
 restricted \write18 enabled.
 %&-line parsing enabled.
**main.tex
(./main.tex
LaTeX2e <2024-06-01> patch level 2
L3 programming layer <2024-05-27>
(./oup-contemporary.cls
Document Class: oup-contemporary 2023/06/12, v1.2
(c:/texlive/2024/texmf-dist/tex/latex/base/article.cls
Document Class: article 2024/02/08 v1.4n Standard LaTeX document class
(c:/texlive/2024/texmf-dist/tex/latex/base/size10.clo
File: size10.clo 2024/02/08 v1.4n Standard LaTeX file (size option)
)
\c@part=\count194
\c@section=\count195
\c@subsection=\count196
\c@subsubsection=\count197
\c@paragraph=\count198
\c@subparagraph=\count199
\c@figure=\count266
\c@table=\count267
\abovecaptionskip=\skip49
\belowcaptionskip=\skip50
\bibindent=\dimen141
) (c:/texlive/2024/texmf-dist/tex/latex/base/inputenc.sty
Package: inputenc 2024/02/08 v1.3d Input encoding file
\inpenc@prehook=\toks17
\inpenc@posthook=\toks18
) (c:/texlive/2024/texmf-dist/tex/latex/base/fontenc.sty
Package: fontenc 2021/04/29 v2.0v Standard LaTeX package
) (c:/texlive/2024/texmf-dist/tex/generic/iftex/ifpdf.sty
Package: ifpdf 2019/10/25 v3.4 ifpdf legacy package. Use iftex instead.
(c:/texlive/2024/texmf-dist/tex/generic/iftex/iftex.sty
Package: iftex 2022/02/03 v1.0f TeX engine tests
)) (c:/texlive/2024/texmf-dist/tex/latex/microtype/microtype.sty
Package: microtype 2024/03/29 v3.1b Micro-typographical refinements (RS)
(c:/texlive/2024/texmf-dist/tex/latex/graphics/keyval.sty
Package: keyval 2022/05/29 v1.15 key=value parser (DPC)
\KV@toks@=\toks19
) (c:/texlive/2024/texmf-dist/tex/latex/etoolbox/etoolbox.sty
Package: etoolbox 2020/10/05 v2.5k e-TeX tools for LaTeX (JAW)
\etb@tempcnta=\count268
)
\MT@toks=\toks20
\MT@tempbox=\box52
\MT@count=\count269
LaTeX Info: Redefining \noprotrusionifhmode on input line 1061.
LaTeX Info: Redefining \leftprotrusion on input line 1062.
\MT@prot@toks=\toks21
LaTeX Info: Redefining \rightprotrusion on input line 1081.
LaTeX Info: Redefining \textls on input line 1392.
```

```
\MT@outer@kern=\dimen142
LaTeX Info: Redefining \textmicrotypecontext on input line 2013.
\MT@listname@count=\count270
(c:/texlive/2024/texmf-dist/tex/latex/microtype/microtype-pdftex.def
File: microtype-pdftex.def 2024/03/29 v3.1b Definitions specific to
pdftex (RS)

LaTeX Info: Redefining \lsstyle on input line 902.
LaTeX Info: Redefining \lslig on input line 902.
\MT@outer@space=\skip51
)
Package microtype Info: Loading configuration file microtype.cfg.
(c:/texlive/2024/texmf-dist/tex/latex/microtype/microtype.cfg
File: microtype.cfg 2024/03/29 v3.1b microtype main configuration file
(RS)
)) (c:/texlive/2024/texmf-dist/tex/latex/euler/euler.sty
Package: euler 1995/03/05 v2.5
Package: `euler' v2.5 <1995/03/05> (FJ and FMi)
LaTeX Font Info:    Redeclaring symbol font `letters' on input line 35.
LaTeX Font Info:    Encoding `OML' has changed to `U' for symbol font
(Font)              `letters' in the math version `normal' on input line
35.
LaTeX Font Info:    Overwriting symbol font `letters' in version `normal'
(Font)                  OML/cmm/m/it --> U/eur/m/n on input line 35.
LaTeX Font Info:    Encoding `OML' has changed to `U' for symbol font
(Font)              `letters' in the math version `bold' on input line
35.
LaTeX Font Info:    Overwriting symbol font `letters' in version `bold'
(Font)                  OML/cmm/b/it --> U/eur/m/n on input line 35.
LaTeX Font Info:    Overwriting symbol font `letters' in version `bold'
(Font)                  U/eur/m/n --> U/eur/b/n on input line 36.
LaTeX Font Info:    Redeclaring math symbol \Gamma on input line 47.
LaTeX Font Info:    Redeclaring math symbol \Delta on input line 48.
LaTeX Font Info:    Redeclaring math symbol \Theta on input line 49.
LaTeX Font Info:    Redeclaring math symbol \Lambda on input line 50.
LaTeX Font Info:    Redeclaring math symbol \Xi on input line 51.
LaTeX Font Info:    Redeclaring math symbol \Pi on input line 52.
LaTeX Font Info:    Redeclaring math symbol \Sigma on input line 53.
LaTeX Font Info:    Redeclaring math symbol \Upsilon on input line 54.
LaTeX Font Info:    Redeclaring math symbol \Phi on input line 55.
LaTeX Font Info:    Redeclaring math symbol \Psi on input line 56.
LaTeX Font Info:    Redeclaring math symbol \Omega on input line 57.
\symEulerFraktur=\mathgroup4
LaTeX Font Info:    Overwriting symbol font `EulerFraktur' in version
`bold'
(Font)                  U/euf/m/n --> U/euf/b/n on input line 63.
LaTeX Info: Redefining \oldstylenums on input line 85.
\symEulerScript=\mathgroup5
LaTeX Font Info:    Overwriting symbol font `EulerScript' in version
`bold'
(Font)                  U/eus/m/n --> U/eus/b/n on input line 93.
LaTeX Font Info:    Redeclaring math symbol \aleph on input line 97.
LaTeX Font Info:    Redeclaring math symbol \Re on input line 98.
LaTeX Font Info:    Redeclaring math symbol \Im on input line 99.
```

```
LaTeX Font Info:    Redeclaring math delimiter \vert on input line 101.
LaTeX Font Info:    Redeclaring math delimiter \backslash on input line
103.
LaTeX Font Info:    Redeclaring math symbol \neg on input line 106.
LaTeX Font Info:    Redeclaring math symbol \wedge on input line 108.
LaTeX Font Info:    Redeclaring math symbol \vee on input line 110.
LaTeX Font Info:    Redeclaring math symbol \setminus on input line 112.
LaTeX Font Info:    Redeclaring math symbol \sim on input line 113.
LaTeX Font Info:    Redeclaring math symbol \mid on input line 114.
LaTeX Font Info:    Redeclaring math delimiter \arrowvert on input line
116.
LaTeX Font Info:    Redeclaring math symbol \mathsection on input line
117.
\symEulerExtension=\mathgroup6
LaTeX Font Info:    Redeclaring math symbol \coprod on input line 125.
LaTeX Font Info:    Redeclaring math symbol \prod on input line 125.
LaTeX Font Info:    Redeclaring math symbol \sum on input line 125.
LaTeX Font Info:    Redeclaring math symbol \intop on input line 130.
LaTeX Font Info:    Redeclaring math symbol \ointop on input line 131.
LaTeX Font Info:    Redeclaring math symbol \braceld on input line 132.
LaTeX Font Info:    Redeclaring math symbol \bracerd on input line 133.
LaTeX Font Info:    Redeclaring math symbol \bracelu on input line 134.
LaTeX Font Info:    Redeclaring math symbol \braceru on input line 135.
LaTeX Font Info:    Redeclaring math symbol \infty on input line 136.
LaTeX Font Info:    Redeclaring math symbol \nearrow on input line 153.
LaTeX Font Info:    Redeclaring math symbol \searrow on input line 154.
LaTeX Font Info:    Redeclaring math symbol \nwarrow on input line 155.
LaTeX Font Info:    Redeclaring math symbol \swarrow on input line 156.
LaTeX Font Info:    Redeclaring math symbol \Leftrightarrow on input line
157.
LaTeX Font Info:    Redeclaring math symbol \Leftarrow on input line 158.
LaTeX Font Info:    Redeclaring math symbol \Rightarrow on input line
159.
LaTeX Font Info:    Redeclaring math symbol \leftrightarrow on input line
160.
LaTeX Font Info:    Redeclaring math symbol \leftarrow on input line 161.
LaTeX Font Info:    Redeclaring math symbol \rightarrow on input line
163.
LaTeX Font Info:    Redeclaring math delimiter \uparrow on input line
166.
LaTeX Font Info:    Redeclaring math delimiter \downarrow on input line
168.
LaTeX Font Info:    Redeclaring math delimiter \updownarrow on input line
170.
LaTeX Font Info:    Redeclaring math delimiter \Uparrow on input line
172.
LaTeX Font Info:    Redeclaring math delimiter \Downarrow on input line
174.
LaTeX Font Info:    Redeclaring math delimiter \Updownarrow on input line
176.
LaTeX Font Info:    Redeclaring math symbol \leftharpoonup on input line
177.
LaTeX Font Info:    Redeclaring math symbol \leftharpoondown on input
line 178.
```

```
LaTeX Font Info:    Redeclaring math symbol \rightharpoonup on input line
179.
LaTeX Font Info:    Redeclaring math symbol \rightharpoondown on input
line 180
.
LaTeX Font Info:    Redeclaring math delimiter \lbrace on input line 182.
LaTeX Font Info:    Redeclaring math delimiter \rbrace on input line 184.
\symcmmigroup=\mathgroup7
LaTeX Font Info:    Overwriting symbol font `cmmigroup' in version `bold'
(Font)                  OML/cmm/m/it --> OML/cmm/b/it on input line 200.
LaTeX Font Info:    Redeclaring math accent \vec on input line 201.
LaTeX Font Info:    Redeclaring math symbol \triangleleft on input line
202.
LaTeX Font Info:    Redeclaring math symbol \triangleright on input line
203.
LaTeX Font Info:    Redeclaring math symbol \star on input line 204.
LaTeX Font Info:    Redeclaring math symbol \lhook on input line 205.
LaTeX Font Info:    Redeclaring math symbol \rhook on input line 206.
LaTeX Font Info:    Redeclaring math symbol \flat on input line 207.
LaTeX Font Info:    Redeclaring math symbol \natural on input line 208.
LaTeX Font Info:    Redeclaring math symbol \sharp on input line 209.
LaTeX Font Info:    Redeclaring math symbol \smile on input line 210.
LaTeX Font Info:    Redeclaring math symbol \frown on input line 211.
LaTeX Font Info:    Redeclaring math accent \grave on input line 245.
LaTeX Font Info:    Redeclaring math accent \acute on input line 246.
LaTeX Font Info:    Redeclaring math accent \tilde on input line 247.
LaTeX Font Info:    Redeclaring math accent \ddot on input line 248.
LaTeX Font Info:    Redeclaring math accent \check on input line 249.
LaTeX Font Info:    Redeclaring math accent \breve on input line 250.
LaTeX Font Info:    Redeclaring math accent \bar on input line 251.
LaTeX Font Info:    Redeclaring math accent \dot on input line 252.
LaTeX Font Info:    Redeclaring math accent \hat on input line 254.
) (c:/texlive/2024/texmf-dist/tex/latex/merriweather/merriweather.sty
Package: merriweather 2022/09/20 (Bob Tennent) Supports
Merriweather(Sans) font
s for all LaTeX engines.
(c:/texlive/2024/texmf-dist/tex/generic/iftex/ifxetex.sty
Package: ifxetex 2019/10/25 v0.7 ifxetex legacy package. Use iftex
instead.
) (c:/texlive/2024/texmf-dist/tex/generic/iftex/ifluatex.sty
Package: ifluatex 2019/10/25 v1.5 ifluatex legacy package. Use iftex
instead.
) (c:/texlive/2024/texmf-dist/tex/latex/base/textcomp.sty
Package: textcomp 2024/04/24 v2.1b Standard LaTeX package
) (c:/texlive/2024/texmf-dist/tex/latex/xkeyval/xkeyval.sty
Package: xkeyval 2022/06/16 v2.9 package option processing (HA)
(c:/texlive/2024/texmf-dist/tex/generic/xkeyval/xkeyval.tex
(c:/texlive/2024/te
xmf-dist/tex/generic/xkeyval/xkvutils.tex
\XKV@toks=\toks22
\XKV@tempa@toks=\toks23
)
\XKV@depth=\count271
```

```
File: xkeyval.tex 2014/12/03 v2.7a key=value parser (HA)
)) (c:/texlive/2024/texmf-dist/tex/latex/base/fontenc.sty
Package: fontenc 2021/04/29 v2.0v Standard LaTeX package
) (c:/texlive/2024/texmf-dist/tex/latex/fontaxes/fontaxes.sty
Package: fontaxes 2020/07/21 v1.0e Font selection axes
LaTeX Info: Redefining \upshape on input line 29.
LaTeX Info: Redefining \itshape on input line 31.
LaTeX Info: Redefining \slshape on input line 33.
LaTeX Info: Redefining \swshape on input line 35.
LaTeX Info: Redefining \scshape on input line 37.
LaTeX Info: Redefining \sscshape on input line 39.
LaTeX Info: Redefining \ulcshape on input line 41.
LaTeX Info: Redefining \textsw on input line 47.
LaTeX Info: Redefining \textssc on input line 48.
LaTeX Info: Redefining \textulc on input line 49.
)) (c:/texlive/2024/texmf-dist/tex/latex/mathastext/mathastext.sty
Package: mathastext 2024/07/27 v1.4b Use the text font in math mode (JFB)

Package mathastext Info: Starting the math mode configuration.
\mst@exists@muskip=\muskip17
\mst@forall@muskip=\muskip18
\mst@prime@muskip=\muskip19
\mst@do@nonletters=\toks24
\mst@undo@nonletters=\toks25
\mst@do@easynonletters=\toks26
\mst@undo@easynonletters=\toks27
\symmtoperatorfont=\mathgroup8
\symmtletterfont=\mathgroup9
(   mathastext:   ) ! and ?
(   mathastext:   ) punctuation: , . : ; and \colon
LaTeX Info: Redefining \relbar on input line 1201.
LaTeX Info: Redefining \rightarrowfill on input line 1202.
LaTeX Info: Redefining \leftarrowfill on input line 1205.
(   mathastext:   ) + and =
LaTeX Info: Redefining \Relbar on input line 1298.
(   mathastext:   ) adding = ; and + to \nfss@catcodes
(   mathastext:   ) parentheses ( ) [ ] and slash /
(   mathastext:   ) alldelims: < > \backslash \setminus | \vert \mid \{
\}
LaTeX Font Info:    Redeclaring math symbol \setminus on input line 1364.
LaTeX Info: Redefining \models on input line 1383.
(   mathastext:   ) \# \mathdollar \% \&
(   mathastext:   ) \imath and \jmath
LaTeX Font Info:    Overwriting math alphabet `\Mathnormalbold' in
version `nor
mal'
(Font)                  T1/Merriwthr-OsF/b/it --> T1/Merriwthr-OsF/b/it
on inpu
t line 2863.
LaTeX Font Info:    Overwriting math alphabet `\Mathnormalbold' in
version `bol
d'
(Font)                  T1/Merriwthr-OsF/b/it --> T1/Merriwthr-OsF/b/it
on inpu
```

```
t line 2863.
LaTeX Font Info:    Overwriting symbol font `mtletterfont' in version
`normal'
(Font)                  T1/Merriwthr-OsF/m/it --> T1/Merriwthr-OsF/m/it
on inpu
t line 2863.
LaTeX Font Info:    Overwriting symbol font `mtletterfont' in version
`bold'
(Font)                  T1/Merriwthr-OsF/m/it --> T1/Merriwthr-OsF/b/it
on inpu
t line 2863.
LaTeX Font Info:    Overwriting symbol font `mtoperatorfont' in version
`normal
'
(Font)                  T1/Merriwthr-OsF/m/n --> T1/Merriwthr-OsF/m/n on
input
line 2863.
LaTeX Font Info:    Overwriting symbol font `mtoperatorfont' in version
`bold'
(Font)                  T1/Merriwthr-OsF/m/n --> T1/Merriwthr-OsF/b/n on
input
line 2863.
LaTeX Font Info:    Overwriting math alphabet `\Mathbf' in version
`normal'
(Font)                  T1/Merriwthr-OsF/b/n --> T1/Merriwthr-OsF/b/n on
input
line 2863.
LaTeX Font Info:    Overwriting math alphabet `\Mathbf' in version `bold'
(Font)                  T1/Merriwthr-OsF/b/n --> T1/Merriwthr-OsF/b/n on
input
line 2863.
LaTeX Font Info:    Overwriting math alphabet `\Mathit' in version
`normal'
(Font)                  T1/Merriwthr-OsF/m/it --> T1/Merriwthr-OsF/m/it
on inpu
t line 2863.
LaTeX Font Info:    Overwriting math alphabet `\Mathit' in version `bold'
(Font)                  T1/Merriwthr-OsF/m/it --> T1/Merriwthr-OsF/b/it
on inpu
t line 2863.
LaTeX Font Info:    Overwriting math alphabet `\Mathsf' in version
`normal'
(Font)                  T1/MerriwthrSans-OsF/m/n --> T1/MerriwthrSans-
OsF/m/n o
n input line 2863.
LaTeX Font Info:    Overwriting math alphabet `\Mathsf' in version `bold'
(Font)                  T1/MerriwthrSans-OsF/m/n --> T1/MerriwthrSans-
OsF/b/n o
n input line 2863.
LaTeX Font Info:    Overwriting math alphabet `\Mathtt' in version
`normal'
(Font)                  T1/lmtt/m/n --> T1/lmtt/m/n on input line 2863.
LaTeX Font Info:    Overwriting math alphabet `\Mathtt' in version `bold'
(Font)                  T1/lmtt/m/n --> T1/lmtt/b/n on input line 2863.
```

```
(   mathastext:   ) Latin letters in the `normal', resp. `bold',
(   mathastext:   ) math versions are now set up to use the fonts
(   mathastext:   ) T1/Merriwthr-OsF/m/it, resp. T1/Merriwthr-OsF/b/it.
(   mathastext:   ) Other characters (digits, ...) and \log-like names
will be
(   mathastext:   ) typeset with the n shape.
(   mathastext:   ) \hbar
(   mathastext:   ) minus as endash
(   mathastext:   ) The italic option is in effect.
(   mathastext:   ) \HUGE has been (re)-defined.
(   mathastext:   ) mathastext has declared larger sizes for subscripts.
(   mathastext:   ) To keep LaTeX defaults, use option
`defaultmathsizes'.

Package mathastext Info: Loading is complete.  You can now use
\Mathastext to
(mathastext)             modify the normal and bold math versions.  Use
it
(mathastext)             with optional argument or use \MTDeclareVersion
to
(mathastext)             declare additional math versions.
) (c:/texlive/2024/texmf-dist/tex/latex/relsize/relsize.sty
Package: relsize 2013/03/29 ver 4.1
) (c:/texlive/2024/texmf-dist/tex/latex/ragged2e/ragged2e.sty
Package: ragged2e 2023/06/22 v3.6 ragged2e Package
\CenteringLeftskip=\skip52
\RaggedLeftLeftskip=\skip53
\RaggedRightLeftskip=\skip54
\CenteringRightskip=\skip55
\RaggedLeftRightskip=\skip56
\RaggedRightRightskip=\skip57
\CenteringParfillskip=\skip58
\RaggedLeftParfillskip=\skip59
\RaggedRightParfillskip=\skip60
\JustifyingParfillskip=\skip61
\CenteringParindent=\skip62
\RaggedLeftParindent=\skip63
\RaggedRightParindent=\skip64
\JustifyingParindent=\skip65
) (c:/texlive/2024/texmf-dist/tex/latex/xcolor/xcolor.sty
Package: xcolor 2023/11/15 v3.01 LaTeX color extensions (UK)
(c:/texlive/2024/texmf-dist/tex/latex/graphics-cfg/color.cfg
File: color.cfg 2016/01/02 v1.6 sample color configuration
)
Package xcolor Info: Driver file: pdftex.def on input line 274.
(c:/texlive/2024/texmf-dist/tex/latex/graphics-def/pdftex.def
File: pdftex.def 2024/04/13 v1.2c Graphics/color driver for pdftex
) (c:/texlive/2024/texmf-dist/tex/latex/graphics/mathcolor.ltx)
Package xcolor Info: Model `cmy' substituted by `cmy0' on input line
1350.
Package xcolor Info: Model `hsb' substituted by `rgb' on input line 1354.
Package xcolor Info: Model `RGB' extended on input line 1366.
Package xcolor Info: Model `HTML' substituted by `rgb' on input line
1368.
```

```
Package xcolor Info: Model `Hsb' substituted by `hsb' on input line 1369.
Package xcolor Info: Model `tHsb' substituted by `hsb' on input line
1370.
Package xcolor Info: Model `HSB' substituted by `hsb' on input line 1371.
Package xcolor Info: Model `Gray' substituted by `gray' on input line
1372.
Package xcolor Info: Model `wave' substituted by `hsb' on input line
1373.
) (c:/texlive/2024/texmf-dist/tex/latex/colortbl/colortbl.sty
Package: colortbl 2024/07/06 v1.0i Color table columns (DPC)
(c:/texlive/2024/texmf-dist/tex/latex/tools/array.sty
Package: array 2024/06/14 v2.6d Tabular extension package (FMi)
\col@sep=\dimen143
\ar@mcellbox=\box53
\extrarowheight=\dimen144
\NC@list=\toks28
\extratabsurround=\skip66
\backup@length=\skip67
\ar@cellbox=\box54
)
\everycr=\toks29
\minrowclearance=\skip68
\rownum=\count272
) (c:/texlive/2024/texmf-dist/tex/latex/graphics/graphicx.sty
Package: graphicx 2021/09/16 v1.2d Enhanced LaTeX Graphics (DPC,SPQR)
(c:/texlive/2024/texmf-dist/tex/latex/graphics/graphics.sty
Package: graphics 2024/05/23 v1.4g Standard LaTeX Graphics (DPC,SPQR)
(c:/texlive/2024/texmf-dist/tex/latex/graphics/trig.sty
Package: trig 2023/12/02 v1.11 sin cos tan (DPC)
) (c:/texlive/2024/texmf-dist/tex/latex/graphics-cfg/graphics.cfg
File: graphics.cfg 2016/06/04 v1.11 sample graphics configuration
)
Package graphics Info: Driver file: pdftex.def on input line 106.
)
\Gin@req@height=\dimen145
\Gin@req@width=\dimen146
) (c:/texlive/2024/texmf-dist/tex/latex/xpatch/xpatch.sty
(c:/texlive/2024/texm
f-dist/tex/latex/l3kernel/expl3.sty
Package: expl3 2024-05-27 L3 programming layer (loader)
(c:/texlive/2024/texmf-dist/tex/latex/l3backend/l3backend-pdftex.def
File: l3backend-pdftex.def 2024-05-08 L3 backend support: PDF output
(pdfTeX)
\l__color_backend_stack_int=\count273
\l__pdf_internal_box=\box55
))
Package: xpatch 2020/03/25 v0.3a Extending etoolbox patching commands
(c:/texlive/2024/texmf-dist/tex/latex/l3packages/xparse/xparse.sty
Package: xparse 2024-05-08 L3 Experimental document command parser
)) (c:/texlive/2024/texmf-dist/tex/latex/environ/environ.sty
Package: environ 2014/05/04 v0.3 A new way to define environments
(c:/texlive/2024/texmf-dist/tex/latex/trimspaces/trimspaces.sty
Package: trimspaces 2009/09/17 v1.1 Trim spaces around a token list
)
```

```
\@envbody=\toks30
) (c:/texlive/2024/texmf-dist/tex/latex/lastpage/lastpage.sty
Package: lastpage 2024/07/07 v2.1c lastpage: 2.09 or 2e? (HMM)
(c:/texlive/2024/texmf-dist/tex/latex/lastpage/lastpage2e.sty
Package: lastpage2e 2024/07/07 v2.1c Decide which 2e lastpage version to
use (H
MM)
(c:/texlive/2024/texmf-dist/tex/latex/lastpage/lastpagemodern.sty
Package: lastpagemodern 2024-07-07 v2.1c Refers to last page's name (HMM;
JPG)
\c@lastpagecount=\count274
)
)) (c:/texlive/2024/texmf-dist/tex/latex/graphics/rotating.sty
Package: rotating 2016/08/11 v2.16d rotated objects in LaTeX
(c:/texlive/2024/texmf-dist/tex/latex/base/ifthen.sty
Package: ifthen 2024/03/16 v1.1e Standard LaTeX ifthen package (DPC)
)
\c@r@tfl@t=\count275
\rotFPtop=\skip69
\rotFPbot=\skip70
\rot@float@box=\box56
\rot@mess@toks=\toks31
) (c:/texlive/2024/texmf-dist/tex/latex/graphics/lscape.sty
Package: lscape 2020/05/28 v3.02 Landscape Pages (DPC)
) (c:/texlive/2024/texmf-dist/tex/latex/tools/afterpage.sty
Package: afterpage 2023/07/04 v1.08 After-Page Package (DPC)
\AP@output=\toks32
\AP@partial=\box57
\AP@footins=\box58
) (c:/texlive/2024/texmf-dist/tex/latex/textpos/textpos.sty
Package: textpos 2022/07/23 v1.10.1
Package textpos Info: choosing support for LaTeX3 on input line 60.
\TP@textbox=\box59
\TP@holdbox=\box60
\TPHorizModule=\dimen147
\TPVertModule=\dimen148
\TP@margin=\dimen149
\TP@absmargin=\dimen150
Grid set 16 x 16 = 37.34424pt x 52.81541pt
\TPboxrulesize=\dimen151
\TP@ox=\dimen152
\TP@oy=\dimen153
\TP@tbargs=\toks33
TextBlockOrigin set to 0pt x 0pt
) (c:/texlive/2024/texmf-dist/tex/latex/url/url.sty
\Urlmuskip=\muskip20
Package: url 2013/09/16  ver 3.4  Verb mode for urls, etc.
) (c:/texlive/2024/texmf-dist/tex/latex/newfloat/newfloat.sty
Package: newfloat 2023/10/01 v1.2 Defining new floating environments (AR)
Package newfloat Info: `rotating' package detected.
) (c:/texlive/2024/texmf-dist/tex/latex/mdframed/mdframed.sty
Package: mdframed 2013/07/01 1.9b: mdframed
(c:/texlive/2024/texmf-dist/tex/latex/kvoptions/kvoptions.sty
```

```
Package: kvoptions 2022-06-15 v3.15 Key value format for package options
(HO)
(c:/texlive/2024/texmf-dist/tex/generic/ltxcmds/ltxcmds.sty
Package: ltxcmds 2023-12-04 v1.26 LaTeX kernel commands for general use
(HO)
) (c:/texlive/2024/texmf-dist/tex/latex/kvsetkeys/kvsetkeys.sty
Package: kvsetkeys 2022-10-05 v1.19 Key value parser (HO)
)) (c:/texlive/2024/texmf-dist/tex/latex/zref/zref-abspage.sty
Package: zref-abspage 2023-09-14 v2.35 Module abspage for zref (HO)
(c:/texlive/2024/texmf-dist/tex/latex/zref/zref-base.sty
Package: zref-base 2023-09-14 v2.35 Module base for zref (HO)
(c:/texlive/2024/texmf-dist/tex/generic/infwarerr/infwarerr.sty
Package: infwarerr 2019/12/03 v1.5 Providing info/warning/error messages
(HO)
) (c:/texlive/2024/texmf-dist/tex/generic/kvdefinekeys/kvdefinekeys.sty
Package: kvdefinekeys 2019-12-19 v1.6 Define keys (HO)
) (c:/texlive/2024/texmf-dist/tex/generic/pdftexcmds/pdftexcmds.sty
Package: pdftexcmds 2020-06-27 v0.33 Utility functions of pdfTeX for
LuaTeX (HO
)
Package pdftexcmds Info: \pdf@primitive is available.
Package pdftexcmds Info: \pdf@ifprimitive is available.
Package pdftexcmds Info: \pdfdraftmode found.
) (c:/texlive/2024/texmf-dist/tex/generic/etexcmds/etexcmds.sty
Package: etexcmds 2019/12/15 v1.7 Avoid name clashes with e-TeX commands
(HO)
) (c:/texlive/2024/texmf-dist/tex/latex/auxhook/auxhook.sty
Package: auxhook 2019-12-17 v1.6 Hooks for auxiliary files (HO)
)
Package zref Info: New property list: main on input line 767.
Package zref Info: New property: default on input line 768.
Package zref Info: New property: page on input line 769.
)
\c@abspage=\count276
Package zref Info: New property: abspage on input line 67.
) (c:/texlive/2024/texmf-dist/tex/latex/needspace/needspace.sty
Package: needspace 2010/09/12 v1.3d reserve vertical space
)
\mdf@templength=\skip71
\c@mdf@globalstyle@cnt=\count277
\mdf@skipabove@length=\skip72
\mdf@skipbelow@length=\skip73
\mdf@leftmargin@length=\skip74
\mdf@rightmargin@length=\skip75
\mdf@innerleftmargin@length=\skip76
\mdf@innerrightmargin@length=\skip77
\mdf@innertopmargin@length=\skip78
\mdf@innerbottommargin@length=\skip79
\mdf@splittopskip@length=\skip80
\mdf@splitbottomskip@length=\skip81
\mdf@outermargin@length=\skip82
\mdf@innermargin@length=\skip83
\mdf@linewidth@length=\skip84
\mdf@innerlinewidth@length=\skip85
```

```
\mdf@middlelinewidth@length=\skip86
\mdf@outerlinewidth@length=\skip87
\mdf@roundcorner@length=\skip88
\mdf@footenotedistance@length=\skip89
\mdf@userdefinedwidth@length=\skip90
\mdf@needspace@length=\skip91
\mdf@frametitleaboveskip@length=\skip92
\mdf@frametitlebelowskip@length=\skip93
\mdf@frametitlerulewidth@length=\skip94
\mdf@frametitleleftmargin@length=\skip95
\mdf@frametitlerightmargin@length=\skip96
\mdf@shadowsize@length=\skip97
\mdf@extratopheight@length=\skip98
\mdf@subtitleabovelinewidth@length=\skip99
\mdf@subtitlebelowlinewidth@length=\skip100
\mdf@subtitleaboveskip@length=\skip101
\mdf@subtitlebelowskip@length=\skip102
\mdf@subtitleinneraboveskip@length=\skip103
\mdf@subtitleinnerbelowskip@length=\skip104
\mdf@subsubtitleabovelinewidth@length=\skip105
\mdf@subsubtitlebelowlinewidth@length=\skip106
\mdf@subsubtitleaboveskip@length=\skip107
\mdf@subsubtitlebelowskip@length=\skip108
\mdf@subsubtitleinneraboveskip@length=\skip109
\mdf@subsubtitleinnerbelowskip@length=\skip110
(c:/texlive/2024/texmf-dist/tex/latex/mdframed/md-frame-0.mdf
File: md-frame-0.mdf 2013/07/01\ 1.9b: md-frame-0
)
\mdf@frametitlebox=\box61
\mdf@footnotebox=\box62
\mdf@splitbox@one=\box63
\mdf@splitbox@two=\box64
\mdf@splitbox@save=\box65
\mdfsplitboxwidth=\skip111
\mdfsplitboxtotalwidth=\skip112
\mdfsplitboxheight=\skip113
\mdfsplitboxdepth=\skip114
\mdfsplitboxtotalheight=\skip115
\mdfframetitleboxwidth=\skip116
\mdfframetitleboxtotalwidth=\skip117
\mdfframetitleboxheight=\skip118
\mdfframetitleboxdepth=\skip119
\mdfframetitleboxtotalheight=\skip120
\mdffootnoteboxwidth=\skip121
\mdffootnoteboxtotalwidth=\skip122
\mdffootnoteboxheight=\skip123
\mdffootnoteboxdepth=\skip124
\mdffootnoteboxtotalheight=\skip125
\mdftotallinewidth=\skip126
\mdfboundingboxwidth=\skip127
\mdfboundingboxtotalwidth=\skip128
\mdfboundingboxheight=\skip129
\mdfboundingboxdepth=\skip130
\mdfboundingboxtotalheight=\skip131
```

```
\mdf@freevspace@length=\skip132
\mdf@horizontalwidthofbox@length=\skip133
\mdf@verticalmarginwhole@length=\skip134
\mdf@horizontalspaceofbox=\skip135
\mdfsubtitleheight=\skip136
\mdfsubsubtitleheight=\skip137
\c@mdfcountframes=\count278

****** mdframed patching \endmdf@trivlist

****** -- success******

\mdf@envdepth=\count279
\c@mdf@env@i=\count280
\c@mdf@env@ii=\count281
\c@mdf@zref@counter=\count282
Package zref Info: New property: mdf@pagevalue on input line 895.
) (c:/texlive/2024/texmf-dist/tex/latex/titlesec/titlesec.sty
Package: titlesec 2023/10/27 v2.16 Sectioning titles
\ttl@box=\box66
\beforetitleunit=\skip138
\aftertitleunit=\skip139
\ttl@plus=\dimen154
\ttl@minus=\dimen155
\ttl@toksa=\toks34
\titlewidth=\dimen156
\titlewidthlast=\dimen157
\titlewidthfirst=\dimen158
) (c:/texlive/2024/texmf-dist/tex/latex/koma-script/scrextend.sty
Package: scrextend 2023/07/07 v3.41 KOMA-Script package (extend other
classes w
ith features of KOMA-Script classes)
(c:/texlive/2024/texmf-dist/tex/latex/koma-script/scrkbase.sty
Package: scrkbase 2023/07/07 v3.41 KOMA-Script package (KOMA-Script-
dependent b
asics and keyval usage)
(c:/texlive/2024/texmf-dist/tex/latex/koma-script/scrbase.sty
Package: scrbase 2023/07/07 v3.41 KOMA-Script package (KOMA-Script-
independent
basics and keyval usage)
(c:/texlive/2024/texmf-dist/tex/latex/koma-script/scrlfile.sty
Package: scrlfile 2023/07/07 v3.41 KOMA-Script package (file load hooks)
(c:/texlive/2024/texmf-dist/tex/latex/koma-script/scrlfile-hook.sty
Package: scrlfile-hook 2023/07/07 v3.41 KOMA-Script package (using LaTeX
hooks)

(c:/texlive/2024/texmf-dist/tex/latex/koma-script/scrlogo.sty
Package: scrlogo 2023/07/07 v3.41 KOMA-Script package (logo)
)))
Applying: [2021/05/01] Usage of raw or classic option list on input line
252.
Already applied: [0000/00/00] Usage of raw or classic option list on
input line
 368.
```

```
))
Package scrextend Info: unexpected definition of `\@makefnmark'.
(scrextend)             Trying to patch it on input line 1762.
Package scrextend Info: patch seems to be successfull on input line 1762.
)

LaTeX Font Warning: Font shape `T1/cmr/m/n' in size <7.5> not available
(Font)              size <7> substituted on input line 69.

(c:/texlive/2024/texmf-dist/tex/latex/tools/calc.sty
Package: calc 2023/07/08 v4.3 Infix arithmetic (KKT,FJ)
\calc@Acount=\count283
\calc@Bcount=\count284
\calc@Adimen=\dimen159
\calc@Bdimen=\dimen160
\calc@Askip=\skip140
\calc@Bskip=\skip141
LaTeX Info: Redefining \setlength on input line 80.
LaTeX Info: Redefining \addtolength on input line 81.
\calc@Ccount=\count285
\calc@Cskip=\skip142
) (c:/texlive/2024/texmf-dist/tex/latex/geometry/geometry.sty
Package: geometry 2020/01/02 v5.9 Page Geometry
(c:/texlive/2024/texmf-dist/tex/generic/iftex/ifvtex.sty
Package: ifvtex 2019/10/25 v1.7 ifvtex legacy package. Use iftex instead.
)
\Gm@cnth=\count286
\Gm@cntv=\count287
\c@Gm@tempcnt=\count288
\Gm@bindingoffset=\dimen161
\Gm@wd@mp=\dimen162
\Gm@odd@mp=\dimen163
\Gm@even@mp=\dimen164
\Gm@layoutwidth=\dimen165
\Gm@layoutheight=\dimen166
\Gm@layouthoffset=\dimen167
\Gm@layoutvoffset=\dimen168
\Gm@dimlist=\toks35
) (c:/texlive/2024/texmf-dist/tex/latex/preprint/authblk.sty
Package: authblk 2001/02/27 1.3 (PWD)
\affilsep=\skip143
\@affilsep=\skip144
\c@Maxaffil=\count289
\c@authors=\count290
\c@affil=\count291
) (c:/texlive/2024/texmf-dist/tex/latex/footmisc/footmisc.sty
Package: footmisc 2023/07/05 v6.0f a miscellany of footnote facilities
\FN@temptoken=\toks36
\footnotemargin=\dimen169
\@outputbox@depth=\dimen170
Package footmisc Info: Declaring symbol style bringhurst on input line
696.
Package footmisc Info: Declaring symbol style chicago on input line 704.
Package footmisc Info: Declaring symbol style wiley on input line 713.
```

```
Package footmisc Info: Declaring symbol style lamport-robust on input
line 724.

Package footmisc Info: Declaring symbol style lamport* on input line 744.
Package footmisc Info: Declaring symbol style lamport*-robust on input
line 765
.
) (c:/texlive/2024/texmf-dist/tex/latex/fancyhdr/fancyhdr.sty
Package: fancyhdr 2024/07/23 v4.3.1 Extensive control of page headers and
foote
rs
\f@nch@headwidth=\skip145
\f@nch@O@elh=\skip146
\f@nch@O@erh=\skip147
\f@nch@O@olh=\skip148
\f@nch@O@orh=\skip149
\f@nch@O@elf=\skip150
\f@nch@O@erf=\skip151
\f@nch@O@olf=\skip152
\f@nch@O@orf=\skip153
) (c:/texlive/2024/texmf-dist/tex/generic/alphalph/alphalph.sty
Package: alphalph 2019/12/09 v2.6 Convert numbers to letters (HO)
(c:/texlive/2024/texmf-dist/tex/generic/intcalc/intcalc.sty
Package: intcalc 2019/12/15 v1.3 Expandable calculations with integers
(HO)
))
\c@authorfn=\count292
(c:/texlive/2024/texmf-dist/tex/latex/abstract/abstract.sty
Package: abstract 2009/06/08 v1.2a configurable abstracts
\abstitleskip=\skip154
\absleftindent=\skip155
\absrightindent=\skip156
\absparindent=\skip157
\absparsep=\skip158
)
Package newfloat Info: New float `keypoints' with options
`placement=t!,name=kp
t' on input line 291.
\c@keypoints=\count293
\newfloat@ftype=\count294
Package newfloat Info: float type `keypoints'=8 on input line 291.
(c:/texlive/2024/texmf-dist/tex/latex/enumitem/enumitem.sty
Package: enumitem 2019/06/20 v3.9 Customized lists
\labelindent=\skip159
\enit@outerparindent=\dimen171
\enit@toks=\toks37
\enit@inbox=\box67
\enit@count@id=\count295
\enitdp@description=\count296
) (c:/texlive/2024/texmf-dist/tex/latex/quoting/quoting.sty
Package: quoting 2014/01/28 v0.1c Consolidated environment for displayed
text
\quo@toppartop=\skip160
) (c:/texlive/2024/texmf-dist/tex/latex/sttools/stfloats.sty
```

```
Package: stfloats 2017/03/27 v3.3 Improve float mechanism and
baselineskip sett
ings
\@dblbotnum=\count297
\c@dblbotnumber=\count298
) (c:/texlive/2024/texmf-dist/tex/latex/booktabs/booktabs.sty
Package: booktabs 2020/01/12 v1.61803398 Publication quality tables
\heavyrulewidth=\dimen172
\lightrulewidth=\dimen173
\cmidrulewidth=\dimen174
\belowrulesep=\dimen175
\belowbottomsep=\dimen176
\aboverulesep=\dimen177
\abovetopsep=\dimen178
\cmidrulesep=\dimen179
\cmidrulekern=\dimen180
\defaultaddspace=\dimen181
\@cmidla=\count299
\@cmidlb=\count300
\@aboverulesep=\dimen182
\@belowrulesep=\dimen183
\@thisruleclass=\count301
\@lastruleclass=\count302
\@thisrulewidth=\dimen184
) (c:/texlive/2024/texmf-dist/tex/latex/tools/tabularx.sty
Package: tabularx 2023/12/11 v2.12a `tabularx' package (DPC)
\TX@col@width=\dimen185
\TX@old@table=\dimen186
\TX@old@col=\dimen187
\TX@target=\dimen188
\TX@delta=\dimen189
\TX@cols=\count303
\TX@ftn=\toks38
)
\enitdp@tablenotes=\count304
(c:/texlive/2024/texmf-dist/tex/latex/caption/caption.sty
Package: caption 2023/08/05 v3.6o Customizing captions (AR)
(c:/texlive/2024/texmf-dist/tex/latex/caption/caption3.sty
Package: caption3 2023/07/31 v2.4d caption3 kernel (AR)
\caption@tempdima=\dimen190
\captionmargin=\dimen191
\caption@leftmargin=\dimen192
\caption@rightmargin=\dimen193
\caption@width=\dimen194
\caption@indent=\dimen195
\caption@parindent=\dimen196
\caption@hangindent=\dimen197
Package caption Info: Standard document class detected.
)
\c@caption@flags=\count305
\c@continuedfloat=\count306
Package caption Info: rotating package is loaded.
Package caption Info: scrextend package is loaded.
\caption@addmargin@hsize=\dimen198
```

```
\caption@addmargin@linewidth=\dimen199
) (c:/texlive/2024/texmf-dist/tex/latex/natbib/natbib.sty
Package: natbib 2010/09/13 8.31b (PWD, AO)
\bibhang=\skip161
\bibsep=\skip162
LaTeX Info: Redefining \cite on input line 694.
\c@NAT@ctr=\count307
)) (c:/texlive/2024/texmf-dist/tex/latex/siunitx/siunitx.sty
Package: siunitx 2024-06-24 v3.3.19 A comprehensive (SI) units package
\l__siunitx_number_uncert_offset_int=\count308
\l__siunitx_number_exponent_fixed_int=\count309
\l__siunitx_number_min_decimal_int=\count310
\l__siunitx_number_min_integer_int=\count311
\l__siunitx_number_round_precision_int=\count312
\l__siunitx_number_lower_threshold_int=\count313
\l__siunitx_number_upper_threshold_int=\count314
\l__siunitx_number_group_first_int=\count315
\l__siunitx_number_group_size_int=\count316
\l__siunitx_number_group_minimum_int=\count317
\l__siunitx_angle_tmp_dim=\dimen256
\l__siunitx_angle_marker_box=\box68
\l__siunitx_angle_unit_box=\box69
\l__siunitx_compound_count_int=\count318
(c:/texlive/2024/texmf-dist/tex/latex/translations/translations.sty
Package: translations 2022/02/05 v1.12 internationalization of LaTeX2e
packages
 (CN)
) (c:/texlive/2024/texmf-dist/tex/latex/amsmath/amstext.sty
Package: amstext 2021/08/26 v2.01 AMS text
(c:/texlive/2024/texmf-dist/tex/latex/amsmath/amsgen.sty
File: amsgen.sty 1999/11/30 v2.0 generic functions
\@emptytoks=\toks39
\ex@=\dimen257
))
\l__siunitx_table_tmp_box=\box70
\l__siunitx_table_tmp_dim=\dimen258
\l__siunitx_table_column_width_dim=\dimen259
\l__siunitx_table_integer_box=\box71
\l__siunitx_table_decimal_box=\box72
\l__siunitx_table_uncert_box=\box73
\l__siunitx_table_before_box=\box74
\l__siunitx_table_after_box=\box75
\l__siunitx_table_before_dim=\dimen260
\l__siunitx_table_carry_dim=\dimen261
\l__siunitx_unit_tmp_int=\count319
\l__siunitx_unit_position_int=\count320
\l__siunitx_unit_total_int=\count321
) (c:/texlive/2024/texmf-dist/tex/latex/hyperref/hyperref.sty
Package: hyperref 2024-07-10 v7.01j Hypertext links for LaTeX
(c:/texlive/2024/texmf-dist/tex/generic/pdfescape/pdfescape.sty
Package: pdfescape 2019/12/09 v1.15 Implements pdfTeX's escape features
(HO)
) (c:/texlive/2024/texmf-dist/tex/latex/hycolor/hycolor.sty
```

```
Package: hycolor 2020-01-27 v1.10 Color options for hyperref/bookmark
(HO)
) (c:/texlive/2024/texmf-dist/tex/latex/hyperref/nameref.sty
Package: nameref 2023-11-26 v2.56 Cross-referencing by name of section
(c:/texlive/2024/texmf-dist/tex/latex/refcount/refcount.sty
Package: refcount 2019/12/15 v3.6 Data extraction from label references
(HO)
) (c:/texlive/2024/texmf-
dist/tex/generic/gettitlestring/gettitlestring.sty
Package: gettitlestring 2019/12/15 v1.6 Cleanup title references (HO)
)
\c@section@level=\count322
) (c:/texlive/2024/texmf-dist/tex/generic/stringenc/stringenc.sty
Package: stringenc 2019/11/29 v1.12 Convert strings between diff.
encodings (HO
)
)
\@linkdim=\dimen262
\Hy@linkcounter=\count323
\Hy@pagecounter=\count324
(c:/texlive/2024/texmf-dist/tex/latex/hyperref/pd1enc.def
File: pd1enc.def 2024-07-10 v7.01j Hyperref: PDFDocEncoding definition
(HO)
Now handling font encoding PD1 ...
... no UTF-8 mapping file for font encoding PD1
)
\Hy@SavedSpaceFactor=\count325
(c:/texlive/2024/texmf-dist/tex/latex/hyperref/puenc.def
File: puenc.def 2024-07-10 v7.01j Hyperref: PDF Unicode definition (HO)
Now handling font encoding PU ...
... no UTF-8 mapping file for font encoding PU
)
Package hyperref Info: Option `colorlinks' set `true' on input line 4040.
Package hyperref Info: Hyper figures OFF on input line 4157.
Package hyperref Info: Link nesting OFF on input line 4162.
Package hyperref Info: Hyper index ON on input line 4165.
Package hyperref Info: Plain pages OFF on input line 4172.
Package hyperref Info: Backreferencing OFF on input line 4177.
Package hyperref Info: Implicit mode ON; LaTeX internals redefined.
Package hyperref Info: Bookmarks ON on input line 4424.
\c@Hy@tempcnt=\count326
LaTeX Info: Redefining \url on input line 4763.
\XeTeXLinkMargin=\dimen263
(c:/texlive/2024/texmf-dist/tex/generic/bitset/bitset.sty
Package: bitset 2019/12/09 v1.3 Handle bit-vector datatype (HO)
(c:/texlive/2024/texmf-dist/tex/generic/bigintcalc/bigintcalc.sty
Package: bigintcalc 2019/12/15 v1.5 Expandable calculations on big
integers (HO
)
))
\Fld@menulength=\count327
\Field@Width=\dimen264
\Fld@charsize=\dimen265
Package hyperref Info: Hyper figures OFF on input line 6042.
```

```
Package hyperref Info: Link nesting OFF on input line 6047.
Package hyperref Info: Hyper index ON on input line 6050.
Package hyperref Info: backreferencing OFF on input line 6057.
Package hyperref Info: Link coloring ON on input line 6060.
Package hyperref Info: Link coloring with OCG OFF on input line 6067.
Package hyperref Info: PDF/A mode OFF on input line 6072.
(c:/texlive/2024/texmf-dist/tex/latex/base/atbegshi-ltx.sty
Package: atbegshi-ltx 2021/01/10 v1.0c Emulation of the original atbegshi
package with kernel methods
)
\Hy@abspage=\count328
\c@Item=\count329
\c@Hfootnote=\count330
)
Package hyperref Info: Driver (autodetected): hpdftex.
(c:/texlive/2024/texmf-dist/tex/latex/hyperref/hpdftex.def
File: hpdftex.def 2024-07-10 v7.01j Hyperref driver for pdfTeX
(c:/texlive/2024/texmf-dist/tex/latex/base/atveryend-ltx.sty
Package: atveryend-ltx 2020/08/19 v1.0a Emulation of the original
atveryend pac
kage
with kernel methods
)
\HyAnn@Count=\count331
\Fld@listcount=\count332
\c@bookmark@seq@number=\count333
(c:/texlive/2024/texmf-dist/tex/latex/rerunfilecheck/rerunfilecheck.sty
Package: rerunfilecheck 2022-07-10 v1.10 Rerun checks for auxiliary files
(HO)
(c:/texlive/2024/texmf-dist/tex/generic/uniquecounter/uniquecounter.sty
Package: uniquecounter 2019/12/15 v1.4 Provide unlimited unique counter
(HO)
)
Package uniquecounter Info: New unique counter `rerunfilecheck' on input
line 2
85.
)
\Hy@SectionHShift=\skip163
)
Package translations Info: No language package found. I am going to use
`englis
h' as default language. on input line 63.
LaTeX Font Info:    Trying to load font information for T1+Merriwthr-OsF
on inp
ut line 63.
(c:/texlive/2024/texmf-dist/tex/latex/merriweather/T1Merriwthr-OsF.fd
File: T1Merriwthr-OsF.fd 2020/08/30 (autoinst) Font definitions for
T1/Merriwth
r-OsF.
)
LaTeX Font Info:    Font shape `T1/Merriwthr-OsF/m/n' will be
(Font)              scaled to size 7.5pt on input line 63.
(./main.aux)
\openout1 = `main.aux'.
```

```
LaTeX Font Info:    Checking defaults for OML/cmm/m/it on input line 63.
LaTeX Font Info:    ... okay on input line 63.
LaTeX Font Info:    Checking defaults for OMS/cmsy/m/n on input line 63.
LaTeX Font Info:    ... okay on input line 63.
LaTeX Font Info:    Checking defaults for OT1/cmr/m/n on input line 63.
LaTeX Font Info:    ... okay on input line 63.
LaTeX Font Info:    Checking defaults for T1/cmr/m/n on input line 63.
LaTeX Font Info:    ... okay on input line 63.
LaTeX Font Info:    Checking defaults for TS1/cmr/m/n on input line 63.
LaTeX Font Info:    ... okay on input line 63.
LaTeX Font Info:    Checking defaults for OMX/cmex/m/n on input line 63.
LaTeX Font Info:    ... okay on input line 63.
LaTeX Font Info:    Checking defaults for U/cmr/m/n on input line 63.
LaTeX Font Info:    ... okay on input line 63.
LaTeX Font Info:    Checking defaults for PD1/pdf/m/n on input line 63.
LaTeX Font Info:    ... okay on input line 63.
LaTeX Font Info:    Checking defaults for PU/pdf/m/n on input line 63.
LaTeX Font Info:    ... okay on input line 63.
LaTeX Info: Redefining \microtypecontext on input line 63.
Package microtype Info: Applying patch `item' on input line 63.
Package microtype Info: Applying patch `toc' on input line 63.
Package microtype Info: Applying patch `eqnum' on input line 63.
Package microtype Info: Applying patch `footnote' on input line 63.
Package microtype Info: Applying patch `verbatim' on input line 63.
Package microtype Info: Generating PDF output.
Package microtype Info: Character protrusion enabled (level 2).
Package microtype Info: Using default protrusion set `alltext'.
Package microtype Info: Automatic font expansion enabled (level 2),
(microtype)             stretch: 20, shrink: 20, step: 1, non-selected.
Package microtype Info: Using default expansion set `alltext-nott'.
LaTeX Info: Redefining \showhyphens on input line 63.
Package microtype Info: No adjustment of tracking.
Package microtype Info: No adjustment of interword spacing.
Package microtype Info: No adjustment of character kerning.
Package microtype Info: Loading generic protrusion settings for font
family
(microtype)             `Merriwthr-OsF' (encoding: T1).
(microtype)             For optimal results, create family-specific
settings.
(microtype)             See the microtype manual for details.
LaTeX Font Info:    Redeclaring symbol font `operators' on input line 63.
LaTeX Font Info:    Encoding `OT1' has changed to `T1' for symbol font
(Font)              `operators' in the math version `normal' on input
line 63.
LaTeX Font Info:    Overwriting symbol font `operators' in version
`normal'
(Font)                  OT1/cmr/m/n --> T1/Merriwthr-OsF/m/up on input
line 63.

LaTeX Font Info:    Encoding `OT1' has changed to `T1' for symbol font
(Font)              `operators' in the math version `bold' on input line
63.
LaTeX Font Info:    Overwriting symbol font `operators' in version `bold'
```

```
(Font)                  OT1/cmr/bx/n --> T1/Merriwthr-OsF/m/up on input
line 63
.
LaTeX Font Info:    Overwriting symbol font `operators' in version `bold'
(Font)                  T1/Merriwthr-OsF/m/up --> T1/Merriwthr-OsF/b/up
on inpu
t line 63.
LaTeX Font Info:    Redeclaring math alphabet \mathbf on input line 63.
LaTeX Font Info:    Overwriting math alphabet `\mathbf' in version
`normal'
(Font)                  OT1/cmr/bx/n --> T1/Merriwthr-OsF/b/up on input
line 63
.
LaTeX Font Info:    Overwriting math alphabet `\mathbf' in version `bold'
(Font)                  OT1/cmr/bx/n --> T1/Merriwthr-OsF/b/up on input
line 63
.
LaTeX Font Info:    Redeclaring math alphabet \mathsf on input line 63.
LaTeX Font Info:    Overwriting math alphabet `\mathsf' in version
`normal'
(Font)                  OT1/cmss/m/n --> T1/MerriwthrSans-OsF/m/up on
input lin
e 63.
LaTeX Font Info:    Overwriting math alphabet `\mathsf' in version `bold'
(Font)                  OT1/cmss/bx/n --> T1/MerriwthrSans-OsF/m/up on
input li
ne 63.
LaTeX Font Info:    Redeclaring math alphabet \mathit on input line 63.
LaTeX Font Info:    Overwriting math alphabet `\mathit' in version
`normal'
(Font)                  OT1/cmr/m/it --> T1/Merriwthr-OsF/m/it on input
line 63
.
LaTeX Font Info:    Overwriting math alphabet `\mathit' in version `bold'
(Font)                  OT1/cmr/bx/it --> T1/Merriwthr-OsF/m/it on input
line 6
3.
LaTeX Font Info:    Redeclaring math alphabet \mathtt on input line 63.
LaTeX Font Info:    Overwriting math alphabet `\mathtt' in version
`normal'
(Font)                  OT1/cmtt/m/n --> T1/lmtt/m/up on input line 63.
LaTeX Font Info:    Overwriting math alphabet `\mathtt' in version `bold'
(Font)                  OT1/cmtt/m/n --> T1/lmtt/m/up on input line 63.
LaTeX Font Info:    Overwriting math alphabet `\mathsf' in version `bold'
(Font)                  T1/MerriwthrSans-OsF/m/up --> T1/MerriwthrSans-
OsF/b/up
 on input line 63.
LaTeX Font Info:    Overwriting math alphabet `\mathit' in version `bold'
(Font)                  T1/Merriwthr-OsF/m/it --> T1/Merriwthr-OsF/b/it
on inpu
t line 63.
\c@mv@tabular=\count334
\c@mv@boldtabular=\count335
(c:/texlive/2024/texmf-dist/tex/context/base/mkii/supp-pdf.mkii
```

```
[Loading MPS to PDF converter (version 2006.09.02).]
\scratchcounter=\count336
\scratchdimen=\dimen266
\scratchbox=\box76
\nofMPsegments=\count337
\nofMParguments=\count338
\everyMPshowfont=\toks40
\MPscratchCnt=\count339
\MPscratchDim=\dimen267
\MPnumerator=\count340
\makeMPintoPDFobject=\count341
\everyMPtoPDFconversion=\toks41
) (c:/texlive/2024/texmf-dist/tex/latex/epstopdf-pkg/epstopdf-base.sty
Package: epstopdf-base 2020-01-24 v2.11 Base part for package epstopdf
Package epstopdf-base Info: Redefining graphics rule for `.eps' on input
line 4
85.
(c:/texlive/2024/texmf-dist/tex/latex/latexconfig/epstopdf-sys.cfg
File: epstopdf-sys.cfg 2010/07/13 v1.3 Configuration of (r)epstopdf for
TeX Liv
e
))
*geometry* driver: auto-detecting
*geometry* detected driver: pdftex
*geometry* verbose mode - [ preamble ] result:
* driver: pdftex
* paper: a4paper
* layout: <same size as paper>
* layoutoffset:(h,v)=(0.0pt,0.0pt)
* modes: includefoot twoside
* h-part:(L,W,R)=(54.64pt, 488.22787pt, 54.64pt)
* v-part:(T,H,B)=(66.0pt, 745.04684pt, 34.0pt)
* \paperwidth=597.50787pt
* \paperheight=845.04684pt
* \textwidth=488.22787pt
* \textheight=715.04684pt
* \oddsidemargin=-17.62999pt
* \evensidemargin=-17.62999pt
* \topmargin=-47.76999pt
* \headheight=17.5pt
* \headsep=24.0pt
* \topskip=10.0pt
* \footskip=30.0pt
* \marginparwidth=48.0pt
* \marginparsep=10.0pt
* \columnsep=18.0pt
* \skip\footins=22.0pt plus 2.0pt
* \hoffset=0.0pt
* \voffset=0.0pt
* \mag=1000
* \@twocolumntrue
* \@twosidetrue
* \@mparswitchtrue
* \@reversemarginfalse
```

```
* (1in=72.27pt=25.4mm, 1cm=28.453pt)

Package caption Info: Begin \AtBeginDocument code.
Package caption Info: hyperref package is loaded.
Package caption Info: End \AtBeginDocument code.

(c:/texlive/2024/texmf-dist/tex/latex/translations/translations-basic-
dictionar
y-english.trsl
File: translations-basic-dictionary-english.trsl (english translation
file `tra
nslations-basic-dictionary')
)
Package translations Info: loading dictionary `translations-basic-
dictionary' f
or `english'. on input line 63.
Package hyperref Info: Link coloring ON on input line 63.
(./main.out) (./main.out)
\@outlinefile=\write3
\openout3 = `main.out'.

\@gscitedetails=\box77
\@gscitedetailsheight=\skip164
\@gsheadbox=\box78
\@gsheadboxheight=\skip165
LaTeX Font Info:    Font shape `T1/Merriwthr-OsF/b/n' will be
(Font)              scaled to size 6.5pt on input line 63.
LaTeX Font Info:    Calculating math sizes for size <7.5> on input line
63.

LaTeX Font Warning: Font shape `T1/Merriwthr-OsF/m/up' undefined
(Font)              using `T1/Merriwthr-OsF/m/n' instead on input line
63.

LaTeX Font Info:    Font shape `T1/Merriwthr-OsF/m/up' will be
(Font)              scaled to size 6.24973pt on input line 63.
LaTeX Font Info:    Font shape `T1/Merriwthr-OsF/m/up' will be
(Font)              scaled to size 5.24997pt on input line 63.
LaTeX Font Info:    Trying to load font information for U+eur on input
line 63.

(c:/texlive/2024/texmf-dist/tex/latex/amsfonts/ueur.fd
File: ueur.fd 2013/01/14 v3.01 Euler Roman
) (c:/texlive/2024/texmf-dist/tex/latex/microtype/mt-eur.cfg
File: mt-eur.cfg 2006/07/31 v1.1 microtype config. file: AMS Euler Roman
(RS)
)

LaTeX Font Warning: Font shape `OMS/cmsy/m/n' in size <7.5> not available
(Font)              size <7> substituted on input line 63.

LaTeX Font Info:    External font `cmex10' loaded for size
(Font)              <7.5> on input line 63.
LaTeX Font Info:    External font `cmex10' loaded for size
```

```
(Font)              <6.24973> on input line 63.
LaTeX Font Info:    External font `cmex10' loaded for size
(Font)              <5.24997> on input line 63.
LaTeX Font Info:    Trying to load font information for U+euf on input
line 63.

(c:/texlive/2024/texmf-dist/tex/latex/amsfonts/ueuf.fd
File: ueuf.fd 2013/01/14 v3.01 Euler Fraktur
) (c:/texlive/2024/texmf-dist/tex/latex/microtype/mt-euf.cfg
File: mt-euf.cfg 2006/07/03 v1.1 microtype config. file: AMS Euler
Fraktur (RS)

)
LaTeX Font Info:    Trying to load font information for U+eus on input
line 63.

(c:/texlive/2024/texmf-dist/tex/latex/amsfonts/ueus.fd
File: ueus.fd 2013/01/14 v3.01 Euler Script
) (c:/texlive/2024/texmf-dist/tex/latex/microtype/mt-eus.cfg
File: mt-eus.cfg 2006/07/28 v1.2 microtype config. file: AMS Euler Script
(RS)
)
LaTeX Font Info:    Trying to load font information for U+euex on input
line 63
.
(c:/texlive/2024/texmf-dist/tex/latex/amsfonts/ueuex.fd
File: ueuex.fd 2013/01/14 v3.01 Euler extra symbols
)

LaTeX Font Warning: Font shape `OML/cmm/m/it' in size <7.5> not available
(Font)              size <7> substituted on input line 63.

LaTeX Font Info:    Font shape `T1/Merriwthr-OsF/m/n' will be
(Font)              scaled to size 6.24973pt on input line 63.
LaTeX Font Info:    Font shape `T1/Merriwthr-OsF/m/n' will be
(Font)              scaled to size 5.24997pt on input line 63.
LaTeX Font Info:    Font shape `T1/Merriwthr-OsF/m/it' will be
(Font)              scaled to size 7.5pt on input line 63.
LaTeX Font Info:    Font shape `T1/Merriwthr-OsF/m/it' will be
(Font)              scaled to size 6.24973pt on input line 63.
LaTeX Font Info:    Font shape `T1/Merriwthr-OsF/m/it' will be
(Font)              scaled to size 5.24997pt on input line 63.
LaTeX Font Info:    Font shape `T1/Merriwthr-OsF/m/n' will be
(Font)              scaled to size 8.0pt on input line 63.
LaTeX Font Info:    Font shape `T1/Merriwthr-OsF/m/it' will be
(Font)              scaled to size 8.0pt on input line 63.
LaTeX Font Info:    Font shape `T1/Merriwthr-OsF/b/it' will be
(Font)              scaled to size 8.0pt on input line 63.
TextBlockOrigin set to 4pc+6.64pt x 4pc+6pt
<gigascience-logo.pdf, id=117, 99.37125pt x 33.12375pt>
File: gigascience-logo.pdf Graphic file (type pdf)
<use gigascience-logo.pdf>
Package pdftex.def Info: gigascience-logo.pdf  used on input line 87.
(pdftex.def)             Requested size: 126.00902pt x 42.0pt.
```

```
Overfull \hbox (54.64pt too wide) in paragraph at lines 87--87
[][]
 []

LaTeX Font Info:    Font shape `T1/Merriwthr-OsF/m/n' will be
(Font)              scaled to size 14.0pt on input line 87.
LaTeX Font Info:    Font shape `T1/Merriwthr-OsF/m/n' will be
(Font)              scaled to size 8.99997pt on input line 87.
LaTeX Font Info:    Calculating math sizes for size <14> on input line
87.
LaTeX Font Info:    Font shape `T1/Merriwthr-OsF/m/up' will be
(Font)              scaled to size 14.0pt on input line 87.
LaTeX Font Info:    Font shape `T1/Merriwthr-OsF/m/up' will be
(Font)              scaled to size 11.66617pt on input line 87.
LaTeX Font Info:    Font shape `T1/Merriwthr-OsF/m/up' will be
(Font)              scaled to size 9.79996pt on input line 87.
LaTeX Font Info:    External font `cmex10' loaded for size
(Font)              <14> on input line 87.
LaTeX Font Info:    External font `cmex10' loaded for size
(Font)              <11.66617> on input line 87.
LaTeX Font Info:    External font `cmex10' loaded for size
(Font)              <9.79996> on input line 87.
LaTeX Font Info:    Font shape `T1/Merriwthr-OsF/m/n' will be
(Font)              scaled to size 11.66617pt on input line 87.
LaTeX Font Info:    Font shape `T1/Merriwthr-OsF/m/n' will be
(Font)              scaled to size 9.79996pt on input line 87.
LaTeX Font Info:    Font shape `T1/Merriwthr-OsF/m/it' will be
(Font)              scaled to size 14.0pt on input line 87.
LaTeX Font Info:    Font shape `T1/Merriwthr-OsF/m/it' will be
(Font)              scaled to size 11.66617pt on input line 87.
LaTeX Font Info:    Font shape `T1/Merriwthr-OsF/m/it' will be
(Font)              scaled to size 9.79996pt on input line 87.
LaTeX Font Info:    Font shape `T1/Merriwthr-OsF/b/n' will be
(Font)              scaled to size 18.0pt on input line 87.
LaTeX Font Info:    Font shape `T1/Merriwthr-OsF/m/n' will be
(Font)              scaled to size 13.0pt on input line 87.
LaTeX Font Info:    Calculating math sizes for size <13> on input line
87.
LaTeX Font Info:    Font shape `T1/Merriwthr-OsF/m/up' will be
(Font)              scaled to size 13.0pt on input line 87.
LaTeX Font Info:    Font shape `T1/Merriwthr-OsF/m/up' will be
(Font)              scaled to size 10.83287pt on input line 87.
LaTeX Font Info:    Font shape `T1/Merriwthr-OsF/m/up' will be
(Font)              scaled to size 9.09996pt on input line 87.

LaTeX Font Warning: Font shape `OMS/cmsy/m/n' in size <13> not available
(Font)              size <12> substituted on input line 87.

LaTeX Font Info:    External font `cmex10' loaded for size
(Font)              <13> on input line 87.
LaTeX Font Info:    External font `cmex10' loaded for size
(Font)              <10.83287> on input line 87.
LaTeX Font Info:    External font `cmex10' loaded for size
```

```
(Font)              <9.09996> on input line 87.

LaTeX Font Warning: Font shape `OML/cmm/m/it' in size <13> not available
(Font)              size <12> substituted on input line 87.

LaTeX Font Info:    Font shape `T1/Merriwthr-OsF/m/n' will be
(Font)              scaled to size 10.83287pt on input line 87.
LaTeX Font Info:    Font shape `T1/Merriwthr-OsF/m/n' will be
(Font)              scaled to size 9.09996pt on input line 87.
LaTeX Font Info:    Font shape `T1/Merriwthr-OsF/m/it' will be
(Font)              scaled to size 13.0pt on input line 87.
LaTeX Font Info:    Font shape `T1/Merriwthr-OsF/m/it' will be
(Font)              scaled to size 10.83287pt on input line 87.
LaTeX Font Info:    Font shape `T1/Merriwthr-OsF/m/it' will be
(Font)              scaled to size 9.09996pt on input line 87.
LaTeX Font Info:    Trying to load font information for TS1+Merriwthr-OsF
on in
put line 87.
(c:/texlive/2024/texmf-dist/tex/latex/merriweather/TS1Merriwthr-OsF.fd
File: TS1Merriwthr-OsF.fd 2020/08/30 (autoinst) Font definitions for
TS1/Merriw
thr-OsF.
)
LaTeX Font Info:    Font shape `TS1/Merriwthr-OsF/m/n' will be
(Font)              scaled to size 10.83287pt on input line 87.
Package microtype Info: Loading generic protrusion settings for font
family
(microtype)             `Merriwthr-OsF' (encoding: TS1).
(microtype)             For optimal results, create family-specific
settings.
(microtype)             See the microtype manual for details.
LaTeX Font Info:    Font shape `T1/Merriwthr-OsF/m/n' will be
(Font)              scaled to size 9.0pt on input line 87.
LaTeX Font Info:    Font shape `T1/Merriwthr-OsF/m/up' will be
(Font)              scaled to size 9.0pt on input line 87.
LaTeX Font Info:    Font shape `T1/Merriwthr-OsF/m/up' will be
(Font)              scaled to size 7.0pt on input line 87.
LaTeX Font Info:    Font shape `T1/Merriwthr-OsF/m/up' will be
(Font)              scaled to size 5.0pt on input line 87.
LaTeX Font Info:    External font `cmex10' loaded for size
(Font)              <9> on input line 87.
LaTeX Font Info:    External font `cmex10' loaded for size
(Font)              <7> on input line 87.
LaTeX Font Info:    External font `cmex10' loaded for size
(Font)              <5> on input line 87.
LaTeX Font Info:    Font shape `T1/Merriwthr-OsF/m/n' will be
(Font)              scaled to size 7.0pt on input line 87.
LaTeX Font Info:    Font shape `T1/Merriwthr-OsF/m/n' will be
(Font)              scaled to size 5.0pt on input line 87.
LaTeX Font Info:    Font shape `T1/Merriwthr-OsF/m/it' will be
(Font)              scaled to size 9.0pt on input line 87.
LaTeX Font Info:    Font shape `T1/Merriwthr-OsF/m/it' will be
(Font)              scaled to size 7.0pt on input line 87.
LaTeX Font Info:    Font shape `T1/Merriwthr-OsF/m/it' will be
```

```
(Font)              scaled to size 5.0pt on input line 87.
LaTeX Font Info:    Font shape `T1/Merriwthr-OsF/m/n' will be
(Font)              scaled to size 6.5pt on input line 87.
LaTeX Font Info:    Calculating math sizes for size <6.5> on input line
87.
LaTeX Font Info:    Font shape `T1/Merriwthr-OsF/m/up' will be
(Font)              scaled to size 6.5pt on input line 87.
LaTeX Font Info:    Font shape `T1/Merriwthr-OsF/m/up' will be
(Font)              scaled to size 5.41643pt on input line 87.
LaTeX Font Info:    Font shape `T1/Merriwthr-OsF/m/up' will be
(Font)              scaled to size 4.54997pt on input line 87.

LaTeX Font Warning: Font shape `OMS/cmsy/m/n' in size <6.5> not available
(Font)              size <6> substituted on input line 87.


LaTeX Font Warning: Font shape `OMS/cmsy/m/n' in size <5.41643> not
available
(Font)              size <5> substituted on input line 87.


LaTeX Font Warning: Font shape `OMS/cmsy/m/n' in size <4.54997> not
available
(Font)              size <5> substituted on input line 87.

LaTeX Font Info:    External font `cmex10' loaded for size
(Font)              <6.5> on input line 87.
LaTeX Font Info:    External font `cmex10' loaded for size
(Font)              <5.41643> on input line 87.
LaTeX Font Info:    External font `cmex10' loaded for size
(Font)              <4.54997> on input line 87.

LaTeX Font Warning: Font shape `OML/cmm/m/it' in size <6.5> not available
(Font)              size <6> substituted on input line 87.


LaTeX Font Warning: Font shape `OML/cmm/m/it' in size <5.41643> not
available
(Font)              size <5> substituted on input line 87.


LaTeX Font Warning: Font shape `OML/cmm/m/it' in size <4.54997> not
available
(Font)              size <5> substituted on input line 87.

LaTeX Font Info:    Font shape `T1/Merriwthr-OsF/m/n' will be
(Font)              scaled to size 5.41643pt on input line 87.
LaTeX Font Info:    Font shape `T1/Merriwthr-OsF/m/n' will be
(Font)              scaled to size 4.54997pt on input line 87.
LaTeX Font Info:    Font shape `T1/Merriwthr-OsF/m/it' will be
(Font)              scaled to size 6.5pt on input line 87.
LaTeX Font Info:    Font shape `T1/Merriwthr-OsF/m/it' will be
(Font)              scaled to size 5.41643pt on input line 87.
LaTeX Font Info:    Font shape `T1/Merriwthr-OsF/m/it' will be
```

```
(Font)              scaled to size 4.54997pt on input line 87.
LaTeX Font Info:    Font shape `TS1/Merriwthr-OsF/m/n' will be
(Font)              scaled to size 5.41643pt on input line 87.

Overfull \hbox (54.64pt too wide) in paragraph at lines 87--87
[][][]
 []

LaTeX Font Info:    Font shape `T1/Merriwthr-OsF/b/n' will be
(Font)              scaled to size 10.0pt on input line 87.
LaTeX Font Info:    Font shape `T1/Merriwthr-OsF/b/n' will be
(Font)              scaled to size 8.0pt on input line 87.
LaTeX Font Info:    Trying to load font information for T1+lmtt on input
line 8
7.
(c:/texlive/2024/texmf-dist/tex/latex/lm/t1lmtt.fd
File: t1lmtt.fd 2015/05/01 v1.6.1 Font defs for Latin Modern
)
Package microtype Info: Loading generic protrusion settings for font
family
(microtype)             `lmtt' (encoding: T1).
(microtype)             For optimal results, create family-specific
settings.
(microtype)             See the microtype manual for details.
LaTeX Font Info:    Font shape `T1/Merriwthr-OsF/m/up' will be
(Font)              scaled to size 8.0pt on input line 87.
LaTeX Font Info:    Font shape `T1/Merriwthr-OsF/m/up' will be
(Font)              scaled to size 6.0pt on input line 87.
LaTeX Font Info:    External font `cmex10' loaded for size
(Font)              <8> on input line 87.
LaTeX Font Info:    External font `cmex10' loaded for size
(Font)              <6> on input line 87.
LaTeX Font Info:    Font shape `T1/Merriwthr-OsF/m/n' will be
(Font)              scaled to size 6.0pt on input line 87.
LaTeX Font Info:    Font shape `T1/Merriwthr-OsF/m/it' will be
(Font)              scaled to size 6.0pt on input line 87.

Overfull \hbox (54.64pt too wide) in paragraph at lines 87--87
[][][]
 []


LaTeX Warning: Optional argument of \twocolumn too tall on page 1.


Underfull \vbox (badness 10000) has occurred while \output is active []




Underfull \vbox (badness 10000) has occurred while \output is active []
```

```
LaTeX Font Info:    Font shape `T1/Merriwthr-OsF/m/n' will be
(Font)              scaled to size 7.8pt on input line 87.
LaTeX Font Info:    Font shape `T1/Merriwthr-OsF/b/n' will be
(Font)              scaled to size 7.8pt on input line 87.
[1{c:/texlive/2024/texmf-
var/fonts/map/pdftex/updmap/pdftex.map}{c:/texlive/202
4/texmf-
dist/fonts/enc/dvips/merriweather/merriwthr_posqbl.enc}{c:/texlive/2024
/texmf-
dist/fonts/enc/dvips/merriweather/merriwthr_owzwzj.enc}{c:/texlive/2024/
texmf-dist/fonts/enc/dvips/lm/lm-ec.enc}


 <./gigascience-logo.pdf>]
LaTeX Font Info:    Font shape `T1/Merriwthr-OsF/b/n' will be
(Font)              scaled to size 7.5pt on input line 91.

Package natbib Warning: Citation `clish_metabolomics_2015' on page 2
undefined
on input line 91.


Package natbib Warning: Citation `emwas_nmr-based_2013' on page 2
undefined on
input line 91.


Package natbib Warning: Citation `zacharias_statistical_2018' on page 2
undefin
ed on input line 91.


Package natbib Warning: Citation `emwas_nmr_2019' on page 2 undefined on
input
line 91.


Package natbib Warning: Citation `zacharias_microbiome_2022' on page 2
undefine
d on input line 91.


Package natbib Warning: Citation `haug_metabolightsopen-access_2013' on
page 2
undefined on input line 91.


Package natbib Warning: Citation `ara_metabolonote_2015' on page 2
undefined on
 input line 91.


Package natbib Warning: Citation `ferry-dumazet_mery-b_2011' on page 2
undefine
```

```
d on input line 91.


Package natbib Warning: Citation `sud_metabolomics_2016' on page 2
undefined on
 input line 91.


Package natbib Warning: Citation `tzanakis_methos_2022' on page 2
undefined on
input line 91.


Package natbib Warning: Citation `temprosa_comets_2022' on page 2
undefined on
input line 91.


Package natbib Warning: Citation `haug_metabolights_2020' on page 2
undefined o
n input line 91.


Package natbib Warning: Citation `wilkinson_fair_2016' on page 2
undefined on i
nput line 91.


Package natbib Warning: Citation `powell_metabolomics_2022' on page 2
undefined
 on input line 91.


Package natbib Warning: Citation `haug_metabolightsopen-access_2013' on
page 2
undefined on input line 93.


Package natbib Warning: Citation `sud_metabolomics_2016' on page 2
undefined on
 input line 93.


Package natbib Warning: Citation `keane_growing_2021' on page 2 undefined
on in
put line 93.

LaTeX Font Info:    Font shape `T1/Merriwthr-OsF/m/up' will be
(Font)              scaled to size 7.5pt on input line 95.

LaTeX Warning: File `Graphics/fig1.png' not found on input line 100.
```

```
! Package pdftex.def Error: File `Graphics/fig1.png' not found: using
draft set
ting.

See the pdftex.def package documentation for explanation.
Type  H <return>  for immediate help.
 ...

l.100 ...[width=0.85\textwidth]{Graphics/fig1.png}

Try typing  <return>  to proceed.
If that doesn't work, type  X <return>  to quit.

LaTeX Font Info:    Font shape `T1/Merriwthr-OsF/b/n' will be
(Font)              scaled to size 6.0pt on input line 101.
LaTeX Font Info:    Font shape `T1/Merriwthr-OsF/b/n' will be
(Font)              scaled to size 8.5pt on input line 107.


LaTeX Font Info:    Font shape `T1/Merriwthr-OsF/b/sl' in size <7.5> not
availa
ble
(Font)              Font shape `T1/Merriwthr-OsF/b/it' tried instead on
input l
ine 109.
LaTeX Font Info:    Font shape `T1/Merriwthr-OsF/b/it' will be
(Font)              scaled to size 7.5pt on input line 109.

Package natbib Warning: Citation `noauthor_elasticsearch_nodate' on page
2 unde
fined on input line 111.


Package natbib Warning: Citation `noauthor_python_2020' on page 2
undefined on
input line 111.


Package natbib Warning: Citation `wishart_hmdb_2022' on page 2 undefined
on inp
ut line 111.


Package natbib Warning: Citation `noauthor_elasticsearch_nodate-1' on
page 2 un
defined on input line 111.


Package natbib Warning: Citation `noauthor_vuejs_nodate' on page 2
undefined on
 input line 115.
```

```
Package natbib Warning: Citation `bierman_understanding_2014' on page 2
undefin
ed on input line 115.


Package natbib Warning: Citation `noauthor_javascript_nodate' on page 2
undefin
ed on input line 115.


Package natbib Warning: Citation `noauthor_pinia_nodate' on page 2
undefined on
 input line 115.


Package natbib Warning: Citation `noauthor_angular_nodate' on page 2
undefined
on input line 115.


Package natbib Warning: Citation `noauthor_welcome_2023' on page 2
undefined on
 input line 119.


Package natbib Warning: Citation `grinberg_flask_2014' on page 2
undefined on i
nput line 119.


Package natbib Warning: Citation `harris_array_2020' on page 2 undefined
on inp
ut line 119.


Package natbib Warning: Citation `mckinney_data_2010' on page 2 undefined
on in
put line 119.


Package natbib Warning: Citation `hunter_matplotlib_2007' on page 2
undefined o
n input line 119.


Package natbib Warning: Citation `waskom_seaborn_2021' on page 2
undefined on i
nput line 119.


Package natbib Warning: Citation `noauthor_xmltodict_nodate' on page 2
undefine
d on input line 119.
```

```
Package natbib Warning: Citation `noauthor_flask-cors_nodate' on page 2 
undefin
ed on input line 119.


Package natbib Warning: Citation `noauthor_flask-swagger-ui_nodate' on 
page 2 u
ndefined on input line 119.


Package natbib Warning: Citation `noauthor_flask-jwt-extended_nodate' on 
page 2
 undefined on input line 119.


Package natbib Warning: Citation `noauthor_werkzeug_nodate' on page 2 
undefined
 on input line 119.


Package natbib Warning: Citation `noauthor_gunicorn_nodate' on page 2 
undefined
 on input line 119.


Package natbib Warning: Citation `hupp_python-magic_2023' on page 2 
undefined o
n input line 119.


Package natbib Warning: Citation `r_lang' on page 2 undefined on input 
line 120
.


Package natbib Warning: Citation `noauthor_framework_nodate' on page 2 
undefine
d on input line 120.


Package natbib Warning: Citation `klein_affine_2021' on page 2 undefined 
on inp
ut line 120.


Package natbib Warning: Citation `wishart_hmdb_2022' on page 2 undefined 
on inp
ut line 130.



LaTeX Font Info:    Font shape `T1/Merriwthr-OsF/m/it' will be
(Font)              scaled to size 7.8pt on input line 132.
```

```
[2] [3]

Package natbib Warning: Citation `zacharias_analysis_2013' on page 4
undefined
on input line 134.


Package natbib Warning: Citation `zacharias_analysis_2013' on page 4
undefined
on input line 134.


Package natbib Warning: Citation `zacharias_identification_2015' on page
4 unde
fined on input line 134.


Package natbib Warning: Citation `fino2024evaluation' on page 4 undefined
on in
put line 138.


LaTeX Warning: File `Graphics/fig2.png' not found on input line 144.


! Package pdftex.def Error: File `Graphics/fig2.png' not found: using
draft set
ting.

See the pdftex.def package documentation for explanation.
Type  H <return>  for immediate help.
 ...

l.144 ...[width=0.85\textwidth]{Graphics/fig2.png}

Try typing  <return>  to proceed.
If that doesn't work, type  X <return>  to quit.




Package natbib Warning: Citation `noauthor_scalability_nodate' on page 4
undefi
ned on input line 157.



LaTeX Font Info:    Font shape `T1/Merriwthr-OsF/m/n' will be
(Font)              scaled to size 8.5pt on input line 162.
[4] [5]

LaTeX Warning: File `Graphics/fig3.png' not found on input line 165.
```

```
! Package pdftex.def Error: File `Graphics/fig3.png' not found: using
draft set
ting.

See the pdftex.def package documentation for explanation.
Type  H <return>  for immediate help.
 ...

l.165 ...ics[width=1\textwidth]{Graphics/fig3.png}

Try typing  <return>  to proceed.
If that doesn't work, type  X <return>  to quit.


Package natbib Warning: Citation `sreekumar_trends_2022' on page 6
undefined on
 input line 170.


Package natbib Warning: Citation `jones_json_2015' on page 6 undefined on
input
 line 172.


LaTeX Warning: File `Graphics/fig4.png' not found on input line 178.


! Package pdftex.def Error: File `Graphics/fig4.png' not found: using
draft set
ting.

See the pdftex.def package documentation for explanation.
Type  H <return>  for immediate help.
 ...

l.178 ...hics[width=\textwidth]{Graphics/fig4.png}

Try typing  <return>  to proceed.
If that doesn't work, type  X <return>  to quit.




Package natbib Warning: Citation `wishart_hmdb_2022' on page 6 undefined
on inp
ut line 188.


LaTeX Warning: File `Graphics/fig5.png' not found on input line 192.


! Package pdftex.def Error: File `Graphics/fig5.png' not found: using
draft set
ting.
```

```
See the pdftex.def package documentation for explanation.
Type  H <return>  for immediate help.
 ...

l.192 ...ics[width=1\textwidth]{Graphics/fig5.png}

Try typing  <return>  to proceed.
If that doesn't work, type  X <return>  to quit.


LaTeX Warning: File `Graphics/fig6.png' not found on input line 203.


! Package pdftex.def Error: File `Graphics/fig6.png' not found: using
draft set
ting.

See the pdftex.def package documentation for explanation.
Type  H <return>  for immediate help.
 ...

l.203 ...ics[width=1\textwidth]{Graphics/fig6.png}

Try typing  <return>  to proceed.
If that doesn't work, type  X <return>  to quit.


Underfull \vbox (badness 1057) has occurred while \output is active []



[6]

Package natbib Warning: Citation `kellum2012kidney' on page 7 undefined
on inpu
t line 215.




Underfull \vbox (badness 10000) has occurred while \output is active []



[7] [8] [9] [10]


Package natbib Warning: Citation `haug_metabolightsopen-access_2013' on
page 11
 undefined on input line 222.
```

Package natbib Warning: Citation `sud_metabolomics_2016' on page 11
undefined o
n input line 222.

Package natbib Warning: Citation `ara_metabolonote_2015' on page 11
undefined o
n input line 222.

Package natbib Warning: Citation `ferry-dumazet_mery-b_2011' on page 11
undefin
ed on input line 222.

Package natbib Warning: Citation `ara_metabolonote_2015' on page 11
undefined o
n input line 222.

Package natbib Warning: Citation `ferry-dumazet_mery-b_2011' on page 11
undefin
ed on input line 222.

Package natbib Warning: Citation `temprosa_comets_2022' on page 11
undefined on
input line 224.

Package natbib Warning: Citation `tzanakis_methos_2022' on page 11
undefined on
input line 224.

Package natbib Warning: Citation `allaway2019engaging' on page 11
undefined on
input line 226.

Package natbib Warning: Citation `allaway2019engaging' on page 11
undefined on
input line 226.

Package natbib Warning: Citation `wolstencroft2015seek' on page 11
undefined on
input line 226.

Package natbib Warning: Citation `wolstencroft2017fairdomhub' on page 11
undefi
ned on input line 226.

```
Package natbib Warning: Citation `wolstencroft2015seek' on page 11
undefined on
 input line 226.



[11]

Package natbib Warning: Citation `keane_growing_2021' on page 12
undefined on i
nput line 228.


Underfull \hbox (badness 10000) in paragraph at lines 232--239

 []

LaTeX Font Info:    Font shape `TS1/Merriwthr-OsF/m/n' will be
(Font)              scaled to size 7.5pt on input line 243.

Underfull \vbox (badness 2012) has occurred while \output is active []




Underfull \hbox (badness 10000) in paragraph at lines 291--292
\T1/Merriwthr-OsF/m/up/7.5 (+20) All metabolomics and phe-no-typic data
used is
 com-pletely
 []

No file main.bbl.

Package natbib Warning: There were undefined citations.



[12]
enddocument/afterlastpage: lastpage setting LastPage.
(./main.aux)
 ***********
LaTeX2e <2024-06-01> patch level 2
L3 programming layer <2020/03/25>
 ***********

LaTeX Font Warning: Size substitutions with differences
(Font)              up to 1.0pt have occurred.


LaTeX Font Warning: Some font shapes were not available, defaults
substituted.

Package rerunfilecheck Info: File `main.out' has not changed.
```

```
(rerunfilecheck)             Checksum:
5C70612B4E933BFA8BEFBD0FECBBCCB4;4326.
 )
Here is how much of TeX's memory you used:
 24378 strings out of 473583
 476728 string characters out of 5732343
 1983908 words of memory out of 5000000
 46239 multiletter control sequences out of 15000+600000
 1860777 words of font info for 555 fonts, out of 8000000 for 9000
 1141 hyphenation exceptions out of 8191
 123i,13n,131p,3134b,1049s stack positions out of
10000i,1000n,20000p,200000b,200000s
<c:/texlive/2024/texmf-dist/fonts/type1/sorkin/merriweather/Merriwthr-
Bold.pf
b><c:/texlive/2024/texmf-dist/fonts/type1/sorkin/merriweather/Merriwthr-
BoldIta
lic.pfb><c:/texlive/2024/texmf-
dist/fonts/type1/sorkin/merriweather/Merriwthr-I
talic.pfb><c:/texlive/2024/texmf-
dist/fonts/type1/sorkin/merriweather/Merriwthr
-Regular.pfb><c:/texlive/2024/texmf-dist/fonts/type1/public/lm/lmtt8.pfb>
Output written on main.pdf (12 pages, 344598 bytes).
PDF statistics:
 263 PDF objects out of 1000 (max. 8388607)
 233 compressed objects within 3 object streams
 48 named destinations out of 1000 (max. 500000)
 201958 words of extra memory for PDF output out of 221844 (max.
10000000)
```

Manuscript_revised
Click here to access/download;Manuscript;MetaboSERV_Manuscript.pdf

Placeholder for OUP logo oup.pdf

GigaScience, 2024, 1–12

doi: xx.xxxx/xxxx
Manuscript in Preparation
Technical Note

TECHNICAL NOTE

MetaboSERV - a platform for selecting, exchanging, and visualizing metabolomics data with controlled data access

Tim Tucholski[1,*], Angela Maennel[2], Yacoub Abelard Njipouombe Nsangou[1,3], Sven Schuchardt[4], Matthias Gruber[5], Fabian Kellermeier[5], Katja Dettmer[5], Peter J. Oefner[5], Wolfram Gronwald[5], Michael Altenbuchinger[1], Jürgen Dönitz[1,3,6,†] and Helena U. Zacharias[2,*,†]

[1]Department of Medical Bioinformatics, University of Göttingen and [2]Peter L. Reichertz Institute for Medical Informatics of TU Braunschweig and Hannover Medical School, Hannover Medical School and [3]Institute of Computational Biology, Helmholtz Center Munich and [4]Department of Bio- and Environmental Analytics, Fraunhofer ITEM, Hannover and [5]Institute of Functional Genomics, University of Regensburg and [6]Campus Institute Data Science (CIDAS) Göttingen

[*]Correspondence: zacharias.helena@mh-hannover.de (Helena U. Zacharias); tim.tucholski@med.uni-goettingen.de (Tim Tucholski)
[†]These authors contributed equally to this work.

Abstract

Background
The growing number of metabolomics studies, based on high-dimensional data measured by hyphenated mass spectrometry (MS) and/or nuclear magnetic resonance (NMR) spectroscopy, has sparked the creation of several public metabolomics data repositories. Each repository emphasizes different aspects regarding data selection and representation, but most offer only limited options for privacy-preserving data sharing.
Results
We present MetaboSERV, an open-source, browser-based metabolomics platform dedicated to the selection, integration and sharing of quantitative metabolomics data and metadata with controlled data access. MetaboSERV aims to aid researchers in analyzing their results by facilitating means to browse, visualize and compare data across available data sets. It provides different access control functionalities, creating an environment in which data can be shared safely in a privacy-preserving manner to support collaborative and interdisciplinary research. Furthermore, it is designed to be extensible and adaptable to existing data management infrastructures through the creation of self-managed MetaboSERV instances, for which we provide the source code and a set of configurable Docker images.
Conclusions
The public MetaboSERV instance is available at `https://metaboserv.ckdn.app`, and the source code can be found at `https://gitlab.gwdg.de/MedBioinf/metabolomics/metaboserv`. The Research Resource Identifier (RRID) for MetaboSERV is *SCR_025496*.

Key words: (privacy-preserving) Data Sharing; Metabolomics; Nuclear Magnetic Resonance Spectroscopy; Mass Spectrometry; Collaborative Research

Background

Metabolomics is the comprehensive study and quantitative analysis of all metabolites that are detectable in a biological specimen. It has found a wide range of applications in the medical field, including the identification of biomarkers and elucidation of molecular pathomechanisms in precision medicine [1, 2, 3]. Nuclear magnetic resonance (NMR) spectroscopy and hyphenated mass spectrometry (MS) are the two most widely used analytical methods in metabolomics and are suitable for large-scale studies [4, 5]. In response to the fast increase in the number of metabolomics studies published and the corresponding generation of vast amounts of research data, different metabolomics data repositories such as MetaboLights [6], Metabolonote [7], Metabolomic Repository Bordeaux (MeRy-B) [8], the Metabolomics Workbench [9], and more recent platforms such as MetHoS [10] and COMETS Analytics [11] have been created. MetaboLights, Metabolomics Workbench, MeRy-B, and Metabolonote primarily focus on fully open public sharing of metabolomics data. MeRy-B solely accommodates NMR-based metabolomics plant data, and Metabolonote solely metabolomics metadata, respectively. COMETS Analytics and MetHoS enable comprehensive data analysis of stored experimental data, the former being specifically designed for meta-analyses and the latter with a particular focus on untargeted MS data. The repositories are constantly evolving to fit the needs of the research community [12], and enable researchers to make their experimental data findable, accessible, interoperable, and re-usable as defined by the FAIR principles for scientific data management and stewardship [13, 14].

Existing repositories, in particular MetaboLights [6] and Metabolomics Workbench [9], focus on fully open public sharing of experimental data and/or metadata upon publication of study results. They only provide limited options for controlled access sharing of metabolomics data within a specific group of researchers. However, the latter is the typical scenario in an interdisciplinary collaboration, where clinicians, metabolomics experimentalists, and (metabolomics) data scientists/bioinformaticians perform dedicated tasks in a joint metabolomics research project (Fig. 1). To foster collaborative and interdisciplinary research, all partners, irrespectively of their (potentially highly diverse) programming skills, should be able to browse and visualize the metabolomics data as well as generate summary statistics and carry out different data analysis tasks within a data privacy preserving environment. Especially biomedical metabolomics data from human studies require specific attention to data security. Just recently, a call for controlled access models for metabolomics data sharing repositories, as a potential requirement due to patient consent statements, personal data regulations such as the European Union General Data Protection Regulation (GDPR) or other relevant legislation, has been issued [15]. This call demonstrates the urgent need of providing metabolomics data repositories with controllable data access.

We present MetaboSERV, an open-source browser-based platform for controlled access sharing of NMR and MS metabolomics data, metadata, and research results. MetaboSERV offers rich and intuitive data selection, browsing and visualization functionalities and aims to facilitate controlled data accessibility within research collaborations, particularly prior to publication of research findings. The MetaboSERV platform can be employed through either the public MetaboSERV instance, available at https://metaboserv.ckdn.app, or fully autonomous, self-managed, local MetaboSERV instances that can be set up and operated by end-users utilizing our configurable Docker images and detailed user guides.

Methods

Implementation

Data Storage

Metabolite concentration data and associated metadata (such as phenotype data) are stored in two different databases, namely an Elasticsearch [16] instance running on version 8.3 and a MariaDB instance running on version 10.11, with a Python interface [17], respectively. Other experimental data (such as raw spectral data) that can be uploaded to MetaboSERV are stored on the server MetaboSERV is hosted on. MariaDB contains numerical data such as metabolite concentrations and reference values retrieved from the Human Metabolome Database (HMDB), version 5.0 [18]. Elasticsearch contains user account data, phenotype data and study metadata. The schemaless data storage provided by Elasticsearch enables MetaboSERV to be flexible with regards to metadata that the user uploads for a study. The Python Elasticsearch Client [19] acts as a wrapper around Elasticsearch, providing basic database querying functions that are then translated into Elasticsearch Query Domain Specific Language (DSL) queries.

User Interaction

The MetaboSERV web interface is tailored to facilitate seamless user interaction. It is based on the VueJS3 [20] framework and mainly implemented in TypeScript [21, 22]. In addition, it makes use of the client-side-store capabilities provided by Pinia [23] and the AG Data Grid [24] package for table creation.

Supplementary Web Services

MetaboSERV utilizes two web services to (1) offer an application programming interface (API) and (2) process raw metabolomics NMR data. The first web service is implemented in Python [25] and uses the package flask [26] to provide the API for supplying data to the web application. Non-native packages used for the service include numpy [27], pandas [28], matplotlib [29], seaborn [30], and xmltodict [31]. Flask-related add-ons include flask-cors [32], flask-swagger-ui [33], flask-jwt-extended [34], and werkzeug [35]. The package gunicorn [36] is used as a production WSGI server on top of flask. The package python-magic [37] helps with file validation. The second web service for processing raw metabolomics data, including raw NMR spectra, is *R*-based [38] and makes use of the packages restRserve [39] and mrbin [40]. For a complete list of used TypeScript, Python and *R* packages and their respective versions, please refer to Supplementary Table S1.

Data privacy and security

Following the principles of personal data minimization in the GDPR, only required personal data is collected and stored in MetaboSERV. New users need to provide an e-mail address upon registration, which is automatically validated and can be used to recover lost passwords, which are saved securely encrypted in the database (salted and hashed). Communication between all modules is handled using Hypertext Transfer Protocol (HTTP) or preferably HTTP-secure (HTTPS) requests and responses. For all non-internal communication, HTTPS is enforced. More details are provided in the Supplementary Section "User Authentication and Password Storage".

Metabolomics metadata

Metabolite metadata consisting of reference concentration ranges for healthy humans for the most common human biofluids (urine, plasma, serum, feces and cerebral spinal fluid) and synonym lists

Compiled on: March 18, 2025.
Draft manuscript prepared by the author.

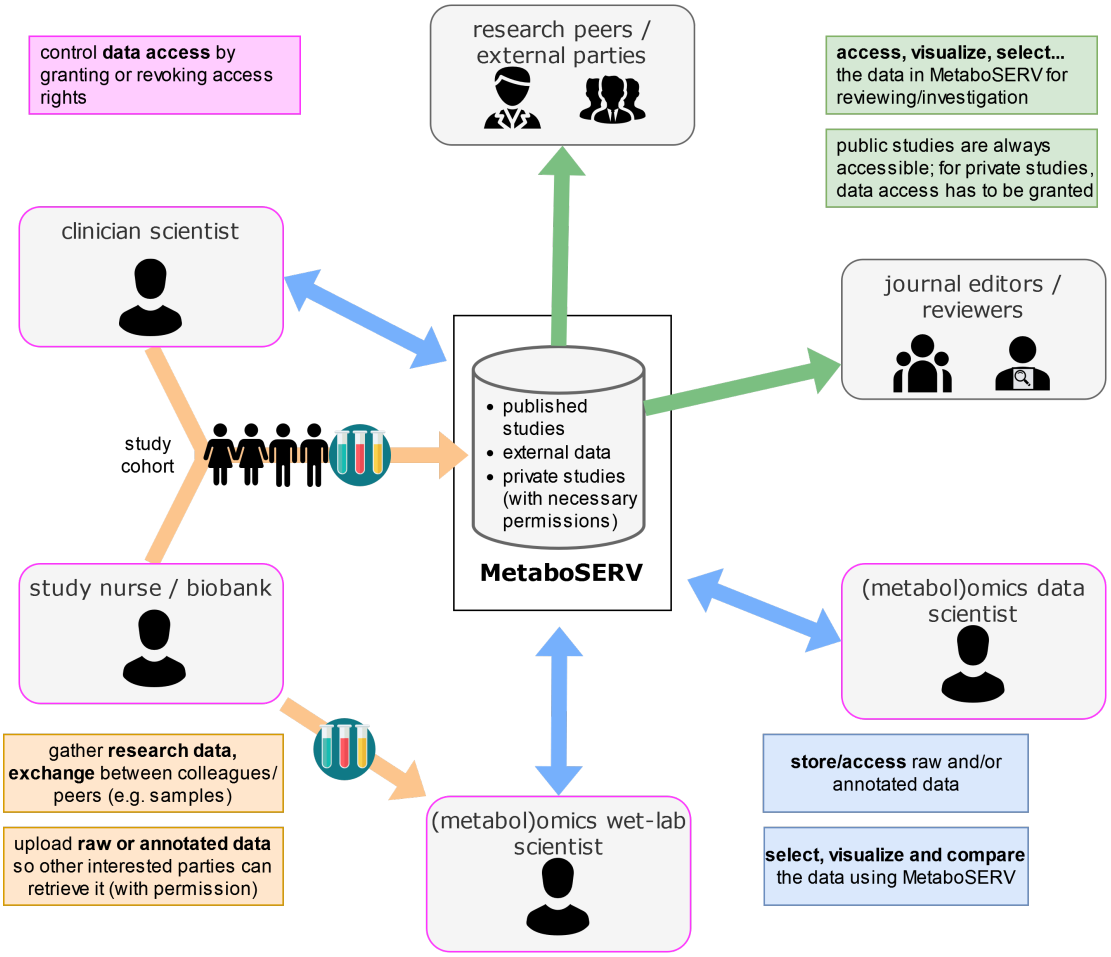

Figure 1. An exemplary schema of how the MetaboSERV platform can connect interdisciplinary collaborators of a research project as well as external parties: clinician scientists and study nurses gather phenotypic information and biofluid specimens from the study cohort, which are further measured by metabolomics wet-lab scientists. All three collaboration partners can store their collected raw and processed data on the MetaboSERV platform, by either employing the public MetaboSERV instance or a local MetaboSERV instance, autonomously set-up at one of the collaborating institutions. (Metabol)omics data scientists can access the data in MetaboSERV, analyse it and upload further results to MetaboSERV. Additional access can be granted to research peers that are interested in the data, as well as to journal editors and reviewers to facilitate peer-review.

were retrieved from the HMDB, version 5.0 [18].

Metabolomics use case data

NMR data from a previous study on 106 patients undergoing cardiac surgery [41] served as an exemplary dataset for MetaboSERV and was used to guide the implementation process. 34 of the 106 patients had been diagnosed with postoperative acute kidney injury (AKI) [41]. It includes 1D ^{1}H NMR spectra from urine specimens collected from all study participants 24 hrs after cardiac surgery with cardiopulmonary bypass (CPB) use. These spectra were acquired using a 600 MHz Bruker Avance III spectrometer (Bruker BioSpin GmbH, Ettlingen, Germany). Additionally, 1D ^{1}H NMR spectra from 85 plasma specimens of the same study participants were collected, measured, and absolutely quantified as described in [42].

A second use case data set includes absolute concentrations of 630 metabolites measured in 9 aliquots of the NIST frozen human plasma Standard Reference Material 1950 (SRM 1950). Data was acquired on an AB Sciex 6500+ triple quadrupole mass spectrometer (AB Sciex Germany GmbH, Darmstadt, Germany) coupled to an ExionLC 30AD (AB Sciex Germany GmbH, Darmstadt, Germany) ultra-high performance liquid chromatography (UHPLC) system employing the MxP Quant 500 kit (Biocrates life sciences, Innsbruck, Austria).

A third use case data set consists of 1,228 unique metabolites semi-quantitatively measured on the Metabolon H4 platform in 1,002 human blood plasma specimens [43]. Data was acquired on a Thermo Scientific Q-Exactive high resolution/accurate mass spectrometer interfaced with a heated electrospray ionization (HESI-II) source and utilizing a Waters ACQUITY ultra-performance liquid chromatography (UPLC) system. A methanol extraction was performed for protein precipitation and the resulting extract of each specimen was divided into five fractions: two fractions were used for analysis by two separate reversed phase (RP)/UPLC-MS/MS methods with positive ion mode electrospray ionization (ESI), one fraction was used for analysis by RP/UPLC-MS/MS with negative ion mode ESI, one fraction was used for analysis by HILIC/UPLC-MS/MS with negative ion mode ESI, and one aliquot was reserved for backup. Data on the original scale, i.e., values normalized in terms of raw area counts without missing value imputation as provided by Metabolon, were downloaded from the Metabolomics Workbench, https://www.metabolomicsworkbench.org, Project ID PR001762.

Results

MetaboSERV architecture

MetaboSERV is a web-based, open-source metabolomics platform, specifically designed for controlled user access and cross-comparison between studies. It includes four interconnected modules, the *MetaboSERV Web Interface*, the *Backend Service*, the *Databases*, and the *Raw Data Parser*, as presented in Fig. 2.

The *MetaboSERV Web Interface* serves as an interface to all functionalities and data contained in MetaboSERV, facilitating seamless interaction with the user. Raw experimental data, e.g., spectra derived from NMR or MS experiments, can also be stored in MetaboSERV. However, raw experimental data is saved as-is on the server without any additional processing. MetaboSERV further includes an *R*-based *Raw Data Parser*, which is capable of parsing, processing and visualizing NMR raw frequency domain data provided in the Bruker format. Finally, in order to encapsulate “create, read, update and delete (CRUD)” operations to the databases and to add logic and visualization options, MetaboSERV includes an extensible web service, referred to as the *Backend Service*, that mediates between the web interface and the other modules. The *Backend Service* also handles user authentication and authorization as well as file validation measures.

MetaboSERV server environment

The public MetaboSERV instance, available at https://metaboserv.ckdn.app, is hosted at the computing center of the Hannover Medical School, Germany (MHH). MHH’s regulations for access control to the server, security updates, backup and monitoring are implemented following the ISO 27001 and the standards of the German Federal Office for Information Security (Bundesamt für Sicherheit in der Informationstechnik, BSI). In particular, the public MetaboSERV server at MHH and the data stored there can only be accessed by authorized administrators. Server access is continuously logged and regularly inspected. The virtual machine is equipped with the latest security updates and regular backups are being taken every six hours.

Local MetaboSERV instances can be set up by cloning our repositories at https://gitlab.gwdg.de/MedBioinf/metabolomics/metaboserv and creating, configuring, and running the respective Docker images. Detailed user guides are provided in Supplementary File S6: “Detailed installation and user guide for the set-up of local MetaboSERV instances” as well as at https://metaboserv.ckdn.app/guide and in our GitLab repositories. MetaboSERV is generally resource-friendly, all core components can be set up on a dual-core machine with 6 GB of random access memory (RAM) and 200 GB of hard disk space for MariaDB and Elasticsearch. Furthermore, a sufficient amount of hard disk space is necessary to store raw metabolomics data. Query speed is heavily dependent on the resources attributed to the underlying Elasticsearch and MariaDB instances. Therefore, it is recommended to set up MetaboSERV on a machine with at least four cores and 16 GB of RAM and to take advantage of Elasticsearch’s *sharding* mechanism [44]. By default, MetaboSERV makes no assumptions about the Elasticsearch environment to avoid structural and capacity-related issues. These self-managed MetaboSERV instances run isolated from the public MetaboSERV instance and allow hosting and managing data on self-governed servers, removing any further data privacy concerns. They can also be altered and configured to fit different research environments and data formats, and assure a degree of system portability due to the nature of container virtualization. More information on user-specific configuration settings are provided in Supplementary File S6.

Data upload and processing

In the MetaboSERV platform, a *study* encapsulates the uploaded experimental data, annotations (such as phenotypes) and metadata, e.g., the study owner and collaborators. The study creation process is represented in Fig. 3A. The user can either upload raw experimental data, absolutely quantified metabolite concentration data or both, and the study must contain mandatory metadata (study authors, at least one biospecimen, at least one analytical method and a year or range of years associated with the study). Raw experimental data of any format, bundled with a common tool like gzip or zip, can be uploaded to and retrieved from MetaboSERV. Concentration data represented as any of the common file formats TSV, CSV (e.g., bucket tables), XLS or XLSX is accepted. It is also possible to add an additional file with phenotype data. The concentration and phenotype data files, however, have to adhere to the layout specified by MetaboSERV, as outlined in Supplementary File S3. A validation procedure, which verifies that data fit the requirements by checking the file structure, is performed instantaneously before the data are further processed and added to MetaboSERV. Finally, additional arbitrary metadata can be added by uploading a JSON/YAML metadata file, as detailed in Supplementary File S3. It is also possible to specify metadata directly, which will overwrite any uploaded metadata with conflicting entries.

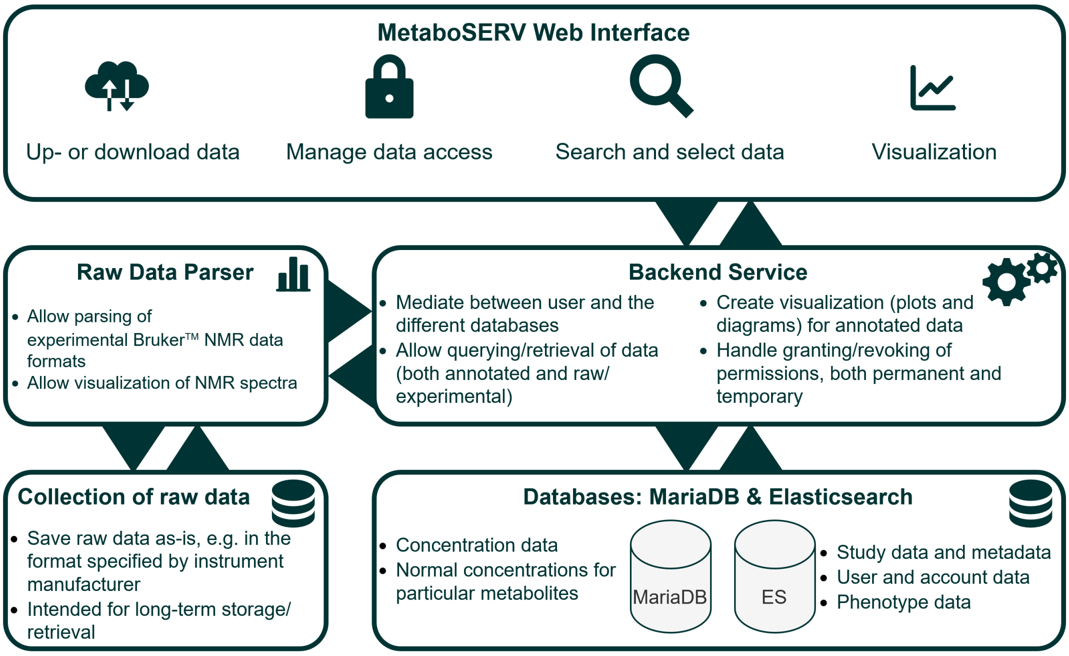

Figure 2. The four modules of MetaboSERV. Users interact solely with the *MetaboSERV Web Interface*, while the *Backend Service* mediates between the user and the *Databases* as well as the *Raw Data Parser* for parsing Bruker NMR spectra.

Data access control and management

The MetaboSERV platform is built around the notion of collaborative research work [45] and aims to simplify the exchange of experimental data and metadata, irrespective of whether the data are meant to be published or to remain private. Each study can be managed separately, as shown in Fig. 3B. In addition to showcasing phenotypes and any study metadata, it allows to modify the metadata.

In order to guarantee data privacy, two different access control mechanisms are implemented for both public and any local MetaboSERV instances. They can be used independently of or in combination with each other. Each MetaboSERV user, who wants to upload data to the MetaboSERV platform, first needs to create an individual user account. Users are required to provide a username, e-mail address, and password, which is stored encrypted (salted and hashed, please refer to Supplementary Section S2 "User Authentication and Password Storage" for more details), to register, and neither their account names nor their personal data are revealed to other users. Upon registration, an e-mail containing a verification code is sent to the e-mail address that was used to register the account. This unique code, which consists of ten random characters, must be entered once after logging in to unlock any permissions associated with the account, which includes viewing studies shared with the account or uploading studies. The user accounts are associated with access rights for specific studies. For each uploaded data set, two different levels of access rights can be granted to other user accounts - either "read-only permissions" or "full data editing and management authority" - by the original uploader or users with "full data editing and management authority" accounts. The account responsible for the initial creation of the study possesses "full data editing and management authority" at any time and can never be removed as a contributor by any other user. Authentication during the log-in process is handled through JSON web tokens [46] (JWTs), which are associated with each user and newly generated on each log-in. Unauthorized access attempts are continuously logged in the Elasticsearch database. The number of unauthorized access attempts, which occurred since the last authorized log-in, is reported to the user upon every log-in. In the public MetaboSERV instance, an alert e-mail is sent to the user's e-mail address linked to the account in case the number of unauthorized access attempts exceeds five attempts, and the account is locked for ten minutes after a total of ten tracked unauthorized access attempts. On a successful log-in attempt, this counter is reset to zero. More information and customization details for local MetaboSERV instances are provided in Supplementary File S2.

Additionally, all users with "full data editing and management authority" have the ability to create authorization tokens. These, in contrast to JWTs, are independent of user accounts. Each token can be associated with an expiration date, a set of permissions for one or more data sets, and a comment. Logging in with an authorization token grants the set of permissions specified by the token creator without having to create a user account, as long as the token is valid and not yet expired. This feature is intended to enable short-term sharing of data, such as providing project results to a peer or journal editor/reviewer for validation, by minimizing the effort required to access the data: Simply sharing the token will allow the recipient to view - or even edit, if allowed - the selected data.

Data search and selection

The core feature of the MetaboSERV platform is a many-faceted, intuitive data selection system that allows for simple and complex queries alike. By configuring search parameters in the web interface, users can specify exactly the data or studies suitable for their research task or use case, thereby filtering out superfluous data. Data can be selected according to studies, metabolites, as well as phenotypes (Fig. 4), and subsequently either be analyzed further in MetaboSERV or downloaded for other purposes. This also facilitates straightforward integration of different studies or research projects, as data from several data sets can be combined arbitrarily, as long as the user has acquired permission to view the respective data.

Data selection revolves around finding data that fit the physiological and phenotypical criteria outlined by the user. In a first step, users can select all studies they want to include in their query (Fig. 4A). Any number of studies can be combined. Next, metabolites can be specified, *e.g.*, to represent the target metabolic profile (Fig. 4B). By default, all metabolite concentration levels are retrieved from the selected studies. It is also possible to exclude a subset of metabolites (Fig. 4B). In an optional next step, users can

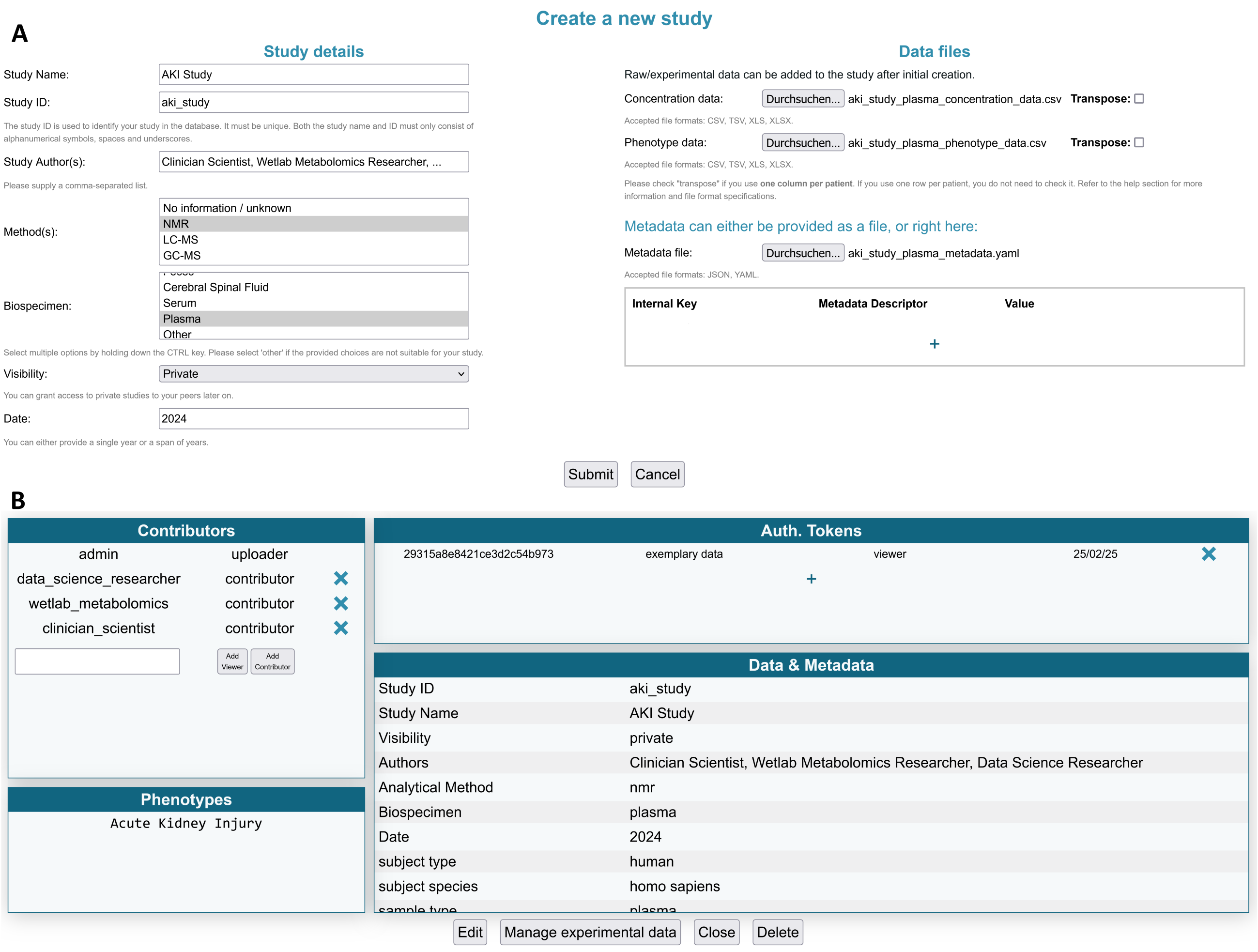

Figure 3. **A** MetaboSERV platform data upload menu. Several files and metadata attributes can be provided for a study. Concentration and phenotype data can be added (concentration data is required). Metadata can be provided by means of a JSON/YAML file or directly in the application. **B** MetaboSERV study management menu. Access can be granted to other users, either "read-only" access or (limited) "write" access (top left). Access can be revoked, though the study uploader privileges can neither be removed nor altered at any time. Available phenotypic information of the stored study is listed under "Phenotypes" (bottom left). Authorization tokens for a particular study can be added/removed, if desired with specific expiration dates, comments and additional studies that are covered by the token (top right). A summary of study data and metadata is provided under "Metadata" (bottom right). By clicking on "Edit", the user can edit the study, while clicking on "Manage experimental data" allows the user to up- or download experimental data.

add constraints by specifying individual concentration ranges for the inclusion or exclusion of metabolite levels (Fig. 4C). Additionally, it is possible to supply a range only for visualization purposes (without affecting the query results) by choosing the filter type "vis". The corresponding metabolite concentration ranges can either be entered by the user, or they can be selected from a pre-defined collection of different reference concentrations from the HMDB [18].

In a final step, users can choose to include phenotype data (or only particular levels of a phenotype, such as "healthy"), if such data are available for any of the selected data sets (Fig. 4D). Phenotype data does not directly affect the data selection process, but can be used for subsequent data analysis and visualization purposes. Query results can be displayed and browsed in a table (see Fig. 6) or visual representation (as shown in Fig. 5). It is also possible to inspect the underlying experimental data, given that it matches a supported format. Currently, raw NMR free induction decays (FIDs) as well as spectra in the frequency domain in the Bruker file format are supported.

Data sets uploaded to the public MetaboSERV instance, as well as combinations or subsets thereof, can be browsed and visualized in the public MetaboSERV web interface, provided the user may access the respective data. Local MetaboSERV instances are initially set up without any public use case data sets, which are, however, available for download at `https://metaboserv.ckdn.app`. Histograms, scatterplots and heatmaps depicting the Pearson correlation between different metabolites (an example is provided in Fig. S2) are created automatically. Different subgroups, such as those defined by phenotypes, are also taken into account (see Fig. 5). They can be configured and created on demand for different combinations of nominal phenotypes or metabolites. Concentration values can be displayed in a table, which highlights whether each value is contained in a selected range or not and additionally displays selected phenotype data for each entry (Fig. 6). NMR raw frequency data in the Bruker file format can also be displayed. Finally, MetaboSERV facilitates the automatic creation of quality control plots (see example provided in Supplementary File S4: Use case 2 and Figure S3), allowing researchers to quickly gauge the amount of missing values, *i.e.* concentration values below the limit of detection (LOD). Here, the user can create further plots showing the distribution of missing values per metabolite across all measured samples, and toggle between showing values over or under the LOD for both types of plots.

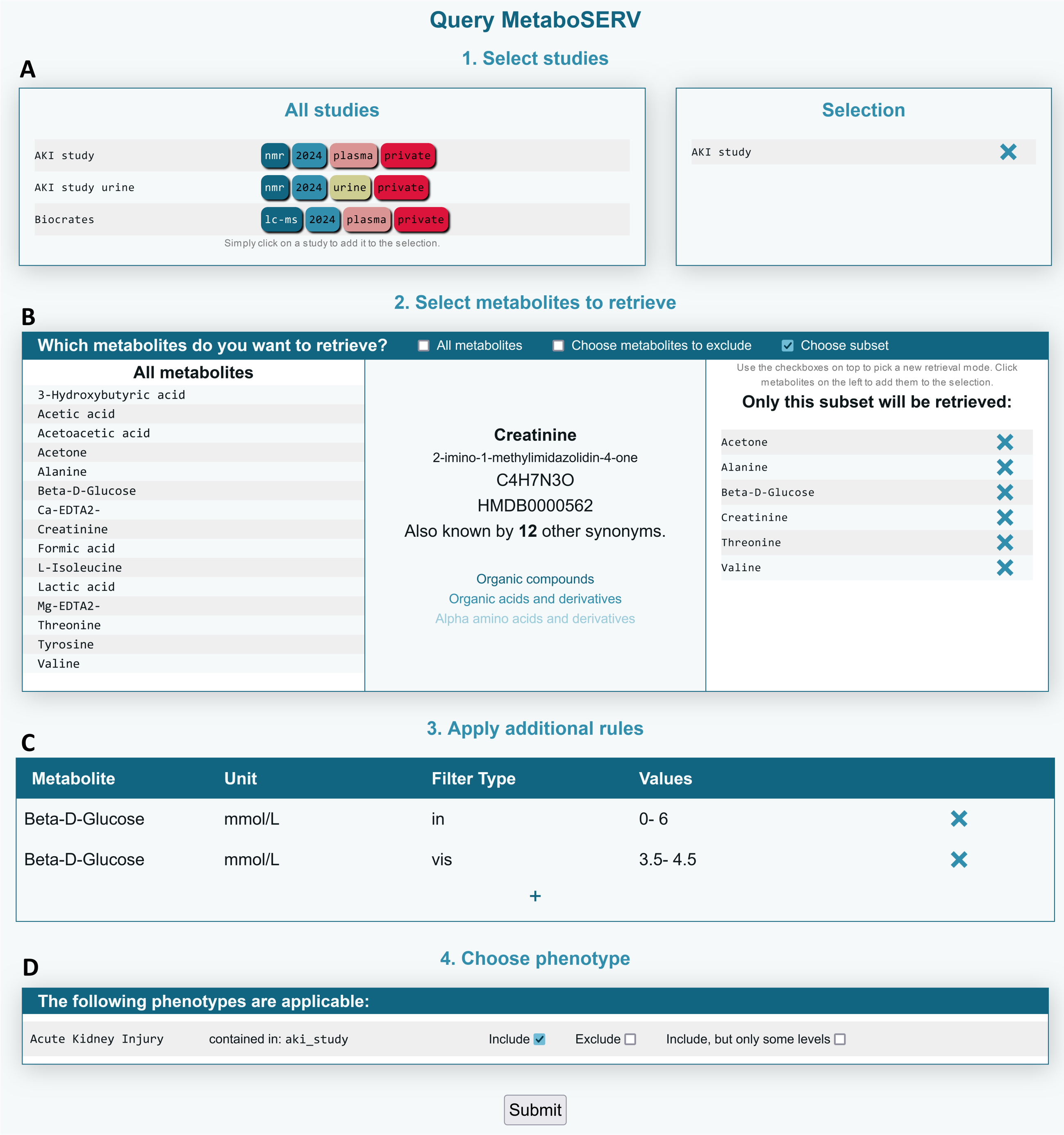

Figure 4. An example query to the public MetaboSERV database. **A** Study selection menu. All accessible studies are shown on the left and can be selected for a query. In this case, only the *AKI study* was selected, consisting of absolutely quantified concentration values of plasma metabolites measured by NMR spectroscopy. **B** Metabolite selection menu. By default, all metabolites are retrieved. If desired, only a particular subset will be retrieved or excluded, respectively. Metabolite information, such as for creatinine, is shown if the user hovers over a metabolite entry. **C** Metabolite filter menu. Specific filtering rules, such as specifying that the concentration level of a particular metabolite has to be included in or excluded from a certain range, can optionally be added here. Metabolite concentration ranges for healthy individuals provided by the HMDB may be used as filter options, too. Alternatively, only a selected concentration range can be visualized without altering the set of results. In this example, all entries with beta-D-glucose levels above 6 mmol/L are ignored, while entries with a beta-D-glucose level between 3.5 and 4.5 mmol/L will be highlighted. **D** Phenotype selection menu. By default, phenotype data is retrieved completely. A subset of phenotypes or phenotype levels can also be selected.

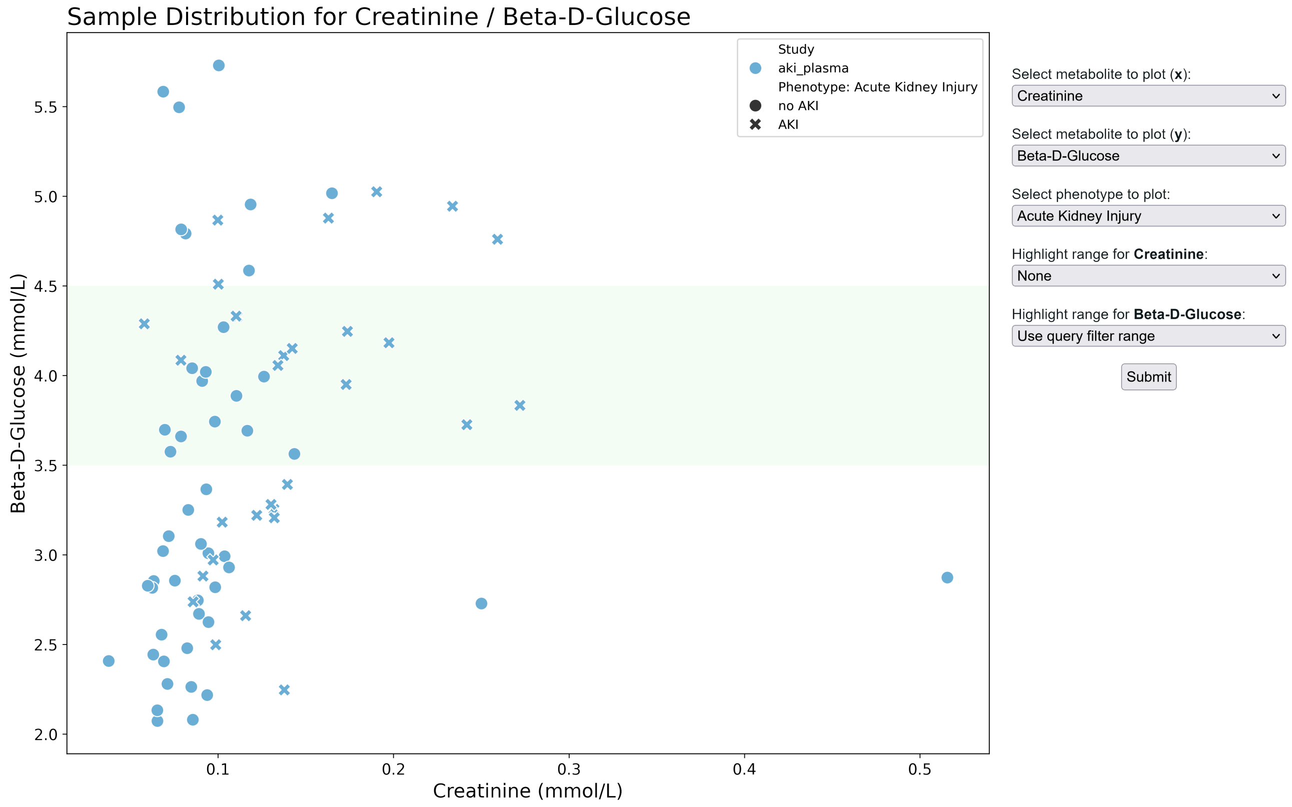

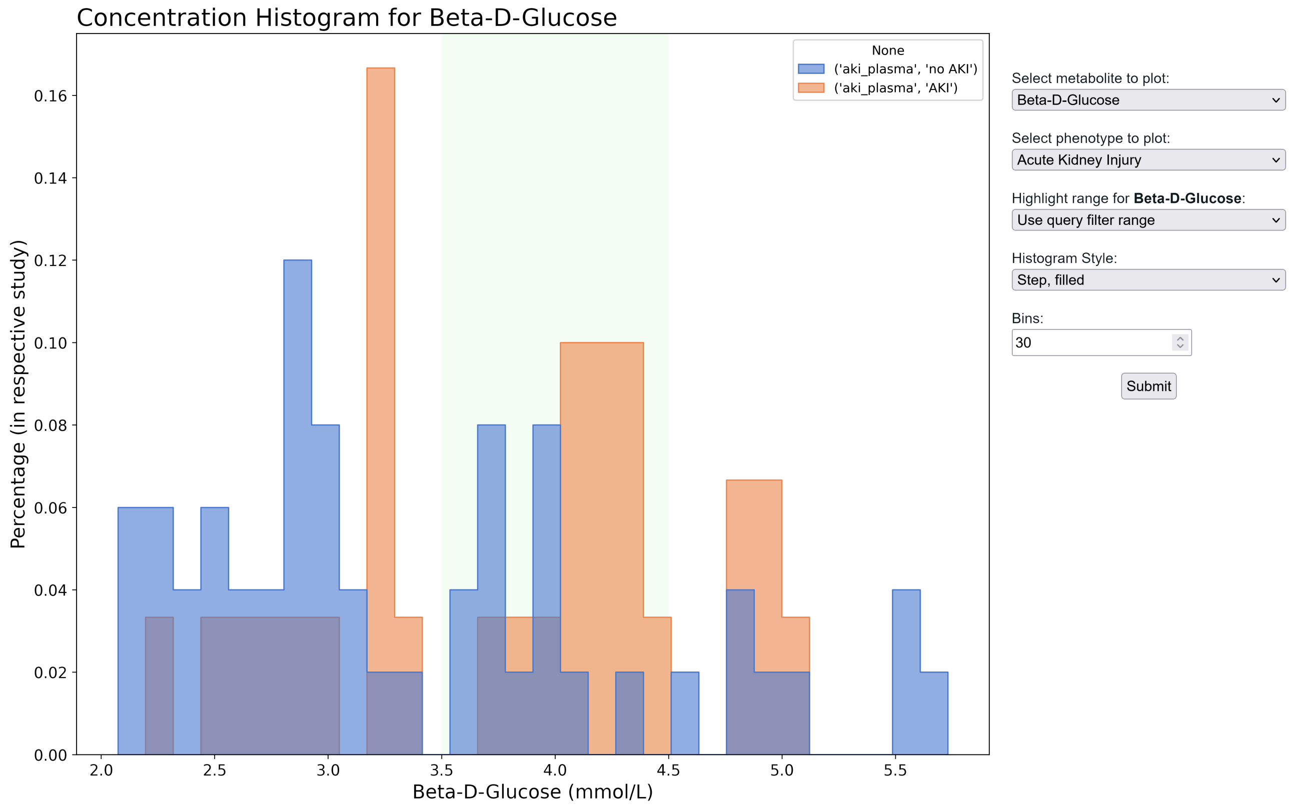

Figure 5. Results of the query pictured in Fig. 4, shown in the form of a sample distribution scatterplot and a concentration histogram, respectively. The metabolites and phenotypes to be plotted can be selected by the user. Specimens with beta-D-glucose levels between 3.5 and 4.5 mmol/L are highlighted, as specified in the query in Fig. 4C and by using the "Use query filter range" option in the "Highlight range" field as seen in the menu on the right.

Source ID	Study	Acute Kidney Injury	Acetone	Alanine	Beta-D-Glucose ↓	Creatinine	Threonine
AKI_51	aki_study	no AKI	1.91072 mmol/L	0.103263 mmol/L	5.73048 mmol/L	0.100439 mmol/L	
AKI_19	aki_study	no AKI	0.357399 mmol/L	0.16642 mmol/L	5.58404 mmol/L	0.0687475 mmol/L	
AKI_20	aki_study	no AKI	0.144835 mmol/L	0.153249 mmol/L	5.49683 mmol/L	0.0778023 mmol/L	
AKI_18	aki_study	AKI	0.0228864 mmol/L	0.417078 mmol/L	5.02584 mmol/L	0.190397 mmol/L	0.116662 mmol/L
AKI_55	aki_study	no AKI	0.0268556 mmol/L	0.241009 mmol/L	5.01733 mmol/L	0.164905 mmol/L	0.0530378 mmol/L
AKI_95	aki_study	no AKI	0.316609 mmol/L	0.193356 mmol/L	4.95481 mmol/L	0.118549 mmol/L	0.0914211 mmol/L
AKI_06	aki_study	AKI	0.0806513 mmol/L	0.24828 mmol/L	4.94507 mmol/L	0.233604 mmol/L	
AKI_94	aki_study	AKI	0.0834382 mmol/L	0.254088 mmol/L	4.87885 mmol/L	0.162888 mmol/L	0.0780183 mmol/L
AKI_46	aki_study	AKI	0.118823 mmol/L	0.169484 mmol/L	4.8676 mmol/L	0.0998487 mmol/L	0.0759844 mmol/L
AKI_59	aki_study	no AKI	0.0574271 mmol/L	0.217274 mmol/L	4.81593 mmol/L	0.0789833 mmol/L	0.10618 mmol/L
AKI_104	aki_study	no AKI	0.0211974 mmol/L	0.363983 mmol/L	4.79278 mmol/L	0.0814931 mmol/L	0.0971578 mmol/L

1 to 11 of 80 | Page 1 of 8

Figure 6. Results of the query pictured in Fig. 4, shown as a table which is sorted according to beta-D-glucose levels in descending order. Concentration values that passed the filtering rules set by the user are highlighted in green. As the filtering rules removed patients with beta-D-glucose levels above 6 mmol/L, the entire beta-D-glucose column is highlighted in green. Phenotypes, such as *Acute Kidney Injury* and the according levels are also shown. Missing values are represented as empty cells.

Data retrieval

Research data, which includes both public data and private data given the required permissions, can also be retrieved from the MetaboSERV platform. This applies to retrieving any raw data as well as generating new documents containing quantified metabolite concentration data for any study or subsets thereof. Currently, JSON, CSV and XLSX formats are supported. Created plots are also available for download in PNG format.

Use case

We demonstrate the capabilities of the MetaboSERV platform in an exemplary use case:

A consortium of clinician scientists, wet-lab metabolomics and data science researchers carries out a metabolomics study on patients undergoing cardiac surgery. The clinician scientists have sent blood plasma specimens to the metabolomics wet lab for measurement by NMR spectroscopy. Furthermore, the clinician scientists have created a new study on the public MetaboSERV instance, named it "AKI study" (Fig. 3A), and uploaded the corresponding phenotype data and study metadata. They further add the wet-lab metabolomics and data science researchers as "contributors" to the study (Fig. 3B). The former upload the measured experimental data, in this case, both raw and absolute concentration data to this MetaboSERV study. The goal of this metabolomics study is to identify possible associations between post-operative acute kidney injury (AKI) and particularly creatinine as well as beta-D-glucose, but also additional metabolites. The statistical analyses are carried out by the data science researchers, who select the "AKI study" as well as the metabolites creatinine and beta-D-glucose, as well as acetone, alanine, threonine and valine in the MetaboSERV query interface (Fig. 4). As the researchers are not interested in effects of extremely high beta-D-glucose levels, they filter out any plasma specimen with a beta-D-glucose level above 6 mmol/L. They also highlight plasma specimens with beta-D-glucose levels between 3.5 and 4.5 mmol/L using a filter of type "vis" (Fig. 4C). Finally, the phenotype "Acute Kidney Injury" is selected. To graphically explore the hypothesized association between AKI and beta-D-glucose, the researchers generate two different visualizations: A histogram of the beta-D-glucose distribution stratified according to AKI diagnosis, and a scatterplot depicting the relation between beta-D-glucose and creatinine, as elevated blood creatinine levels are a strong indicator for impaired renal function and thus a marker for AKI [47] (Fig. 5). A clear association between higher beta-D-glucose levels and AKI diagnosis can be seen in both visualizations. Finally, the wet-lab metabolomics researchers want to show the metabolite concentration data to external collaboration partners. As they only want to provide them with a temporary data access for seven days without any editing permissions, they generate the corresponding authentification token "exemplary data" (see Fig. 3B) and share it with their collaboration partners. Two additional exemplary use cases, the first demonstrating a quality assessment of mass spectrometry data using MetaboSERV and the second illustrating MetaboSERV's capabilities of handling a large-scale, multi-modal, untargeted mass spectrometry data set, are provided in Supplementary Sections "3.4 File S4: Use case 2: Quality assessment of mass spectrometry data" and "3.5 File S5: Use case 3: Large-scale, multi-modal, untargeted mass spectrometry data".

Discussion

MetaboSERV is a browser-based, extensible metabolomics platform with a focus on (absolutely) quantified metabolomics research data from NMR and LC-MS measurements. A major goal of MetaboSERV is to provide researchers with the means to autonomously control access to their metabolomics data. The MetaboSERV platform differentiates between public and private data and offers two distinct and independent ways to grant access to private data sets, namely account- and token-based authentication. Data access can be limited to a customized time frame or revoked at any time by the owner, i.e., the user who originally uploaded the data set. Thus, MetaboSERV provides the research community with a privacy-preserving platform for exchanging metabolomics data and research findings. It is particularly designed for interdisciplinary collaborations between metabolomics experts, biological or medical scientists, and data analysts in different research settings, such as contract work of a metabolomics core facility, large third-party funded collaboration projects, *e.g.*, within (transregional) collaborative research centers, or institutional, national, and international research collaborations. MetaboSERV supports common data and metadata exchange formats (Bruker, CSV/TSV, XLSX and YAML, respectively) and is flexible with regards to data formatting, particularly for metadata and phenotypical data. The platform is completely open-source and also offers configurable and portable Docker containers for the purpose of hosting self-managed and self-maintained local MetaboSERV instances in addition to the

centralized, public MetaboSERV instance, hosted at MHH. These characteristics allow a seamless integration of MetaboSERV into local (biomedical) data exchange infrastructures. The MetaboSERV platform is efficient in terms of speed and flexible in terms of memory usage thanks to Elasticsearch and MariaDB. It is also extensible to suit the needs of different research institutions: both the functionality and the accepted data formats are designed to be adaptable, and MetaboSERV does not require a specific environment apart from a system containing Docker.

A second major feature of the MetaboSERV platform is to allow users to store and find data suitable for their research projects. In the public MetaboSERV instance, public datasets, such as the AKI study or reference concentration values for metabolites, can be retrieved or combined with available data, enabling complex queries. The MetaboSERV platform also provides visualization options for both raw NMR spectra and summary statistics of absolute metabolite concentrations. While MetaboSERV includes reference concentration ranges for a large number of metabolites from different biofluids provided by the HMDB, arbitrary concentration ranges can likewise be selected. Metadata and phenotypical data for studies and research projects can be kept alongside concentration data and the latter is also available for database queries.

MetaboSERV fills an important gap in the already established metabolomics data repository landscape: it enables interdisciplinary research collaborations to share their metabolomics experimental, phenotypic, as well as metadata within a user friendly platform with fully controlled data access and advanced data browsing and visualization options prior to publication of study results. It provides a user-controlled permission system for uploaded data, which can selectively allow access (both read-only and editorial) to particular data sets for different institutions, researchers and affiliated peers. Self-managed, local MetaboSERV instances can be created by any user employing the fully configurable Docker images, guaranteeing full autonomy as well as data privacy. These features set MetaboSERV apart from the largest and most widely used metabolomics data repositories, MetaboLights, the Metabolomics Workbench, Metabolonote, and MeRy-B, which are designed to share experimental metabolomics data and/or metadata with completely open access [6, 9, 7, 8]. Similar to MetaboLights and the Metabolomics Workbench, MetaboSERV can accommodate experimental data regardless of species, sample or analytical method, as well as metabolomics metadata. In contrast, Metabolonote is designed to exclusively hold metabolomics metadata [7], and MeRy-B focusses solely on NMR-based metabolomics plant data [8].

Another difference between MetaboSERV and the metabolomics data repositories discussed above is the rich data selection, visualization and querying functionality for absolutely quantified data. This selection process allows the combination of different studies and the integration of already published studies into new research projects. COMETS Analytics offers similar selection features and advanced data analysis tools across different studies, but it is designed specifically for standardized meta-analyses of multiple metabolomics studies rather than serving as a metabolomics study repository [11]. Likewise, MetHos focusses on large-scale processing, storage, and analysis of mass spectrometry data, without elaborate, privacy-preserving data sharing options as provided by MetaboSERV [10]. Furthermore, MetaboSERV allows the recording of methodological metadata without any format and/or content restrictions. To facilitate a low-threshold user experience of MetaboSERV, we implemented a very flexible metadata upload by deliberately not enforcing mandatory metadata specifications. In contrast, established metabolomics data repositories such as MetaboLights and the Metabolomics Workbench face the users with rather strict mandatory metadata as well as experimental data upload requirements.

Besides metabolomics-focussed data repositories, a large number of workspaces for data sharing and collaboration with options for privacy preservation have been released in the last decades, including commercial applications like Google Workspace and Nextcloud, as well as a multitude of freely available solutions including Figshare or Synapse/NF Data Portal [48]. Additionally, academic institutions worldwide start building up their own data sharing repositories, e.g., the Academic Cloud service for Lower Saxony, or RepoMed, the institutional repository of Hannover Medical School. However, none of these workspaces and solutions are designed to the specific needs of metabolomics data repositories, but rather provide "data-type agnostic" data lakes for the storage and retrieval of individual data sets [48]. In comparison to MetaboSERV, they do not provide smart search functions for metabolites across several, independent studies, no data analysis or visualization options, and, more importantly, do not support the set-up of self-administered, configurable instances, which are completely independent of the providers. The freely available software FAIRDOM-SEEK [49, 50] can be, similar to MetaboSERV, also deployed locally, however, it is designed particularly for data spanning multiple omics types or interconnecting datasets and systems biology models [49], and not for metabolomics data. Thus, uploaded data sets cannot be systematically queried or analysed with respect to individual metabolites and/or across studies.

The demands for privacy-preserving data sharing will further increase with technical advances in metabolic fingerprinting of human individuals on the one hand, and ongoing, large-scale rollout of artificial intelligence (AI) for metabolomics data analysis on the other hand. Analytical sensitivity and specificity of metabolic fingerprints will further increase due to technical progress, and will potentially facilitate patient re-identification [15]. AI, in particular, demands large, highly standardized (metabolomics) data sets to ensure maximum performance. MetaboSERV can build the basis for data scientists to access and select multiple metabolomics data sets measured at different metabolomics wet-labs for subsequent AI-based analysis within a privacy-preserving environment. Its open-source architecture allows full user control, adaptability and moreover, seamless integration into already existing research data infrastructures and AI analysis platforms.

Availability of source code and requirements

Project name: MetaboSERV
Project homepage:
https://gitlab.gwdg.de/MedBioinf/metabolomics/metaboserv
Operating system(s): Platform independent
Programming language: Python, R, TypeScript
Other requirements: Docker, Elasticsearch, MariaDB
License: MIT License
RRID: SCR_025496

Minimum installation requirements (recommended):

- 2 cores
- 100GB of hard-drive space (SSD recommended)
- 6GB of RAM

Additional Files

Supplementary Fig. S1. Data model (database schema) of the MetaboSERV Elasticsearch and MariaDB databases.
Supplementary Fig. S2. Exemplary heatmap showing pairwise Pearson's correlation coefficients between different metabolites in the AKI plasma study.
Supplementary Fig. S3. Exemplary quality control plot for the Biocrates test data discussed in Use Case 2.
Supplementary Fig. S4. Exemplary query to the public MetaboSERV database discussed in Use Case 3.
Supplementary Fig. S5. Part of the query results of Use Case 3.

Supplementary Fig. S6. Scatter plots of Use Case 3.
Supplementary Table S1. List of all programming languages and packages used to implement MetaboSERV, as well as their respective versions.
Supplementary File S1. Detailed description of MetaboSERV data model.
Supplementary File S2. Details on user authentication and password storage.
Supplementary File S3. MetaboSERV file specifications.
Supplementary File S4. Use case 2: Quality assessment of mass spectrometry data.
Supplementary File S5. Use case 3: Large-scale, multi-modal, untargeted mass spectrometry data.
Supplementary File S6. Detailed installation and user guide for the set-up of local MetaboSERV instances.

Declarations

List of abbreviations

AI - artificial intelligence
AKI - acute kidney injury
API - application programming interface
CPB - cardiopulmonary bypass
CRUD - create, read, update, delete
DSL - domain-specific language
FAIR - findable, accessible, interoperable, reusable
FID - free induction decay
GDPR - General Data Protection Regulation
HMDB - Human Metabolome Database
JWT - JSON web token
LC-MS - liquid chromatography – mass spectrometry
LOD - limit of detection
MeRy-B - Metabolomics Repository Bordeaux
MHH - Hannover Medical School
NMR - nuclear magnetic resonance
RRID - Research Resource Identifier

Ethical Approval

NMR data of the AKI use case had been previously collected with written informed patient consent upon ethical approval from the University Clinic Erlangen.

Consent for publication

All metabolomics and phenotypic data used is completely anonymized. No further consent is required.

Competing Interests

The authors declare that they have no competing interests.

Funding

This work was supported by the German Federal Ministry of Education and Research (BMBF) within the framework of the e:Med research and funding concept (grant numbers: 01ZX1912A, 01ZX1912C, and 01ZX1912D).

Author's Contributions

TT Software, Writing - Original Draft Preparation, Methodology, Visualization; AM Software, Conceptualization, Validation; YNN Validation; SS Conceptualization; MG Resources; FK Resources; KD Resources; PJO Conceptualization and Resources; WG Conceptualization and Resources; MA Conceptualization and Supervision; JD Conceptualization, Methodology, and Supervision; HZ Conceptualization, Writing - Original Draft Preparation, Methodology, Funding Acquisition, Resources, Formal Analysis; all Writing - Review & Editing of the manuscript.

Acknowledgments

The authors are grateful to Mr. Norman Schönfeld (MHH Information Technology, MHH) and Mr. Merlin-Puck Rietschel (Peter L. Reichertz Institute for Medical Informatics, MHH) for IT advise and support.

References

1. Clish CB. Metabolomics: an emerging but powerful tool for precision medicine. Cold Spring Harbor Molecular Case Studies 2015 Oct;1(1):a000588. https://www.ncbi.nlm.nih.gov/pmc/articles/PMC4850886/.
2. Emwas AHM, Salek RM, Griffin JL, Merzaban J. NMR-based metabolomics in human disease diagnosis: applications, limitations, and recommendations. Metabolomics 2013 Oct;9(5):1048–1072. https://doi.org/10.1007/s11306-013-0524-y.
3. Zacharias HU, Altenbuchinger M, Gronwald W. Statistical Analysis of NMR Metabolic Fingerprints: Established Methods and Recent Advances. Metabolites 2018 Sep;8(3):47. https://www.mdpi.com/2218-1989/8/3/47, number: 3 Publisher: Multidisciplinary Digital Publishing Institute.
4. Emwas AH, Roy R, McKay RT, Tenori L, Saccenti E, Gowda GAN, et al. NMR Spectroscopy for Metabolomics Research. Metabolites 2019 Jul;9(7):123. https://www.mdpi.com/2218-1989/9/7/123, number: 7 Publisher: Multidisciplinary Digital Publishing Institute.
5. Zacharias HU, Kaleta C, Cossais F, Schaeffer E, Berndt H, Best L, et al. Microbiome and Metabolome Insights into the Role of the Gastrointestinal–Brain Axis in Parkinson's and Alzheimer's Disease: Unveiling Potential Therapeutic Targets. Metabolites 2022 Dec;12(12):1222. https://www.mdpi.com/2218-1989/12/12/1222, number: 12 Publisher: Multidisciplinary Digital Publishing Institute.
6. Haug K, Salek RM, Conesa P, Hastings J, de Matos P, Rijnbeek ML, et al. MetaboLights—an open-access general-purpose repository for metabolomics studies and associated meta-data. Nucleic Acids Research 2013 Jan;41(Database issue:D781–D786. https://www.scinapse.io/papers/2069928158.
7. Ara T, Enomoto M, Arita M, Ikeda C, Kera K, Yamada M, et al. Metabolonote: A Wiki-Based Database for Managing Hierarchical Metadata of Metabolome Analyses. Frontiers in Bioengineering and Biotechnology 2015 Apr;3:38. https://www.ncbi.nlm.nih.gov/pmc/articles/PMC4388006/.
8. Ferry-Dumazet H, Gil L, Deborde C, Moing A, Bernillon S, Rolin D, et al. MeRy-B: a web knowledgebase for the storage, visualization, analysis and annotation of plant NMR metabolomic profiles. BMC Plant Biology 2011 Jun;11:104. https://www.ncbi.nlm.nih.gov/pmc/articles/PMC3141636/.
9. Sud M, Fahy E, Cotter D, Azam K, Vadivelu I, Burant C, et al. Metabolomics Workbench: An international repository for metabolomics data and metadata, metabolite standards, protocols, tutorials and training, and analysis tools. Nucleic Acids Research 2016 Jan;44(D1):D463–D470. https://doi.org/10.1093/nar/gkv1042.
10. Tzanakis K, Nattkemper TW, Niehaus K, Albaum SP. MetHoS: a platform for large-scale processing, storage and analysis of

metabolomics data. BMC Bioinformatics 2022 Jul;23(1):267. https://doi.org/10.1186/s12859-022-04793-w.

11. Temprosa M, Moore SC, Zanetti KA, Appel N, Ruggieri D, Mazzilli KM, et al. COMETS Analytics: An Online Tool for Analyzing and Meta-Analyzing Metabolomics Data in Large Research Consortia. American Journal of Epidemiology 2022 Jan;191(1):147–158. https://research.wur.nl/en/publications/comets-analytics-an-online-tool-for-analyzing-and-meta-analyzing, publisher: Oxford University Press.
12. Haug K, Cochrane K, Nainala VC, Williams M, Chang J, Jayaseelan KV, et al. MetaboLights: a resource evolving in response to the needs of its scientific community. Nucleic Acids Research 2020 Jan;48(D1):D440–D444. https://doi.org/10.1093/nar/gkz1019.
13. Wilkinson MD, Dumontier M, Aalbersberg IJ, Appleton G, Axton M, Baak A, et al. The FAIR Guiding Principles for scientific data management and stewardship. Scientific Data 2016 Mar;3(1):160018. https://www.nature.com/articles/sdata201618, number: 1 Publisher: Nature Publishing Group.
14. Powell CD, Moseley HNB. The Metabolomics Workbench File Status Website: A Metadata Repository Promoting FAIR Principles of Metabolomics Data. BMC Bioinformatics 2023 Jul;24(24):299. https://bmcbioinformatics.biomedcentral.com/articles/10.1186/s12859-023-05423-9.
15. Keane TM, O'Donovan C, Vizcaíno JA. The growing need for controlled data access models in clinical proteomics and metabolomics. Nature Communications 2021 Oct;12(1):5787. https://www.nature.com/articles/s41467-021-26110-4, number: 1 Publisher: Nature Publishing Group.
16. Elasticsearch: The Official Distributed Search & Analytics Engine;. https://www.elastic.co/de/elasticsearch.
17. Python to MariaDB Connector; 2020. https://mariadb.com/resources/blog/how-to-connect-python-programs-to-mariadb/.
18. Wishart DS, Guo A, Oler E, Wang F, Anjum A, Peters H, et al. HMDB 5.0: the Human Metabolome Database for 2022. Nucleic Acids Research 2022 Jan;50(D1):D622–D631.
19. elasticsearch: Python client for Elasticsearch;. https://github.com/elastic/elasticsearch-py.
20. Vue.js - The Progressive JavaScript Framework | Vue.js;. https://vuejs.org/.
21. Bierman G, Abadi M, Torgersen M. Understanding TypeScript. In: Jones R, editor. ECOOP 2014 – Object-Oriented Programming Lecture Notes in Computer Science, Berlin, Heidelberg: Springer; 2014. p. 257–281.
22. TypeScript: JavaScript With Syntax For Types.;. https://www.typescriptlang.org/.
23. Pinia | The intuitive store for Vue.js;. https://pinia.vuejs.org.
24. Angular Data Grid: Documentation;. https://www.ag-grid.com/angular-data-grid/.
25. The official home of the Python Programming Language; 2023. https://www.python.org/.
26. Grinberg M. Flask Web Development: Developing Web Applications with Python. 1st ed. O'Reilly Media, Inc.; 2014.
27. Harris CR, Millman KJ, van der Walt SJ, Gommers R, Virtanen P, Cournapeau D, et al. Array programming with NumPy. Nature 2020 Sep;585(7825):357–362. https://www.nature.com/articles/s41586-020-2649-2, number: 7825 Publisher: Nature Publishing Group.
28. McKinney W. Data Structures for Statistical Computing in Python. Austin, Texas; 2010. p. 56–61. https://conference.scipy.org/proceedings/scipy2010/mckinney.html.
29. Hunter JD. Matplotlib: A 2D Graphics Environment. Computing in Science & Engineering 2007 May;9(3):90–95. Conference Name: Computing in Science & Engineering.
30. Waskom ML. seaborn: statistical data visualization. Journal of Open Source Software 2021 Apr;6(60):3021. https://joss.theoj.org/papers/10.21105/joss.03021.
31. xmltodict: Makes working with XML feel like you are working with JSON;. https://github.com/martinblech/xmltodict.
32. Flask-Cors: A Flask extension adding a decorator for CORS support;. https://github.com/corydolphin/flask-cors.
33. flask-swagger-ui: Swagger UI blueprint for Flask;. https://github.com/sveint/flask-swagger-ui.
34. Flask-JWT-Extended: Extended JWT integration with Flask;. https://github.com/vimalloc/flask-jwt-extended.
35. Werkzeug: The comprehensive WSGI web application library.;. https://palletsprojects.com/p/werkzeug/.
36. Gunicorn - WSGI Server;. https://docs.gunicorn.org/en/stable/.
37. Hupp A, python-magic; 2023. https://github.com/ahupp/python-magic.
38. R Core Team. R: A Language and Environment for Statistical Computing. R Foundation for Statistical Computing, Vienna, Austria; 2022, https://www.R-project.org/.
39. RestRserve: A Framework for Building HTTP API;. https://restrserve.org/.
40. Klein MS. Affine Transformation of Negative Values for NMR Metabolomics Using the mrbin R Package. Journal of Proteome Research 2021 Feb;20(2):1397–1404. https://doi.org/10.1021/acs.jproteome.0c00684.
41. Zacharias HU, Schley G, Hochrein J, Klein MS, Köberle C, Eckardt KU, et al. Analysis of human urine reveals metabolic changes related to the development of acute kidney injury following cardiac surgery. Metabolomics 2013 Jun;9(3):697–707. https://doi.org/10.1007/s11306-012-0479-4.
42. Zacharias HU, Hochrein J, Vogl FC, Schley G, Mayer F, Jeleazcov C, et al. Identification of Plasma Metabolites Prognostic of Acute Kidney Injury after Cardiac Surgery with Cardiopulmonary Bypass. Journal of Proteome Research 2015 Jul;14(7):2897–2905.
43. Fino NF, Adingwupu OM, Coresh J, Greene T, Haaland B, Shlipak MG, et al. Evaluation of novel candidate filtration markers from a global metabolomic discovery for glomerular filtration rate estimation. Kidney international 2024;105(3):582–592.
44. Scalability and resilience: clusters, nodes, and shards | Elasticsearch Guide [8.10] | Elastic; 2023. https://www.elastic.co/guide/en/elasticsearch/reference/current/scalability.html.
45. Sreekumar D, Trends in research collaborations: An overview | Researcher.Life; 2022. https://researcher.life/blog/article/trends-in-research-collaborations/.
46. Jones M, Bradley J, Sakimura N. JSON Web Token (JWT). Internet Engineering Task Force; 2015.
47. Kellum JA, Lameire N, Aspelin P, Barsoum RS, Burdmann EA, Goldstein SL, et al. Kidney disease: improving global outcomes (KDIGO) acute kidney injury work group. KDIGO clinical practice guideline for acute kidney injury. Kidney international supplements 2012;2(1):1–138.
48. Allaway RJ, La Rosa S, Verma S, Mangravite L, Guinney J, Blakeley J, et al. Engaging a community to enable disease-centric data sharing with the NF Data Portal. Scientific data 2019;6(1):319.
49. Wolstencroft K, Owen S, Krebs O, Nguyen Q, Stanford NJ, Golebiewski M, et al. SEEK: a systems biology data and model management platform. BMC systems biology 2015;9:1–12.
50. Wolstencroft K, Krebs O, Snoep JL, Stanford NJ, Bacall F, Golebiewski M, et al. FAIRDOMHub: a repository and collaboration environment for sharing systems biology research. Nucleic acids research 2017;45(D1):D404–D407.

Manuscript_tracked changes
Click here to access/download;Manuscript;MetaboSERV_Manuscript_diff.pd

Placeholder for OUP logo oup.pdf

GigaScience, 2024, 1–13

doi: xx.xxxx/xxxx
Manuscript in Preparation
Technical Note

TECHNICAL NOTE

MetaboSERV - a platform for selecting, exchanging, and visualizing metabolomics data with controlled data access

Tim Tucholski[1,*], Angela Maennel[2], Yacoub Abelard Njipouombe Nsangou[1,3], Sven Schuchardt[4], Matthias Gruber[5], Fabian Kellermeier[5], Katja Dettmer[5], Peter J. Oefner[5], Wolfram Gronwald[5], Michael Altenbuchinger[1], Jürgen Dönitz[1,3,6,†] and Helena U. Zacharias[2,*,†]

[1]Department of Medical Bioinformatics, University of Göttingen and [2]Peter L. Reichertz Institute for Medical Informatics of TU Braunschweig and Hannover Medical School, Hannover Medical School and [3]Institute of Computational Biology, Helmholtz Center Munich and [4]Department of Bio- and Environmental Analytics, Fraunhofer ITEM, Hannover and [5]Institute of Functional Genomics, University of Regensburg and [6]Campus Institute Data Science (CIDAS) Göttingen

[*]Correspondence: zacharias.helena@mh-hannover.de (Helena U. Zacharias); tim.tucholski@med.uni-goettingen.de (Tim Tucholski)
[†]These authors contributed equally to this work.

Abstract

Background
The growing number of metabolomics studies, based on high-dimensional data measured by hyphenated mass spectrometry (MS) and/or nuclear magnetic resonance (NMR) spectroscopy, has sparked the creation of several public metabolomics data repositories. Each repository emphasizes different aspects regarding data selection and representation, but most offer only limited options for privacy-preserving data sharing.
Results
We present MetaboSERV, an open-source, browser-based metabolomics platform dedicated to the selection, integration and sharing of quantitative metabolomics data and metadata with controlled data access. MetaboSERV aims to aid researchers in analyzing their results by facilitating means to browse, visualize and compare data across available data sets. It provides different access control functionalities, creating an environment in which data can be shared safely in a privacy-preserving manner to support collaborative and interdisciplinary research. Furthermore, it is designed to be extensible and adaptable to existing data management infrastructures through the creation of self-managed MetaboSERV instances, for which we provide the source code and a set of ~~pre-built~~ configurable Docker images.
Conclusions
~~A~~ The public MetaboSERV instance is available at https://metaboserv.ckdn.app, and the source code can be found at https://gitlab.gwdg.de/MedBioinf/metabolomics/metaboserv. ~~Docker images can be found at .~~ The Research Resource Identifier (RRID) for ~~MetaboServ~~ MetaboSERV is *SCR_025496*.

Key words: (privacy-preserving) Data Sharing; Metabolomics; Nuclear Magnetic Resonance Spectroscopy; Mass Spectrometry; Collaborative Research

Background

Metabolomics is the comprehensive study and quantitative analysis of all metabolites that are detectable in a biological specimen. It has found a wide range of applications in the medical field, including the identification of biomarkers and elucidation of ~~important biological~~ molecular pathomechanisms in precision medicine [1, 2, 3]. Nuclear magnetic resonance (NMR) spectroscopy and hyphenated mass spectrometry (MS) are the two most widely used analytical methods in metabolomics and are suitable for large-scale studies [4, 5]. In response to the fast increase in the number of metabolomics studies published and the corresponding generation of vast amounts of research data, different metabolomics data repositories such as MetaboLights [6], Metabolonote [7], Metabolomic Repository Bordeaux (MeRy-B) [8], the Metabolomics Workbench [9], and more recent platforms such as MetHoS [10] and COMETS Analytics [11] ~~were~~ have been created. MetaboLights, Metabolomics Workbench, MeRy-B, and Metabolonote primarily focus on fully open public sharing of metabolomics data~~, whereas COMETS Analytics and MetHoS solely accommodate~~ . MeRy-B solely accommodates NMR-based metabolomics plant data~~or~~ , and Metabolonote solely metabolomics metadata, respectively. COMETS Analytics and MetHoS enable comprehensive data analysis of stored experimental data, the former being specifically designed for meta-analyses and the latter with a particular focus on untargeted MS data. The repositories are constantly evolving to fit the needs of the research community [12], and enable researchers to make their experimental data findable, accessible, interoperable, and re-usable as defined by the FAIR principles for scientific data management and stewardship [13, 14].

Existing ~~metabolomics~~ repositories, in particular MetaboLights [6] and Metabolomics Workbench [9], focus on fully open public sharing of experimental data and/or metadata upon ~~publishing~~ publication of study results. They only provide limited options for controlled access sharing of metabolomics data within a specific group of researchers. However, the latter is the typical scenario in an interdisciplinary collaboration, where clinicians, metabolomics experimentalists, and (metabolomics) data scientists/bioinformaticians perform dedicated tasks in a joint metabolomics research project (Fig. 1). To foster collaborative and interdisciplinary research, all ~~collaboration partners, irrespective~~ partners, irrespectively of their (potentially highly diverse) programming skills, should be able to browse and visualize the metabolomics data as well as generate summary statistics and carry out different data analysis tasks within a data privacy preserving environment. Especially biomedical metabolomics data from human studies require specific attention to data security. Just recently, a call for controlled access models for metabolomics data sharing repositories, as a potential requirement due to patient consent statements, personal data regulations such as the European Union General Data Protection Regulation (GDPR) or other relevant legislation, has been issued [15]. This call demonstrates the urgent need of providing metabolomics data repositories with controllable data access.

We present MetaboSERV, an open-source browser-based platform for controlled access sharing of NMR and MS metabolomics data, metadata, and research results. MetaboSERV offers rich and intuitive data selection, browsing and visualization functionalities and aims to facilitate controlled data accessibility within research collaborations, particularly prior to publication of research findings. ~~Besides the web-based~~ The MetaboSERV platform can be employed through either the public MetaboSERV instance, ~~we provide pre-built Docker images to allow researchers the set up of~~ available at `https://metaboserv.ckdn.app`, or fully autonomous, self-managed~~MetaboSERV instances within their research environments~~, local MetaboSERV instances that can be set up and operated by end-users utilizing our configurable Docker images and detailed user guides.

Methods

Implementation

Data Storage

~~Research~~ Metabolite concentration data and associated metadata (such as phenotype data) are stored in two different databases, namely an Elasticsearch [16] instance running on version 8.3 and a MariaDB instance running on version 10.11, with a Python interface [17], respectively. Other experimental data (such as raw spectral data) that can be uploaded to MetaboSERV are stored on the server MetaboSERV is hosted on. MariaDB contains numerical data such as metabolite ~~concentration levels~~ concentrations and reference values retrieved from the Human Metabolome Database (HMDB), version 5.0 [18]. Elasticsearch contains user account data, phenotype data and study metadata. The schemaless data storage provided by Elasticsearch enables MetaboSERV to be flexible with regards to metadata that the user uploads for a study. The Python Elasticsearch Client [19] acts as a wrapper around Elasticsearch, providing basic database querying functions that are then translated into Elasticsearch Query Domain Specific Language (DSL) queries.

User Interaction

The MetaboSERV web interface is tailored to facilitate seamless user interaction. It is based on the VueJS3 [20] framework and mainly implemented in TypeScript [21, 22]. In addition, it makes use of the ~~client-side store~~ client-side-store capabilities provided by Pinia [23] and the AG Data Grid [24] package for table creation.

Supplementary Web Services

MetaboSERV utilizes two web services to (1) offer an application programming interface (API) and (2) process raw metabolomics NMR data. The first web service is implemented in Python [25] and uses the package flask [26] to provide the API for supplying data to the web application. Non-native packages used for the service include numpy [27], pandas [28], matplotlib [29], seaborn [30], and xmltodict [31]. Flask-related add-ons include flask-cors [32], flask-swagger-ui [33], flask-jwt-extended [34], and werkzeug [35]. The package gunicorn [36] is used as a production WSGI server on top of flask. The package python-magic [37] helps with file validation. The second web service for processing raw metabolomics data, including raw NMR spectra, is *R*-based [38] and makes use of the packages restRserve [39] and mrbin [40]. For a complete list of used TypeScript, Python and *R* packages and their respective versions, please refer to Supplementary Table S1.

Data privacy and security

Following the principles of personal data minimization in the GDPR, only required personal data is collected and stored in MetaboSERV. New users need to provide an e-mail address upon registration, which is automatically validated and can be used to recover lost passwords, which are saved securely encrypted in the database (salted and hashed). Communication between all modules is handled using Hypertext Transfer Protocol (HTTP) or preferably HTTP-secure (HTTPS) requests and responses. For all non-internal communication, HTTPS is enforced. More details are provided in the Supplementary Section "User Authentication and Password

Compiled on: March 18, 2025.
Draft manuscript prepared by the author.

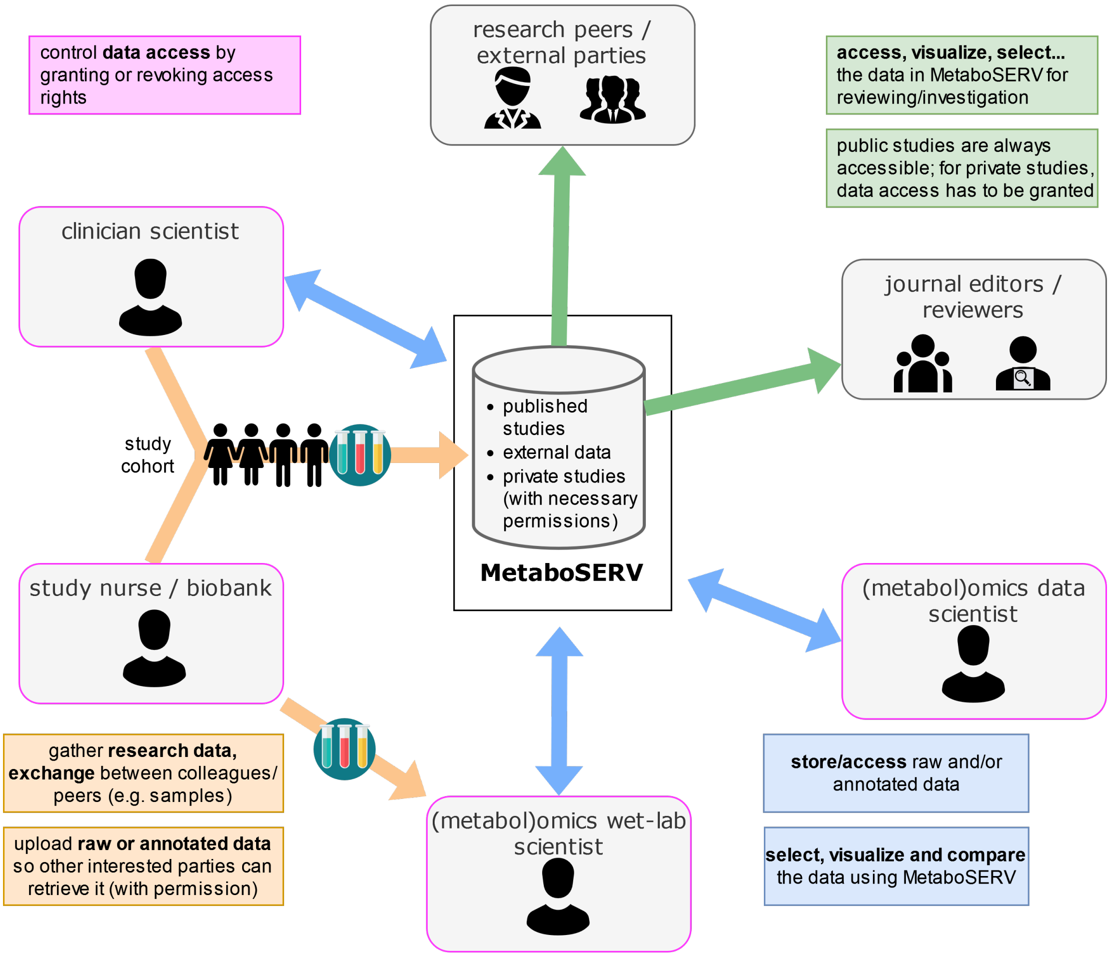

Figure 1. An exemplary schema of how the MetaboSERV platform can connect interdisciplinary collaborators of a research project as well as external parties: clinician scientists and study nurses gather phenotypic information and biofluid specimens from the study cohort, which are further measured by metabolomics wet-lab scientists. All three collaboration partners can store their collected raw and processed data ~~in~~ on the MetaboSERV platform, by either employing the public MetaboSERV instance or a local MetaboSERV instance, autonomously set-up at one of the collaborating institutions. (Metabol)omics data scientists can access the data in MetaboSERV, analyse it and upload further results to MetaboSERV. Additional access can be granted to research peers that are interested in the data, as well as to journal editors and reviewers to facilitate peer-review.

Storage".

Metabolomics metadata

Metabolite metadata consisting of reference concentration ranges for healthy humans for the most common human biofluids (urine, plasma, serum, feces and cerebral spinal fluid) and synonym lists were retrieved from the HMDB, version 5.0 [18].

Metabolomics use case data

NMR data from a previous study on 106 patients undergoing cardiac surgery [41] served as an ~~examplary~~ exemplary dataset for MetaboSERV and was used to guide the implementation process. 34 of the 106 patients had been diagnosed with postoperative acute kidney injury (AKI) [41]. It includes 1D ^{1}H NMR spectra from urine specimens collected from all study participants 24 hrs after cardiac surgery with cardiopulmonary bypass (CPB) use. These spectra were acquired using a 600 MHz Bruker Avance III spectrometer (Bruker BioSpin GmbH, Ettlingen, Germany). Additionally, 1D ^{1}H NMR spectra from 85 plasma specimens of the same study participants were collected, measured, and absolutely quantified as described in [42].

A ~~third~~ second use case data set includes absolute concentrations of 630 metabolites measured in 9 ~~NIST~~ aliquots of the NIST frozen human plasma Standard Reference Material 1950 (SRM 1950)~~human plasma specimens~~. Data was acquired on an AB Sciex 6500+ triple quadrupole mass spectrometer (AB Sciex Germany GmbH, Darmstadt, Germany) coupled to an ExionLC 30AD (AB Sciex Germany GmbH, Darmstadt, Germany) ultra-high performance liquid chromatography (UHPLC) system employing the MxP Quant 500 kit (Biocrates life sciences, Innsbruck, Austria).

A third use case data set consists of 1,228 unique metabolites semi-quantitatively measured on the Metabolon H4 platform in 1,002 human blood plasma specimens [43]. Data was acquired on a Thermo Scientific Q-Exactive high resolution/accurate mass spectrometer interfaced with a heated electrospray ionization (HESI-II) source and utilizing a Waters ACQUITY ultra-performance liquid chromatography (UPLC) system. A methanol extraction was performed for protein precipitation and the resulting extract of each specimen was divided into five fractions: two fractions were used for analysis by two separate reversed phase (RP)/UPLC-MS/MS methods with positive ion mode electrospray ionization (ESI), one fraction was used for analysis by RP/UPLC-MS/MS with negative ion mode ESI, one fraction was used for analysis by HILIC/UPLC-MS/MS with negative ion mode ESI, and one aliquot was reserved for backup. Data on the original scale, i.e., values normalized in terms of raw area counts without missing value imputation as provided by Metabolon, were downloaded from the Metabolomics Workbench, `https://www.metabolomicsworkbench.org`, Project ID PR001762.

Results

MetaboSERV architecture

MetaboSERV is a web-based, open-source metabolomics platform, specifically designed for controlled user access and cross-comparison between studies. It includes four interconnected modules, the *MetaboSERV Web Interface*, the *Backend Service*, the *Databases*, and the *Raw Data Parser*, as presented in Fig. 2.

The *MetaboSERV Web Interface* serves as an interface to all functionalities and data contained in MetaboSERV, facilitating seamless interaction with the user. Raw experimental data, e.g., spectra derived from NMR or MS experiments, can also be stored in MetaboSERV. However, raw experimental data is saved as-is on the server without any additional processing. MetaboSERV further includes an *R*-based *Raw Data Parser*, which is capable of parsing, processing and visualizing NMR raw frequency domain data provided in the Bruker format. Finally, in order to encapsulate ~~"~~"create, read, update and delete (CRUD)~~"~~" operations to the databases and to add logic and visualization options, MetaboSERV includes an extensible web service, referred to as the *Backend Service*, that mediates between the web interface and the other modules. The *Backend Service* also handles user authentication and authorization as well as file validation measures.

MetaboSERV server environment

~~MetaboSERV is~~ The public MetaboSERV instance, available at `https://metaboserv.ckdn.app`, is hosted at the computing center of the Hannover Medical School, Germany (MHH). MHH's regulations for access control to the server, security updates, backup and monitoring are implemented following the ISO 27001 and the standards of the German Federal Office for Information Security (Bundesamt für Sicherheit in der Informationstechnik, BSI). In particular, the public MetaboSERV server at MHH and the data stored there can only be accessed by authorized administrators. Server access is continuously logged and regularly inspected. The virtual machine is equipped with the latest security updates and regular backups are being taken every six hours.

Local MetaboSERV instances can be set up by cloning our repositories at `https://gitlab.gwdg.de/MedBioinf/metabolomics/metaboserv` and creating, configuring, and running the respective Docker images. Detailed user guides are provided in Supplementary File S6: "Detailed installation and user guide for the set-up of local MetaboSERV instances" as well as at `https://metaboserv.ckdn.app/guide` and in our GitLab repositories. MetaboSERV is generally resource-friendly, all core components can be set up on a dual-core machine with ~~6GB~~ 6 GB of random access memory (RAM) and ~~200GB~~ 200 GB of hard disk space for MariaDB and Elasticsearch. Furthermore, a sufficient amount of hard disk space is necessary to store raw metabolomics data. Query speed is heavily dependent on the resources attributed to the underlying Elasticsearch and MariaDB instances. Therefore, it is recommended to set up MetaboSERV on a machine with at least four cores and ~~16GB~~ 16 GB of RAM and to take advantage of Elasticsearch's *sharding* mechanism [44]. By default, MetaboSERV makes no assumptions about the Elasticsearch environment to avoid structural and capacity-related issues. These self-managed MetaboSERV instances run isolated from the public MetaboSERV instance and allow hosting and managing data on self-governed servers, removing any further data privacy concerns. They can also be altered and configured to fit different research environments and data formats, and assure a degree of system portability due to the nature of container virtualization. More information on user-specific configuration settings are provided in Supplementary File S6.

Data upload and processing

In ~~MetaboSERV~~the MetaboSERV platform, a *study* encapsulates the uploaded experimental data, annotations (such as phenotypes) and metadata, e.g., the study owner and collaborators. The study creation process is represented in Fig. 3A. The user can either upload raw experimental data, absolutely quantified metabolite concentration data or both, and the study must contain mandatory metadata (study authors, at least one biospecimen, at least one analytical method and a year or range of years associated with the study). Raw experimental data of any format, bundled with a common tool like gzip or zip, can be uploaded to and retrieved from MetaboSERV. Concentration data represented as any of the common file formats

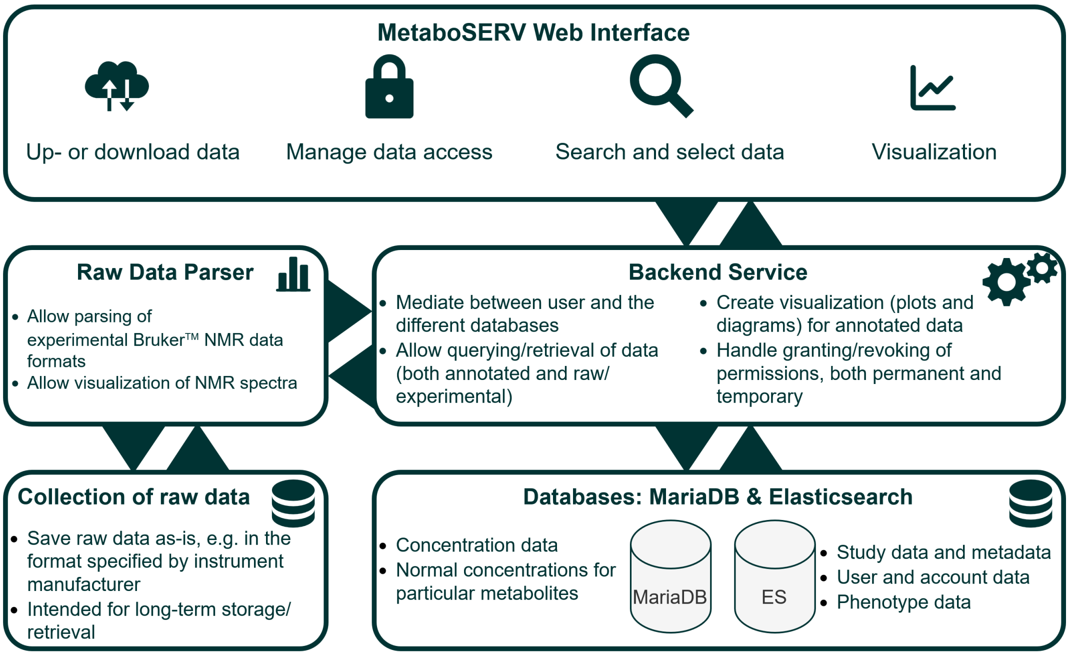

Figure 2. The four modules of MetaboSERV. Users interact solely with the *MetaboSERV Web Interface*, while the *Backend Service* mediates between the user and the *Databases* as well as the *Raw Data Parser* for parsing ~~and supplying raw data such as~~ Bruker NMR ~~and MS~~ spectra.

TSV, CSV (e.g., bucket tables), XLS or XLSX is accepted. It is also possible to add an additional file with phenotype data. The concentration ~~data~~ and phenotype data files, however, have to adhere to the layout specified by MetaboSERV, as outlined in Supplementary File S3. A validation procedure, which verifies that data fit the requirements by checking the file structure, is performed instantaneously before the data are further processed and added to MetaboSERV. Finally, additional arbitrary metadata can be added by uploading a JSON/YAML metadata file, as detailed in Supplementary File S3. It is also possible to specify metadata directly, which will overwrite any uploaded metadata with conflicting entries.

Data access control and management

~~MetaboSERV~~ The MetaboSERV platform is built around the notion of collaborative research work [45] and aims to simplify the exchange of experimental data and metadata, irrespective of whether the data are meant to be published or to remain private. Each study can be managed separately, as shown in Fig. 3B. In addition to showcasing phenotypes and any study metadata, it ~~also~~ allows to modify the metadata.

In order to guarantee data privacy, two different access control mechanisms are implemented ~~and~~ for both public and any local MetaboSERV instances. They can be used independently of or in combination with each other. Each MetaboSERV user, who wants to upload data to ~~MetaboSERV~~the MetaboSERV platform, first needs to create an individual user account. Users are ~~only~~ required to provide a username, e-mail address, and password, which is stored encrypted (salted and hashed, please refer to Supplementary Section ~~"~~S2 "User Authentication and Password Storage~~"~~" for more details), to register, and neither their account names nor their personal data are ~~shown~~ revealed to other users~~in the MetaboSERV web interface. Authentication is handled through JSON web tokens [46] (JWTs), which are associated with each user and newly generated on each log-in~~. Upon registration, an e-mail containing a verification code is sent to the e-mail address that was used to register the account. This unique code, which consists of ten random characters, must be entered once after logging in to unlock any permissions associated with the account, which includes viewing studies shared with the account or uploading studies. The user accounts are associated with access rights for specific studies. For each uploaded data set, two different levels of access rights can be granted to other user accounts – either ~~"~~"read-only permissions~~" or "~~" or "full data editing and management authority~~"~~" – by the original uploader or users with ~~"~~"full data editing and management authority~~"~~" accounts. The account responsible for the initial creation of the study possesses ~~"~~"full data editing and management authority~~"~~" at any time and can never be removed as a contributor by any other user. Authentication during the log-in process is handled through JSON web tokens [46] (JWTs), which are associated with each user and newly generated on each log-in. Unauthorized access attempts are continuously logged in the Elasticsearch database. The number of unauthorized access attempts, which occurred since the last authorized log-in, is reported to the user upon every log-in. In the public MetaboSERV instance, an alert e-mail is sent to the user's e-mail address linked to the account in case the number of unauthorized access attempts exceeds five attempts, and the account is locked for ten minutes after a total of ten tracked unauthorized access attempts. On a successful log-in attempt, this counter is reset to zero. More information and customization details for local MetaboSERV instances are provided in Supplementary File S2.

Additionally, all users with ~~"~~"full data editing and management authority~~"~~" have the ability to create authorization tokens. These, in contrast to JWTs, are independent of user accounts. Each token can be associated with an expiration date, a set of permissions for one or more data sets, and a comment. Logging in with an authorization token grants the set of permissions specified by the token creator without having to create a user account, as long as the token is valid and not yet expired. This feature is intended to enable ~~short–time~~ short-term sharing of data, such as providing project results to a peer or journal editor/reviewer for validation, by minimizing the effort required to access the data: Simply sharing the token will allow the recipient to view – or even edit, if ~~desired~~ allowed – the selected data.

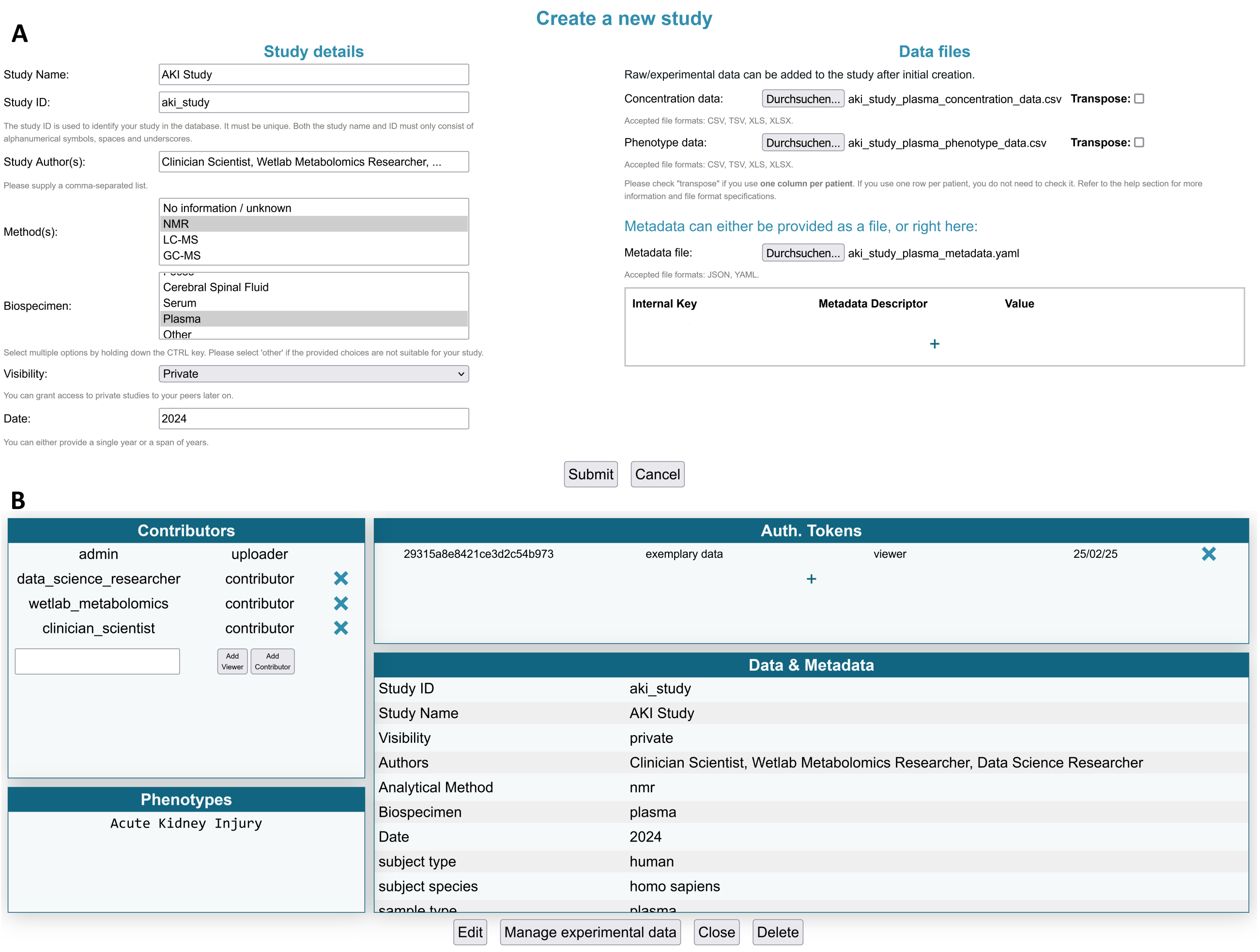

Figure 3. **A** MetaboSERV platform data upload menu. Several files and metadata attributes can be provided for a study. ~~Raw data as well as concentration~~ Concentration and phenotype data can be added (~~at least one out of three~~ concentration data is required). Metadata can be provided by means of a JSON/YAML file or directly in the application. **B** MetaboSERV study management menu. Access can be granted to other users, either ~~"~~“read-only~~"~~” access or (limited) ~~"~~“write~~"~~” access (top left). ~~It~~ Access can ~~also~~ be revoked~~again~~, though the study uploader privileges can neither be removed nor altered at any time. Available phenotypic information of the stored study is listed under ~~"~~“Phenotypes~~"~~” (bottom left). Authorization tokens for a particular study can be added/removed, if desired with specific expiration dates, comments and additional studies that are covered by the token (top right). A summary of study data and metadata is provided under ~~"~~“Metadata~~"~~” (bottom right). ~~Clicking~~ By clicking on ~~an entry lets~~ “Edit”, the user ~~alter~~ can edit the ~~contents~~study, while clicking on “Manage experimental data” allows the user to up- or download experimental data.

~~Self-managed MetaboSERV instances~~

~~In addition to the browser-based MetaboSERV instance, we offer a set of configurable Docker images, freely available at , that allow the creation of self-managed MetaboSERV instances. Set-up requirements are listed in the section "Availability of source code and requirements" below.~~

~~These self-managed MetaboSERV instances run isolated from the centralized MetaboSERV platform and allow hosting and managing data on self-governed servers, removing any further data privacy concerns. They can also be altered and configured to fit different research environments and data formats, and assure a degree of system portability due to the nature of container virtualization.~~

Data search and selection

The core feature of ~~MetaboSERV~~ the MetaboSERV platform is a many-faceted, intuitive data selection system that allows for simple and complex queries alike. By configuring search parameters in the web interface, users can specify exactly the data or studies suitable for their research task or use case, thereby filtering out superfluous data. Data can be selected according to studies, metabolites, as well as phenotypes (Fig. 4), and subsequently either be analyzed further in MetaboSERV or downloaded for other purposes. This also facilitates straightforward integration of different studies or research projects, as data from several data sets can be combined arbitrarily, as long as the user has acquired permission to view the respective data.

Data selection revolves around finding data that fit the physiological and phenotypical criteria outlined by the user. In a first step, users can select all studies they want to include in their query (Fig. 4A). Any number of studies can be combined. Next, metabolites can be specified, *e.g.*, to represent the target metabolic profile (Fig. 4B). By default, all metabolite concentration levels are retrieved from the selected studies. It is also possible to exclude a subset of metabolites (Fig. 4B). In an optional next step, users can add ~~further~~ constraints by specifying individual concentration ranges for the inclusion or exclusion of ~~metablite~~ metabolite levels (Fig. 4C). Additionally, it is possible to supply a range only for visualization purposes (without affecting the query results) by choosing the filter type ~~"vis"~~ “vis”. The corresponding metabolite concentration ranges can either be entered by the user, or they can be selected from a pre-defined collection of different reference concentrations from the HMDB [18].

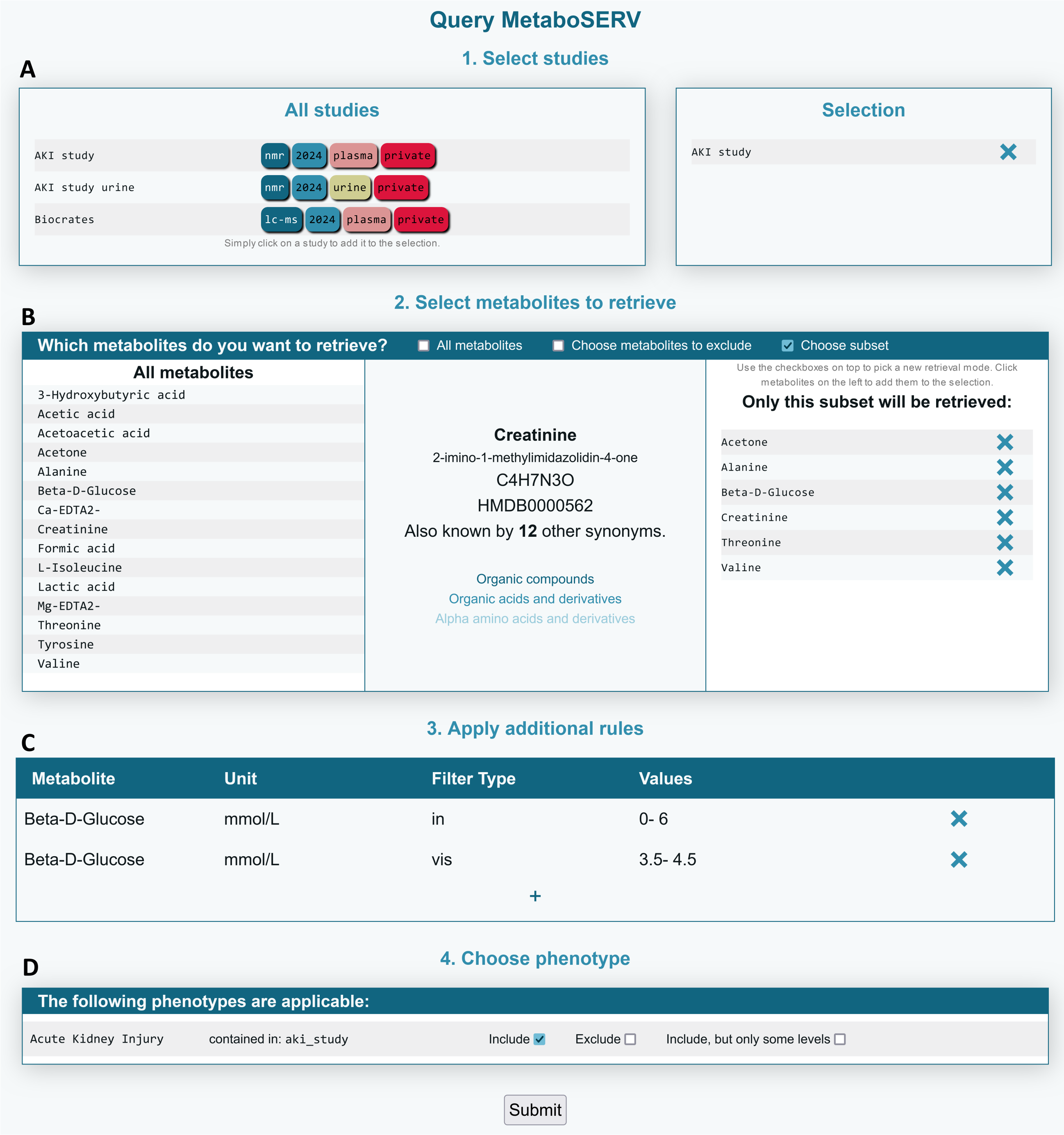

Figure 4. An example query to the public MetaboSERV database. **A** Study selection menu. All accessible studies are shown on the left and can be selected for a query. In this case, only the *AKI study* was selected, consisting of absolutely quantified concentration values of plasma metabolites measured by NMR spectroscopy. **B** Metabolite selection menu. By default, all metabolites are retrieved. If desired, only a particular subset will be retrieved or excluded, respectively. Metabolite information, such as for creatinine, is shown if the user hovers over a metabolite entry. **C** Metabolite filter menu. Specific filtering rules, such as specifying that the concentration level of a particular metabolite has to be included in or excluded from a certain range, can optionally be added here. Metabolite concentration ranges for healthy individuals provided by the HMDB may be used as filter options, too. Alternatively, only a selected concentration range can ~~only~~ be visualized without altering the set of results. In this example, all entries with ~~glucose~~ beta-D-glucose levels above 6 mmol/L are ignored, while entries with a ~~glucose~~ beta-D-glucose level between 3.5 and 4.5 mmol/L will be highlighted. **D** Phenotype selection menu. By default, phenotype data is retrieved completely. A subset of phenotypes or phenotype levels can also be selected.

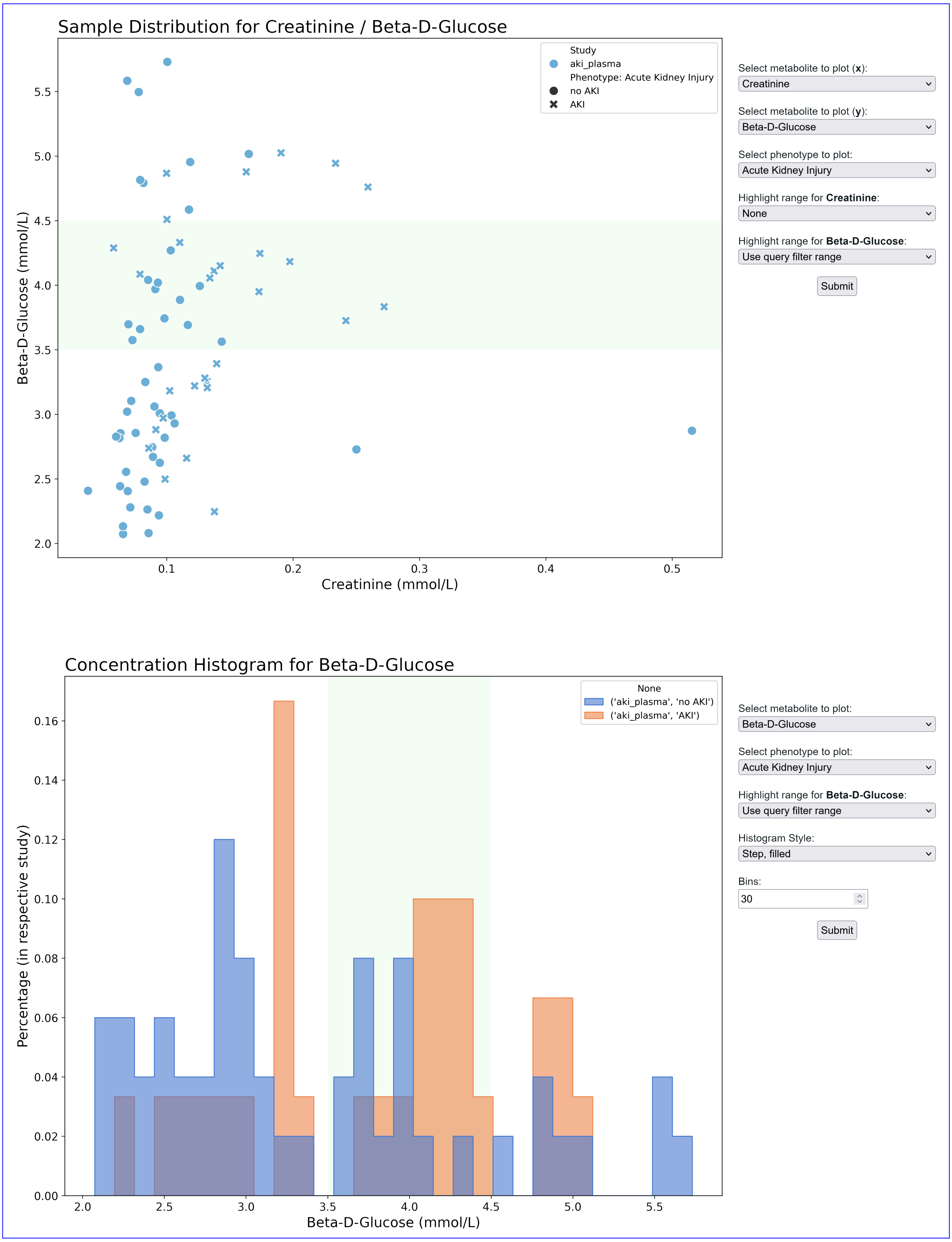

Figure 5. Results of the query pictured in Fig. 4, shown in the form of a ~~concentration histogram and a~~ sample distribution scatterplot and a concentration histogram, respectively. The metabolites and phenotypes to be plotted can be selected by the user. Specimens with ~~glucose~~ beta-D-glucose levels between 3.5 and 4.5 mmol/L are highlighted, as specified in the query in Fig. 4C and by using the "Use query filter range" option in the "Highlight range" field as seen in the menu on the right.

Source ID	Study	Acute Kidney Injury	Acetone	Alanine	Beta-D-Glucose ↓	Creatinine	Threonine
AKI_51	aki_study	no AKI	1.91072 mmol/L	0.103263 mmol/L	5.73048 mmol/L	0.100439 mmol/L	
AKI_19	aki_study	no AKI	0.357399 mmol/L	0.16642 mmol/L	5.58404 mmol/L	0.0687475 mmol/L	
AKI_20	aki_study	no AKI	0.144835 mmol/L	0.153249 mmol/L	5.49683 mmol/L	0.0778023 mmol/L	
AKI_18	aki_study	AKI	0.0228864 mmol/L	0.417078 mmol/L	5.02584 mmol/L	0.190397 mmol/L	0.116662 mmol/L
AKI_55	aki_study	no AKI	0.0268556 mmol/L	0.241009 mmol/L	5.01733 mmol/L	0.164905 mmol/L	0.0530378 mmol/L
AKI_95	aki_study	no AKI	0.316609 mmol/L	0.193356 mmol/L	4.95481 mmol/L	0.118549 mmol/L	0.0914211 mmol/L
AKI_06	aki_study	AKI	0.0806513 mmol/L	0.24828 mmol/L	4.94507 mmol/L	0.233604 mmol/L	
AKI_94	aki_study	AKI	0.0834382 mmol/L	0.254088 mmol/L	4.87885 mmol/L	0.162888 mmol/L	0.0780183 mmol/L
AKI_46	aki_study	AKI	0.118823 mmol/L	0.169484 mmol/L	4.8676 mmol/L	0.0998487 mmol/L	0.0759844 mmol/L
AKI_59	aki_study	no AKI	0.0574271 mmol/L	0.217274 mmol/L	4.81593 mmol/L	0.0789833 mmol/L	0.10618 mmol/L
AKI_104	aki_study	no AKI	0.0211974 mmol/L	0.363983 mmol/L	4.79278 mmol/L	0.0814931 mmol/L	0.0971578 mmol/L

1 to 11 of 80 |< < Page 1 of 8 > >|

Figure 6. Results of the query pictured in Fig. 4, shown as a table which is sorted according to ~~glucose~~ beta-D-glucose levels in descending order. Concentration values that passed the filtering rules set by the user are highlighted in green. As the filtering rules removed patients with ~~glucose~~ beta-D-glucose levels above 6 mmol/L, the entire ~~glucose~~ beta-D-glucose column is highlighted in green. Phenotypes, such as *Acute Kidney Injury* and the according levels are also shown. Missing values are represented as empty cells.

In a final step, users can choose to include phenotype data (or only particular levels of a phenotype, such as ~~"healthy"~~ “healthy”), if such data are available for any of the selected data sets (Fig. 4D). Phenotype data does not directly affect the data selection process, but can be used for subsequent data analysis and visualization purposes. Query results can be displayed and browsed in a table (see Fig. 6) or visual representation (as shown in Fig. 5). It is also possible to inspect the underlying experimental data, given that it matches a supported format. Currently, raw NMR free induction decays (FIDs) as well as spectra in the frequency domain in the Bruker file format are supported.

Data ~~set uploaded to MetaboSERV~~sets uploaded to the public MetaboSERV instance, as well as combinations or subsets thereof, can be browsed and visualized in the public MetaboSERV web interface, provided the user may access the respective data. Local MetaboSERV instances are initially set up without any public use case data sets, which are, however, available for download at `https://metaboserv.ckdn.app.` Histograms, scatterplots and heatmaps depicting the Pearson correlation between different metabolites (an example is provided in Fig. S2) are created automatically. Different subgroups, such as those defined by phenotypes, are also taken into account (see Fig. 5). They can be configured and created on demand for different combinations of nominal phenotypes or metabolites. Concentration values can be displayed in a table, which highlights whether each value is contained in a selected range or not and additionally displays selected phenotype data for each entry (Fig. 6). ~~Raw frequency data , for example NMR spectral data,~~ NMR raw frequency data in the Bruker file format can also be displayed~~, provided the data format is supported by MetaboSERV~~. Finally, MetaboSERV facilitates the automatic creation of quality control plots (see example provided in Supplementary File S4: Use case 2 and Figure S3), allowing researchers to quickly gauge the amount of missing values, *i.e.* concentration values below the limit of detection (LOD). Here, the user can create further plots showing the distribution of missing values per metabolite across all measured samples, and toggle between showing values over or under the LOD for both types of plots.

Data retrieval

Research data, which includes both public data and private data given the required permissions, can also be retrieved from ~~MetaboSERV~~the MetaboSERV platform. This applies to retrieving any raw data as well as generating new documents containing quantified metabolite concentration data for any study or subsets thereof. Currently, JSON, CSV and XLSX formats are supported. Created plots are also available for download in PNG format.

Use case

We demonstrate the capabilities of ~~MetaboSERV~~ the MetaboSERV platform in an exemplary use case:

A consortium of clinician scientists, wet-lab metabolomics and data science researchers carries out a metabolomics study on patients undergoing cardiac surgery. The clinician scientists have sent blood plasma specimens to the metabolomics wet lab ~~, where those have been measured~~ for measurement by NMR spectroscopy. Furthermore, the clinician scientists have created a new study on ~~MetaboSERV~~the public MetaboSERV instance, named it ~~"AKI study"~~ “AKI study” (Fig. 3A), and uploaded the corresponding phenotype data and study metadata. They further add the wet-lab metabolomics and data science researchers as ~~"contributors"~~ “contributors” to the study (Fig. 3B). The former upload the measured experimental data, in this case, both raw and absolute concentration data to this MetaboSERV study. The goal of this metabolomics study is to identify possible associations between post-operative acute kidney injury (AKI) and particularly creatinine as well as ~~glucose~~beta-D-glucose, but also additional metabolites. The statistical analyses are carried out by the data science researchers, who select the ~~"AKI study"~~ “AKI study” as well as the metabolites creatinine and ~~glucose~~beta-D-glucose, as well as acetone, alanine, threonine and valine in the MetaboSERV query interface (Fig. 4). As the researchers are not interested in effects of extremely high ~~glucose~~ beta-D-glucose levels, they filter out any plasma specimen with a ~~glucose~~ beta-D-glucose level above 6 mmol/L. They also highlight plasma specimens with ~~glucose~~ beta-D-glucose levels between 3.5 and 4.5 mmol/L using a filter of type ~~"vis"~~ “vis” (Fig. 4C). Finally, the phenotype ~~"~~“Acute Kidney Injury~~"~~” is selected. To graphically explore the hypothesized association between AKI and ~~glucose~~beta-D-glucose, the researchers generate two different visualizations: A histogram of the ~~glucose~~ beta-D-glucose distribution stratified according to AKI diagnosis, and a scatterplot depicting the relation between ~~glucose~~ beta-D-glucose and creatinine, as elevated blood creatinine levels are a strong indicator for impaired renal function and thus a marker for AKI [47] (Fig. 5). A clear association between higher ~~glucose~~

beta-D-glucose levels and AKI diagnosis can be seen in both visualizations. Finally, the wet-lab metabolomics researchers want to show the metabolite concentration data to external collaboration partners. As they only want to provide them with a temporary data access for seven days without any editing permissions, they generate the corresponding authentification token ~~"exemplary data"~~ "exemplary data" (see Fig. 3B) and share it with their collaboration partners. ~~A second exemplary use case~~ Two additional exemplary use cases, the first demonstrating a quality assessment of mass spectrometry data using MetaboSERV ~~is~~ and the second illustrating MetaboSERV's capabilities of handling a large-scale, multi-modal, untargeted mass spectrometry data set, are provided in Supplementary ~~Section "~~Sections "3.4 File S4: Use case 2: Quality assessment of mass spectrometry data~~"~~" and "3.5 File S5: Use case 3: Large-scale, multi-modal, untargeted mass spectrometry data".

Discussion

MetaboSERV is a browser-based, extensible metabolomics platform with a focus on ~~absolutely~~ (absolutely) quantified metabolomics research data from NMR and LC-MS measurements. A major goal of MetaboSERV is to provide researchers with the means to autonomously control access to their metabolomics data. ~~MetaboSERV~~ The MetaboSERV platform differentiates between public and private data and offers two distinct and independent ways to grant access to private data sets, namely account- and token-based authentication. Data access can be limited to a customized ~~timeframe~~ time frame or revoked at any time by the owner, i.e., the user who originally uploaded the data set. Thus, MetaboSERV provides the research community with a privacy-preserving platform for exchanging metabolomics data and research findings. It is particularly designed for interdisciplinary collaborations between metabolomics experts, biological or medical scientists, and data analysts in different research settings, such as contract work of a metabolomics core facility, large third-party funded collaboration projects, *e.g.*, within (transregional) collaborative research centers, or institutional, national, and international research collaborations. MetaboSERV supports common data and metadata exchange formats (Bruker, CSV/TSV, XLSX and YAML, respectively) and is flexible with regards to data formatting, particularly for metadata and phenotypical data. The platform is completely open-source and also offers configurable and portable Docker containers for the purpose of hosting self-managed and self-maintained local MetaboSERV instances in addition to ~~a centralizedMetaboSERV platform~~the centralized, public MetaboSERV instance, hosted at MHH. These characteristics allow a seamless integration of MetaboSERV into local (biomedical) data exchange infrastructures. ~~MetaboSERV~~ The MetaboSERV platform is efficient in terms of speed and flexible in terms of memory usage thanks to Elasticsearch and MariaDB. It is also extensible to suit the needs of different research institutions: both the functionality and the accepted data formats are designed to be adaptable, and MetaboSERV does not require a specific environment apart from a system containing Docker.

A second major feature of ~~MetaboSERV~~ the MetaboSERV platform is to allow users to store and find data suitable for their research projects. ~~Public~~ In the public MetaboSERV instance, public datasets, such as the AKI study or reference concentration values for metabolites, can be retrieved or combined with available data, enabling complex queries. ~~MetaboSERV~~ The MetaboSERV platform also provides visualization options for both raw NMR spectra and summary statistics of absolute metabolite concentrations. While MetaboSERV includes reference concentration ranges for a large number of metabolites from different biofluids provided by the HMDB, arbitrary concentration ranges can likewise be selected. Metadata and phenotypical data for studies and research projects can be kept alongside concentration data and the latter is also available for database queries.

MetaboSERV fills an important gap in the already established metabolomics data repository landscape: it enables interdisciplinary research collaborations to share their metabolomics experimental, phenotypic, as well as metadata within a user friendly platform with fully controlled data access and advanced data browsing and visualization options prior to publication of study results. It provides a user-controlled permission system for uploaded data, which can selectively allow access (both read-only and editorial) to particular data sets for different institutions, researchers and affiliated peers. Self-managed, local MetaboSERV instances can be created by any user employing the fully configurable Docker images, guaranteeing full autonomy as well as data privacy. These features set MetaboSERV apart from the largest and most widely used metabolomics data repositories, MetaboLights, the Metabolomics Workbench, Metabolonote, and MeRy-B, which are designed to share experimental metabolomics data and/or metadata with completely open access [6, 9, 7, 8]. Similar to MetaboLights and the Metabolomics Workbench, MetaboSERV can ~~accomodate~~ accommodate experimental data regardless of species, sample or analytical method, as well as metabolomics metadata. In contrast, Metabolonote is designed to exclusively hold metabolomics metadata [7], and MeRy-B focusses solely on NMR-based metabolomics plant data [8].

Another difference between MetaboSERV and the metabolomics data repositories discussed above is the rich data selection, visualization and querying functionality for absolutely quantified data. This selection process allows the combination of different studies and the integration of already published studies into new research projects. COMETS Analytics offers similar selection features and advanced data analysis tools across different studies, but it is ~~specifically designed~~ designed specifically for standardized meta-analyses of multiple metabolomics studies rather than serving as a metabolomics study repository [11]. Likewise, MetHos focusses on large-scale processing, storage, and analysis of mass spectrometry data, without elaborate, privacy-preserving data sharing options as provided by MetaboSERV [10]. Furthermore, MetaboSERV allows the recording of methodological metadata without any format and/or content restrictions. To facilitate a low-threshold user experience of MetaboSERV, we implemented a very flexible metadata upload by deliberately not enforcing mandatory metadata specifications. In contrast, established metabolomics data repositories such as MetaboLights and the Metabolomics Workbench face the users with rather strict mandatory metadata as well as experimental data upload requirements.

Besides metabolomics-focussed data repositories, a large number of workspaces for data sharing and collaboration with options for privacy preservation have been released in the last decades, including commercial applications like Google Workspace and Nextcloud, as well as a multitude of freely available solutions including Figshare or Synapse/NF Data Portal [48]. Additionally, academic institutions worldwide start building up their own data sharing repositories, e.g., the Academic Cloud service for Lower Saxony, or RepoMed, the institutional repository of Hannover Medical School. However, none of these workspaces and solutions are designed to the specific needs of metabolomics data repositories, but rather provide "data-type agnostic" data lakes for the storage and retrieval of individual data sets [48]. In comparison to MetaboSERV, they do not provide smart search functions for metabolites across several, independent studies, no data analysis or visualization options, and, more importantly, do not support the set-up of self-administered, configurable instances, which are completely independent of the providers. The freely available software FAIRDOM-SEEK [49, 50] can be, similar to MetaboSERV, also deployed locally, however, it is designed particularly for data spanning multiple omics types or interconnecting datasets and systems biology models [49], and not for metabolomics data. Thus, uploaded data sets cannot

be systematically queried or analysed with respect to individual metabolites and/or across studies.

The demands for privacy-preserving data sharing will further increase with technical advances in metabolic fingerprinting of human individuals on the one hand, and ongoing, large-scale rollout of ~~artifical~~ artificial intelligence (AI) for metabolomics data analysis on the other hand. Analytical sensitivity and specificity of metabolic fingerprints will further increase due to technical progress, and will potentially facilitate patient re-identification [15]. AI, in particular, demands large, highly standardized (metabolomics) data sets to ensure maximum performance. MetaboSERV can build the basis for data scientists to access and select multiple metabolomics data sets measured at different metabolomics wet-labs for subsequent AI-based analysis within a privacy-preserving environment. Its open-source architecture allows full user control, adaptability and moreover, seamless integration into already existing research data infrastructures and AI analysis platforms.

Availability of source code and requirements

Project name: MetaboSERV
Project homepage:
https://gitlab.gwdg.de/MedBioinf/metabolomics/metaboserv
Operating system(s): Platform independent
Programming language: Python, R, TypeScript
Other requirements: Docker, Elasticsearch, MariaDB
License: MIT License
RRID: SCR_025496

Minimum installation requirements (recommended):

- 2 cores
- 100GB of hard-drive space (SSD recommended)
- 6GB of RAM

Additional Files

Supplementary Fig. S1. Data model (database schema) of the MetaboSERV Elasticsearch and MariaDB databases.
Supplementary Fig. S2. Exemplary heatmap showing pairwise Pearson's correlation coefficients between different metabolites in the AKI plasma study.
Supplementary Fig. S3. Exemplary quality control plot for the Biocrates ~~Test~~ test data discussed in Use Case 2.
Supplementary Fig. S4. Exemplary query to the public MetaboSERV database discussed in Use Case 3.
Supplementary Fig. S5. Part of the query results of Use Case 3.
Supplementary Fig. S6. Scatter plots of Use Case 3.
Supplementary Table S1. List of all programming languages and packages used to implement MetaboSERV, as well as their respective versions.
Supplementary File S1. Detailed description of MetaboSERV data model.
Supplementary File S2. Details on user authentication and password storage.
Supplementary File S3. MetaboSERV file specifications.
Supplementary File S4. Use case 2: Quality assessment of mass spectrometry data.
Supplementary File S5. Use case 3: Large-scale, multi-modal, untargeted mass spectrometry data.
Supplementary File S6. Detailed installation and user guide for the set-up of local MetaboSERV instances.

Declarations

List of abbreviations

AI - artificial intelligence
AKI - acute kidney injury
API - application programming interface
CPB - cardiopulmonary bypass
CRUD - create, read, update, delete
DSL - domain-specific language
FAIR - findable, accessible, interoperable, reusable
FID - free induction decay
GDPR - General Data Protection Regulation
HMDB - Human Metabolome Database
JWT - JSON web token
LC-MS - liquid chromatography – mass spectrometry
LOD - limit of detection
MeRy-B - Metabolomics Repository Bordeaux
MHH - Hannover Medical School
NMR - nuclear magnetic resonance
RRID - Research Resource Identifier

Ethical Approval ~~(optional)~~

NMR data of the AKI use case had been previously collected with written informed patient consent upon ethical approval from the University Clinic Erlangen.

Consent for publication

All metabolomics and phenotypic data used is completely anonymized. No further consent is required.

Competing Interests

The authors declare that they have no competing interests.

Funding

This work was supported by the German Federal Ministry of Education and Research (BMBF) within the framework of the e:Med research and funding concept (grant numbers: 01ZX1912A, 01ZX1912C, and 01ZX1912D).

Author's Contributions

TT Software, Writing - Original Draft Preparation, Methodology, Visualization; AM ~~Conceptualization and~~ Software, Conceptualization, Validation; YNN Validation; SS Conceptualization; MG Resources; FK Resources; KD Resources; PJO Conceptualization and Resources; WG Conceptualization and Resources; MA Conceptualization and Supervision; JD Conceptualization, Methodology, and Supervision; HZ Conceptualization, Writing - Original Draft Preparation, Methodology, Funding Acquisition, Resources, Formal Analysis; all Writing - Review & Editing of the manuscript.

~~Acknowledgements~~ Acknowledgments

~~Not applicable~~ The authors are grateful to Mr. Norman Schönfeld (MHH Information Technology, MHH) and Mr. Merlin-Puck Rietschel (Peter L. Reichertz Institute for Medical Informatics, MHH) for IT advise and support.

References

1. Clish CB. Metabolomics: an emerging but powerful tool for precision medicine. Cold Spring Harbor Molecular Case Studies 2015 Oct;1(1):a000588. https://www.ncbi.nlm.nih.gov/pmc/articles/PMC4850886/.
2. Emwas AHM, Salek RM, Griffin JL, Merzaban J. NMR-based metabolomics in human disease diagnosis: applications, limitations, and recommendations. Metabolomics 2013 Oct;9(5):1048–1072. https://doi.org/10.1007/s11306-013-0524-y.
3. Zacharias HU, Altenbuchinger M, Gronwald W. Statistical Analysis of NMR Metabolic Fingerprints: Established Methods and Recent Advances. Metabolites 2018 Sep;8(3):47. https://www.mdpi.com/2218-1989/8/3/47, number: 3 Publisher: Multidisciplinary Digital Publishing Institute.
4. Emwas AH, Roy R, McKay RT, Tenori L, Saccenti E, Gowda GAN, et al. NMR Spectroscopy for Metabolomics Research. Metabolites 2019 Jul;9(7):123. https://www.mdpi.com/2218-1989/9/7/123, number: 7 Publisher: Multidisciplinary Digital Publishing Institute.
5. Zacharias HU, Kaleta C, Cossais F, Schaeffer E, Berndt H, Best L, et al. Microbiome and Metabolome Insights into the Role of the Gastrointestinal–Brain Axis in Parkinson's and Alzheimer's Disease: Unveiling Potential Therapeutic Targets. Metabolites 2022 Dec;12(12):1222. https://www.mdpi.com/2218-1989/12/12/1222, number: 12 Publisher: Multidisciplinary Digital Publishing Institute.
6. Haug K, Salek RM, Conesa P, Hastings J, de Matos P, Rijnbeek ML, et al. MetaboLights—an open-access general-purpose repository for metabolomics studies and associated meta-data. Nucleic Acids Research 2013 Jan;41(Database issue:D781–D786. https://www.scinapse.io/papers/2069928158.
7. Ara T, Enomoto M, Arita M, Ikeda C, Kera K, Yamada M, et al. Metabolonote: A Wiki-Based Database for Managing Hierarchical Metadata of Metabolome Analyses. Frontiers in Bioengineering and Biotechnology 2015 Apr;3:38. https://www.ncbi.nlm.nih.gov/pmc/articles/PMC4388006/.
8. Ferry-Dumazet H, Gil L, Deborde C, Moing A, Bernillon S, Rolin D, et al. MeRy-B: a web knowledgebase for the storage, visualization, analysis and annotation of plant NMR metabolomic profiles. BMC Plant Biology 2011 Jun;11:104. https://www.ncbi.nlm.nih.gov/pmc/articles/PMC3141636/.
9. Sud M, Fahy E, Cotter D, Azam K, Vadivelu I, Burant C, et al. Metabolomics Workbench: An international repository for metabolomics data and metadata, metabolite standards, protocols, tutorials and training, and analysis tools. Nucleic Acids Research 2016 Jan;44(D1):D463–D470. https://doi.org/10.1093/nar/gkv1042.
10. Tzanakis K, Nattkemper TW, Niehaus K, Albaum SP. MetHoS: a platform for large-scale processing, storage and analysis of metabolomics data. BMC Bioinformatics 2022 Jul;23(1):267. https://doi.org/10.1186/s12859-022-04793-w.
11. Temprosa M, Moore SC, Zanetti KA, Appel N, Ruggieri D, Mazzilli KM, et al. COMETS Analytics: An Online Tool for Analyzing and Meta-Analyzing Metabolomics Data in Large Research Consortia. American Journal of Epidemiology 2022 Jan;191(1):147–158. https://research.wur.nl/en/publications/comets-analytics-an-online-tool-for-analyzing-and-meta-analyzing, publisher: Oxford University Press.
12. Haug K, Cochrane K, Nainala VC, Williams M, Chang J, Jayaseelan KV, et al. MetaboLights: a resource evolving in response to the needs of its scientific community. Nucleic Acids Research 2020 Jan;48(D1):D440–D444. https://doi.org/10.1093/nar/gkz1019.
13. Wilkinson MD, Dumontier M, Aalbersberg IJ, Appleton G, Axton M, Baak A, et al. The FAIR Guiding Principles for scientific data management and stewardship. Scientific Data 2016 Mar;3(1):160018. https://www.nature.com/articles/sdata201618, number: 1 Publisher: Nature Publishing Group.
14. Powell CD, Moseley HNB. The Metabolomics Workbench File Status Website: A Metadata Repository Promoting FAIR Principles of Metabolomics Data. BMC Bioinformatics 2023 Jul;24(24):299. https://bmcbioinformatics.biomedcentral.com/articles/10.1186/s12859-023-05423-9.
15. Keane TM, O'Donovan C, Vizcaíno JA. The growing need for controlled data access models in clinical proteomics and metabolomics. Nature Communications 2021 Oct;12(1):5787. https://www.nature.com/articles/s41467-021-26110-4, number: 1 Publisher: Nature Publishing Group.
16. Elasticsearch: The Official Distributed Search & Analytics Engine;. https://www.elastic.co/de/elasticsearch.
17. Python to MariaDB Connector; 2020. https://mariadb.com/resources/blog/how-to-connect-python-programs-to-mariadb/.
18. Wishart DS, Guo A, Oler E, Wang F, Anjum A, Peters H, et al. HMDB 5.0: the Human Metabolome Database for 2022. Nucleic Acids Research 2022 Jan;50(D1):D622–D631.
19. elasticsearch: Python client for Elasticsearch;. https://github.com/elastic/elasticsearch-py.
20. Vue.js - The Progressive JavaScript Framework | Vue.js;. https://vuejs.org/.
21. Bierman G, Abadi M, Torgersen M. Understanding TypeScript. In: Jones R, editor. ECOOP 2014 – Object-Oriented Programming Lecture Notes in Computer Science, Berlin, Heidelberg: Springer; 2014. p. 257–281.
22. TypeScript: JavaScript With Syntax For Types.;. https://www.typescriptlang.org/.
23. Pinia | The intuitive store for Vue.js;. https://pinia.vuejs.org.
24. Angular Data Grid: Documentation;. https://www.ag-grid.com/angular-data-grid/.
25. The official home of the Python Programming Language; 2023. https://www.python.org/.
26. Grinberg M. Flask Web Development: Developing Web Applications with Python. 1st ed. O'Reilly Media, Inc.; 2014.
27. Harris CR, Millman KJ, van der Walt SJ, Gommers R, Virtanen P, Cournapeau D, et al. Array programming with NumPy. Nature 2020 Sep;585(7825):357–362. https://www.nature.com/articles/s41586-020-2649-2, number: 7825 Publisher: Nature Publishing Group.
28. McKinney W. Data Structures for Statistical Computing in Python. Austin, Texas; 2010. p. 56–61. https://conference.scipy.org/proceedings/scipy2010/mckinney.html.
29. Hunter JD. Matplotlib: A 2D Graphics Environment. Computing in Science & Engineering 2007 May;9(3):90–95. Conference Name: Computing in Science & Engineering.
30. Waskom ML. seaborn: statistical data visualization. Journal of Open Source Software 2021 Apr;6(60):3021. https://joss.theoj.org/papers/10.21105/joss.03021.
31. xmltodict: Makes working with XML feel like you are working with JSON;. https://github.com/martinblech/xmltodict.
32. Flask-Cors: A Flask extension adding a decorator for CORS support;. https://github.com/corydolphin/flask-cors.
33. flask-swagger-ui: Swagger UI blueprint for Flask;. https://github.com/sveint/flask-swagger-ui.
34. Flask-JWT-Extended: Extended JWT integration with Flask;. https://github.com/vimalloc/flask-jwt-extended.
35. Werkzeug: The comprehensive WSGI web application library.;. https://palletsprojects.com/p/werkzeug/.
36. Gunicorn - WSGI Server;. https://docs.gunicorn.org/en/stable/.
37. Hupp A, python-magic; 2023. https://github.com/ahupp/python-magic.
38. R Core Team. R: A Language and Environment for Statistical

Computing. R Foundation for Statistical Computing, Vienna, Austria; 2022, https://www.R-project.org/.
39. RestRserve: A Framework for Building HTTP API;. https://restrserve.org/.
40. Klein MS. Affine Transformation of Negative Values for NMR Metabolomics Using the mrbin R Package. Journal of Proteome Research 2021 Feb;20(2):1397–1404. https://doi.org/10.1021/acs.jproteome.0c00684.
41. Zacharias HU, Schley G, Hochrein J, Klein MS, Köberle C, Eckardt KU, et al. Analysis of human urine reveals metabolic changes related to the development of acute kidney injury following cardiac surgery. Metabolomics 2013 Jun;9(3):697–707. https://doi.org/10.1007/s11306-012-0479-4.
42. Zacharias HU, Hochrein J, Vogl FC, Schley G, Mayer F, Jeleazcov C, et al. Identification of Plasma Metabolites Prognostic of Acute Kidney Injury after Cardiac Surgery with Cardiopulmonary Bypass. Journal of Proteome Research 2015 Jul;14(7):2897–2905.
43. Fino NF, Adingwupu OM, Coresh J, Greene T, Haaland B, Shlipak MG, et al. Evaluation of novel candidate filtration markers from a global metabolomic discovery for glomerular filtration rate estimation. Kidney international 2024;105(3):582–592.
44. Scalability and resilience: clusters, nodes, and shards | Elasticsearch Guide [8.10] | Elastic; 2023. https://www.elastic.co/guide/en/elasticsearch/reference/current/scalability.html.
45. Sreekumar D, Trends in research collaborations: An overview | Researcher.Life; 2022. https://researcher.life/blog/article/trends-in-research-collaborations/.
46. Jones M, Bradley J, Sakimura N. JSON Web Token (JWT). Internet Engineering Task Force; 2015.
47. Kellum JA, Lameire N, Aspelin P, Barsoum RS, Burdmann EA, Goldstein SL, et al. Kidney disease: improving global outcomes (KDIGO) acute kidney injury work group. KDIGO clinical practice guideline for acute kidney injury. Kidney international supplements 2012;2(1):1–138.
48. Allaway RJ, La Rosa S, Verma S, Mangravite L, Guinney J, Blakeley J, et al. Engaging a community to enable disease-centric data sharing with the NF Data Portal. Scientific data 2019;6(1):319.
49. Wolstencroft K, Owen S, Krebs O, Nguyen Q, Stanford NJ, Golebiewski M, et al. SEEK: a systems biology data and model management platform. BMC systems biology 2015;9:1–12.
50. Wolstencroft K, Krebs O, Snoep JL, Stanford NJ, Bacall F, Golebiewski M, et al. FAIRDOMHub: a repository and collaboration environment for sharing systems biology research. Nucleic acids research 2017;45(D1):D404–D407.

Figure 1

Click here to access/download;Figure;fig1.png

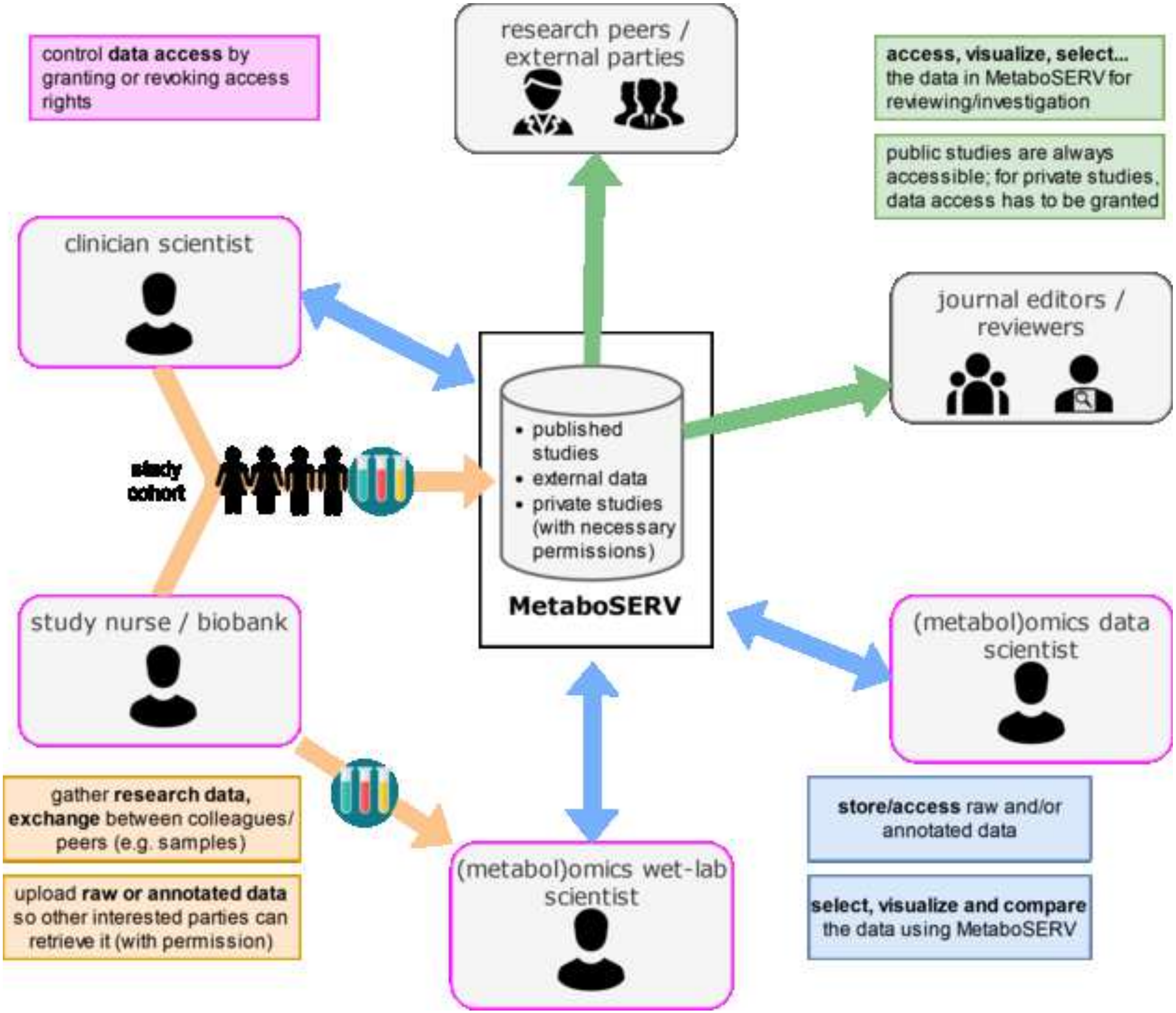

Figure 2 - revised

Click here to access/download;Figure;fig2.png

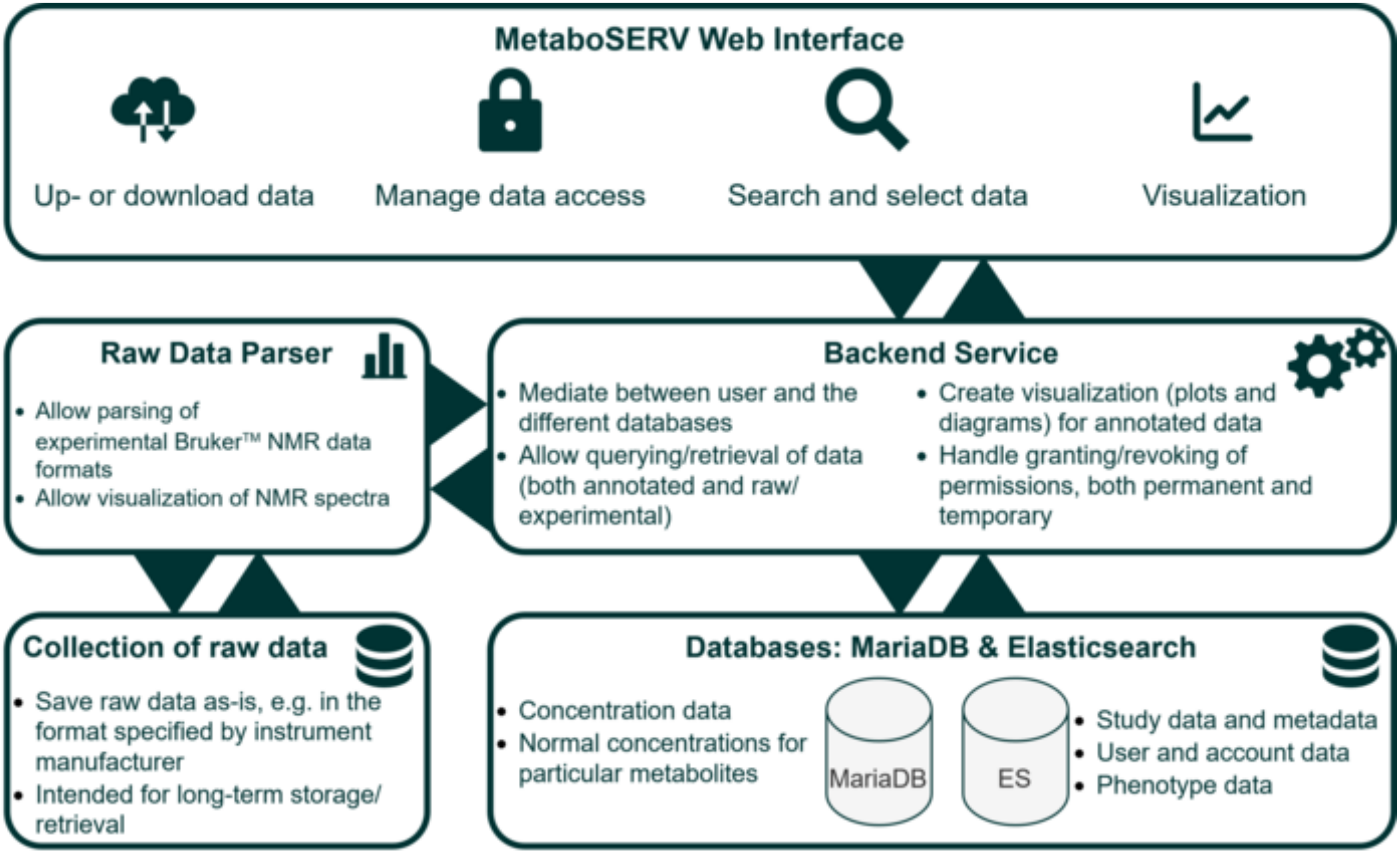

Figure 3 - revised

Click here to access/download;Figure;fig3.png

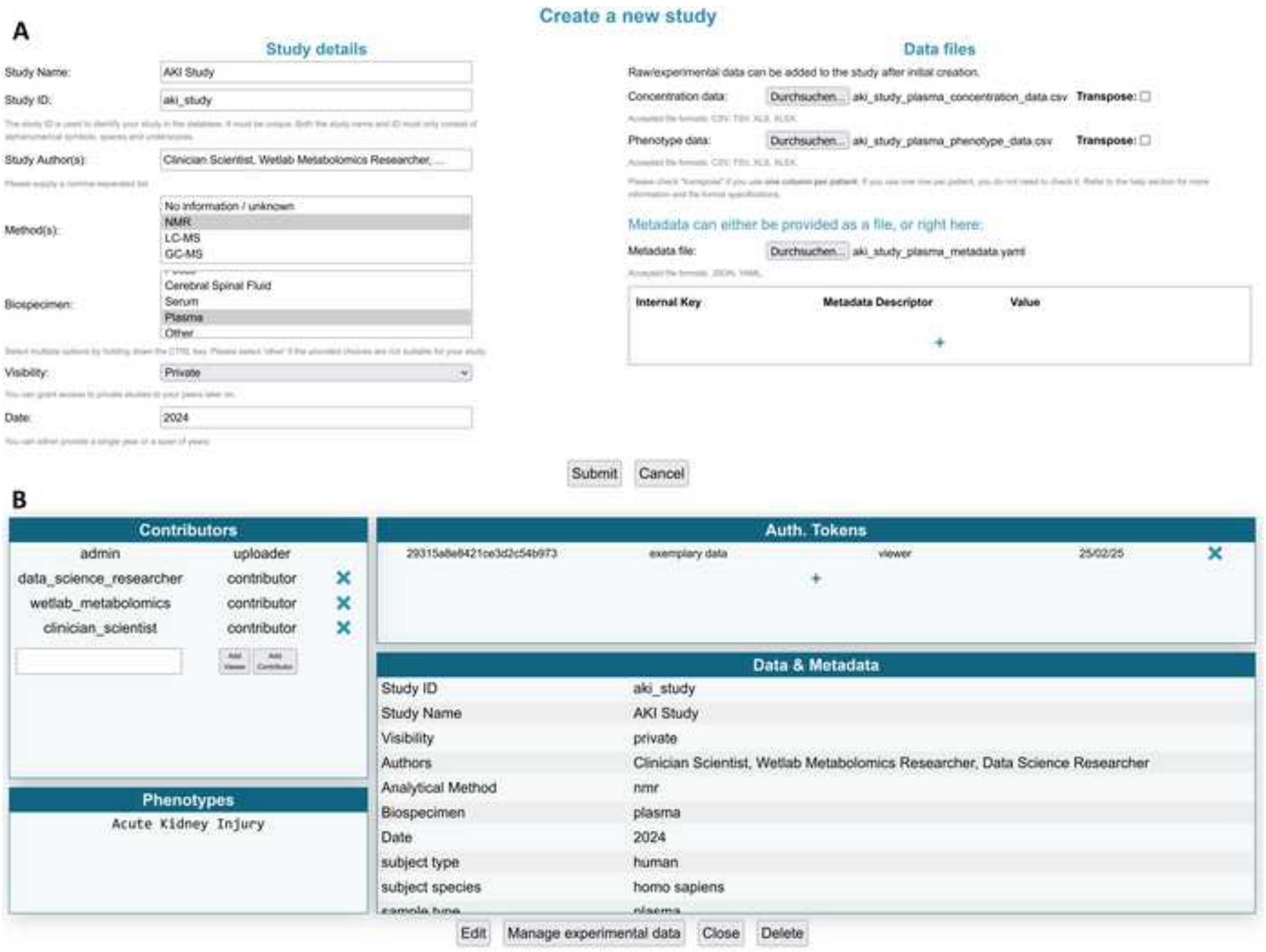

Figure 4

Click here to access/download;Figure;fig4.png

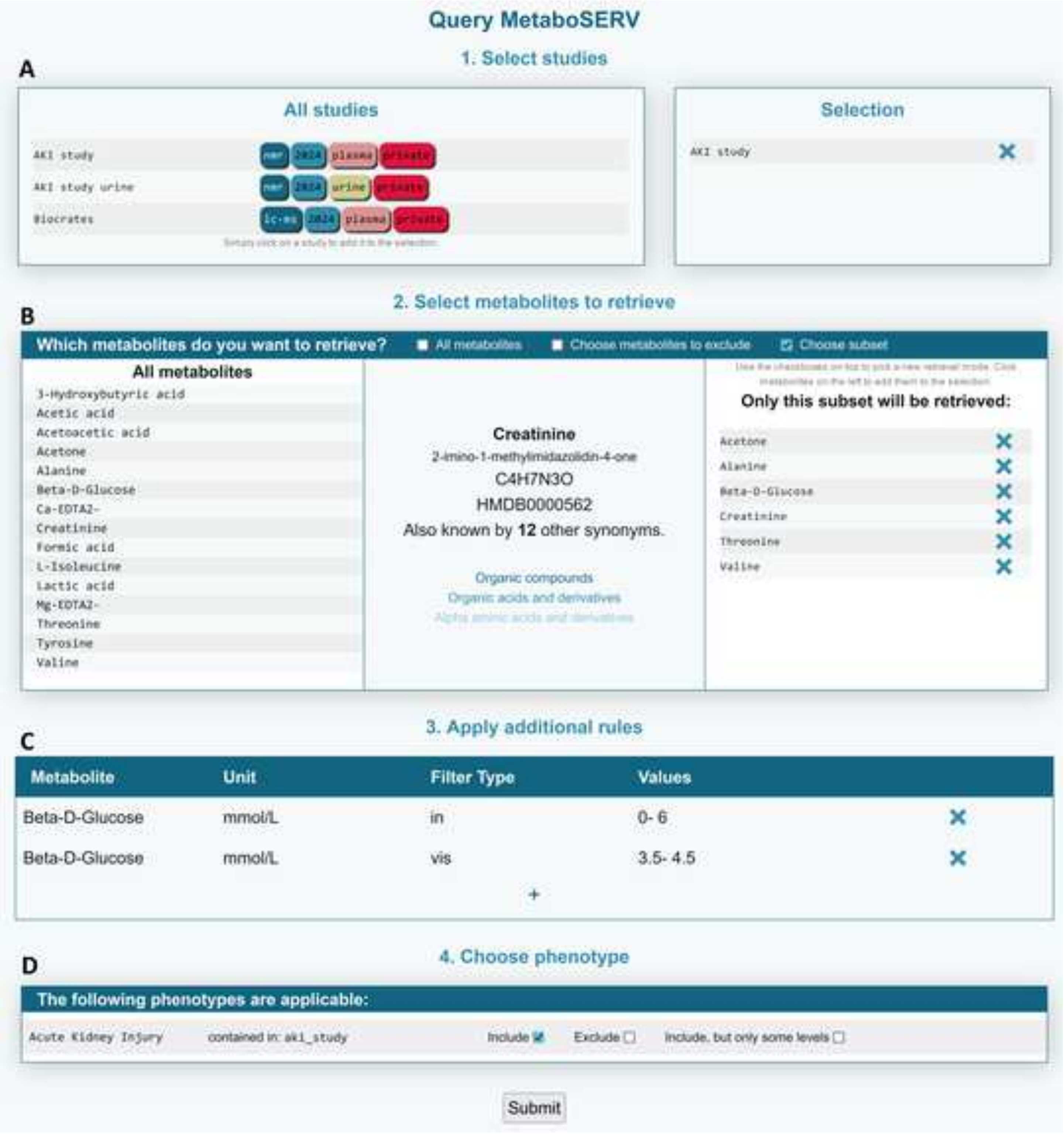

Figure 5 - revised

Click here to access/download;Figure;fig5.png

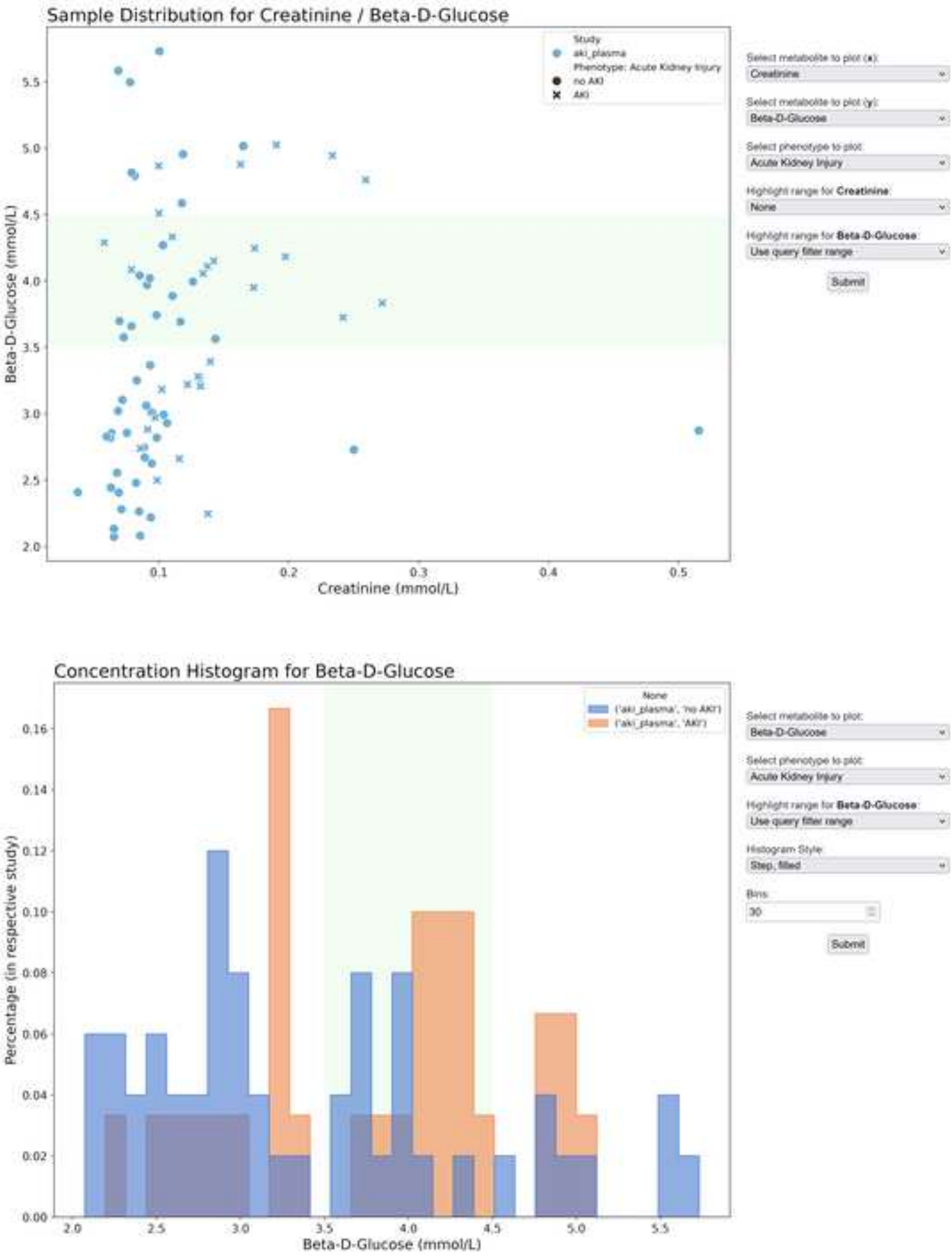

Figure 6

Click here to access/download;Figure;fig6.png

Source ID	Study	Acute Kidney Injury	Acetone	Alanine	Beta-D-Glucose ↓	Creatinine	Threonine
AKI_51	aki_study	no AKI	1.91072 mmol/L	0.103263 mmol/L	5.73048 mmol/L	0.100439 mmol/L	
AKI_19	aki_study	no AKI	0.357399 mmol/L	0.16642 mmol/L	5.58404 mmol/L	0.0687475 mmol/L	
AKI_20	aki_study	no AKI	0.144835 mmol/L	0.153249 mmol/L	5.49683 mmol/L	0.0778023 mmol/L	
AKI_18	aki_study	AKI	0.0228864 mmol/L	0.417078 mmol/L	5.02584 mmol/L	0.190397 mmol/L	0.116662 mmol/L
AKI_55	aki_study	no AKI	0.0268556 mmol/L	0.241009 mmol/L	5.01733 mmol/L	0.164905 mmol/L	0.0530378 mmol/L
AKI_95	aki_study	no AKI	0.316609 mmol/L	0.193356 mmol/L	4.95481 mmol/L	0.118549 mmol/L	0.0914211 mmol/L
AKI_06	aki_study	AKI	0.0806513 mmol/L	0.24828 mmol/L	4.94507 mmol/L	0.233604 mmol/L	
AKI_94	aki_study	AKI	0.0834382 mmol/L	0.254088 mmol/L	4.87885 mmol/L	0.162888 mmol/L	0.0780183 mmol/L
AKI_46	aki_study	AKI	0.118823 mmol/L	0.169484 mmol/L	4.8676 mmol/L	0.0998487 mmol/L	0.0759844 mmol/L
AKI_59	aki_study	no AKI	0.0574271 mmol/L	0.217274 mmol/L	4.81593 mmol/L	0.0789833 mmol/L	0.10618 mmol/L
AKI_104	aki_study	no AKI	0.0211974 mmol/L	0.363983 mmol/L	4.79278 mmol/L	0.0814931 mmol/L	0.0971578 mmol/L

1 to 11 of 80 | Page 1 of 8

Figure S1

Click here to access/download;Figure;fig_s1.png

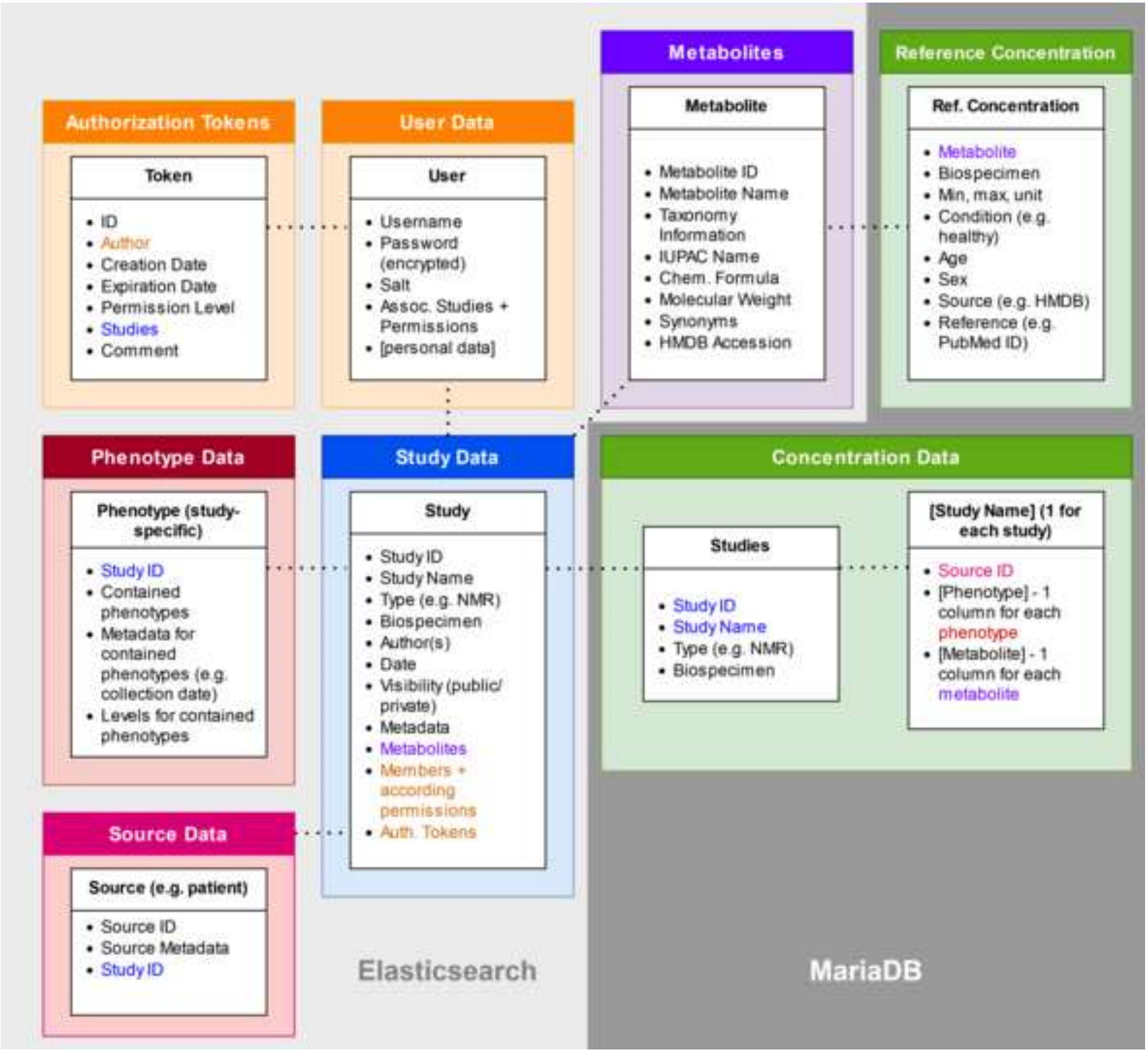

Figure S2

Click here to access/download;Figure;fig_s2.png

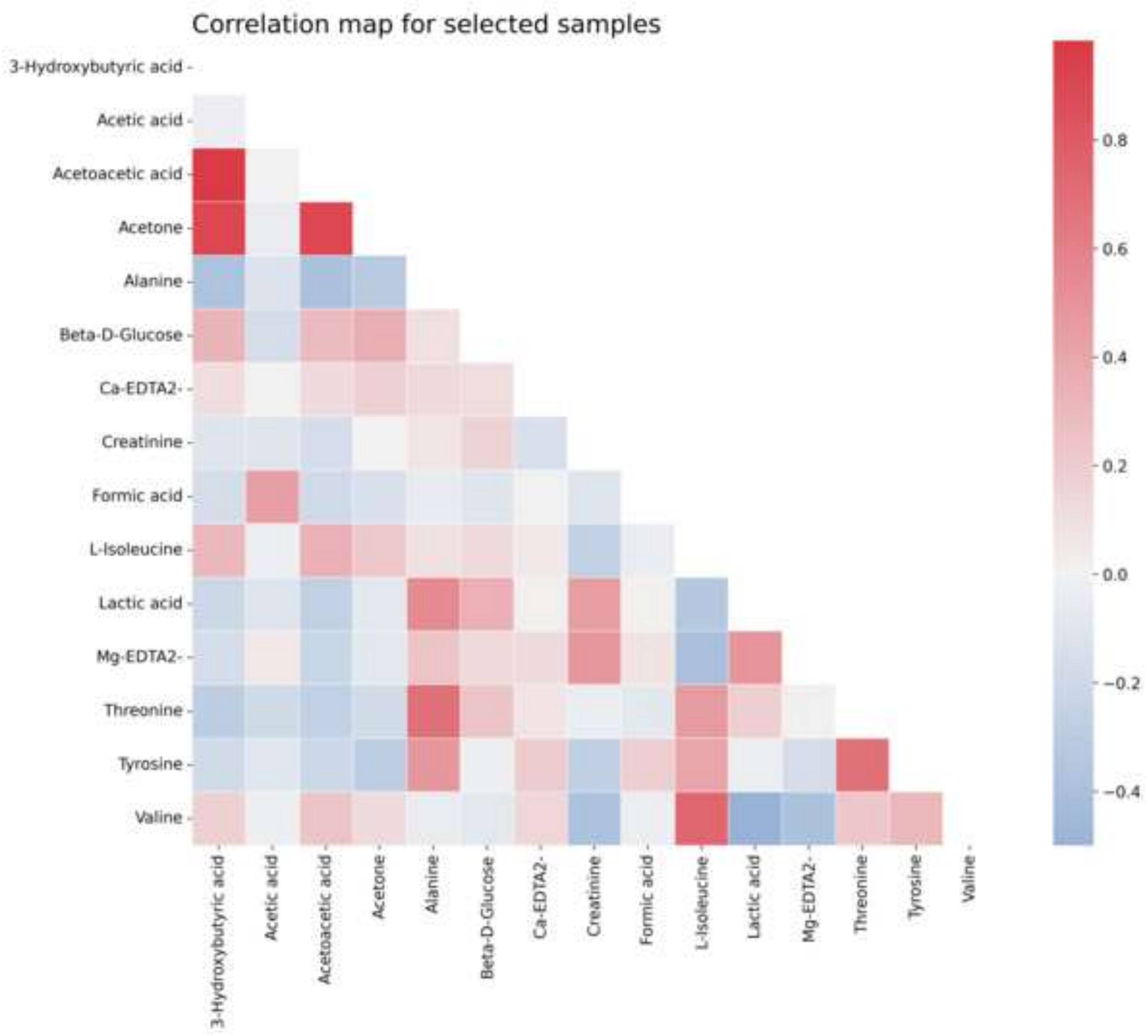

Figure S3

Click here to access/download;Figure;fig_s3.png

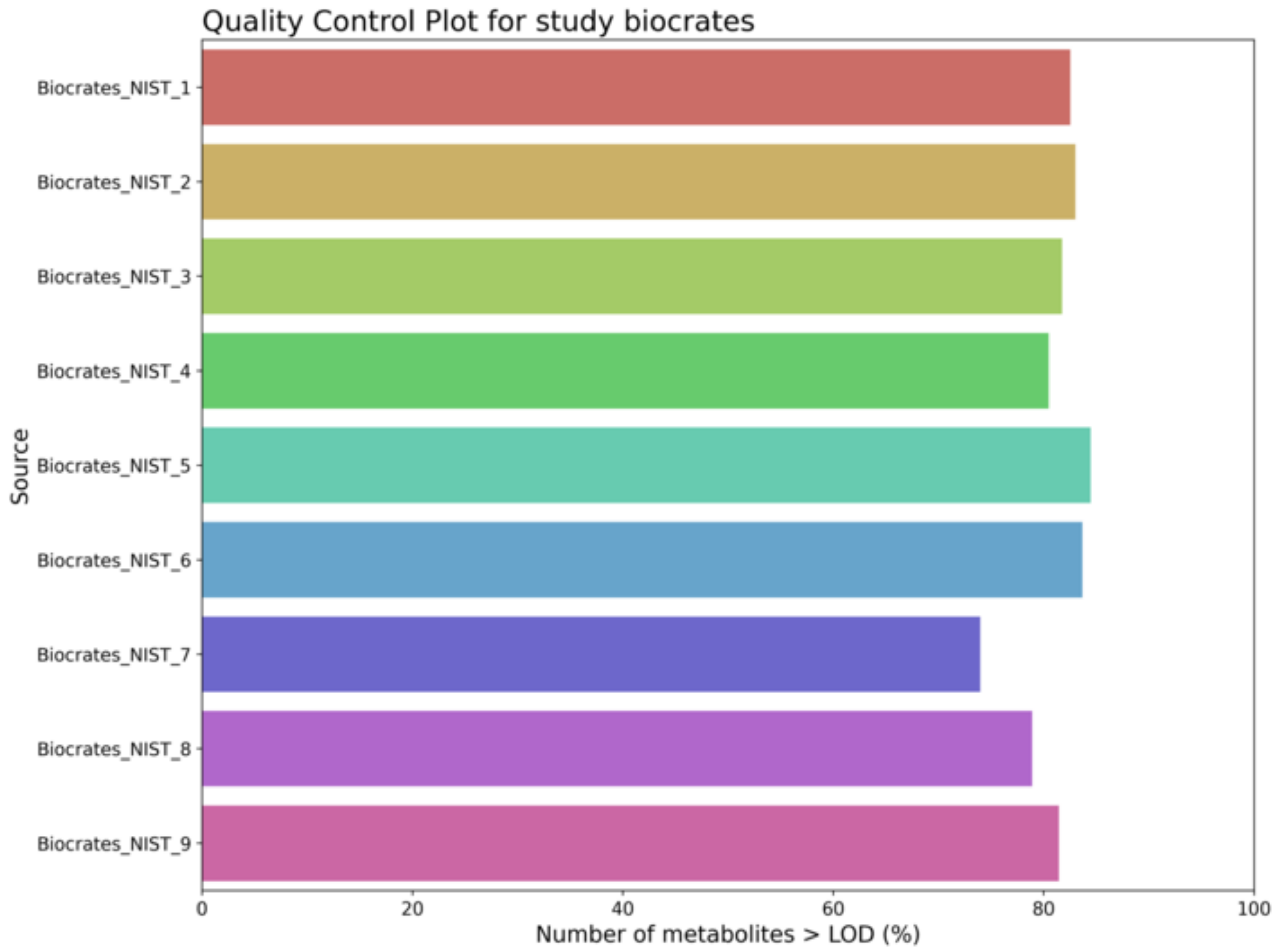

Figure S4

Click here to access/download;Figure;fig_s4.png

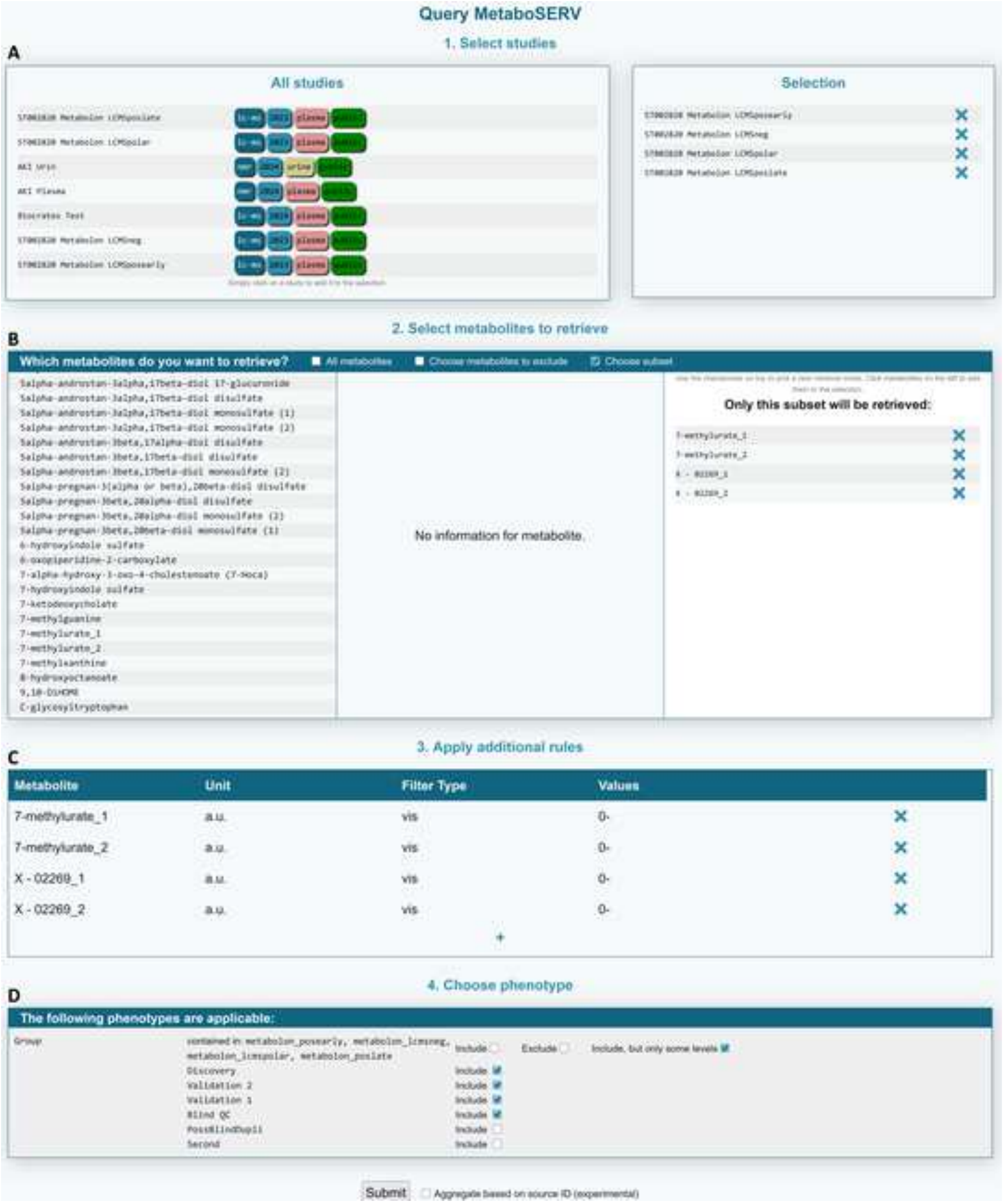

Figure S5

Click here to access/download;Figure;fig_s5.png

Source ID	Study	7-methylurate_1	7-methylurate_2	X - 02269_1	X - 02269_2	Group ↑
META-96843	metabolon_posearly		63361.797 a.u.		6569484.5 a.u.	Discovery
META-96845	metabolon_posearly				723525.5 a.u.	Discovery
META-96846	metabolon_posearly				1073004.625 a.u.	Discovery
META-96848	metabolon_posearly		66113.367 a.u.		1207107.375 a.u.	Discovery
META-96849	metabolon_posearly		177703.328 a.u.		2262345.75 a.u.	Discovery
META-96850	metabolon_posearly		190429.125 a.u.		4509315 a.u.	Discovery
META-95838	metabolon_lcmsneg			14756424 a.u.		Discovery
META-95842	metabolon_lcmsneg			13849085 a.u.		Discovery
META-95843	metabolon_lcmsneg			3702497 a.u.		Discovery
META-95844	metabolon_lcmsneg	1046539.25 a.u.		693650.688 a.u.		Discovery
META-95848	metabolon_lcmsneg	79322.922 a.u.		5532982.5 a.u.		Discovery

551 to 561 of 2,000 | Page 51 of 182

Figure S6A

Click here to access/download;Figure;fig_s6A.png

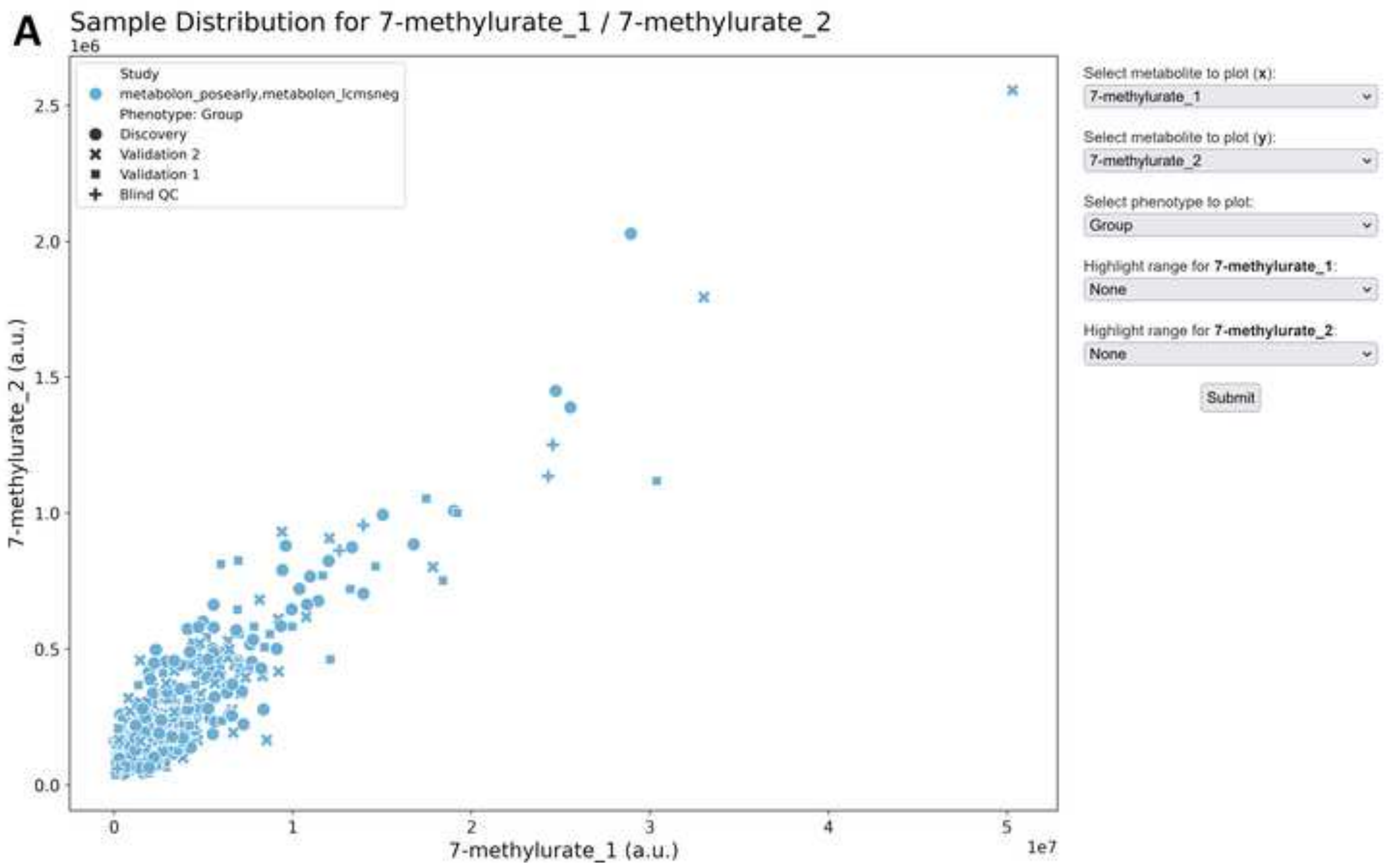

Figure S6B Click here to access/download;Figure;fig_s6B.png

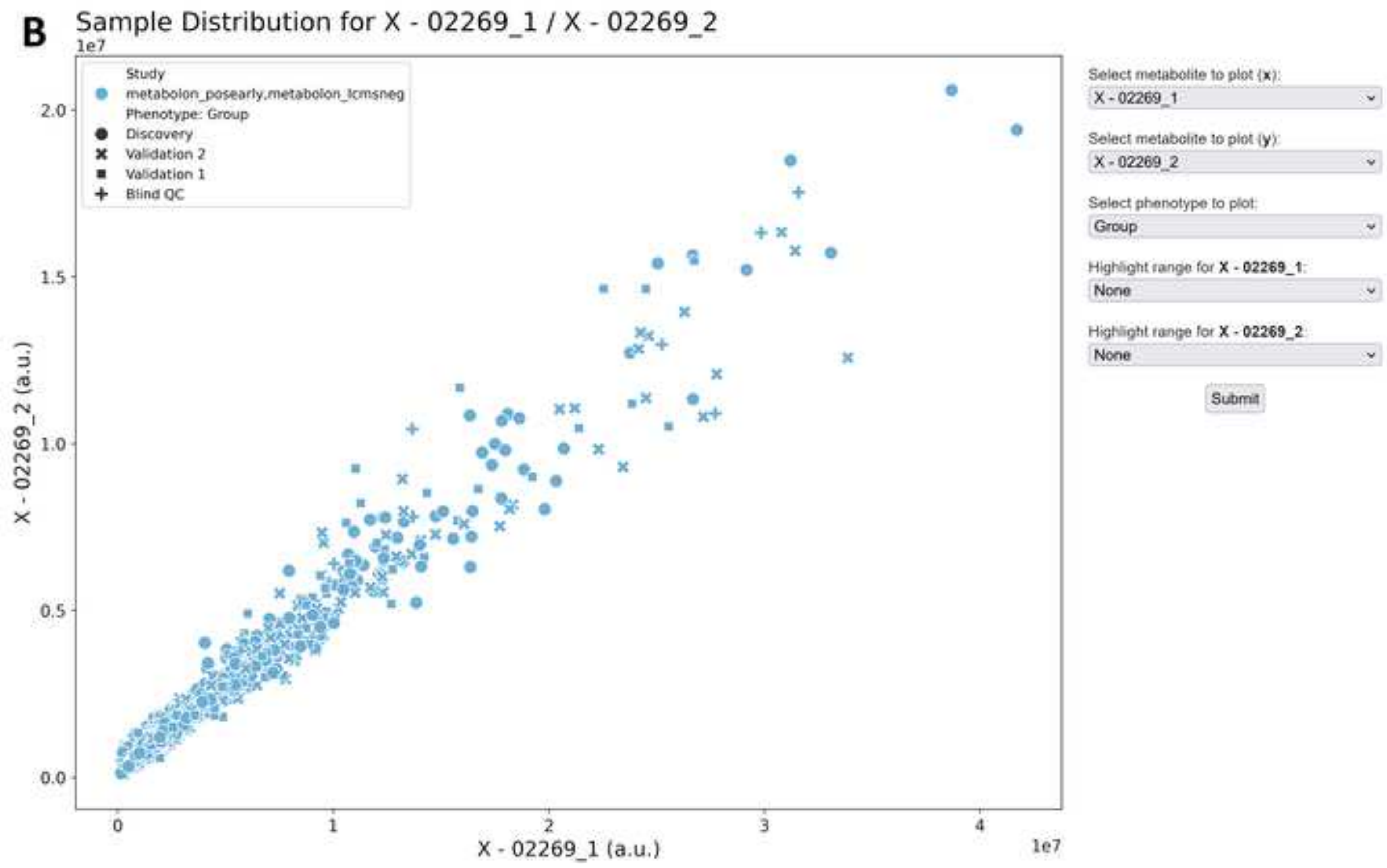

Click here to access/download
Supplementary Material
MetaboSERV_Supplement.pdf

Supplementary Material_tracked changes

Click here to access/download
Supplementary Material
MetaboSERV_Supplement_diff.pdf
