# Supplementary material for: MetaboSERV—a platform for selecting, exchanging, and visualizing metabolomics data with controlled data access
Source: Gigascience. 2025 Aug 1;14:giaf075. doi: 10.1093/gigascience/giaf075 (PMC12315529; doi:10.1093/gigascience/giaf075)
Supplement: giaf075_Authors_Response_To_Reviewer_Comments_Original_Submission [file giaf075_authors_response_to_reviewer_comments_original_submission.pdf]

Author's Response To Reviewer Comments

Dear Dr. Zauner,

we are very thankful for the opportunity to submit a revised version of our manuscript "MetaboSERV - a platform for selecting, exchanging, and visualizing metabolomics data with controlled data access" (GIGA-D-24-00275) to GigaScience. In particular, we would like to thank you and the reviewers for the elaborate assessment of our manuscript and the fruitful comments, which we carefully address in a point-by-point reply below.

As you have particularly highlighted the reviewers' comments on data privacy, we would like to briefly elaborate on our efforts in this regards:

We fully agree with you and the reviewers that secure data handling is an important point which affects the project in different aspects, in particular (1) the operation of the public MetaboSERV instance, (2) the general architectural design and implementation of the MetaboSERV platform, and (3) the operation of local MetaboSERV instances.

The public instance of MetaboSERV, as described in the manuscript, is hosted in the computing center of the Hannover Medical School, Germany (MHH). MHH's regulations for access control to the server, security updates, backup and monitoring are implemented following the ISO 27001 and the standards of the German Federal Office for Information Security (Bundesamt für Sicherheit in der Informationstechnik, BSI), are focussed on handling sensitive clinical data, and therefore overfulfill the requirements for MetaboSERV. In particular, the public MetaboSERV server at MHH and the data stored there can only be accessed by authorized administrators. Server access is continuously logged and regularly inspected. The virtual machine is equipped with the latest security updates and regular backups are being taken every six hours.

Following GDPR regulations, the amount of personal data stored in MetaboSERV is minimized. As explicitly stated in the "Data privacy" statement on the public MetaboSERV instance, data uploaded to MetaboSERV should always be pseudonymized and contain no information to identify study participants. To create and manage MetaboSERV accounts, a minimum amount of personal information (username, e-mail address, and optionally institution) is requested and the necessity for their storage is explained in the "Data privacy" statement. Except for the e-mail address, no provided information is verified, as it mainly has the purpose to facilitate the permission structure of MetaboSERV. The user is free to choose a username with no link to their real-world identity. Upon account creation, the accounts are initially verified by an automated e-mail verification system and unauthorized access attempts are logged and reported back to the user (for more details on e-mail verification as well as access control, see our response to reviewer 1, comment 4 and reviewer 2, comment Q1). Passwords are only stored in an encrypted manner (hashed and salted). The risk of data loss is reduced by organisational measures and the effect of a data loss is minimized by encryption of the passwords.

For local installations and operations of MetaboSERV instances, the user remains fully responsible for the security of the installation, e.g., that server access is controlled or data is not forwarded to third-parties. We have now created a video tutorial on the installation and set-up of local MetaboSERV instances, available at https://www.youtube.com/watch?v=vbqY2qJcgLk, where we also explicitly highlight steps during the installation set-up addressing vital data privacy aspects.

In the manuscript, we added a new subsection "Data privacy and security" at the end of the "Methods: Implementation" section, describing the GDPR-conform data handling in MetaboSERV. In the subsection "Results: MetaboSERV server environment", the secure hosting of the public MetaboSERV instance is described. Extended descriptions are provided in Supplementary File S2, "User Authentication and Password Storage".

You will find our complete point-by-point reply to the reviewer comments below.

There are no actual or potential conflicts of interest for any of the authors involved in this study. The results of this manuscript have not been published elsewhere, nor are they under consideration at any other journal. The manuscript has been read and approved by all authors. All the persons listed as co-authors have qualified for authorship and have approved the manuscript for submission.

We would like to thank you once again for your time reviewing this submission. Please contact us (Prof. Dr. Helena Zacharias: Zacharias.Helena@mh-hannover.de; Dr. Juergen Doenitz: juergen.doenitz@bioinf.med.uni-goettingen.de) in case any questions might arise.

We are looking forward to hearing from you.

Best regards,
Tim Tucholski, Helena Zacharias, and Juergen Doenitz

--- Reviewer reports ---

--- Reviewer 1 ---

The manuscript "MetaboSERV - platform for selecting, exchanging, and visualizing metabolomics data with controlled data access" presents an ambitious resource for metabolomics research which aims to provide a collaborative workspace tool designed for team-driven research efforts. The technical note emphasizes the privacy-preserving functionality of MetaboSERV and offers selective curation tools to evaluate multiple experimental outcomes simultaneously. The platform presented in this paper has the potential to be a valuable tool to research teams, but this reviewer was confused on what role the proposed platform serves in a broader scientific context. Below is a list of issues that should be addressed before this manuscript is suitable for publication:

Response by authors:

We are very grateful to the reviewer for his/her appreciation as well as detailed comments, and we have carefully tried to address the reviewer's concerns as outlined below.

-- Major Issues --

1. MetaboSERV is regularly compared to existing public metabolomics data repositories, but it was unclear if it is a public repository itself. The public instance of MetaboSERV only contains the example results outlined in this manuscript and none of the raw data from those experiments was available for download from the public instance. Additionally, this reviewer was unable to upload raw experimental data to the public instance on their own account. Is the public instance of MetaboSERV intended for end users or just a demo of a potential private instance? The manuscript is so focused on privacy and team collaboration, it is entirely unclear if this platform serves any functionality as a public data repository. As the introduction and discussion sections of this manuscript heavily compare MetaboSERV to existing public data repositories, the authors need to disambiguate this potential source of confusion.

Response by authors:

We are very thankful to the reviewer for pointing out this potential confusion. The public MetaboSERV instance is indeed not only a demo, but is intended as a fully functioning platform freely available to end-users. During our first submission, the data upload was disabled. We have now removed these restrictions and both data upload and download is now enabled for registered users. In order to disambiguate the intended role of the public MetaboSERV instance, we have carefully rephrased the complete manuscript and supplement: we now persistently differentiate between the "public MetaboSERV instance", hosted at MHH, and "local MetaboSERV instances", which are autonomously set-up at the end-user's institutions. Furthermore, we consistently use the term "MetaboSERV platform" (or simply "MetaboSERV") in case we describe general functionalities, which are valid for both the public and any local MetaboSERV instances. Furthermore, we completely revised the following section:

"Results: MetaboSERV server environment
The public MetaboSERV instance, available at https://metaboserv.ckdn.app, is hosted at the computing center of the Hannover Medical School, Germany (MHH). MHH's regulations for access control to the server, security updates, backup and monitoring are implemented following the ISO 27001 and the standards of the German Federal Office for Information Security (Bundesamt für Sicherheit in der Informationstechnik, BSI). In particular, the public MetaboSERV server at MHH and the data stored there can only be accessed by authorized administrators. Server access is continuously logged and regularly inspected. The virtual machine is equipped with the latest security updates and regular backups are being taken every six hours.

Local MetaboSERV instances can be set up by cloning our repositories at https://gitlab.gwdg.de/MedBioinf/metabolomics/metaboserv and creating, configuring, and running the respective Docker images. Detailed user guides are provided in Supplementary File S6: "Detailed installation and user guide for the set-up of local MetaboSERV instances" as well as at https://metaboserv.ckdn.app/guide and in our GitLab repositories. MetaboSERV is generally resource-friendly, all core components can be set up on a dual-core machine with 6 GB of random access memory (RAM) and 200 GB of hard disk space for MariaDB and Elasticsearch. Furthermore, a sufficient amount of hard disk space is necessary to store raw metabolomics data. Query speed is heavily dependent on the resources attributed to the underlying Elasticsearch and MariaDB instances. Therefore, it is recommended to set up MetaboSERV on a machine with at least four cores and 16 GB of RAM and to take advantage of Elasticsearch's sharding mechanism [44]. By default, MetaboSERV makes no assumptions about the Elasticsearch environment to avoid structural and capacity-related issues. These self-managed MetaboSERV instances run isolated from the public MetaboSERV instance and allow hosting and managing data on self-governed servers, removing any further data privacy concerns. They can also be altered and configured to fit different research environments and data formats, and assure a degree of system portability due to the nature of container virtualization. More information on user-specific configuration settings are provided in Supplementary File S6.''

2. The authors make no mention of any existing privacy-focused workspaces for data sharing and collaboration. There are multiple commercial tools, such as Google Workspace, that enable sharing of experimental raw data and meta data. Many of these tools provide privacy-preserving functionality and are marketed to be in-compliance with protected health data security policy. This reviewer felt only comparing MetaboSERV to public metabolomics data repositories misrepresented available options for secure collaborative research. The authors should mention the state of existing commercial and open-source tools for secure data sharing and collaboration, and what their platform offers relative to these tools to better represent the impact of their platform.

Response by authors:

We are very thankful to the reviewer for pointing out a missing discussion on existing privacy-focused workspaces for data sharing and collaboration beyond metabolomics repositories. We have therefore extended our discussion on this topic and have added the following paragraph to the main manuscript:

"Besides metabolomics-focussed data repositories, a large number of workspaces for data sharing and collaboration with options for privacy preservation have been released in the last decades, including commercial applications like Google Workspace and Nextcloud, as well as a multitude of freely available solutions including Figshare or Synapse/NF Data Portal [48]. Additionally, academic institutions worldwide start building up their own data sharing repositories, e.g., the Academic Cloud service for Lower Saxony, or RepoMed, the institutional repository of Hannover Medical School. However, none of these workspaces and solutions are designed to the specific needs of metabolomics data repositories, but rather provide "data-type agnostic" data lakes for the storage and retrieval of individual data sets [48]. In comparison to MetaboSERV, they do not provide smart search functions for metabolites across several, independent studies, no data analysis or visualization options, and, more importantly, do not support the set-up of self-administered, configurable instances, which are completely independent of the providers. The freely available software FAIRDOM-SEEK [49, 50] can be, similar to MetaboSERV, also deployed locally, however, it is designed particularly for data spanning multiple omics types or interconnecting datasets and systems biology models [49], and not for metabolomics data. Thus, uploaded data sets cannot be systematically

queried or analysed with respect to individual metabolites and/or across studies."

-- Moderate Issues --

3. This reviewer observed some issues with metabolite metadata interactions when using MetaboSERV. The authors mention their tool will search for known synonyms of metabolites to match measures between studies that use different identifiers for the same molecule. However, this reviewer noted the metabolites in the 'AKI Study' and 'Biocrates Test' experiments could not be selectively queried together due to different synonym inputs. For example, 'L-Isoleucine' in the AKI Study did not match the 'Ile' metabolite abbreviation in the Biocrates Test experiment. This easily encountered conflict is highly concerning and suggests conflicting names will severely limit the cross-experiment selection functionality of MetaboSERV. Could the authors enforce uploads using chemical identifiers, such as HMDB, CAS, InChiKey, to enhance integration of multiple independent data sources?

Response by authors:

We thank the reviewer for his/her suggestion to integrate common chemical identifiers into MetaboSERV. We have decided not to enforce the use of chemical identifiers for our public MetaboSERV instance as not to impede the upload of metabolomics experiments containing unknown/unidentified metabolites. Nevertheless, we have now implemented the optional feature of mapping metabolite names to HMDB identifiers directly on the MetaboSERV website prior to data upload, which helps resolving metabolite synonyms. For this mapping process, we use data provided by the HMDB as well as The Chemical Translation Service (CTS) by G. Wohlgemuth et al. (http://cts.fiehnlab.ucdavis.edu). An enforcement of HMDB identifiers can optionally be used for self-managed MetaboSERV instances and can be toggled on or off at any point in time without retroactively affecting already uploaded studies. Enforcing HMDB identifiers will require users to either provide HMDB identifiers instead of metabolite names, or optionally have them mapped to the respective HMDB identifiers during the study creation process. We have thus updated section 3.3.1 in Supplementary File S3: MetaboSERV file specifications:

"The public MetaboSERV platform does not enforce the use of chemical identifiers for metabolite names as not to impede the upload of metabolomics experiments containing unknown/unidentified metabolites. However, to further ease seamless matching of metabolite names across different studies, MetaboSERV offers the optional, semi-automatic mapping of metabolite names to HMDB identifiers directly on the public MetaboSERV instance website prior to data upload. For this mapping process, we use data provided by the HMDB as well as The Chemical Translation Service (CTS) by G. Wohlgemuth et al. [27]. An enforcement of HMDB identifiers can optionally be used for self-managed, local MetaboSERV instances and can be toggled on or off at any point in time without retroactively affecting already uploaded studies. Enforcing HMDB identifiers will require users to either provide HMDB identifiers instead of metabolite names, or optionally have them mapped to the respective HMDB identifiers during the study creation process."

Please note that, however, if metabolites are measured in different units across studies, we have now implemented a functionality that stops the user from creating multi-study-plots to avoid confusion and prevent the creation of misleading plots.

4. The manuscript mentions the cyber security efforts for ensuring the privacy of user created accounts and passwords. This reviewer observed that newly registered accounts did not require email validation, thus it would be very easy to impersonate any researcher by creating an account using a known email address.

Response by authors:

We are very thankful to the reviewer for pointing this out. To address this important comment, we have now introduced an e-mail verification system: Upon account creation, an e-mail containing a randomly generated, unique verification code is sent to the e-mail address specified by the new user. This code - which is valid for 24 hours - has to be entered once per account to verify it. Unverified accounts are treated as accounts with no special permissions and can only view or browse public studies. The e-mails are sent using SMTP by a function e-mail account, which has to be provided by administrators of self-managed

MetaboSERV instances, should they choose to use this feature. While this e-mail verification step can be toggled off for self-managed MetaboSERV instances (which will cause all accounts to be auto-verified), it is mandatory for the public MetaboSERV instance. We provide detailed information on this e-mail verification system in the main manuscript (section "Data access control and management"):

"Upon registration, an e-mail containing a verification code is sent to the e-mail address that was used to register the account. This unique code, which consists of ten random characters, must be entered once after logging in to unlock any permissions associated with the account, which includes viewing studies shared with the account or uploading studies. The user accounts are associated with access rights for specific studies. For each uploaded data set, two different levels of access rights can be granted to other user accounts - either "read-only permissions" or "full data editing and management authority" - by the original uploader or users with "full data editing and management authority" accounts. The account responsible for the initial creation of the study possesses "full data editing and management authority" at any time and can never be removed as a contributor by any other user."

as well as in Supplementary File S2: User Authentication and Password Storage:

"In order to fully activate an account, the MetaboSERV platform uses an e-mail verification system, which can optionally be disabled for self-managed MetaboSERV instances. Upon registration, an e-mail containing a verification code is sent to the e-mail address that was used to register the account. This unique code, which consists of ten random characters, must be entered once after logging in to unlock any permissions associated with the account, which includes viewing studies shared with the account or uploading studies. Each code is valid for 24 hours or until a new code is generated. Failed login attempts are tracked and displayed to the user when they successfully log in. After a default number of five failed attempts, an e-mail reporting potentially suspicious activities is sent to the e-mail address associated with the account. After a default number of ten failed attempts, the account is temporarily locked for ten minutes. The number of attempts for both of those events can be configured for self-managed instances. A password recovery service allows the recovery of lost passwords associated with an account by employing the associated e-mail address. The e-mails are sent using SMTP by a function e-mail account, which has to be provided by administrators of self-managed MetaboSERV instances, should they choose to use this feature."

-- Minor Issues --

5. It would be very helpful to provide example upload templates for the 'Concentration data' and 'Phenotype data' study sections on MetaboSERV to ease the new user experience of this platform.

Response by authors:

We thank the reviewer for this thoughtful comment and now provide templates for the 'Concentration data', 'Phenotype data', as well as 'metadata'-YAML-files for download on the public MetaboSERV landing page. Additionally, we provide complete example data files for the three use cases on the MetaboSERV landing page. We now point out this information to the reader in Supplementary File S3: MetaboSERV file specifications:

"Templates for 'Concentration data', 'Phenotype data', as well as 'metadata'-YAML-files are provided for download on the public MetaboSERV landing page at https://metaboserv.ckdn.app/. Furthermore, complete example data files for the three use cases are provided on the same webpage."

6. The liquid chromatography platform model is missing from the description of the Biocrates MxP Quant 500 kit experiment in the methods section.

Response by authors:

We thank the reviewer for pointing out this missing information, which we now added to the following section in the main manuscript:

"Data was acquired on an AB Sciex 6500+ triple quadrupole mass spectrometer (AB Sciex Germany GmbH, Darmstadt, Germany) coupled to an ExionLC 30AD (AB Sciex Germany GmbH, Darmstadt, Germany) ultra-high performance liquid chromatography (UHPLC) system employing the MxP Quant 500 kit (Biocrates life sciences, Innsbruck, Austria)."

7. It appears that studies cannot be edited after creation when using the public instance. Is this the intended functionality or a bug? The manuscript alludes to that multiple researchers could upload the raw data, meta data, and phenotypes, but it is unclear how this could be accomplished if studies are not editable after creation.

Response by authors:

We apologize that during our first submission, the studies were not yet editable. We have now reactivated this functionality and all studies are editable by the study owners and users with the respective "full data editing and management authority" permissions.

8. How are unique study IDs enforced? Would private studies require unique study IDs from other private studies? How would users know what IDs are unavailable for use?

Response by authors:

We are very thankful to the reviewer for his/her inquiry about unique study IDs. We have now included the following paragraph in the Supplementary File S1, section 3.1.1:

"Each study object - such as the AKI study - is associated with a particular set of experiments and relevant metadata attributes such as the study authors, one or more biospecimens, the publication date or relevant filenames, as well as a unique study ID. The user provides a unique study ID, which is automatically compared to all existing study identifiers of the used MetaboSERV instance which are stored in both the MariaDB and Elasticsearch database. In the case a new study ID is already stored in the databases, a conflict message is shown and the user is asked to provide a new study ID."

9. When a user's study uses multiple methods, there is nowhere to specify which multiple methods where used. For example, a typical Biocrates MxP Quant 500 experiment includes an LC and flow injection component, but the example data only notes the LC method. This issue is also observed in the Biospecimen category as well.

Response by authors:

We are very thankful to the reviewer for pointing this out. We now revised the implementation of both the "Analytical Method" and "Biospecimen" selection tabs on the "Create a new study" site to allow the selection of multiple options. On the corresponding "Data & Metadata" project site, these multiple methods and/or biospecimen types are then listed individually. These edits affect Figure 3A in the main manuscript, which has been updated accordingly.

Furthermore, we would like to point out that a data set comprising metabolites measured, for example, by different hyphenated MS modalities can be separated into multiple studies, each comprising metabolites measured by one specific modality. This allows the detailed description of the different methodological metadata associated with each modality. We describe this scenario in the newly added use case 3 in Supplementary File S5.

10. Does MetaboSERV also record methodological meta data, such as chromatography, MS, or NMR parameters? These are critical components to existing public metabolomics data repositories mentioned throughout this manuscript but are undiscussed in the context of this manuscript.

Response by authors:

MetaboSERV allows the recording of methodological metadata without any format and/or content

restrictions. These methodological metadata can be provided by JSON/YAML metadata files and the deposited metadata is displayed on the corresponding "Data & Metadata" project site. To facilitate a low-threshold user experience of MetaboSERV, we have implemented a very flexible metadata upload by deliberately not enforcing mandatory metadata specifications. To illustrate this flexibility, we now specifically provide metadata of various resolution for the different use cases on the MetaboSERV public instance. Example JSON/YAML metadata files of all use cases as well as template files are further provided for download both on the public MetaboSERV landing page as well as the MetaboSERV GitLab repository (see also our response to comment 5 of reviewer 1). We now contrast MetaboSERV's flexibility with respect to methodological metadata in comparison to existing metabolomics data repositories in the discussion:

"Furthermore, MetaboSERV allows the recording of methodological metadata without any format and/or content restrictions. To facilitate a low-threshold user experience of MetaboSERV, we implemented a very flexible metadata upload by deliberately not enforcing mandatory metadata specifications. In contrast, established metabolomics data repositories such as MetaboLights and the Metabolomics Workbench face the users with rather strict mandatory metadata as well as experimental data upload requirements."

--- Reviewer 2 ---

The manuscript presents MetaboSERV, an open-source platform for metabolomics data selection, integration, and controlled access sharing. The manuscript is well-structured and clearly explains the functionalities and use cases of MetaboSERV. However, a few areas where additional detail and clarification could enhance the manuscript's clarity and impact.

Response by authors:

We thank the reviewer for his/her positive feedback and appreciation and have carefully tried to address his/her concerns as outlined below.

Q1: The authors highlight MetaboSERV's potential in maintaining data privacy in compliance with GDPR and similar regulations. Can MetaboSERV detect and log unauthorized access attempts, and if so, how are these managed? A deeper discussion on MetaboSERV's robustness in data security would better inform readers of its suitability for handling sensitive biomedical data.

Response by authors:

We thank the reviewer for his/her suggestion to log unauthorized access attempts, which we have now implemented. The number of unauthorized attempts, which occurred since their last successful login, is now displayed to the user whenever they sign in. Also, an e-mail is sent to the e-mail address linked with the MetaboSERV account after a configurable number of tracked unauthorized access attempts, and the account is locked (i.e. unable for login) for ten minutes after another configurable number of tracked unauthorized access attempts. On the public instance, these are set to five and ten attempts, respectively. Moreover, we have implemented a password recovery service which allows the recovery of lost passwords associated with an account by employing the associated e-mail address.

We have included this information in the main manuscript, section "Data access control and management":

"Unauthorized access attempts are continuously logged in the Elasticsearch database. The number of unauthorized access attempts, which occurred since the last authorized log-in, is reported to the user upon every log-in. In the public MetaboSERV instance, an alert e-mail is sent to the user's e-mail address linked to the account in case the number of unauthorized access attempts exceeds five attempts, and the account is locked for ten minutes after a total of ten tracked unauthorized access attempts. On a successful log-in attempt, this counter is reset to zero. More information and customization details for local MetaboSERV instances are provided in Supplementary File S2."

and provide detailed information in Supplementary File S2: User Authentication and Password Storage:

"In order to fully activate an account, the MetaboSERV platform uses an e-mail verification system, which

can optionally be disabled for self-managed MetaboSERV instances. Upon registration, an e-mail containing a verification code is sent to the e-mail address that was used to register the account. This unique code, which consists of ten random characters, must be entered once after logging in to unlock any permissions associated with the account, which includes viewing studies shared with the account or uploading studies. Each code is valid for 24 hours or until a new code is generated. Failed login attempts are tracked and displayed to the user when they successfully log in. After a default number of five failed attempts, an e-mail reporting potentially suspicious activities is sent to the e-mail address associated with the account. After a default number of ten failed attempts, the account is temporarily locked for ten minutes. The number of attempts for both of those events can be configured for self-managed instances. A password recovery service allows the recovery of lost passwords associated with an account by employing the associated e-mail address. The e-mails are sent using SMTP by a function e-mail account, which has to be provided by administrators of self-managed MetaboSERV instances, should they choose to use this feature."

Furthermore, we now elaborately discuss the server environment structure and organizational security measures of the public MetaboSERV instance hosted at MHH in the section "MetaboSERV server environment":

"The public MetaboSERV instance, available at https://metaboserv.ckdn.app, is hosted at the computing center of the Hannover Medical School, Germany (MHH). MHH's regulations for access control to the server, security updates, backup and monitoring are implemented following the ISO 27001 and the standards of the German Federal Office for Information Security (Bundesamt für Sicherheit in der Informationstechnik, BSI). In particular, the public MetaboSERV server at MHH and the data stored there can only be accessed by authorized administrators. Server access is continuously logged and regularly inspected. The virtual machine is equipped with the latest security updates and regular backups are being taken every six hours."

Q2: How does MetaboSERV scale with larger datasets, such as those often encountered in multi-site studies or large biobanks? The examples provided as test data are relatively limited in size. Clinical metabolomics datasets can reach thousands of samples, so this aspect of scalability warrants further clarification.

Response by authors:

To demonstrate the scalability of MetaboSERV, we have added a third use case in Supplementary Section S5: "Use case 3: Large-scale, multi-modal, untargeted mass spectrometry data", employing a publicly available dataset from a study provided by Lesley A. Inker at Tufts Medical Center, University of Minnesota, including 1,002 samples x 1,228 metabolites. The dataset was split into four different MetaboSERV studies - one for each of the four different LC/MS modalities used for data collection - to comply with MetaboSERV's limit of 1,016 metabolites per uploaded study. By conducting this data subsetting, we also intend to show a possible representation of multiple LC/MS methods as suggested by comment 9 of reviewer 1. All four subsets contain data on the same 1,002 samples. The four LC/MS modalities and resulting dataset dimensionalities are: "LC/MS Neg" (692 metabolites), "LC/MS Polar" (79 metabolites), "LC/MS Pos Late" (173 metabolites), and "LC/MS Pos Early" (284 metabolites). More details on the different LC/MS modalities are provided in the main manuscript as well as in the Supplementary Material. Metabolite concentrations are given in arbitrary units ("a.u.").

To aid in displaying data from a study partitioned in this manner, we have also implemented an optional functionality that aggregates data based on the sample identifiers. As the same 1,002 samples are contained in all four studies, a joint query on all four studies can be performed in order to display the results as if they were retrieved from a single study. This feature can also be used to subset the data - for example, a joint query on only three out of the four studies could be performed in order to only retrieve data of three of the four LC/MS modalities.

We timed the upload and querying processes on a system that does not meet our minimum hardware recommendations - at least 2 CPU cores and 6GB of RAM - in order to determine MetaboSERV's performance on large datasets and to obtain a lower bound for the expected waiting time when dealing

with large-scale metabolomics experiments using MetaboSERV. The system we used runs on 4GB of RAM and 2 cores.
Uploading times during study creation for each of the four studies ranged between 20 and 90 seconds, with the LC/MS Neg dataset taking the longest time due to the largest number of metabolites. The most performance-heavy query possible - the aforementioned joint query on all four study partitions, with the aggregation feature turned on - took 37 seconds to process on this setup.
Quality control plots for each of the partitions are generated in less than 30 seconds. Quality control plots based on the samples (as opposed to the metabolites), however, are generally generated much faster.

We describe use case 3 as follows in Supplementary Section S5:

"File S5: Use case 3: Large-scale, multi-modal, untargeted mass spectrometry data

In a third use case, we demonstrate the application of MetaboSERV on a large-scale, multi-modal, untargeted mass spectrometry data set, a typical scenario for major epidemiological studies:

An epidemiological research group would like to share a data set consisting of 1,228 unique metabolites semi-quantitatively measured by Metabolon in 1,002 human blood plasma specimens as well as the corresponding phenotype data on discovery/validation as well as quality control sample cohorts with two international collaboration partners. As these metabolites had been measured by four different LC/MS methods, they divide the data set into four subsets: (1) the LCMSneg subset, comprising 692 metabolites analyzed using basic negative ion optimized conditions, (2) the LCMSposearly subset, comprising 284 metabolites analyzed using acidic positive ion conditions, chromatographically optimized for more hydrophilic compounds, (3) the LCMSposlate subset with 173 metabolites analyzed using acidic positive ion conditions, chromatographically optimized for more hydrophobic compounds, and (4) the LCMSpolar subset consisting of 79 metabolites analyzed via negative ionization following elution from a HILIC column [28]. For each data subset, they have created separate YAML files comprising individual experimental metadata. As the metabolites have only been semi-quantitatively measured, the epidemiological researchers specify the unit as "a.u.", i.e., arbitrary unit, in the concentration files. They upload each concentration and corresponding YAML file separately, which takes between 20 to 90 seconds depending on the amount of contained metabolites. They add their collaboration partners as "contributors" to the study.

The collaboration partners log into MetaboSERV and select all four studies, "ST002820 Metabolon LCMSneg", "ST002820 Metabolon LCMSposlate", "ST002820 Metabolon LCMSposearly", and "ST002820 Metabolon LCMSpolar" in the 'Query' browser. They scroll through the list of metabolites, alphabetically ordered across all four studies, and realize that the unidentified metabolite 'X - 02269' appears twice in the list, one time with the name 'X - 02269_1' and one time with the name 'X - 02269_2'. The same holds true for the metabolite '7-methylurate', which appears twice in the list. As the collaborators would like to understand why these two metabolites each appear twice in the list, they retrieve only these four metabolites in the 'Metabolite Selection' menu by selecting 'Choose subset', shown in Supplementary Figure S4. Furthermore, they choose the 'Filter Type' 'vis' for all four metabolites and for the phenotype information, only include the levels "Discovery", "Validation 1", "Validation 2", and "Blind QC". They hit the 'Submit' button without aggregation. In the 'Query Results' table, they order the results according to the provided phenotype variable, scroll through the individual Source IDs and realize that '7-methylurate_1' and 'X - 02269_1' are only available in the "LCMSneg" study, and '7-methylurate_2' and 'X - 02269_2' are only available in the "LCMSposearly" study (Supplementary Figure S5). As they are curious about the relationships between the respective metabolites, the collaboration partners first visualize the association between '7-methylurate_1' and '7-methylurate_2' in a scatterplot by selecting these two metabolites (Supplementary Figure S6A). In a second step, they perform the same analysis for the metabolites 'X - 02269_1' and 'X - 02269_2' (Supplementary Figure S6B). In both scatterplots, a high correlation between the respective metabolites can be detected, but it becomes also apparent, that the intensity scales seem to differ between the two LC/MS methods. The collaboration partners contact the epidemiology research group and point out their observations in order to discuss further data preprocessing steps."

Q3: The manuscript mentions the availability of Docker images for self-managed instances of MetaboSERV. Are these images easy to configure for users with limited technical backgrounds? In my lab, I use multiple software tools for processing raw data (e.g., MZmine, MS-DIAL, XCMS), structure elucidation (e.g., MS-

FINDER), and in-house Python-based programs for data curation—all of which do not require extensive IT skills to set up. MetaboSERV seems more challenging to install, so a detailed manual would be beneficial.

Response by authors:

We provide a detailed installation and user guide in Supplementary File S6, section 3.6 "File S6: Detailed installation and user guide for the set-up of local MetaboSERV instances", which is also available at https://metaboserv.ckdn.app/guide on the public MetaboSERV instance as well as on the Gitlab repository at https://gitlab.gwdg.de/metaboserv2/backend/-/blob/main/README.md. Furthermore, we have created a video tutorial on the installation and management of local MetaboSERV instances, which is freely available at https://www.youtube.com/watch?v=vbqY2qJcgLk. We anticipate that these detailed manuals enable users without extensive IT skills to independently set-up and manage local MetaboSERV instances. For further support, we have added contact details for technical support on the landing page of the public MetaboSERV instance.

Q4: Is technical support available for installation and setup, or are there resources like a detailed user guide? Addressing this would reassure researchers of varying technical skill levels about the feasibility of independently setting up MetaboSERV.

Response by authors:

A detailed installation manual and user guide can be found on the "Guide"-tab of the MetaboSERV public instance, to which we provide a link on the corresponding landing page, as well as on the MetaboSERV GitLab page. Additionally, we have created a video tutorial on the installation and management of local MetaboSERV instances, which is freely available at https://www.youtube.com/watch?v=vbqY2qJcgLk. Moreover, we have now included the detailed user guide in the Supplement as Supplementary File S6: "Detailed installation and user guide for the set-up of local MetaboSERV instances", section 3.6.2 "Using MetaboSERV". Likewise, we have added contact details for technical support on the landing page of the MetaboSERV public instance.

Q5: The authors limit the application of MetaboSERV to projects where metabolite concentrations are reported in molar units. This could exclude a substantial portion of metabolomics studies starting with a discovery cohort typically acquired using untargeted LC-MS or GC-MS-based metabolomics, often reported as peak areas or intensities in arbitrary units. Broadening this scope may enhance the platform's usability across a wider range of studies.

Response by authors:

MetaboSERV supports completely arbitrary units, as it simply uses what is provided by the user. However, if metabolites are measured in different units across studies, we have implemented a functionality that stops the user from creating multi-study-plots to avoid confusion and prevent the creation of misleading plots. To point out the flexibility of MetaboSERV with respect to concentration units to the reader, we now explicitly mention this in Supplementary File S3: MetaboSERV file specifications, section 3.3.1:

"The unit of measurement for each metabolite can be indicated by adding an extra 'unit' column and providing the unit for each metabolite in that column. To avoid the creation of misleading plots, MetaboSERV, however, does not allow the creation of multi-study-plots featuring the same metabolite measured in different units. To facilitate such analyses, the user needs to match the respective concentration units by converting them appropriately prior to data upload to MetaboSERV."

Moreover, we have now added a third use case with untargeted LC-MS data provided in arbitrary units, as detailed in our response to comment Q2 of reviewer 2.

Q6: How does MetaboSERV handle uploading raw LC-MS or GC-MS data? Here, raw data refer to the files generated directly from mass spectrometers (e.g., Agilent .d, Thermo .raw, Sciex .wiff, .wiff2). While MetaboSERV supports the upload and visualization of NMR spectra, extending this support to MS data would be beneficial, as LC-MS is a cornerstone in metabolomics.

Response by authors:

MetaboSERV allows the upload of raw (hyphenated) MS data of any data format, as long as it is bundled into a zip-file. In contrast to NMR metabolomics, which, in most cases, employs a Bruker NMR spectrometer and therefore, most of the NMR raw data available is provided in the Bruker file format, MS utilizes a large variety of different instrument providers, which all use their own data formats (as also reflected by the list of MS file formats provided by the reviewer). Since freely available software for the visualization of raw MS data are typically limited to specific raw data file formats, we have not implemented visualization options for raw (hyphenated) MS data. Moreover, MetaboSERV is designed for interdisciplinary collaborations involving researchers with varying background knowledge on MS. Since the profound judgment of the data quality of any raw metabolomics experiment by visual inspection is, at least to our knowledge, only possible by wet-lab metabolomics experts, we believe that the use of data visualizations of raw MS data within MetaboSERV is rather limited to wet-lab metabolomics experts. The latter, however, are typically able to easily visualize the raw data in the corresponding MS vendor software. In case the wet-lab metabolomics experts want to share these visualizations, they still have the option to share any visualization files, generated outside of MetaboSERV, within the zip-file bundle of the raw experimental data uploaded to MetaboSERV. To stress that the visualization option in the MetaboSERV platform is restricted to raw NMR data in the Bruker format, we have modified Figure 2 in the main manuscript accordingly.

Q7: Using the https://metaboserv.ckdn.app/ website, I attempted to replicate Figure 4. However, no response was received after submitting, so I could not test the functionalities associated with Figures 5 and 6.

Response by authors:

We are very sorry about this inconvenience and have carefully rechecked all functionalities prior to resubmission.

Editorial Manager®

Cookie Preference Center

We use cookies which are necessary to make our site work. We may also use additional cookies to analyze, improve and personalize our content and your digital experience. For more information, see our Cookie Policy.

You may choose not to allow some types of cookies. However, blocking some types may impact your experience of our site and the services we are able to offer. See the different category headings below to find out more or change your settings.

Allow all

Manage Consent Preferences

Strictly Necessary Cookies

Always active

These cookies are necessary for the website to function and cannot be switched off in our systems. They are usually only set in response to actions made by you which amount to a request for services, such as setting your privacy preferences, logging in or filling in forms. You can set your browser to block or alert you about these cookies, but some parts of the site will not then work.

Functional Cookies

These cookies enable the website to provide enhanced functionality and personalisation. They may be set by us or by third party providers whose services we have added to our pages. If you do not allow these cookies then some or all of these services may not function properly.

Performance Cookies

These cookies allow us to count visits and traffic sources so we can measure and improve the performance of our site. They help us to know which pages are the most and least popular and see how visitors move around the site.

Targeting Cookies

These cookies may be set through our site by our advertising partners. They may be used by those companies to build a profile of your interests and show you relevant adverts on other sites. If you do not allow these cookies, you will experience less targeted advertising.

Cookie List

Clear

Apply Cancel

Consent Leg.Interest

Confirm my choices

Powered by onetrust
