# Supplementary material for: MetaboSERV—a platform for selecting, exchanging, and visualizing metabolomics data with controlled data access
Source: Gigascience. 2025 Aug 1;14:giaf075. doi: 10.1093/gigascience/giaf075 (PMC12315529; doi:10.1093/gigascience/giaf075)
Supplement: giaf075_Reviewer_1_Report_Original_Submission — Kevin Murray, Ph.D. -- 10/19/2024 [file giaf075_reviewer_1_report_original_submission.pdf]

Reviewer Report

Title: MetaboSERV - a platform for selecting, exchanging, and visualizing metabolomics data with controlled data access

Version: Original Submission **Date:** 10/19/2024

Reviewer name: Kevin Murray, Ph.D.

Reviewer Comments to Author:

The manuscript "MetaboSERV - platform for selecting, exchanging, and visualizing metabolomics data with controlled data access" presents an ambitious resource for metabolomics research which aims to provide a collaborative workspace tool designed for team-driven research efforts. The technical note emphasizes the privacy-preserving functionality of MetaboSERV and offers selective curation tools to evaluate multiple experimental outcomes simultaneously. The platform presented in this paper has the potential to be a valuable tool to research teams, but this reviewer was confused on what role the proposed platform serves in a broader scientific context. Below is a list of issues that should be addressed before this manuscript is suitable for publication:Major Issues1. MetaboSERV is regularly compared to existing public metabolomics data repositories, but it was unclear if it is a public repository itself. The public instance of MetaboSERV only contains the example results outlined in this manuscript and none of the raw data from those experiments was available for download from the public instance. Additionally, this reviewer was unable to upload raw experimental data to the public instance on their own account. Is the public instance of MetaboSERV intended for end users or just a demo of a potential private instance? The manuscript is so focused on privacy and team collaboration, it is entirely unclear if this platform serves any functionality as a public data repository. As the introduction and discussion sections of this manuscript heavily compare MetaboSERV to existing public data repositories, the authors need to disambiguate this potential source of confusion.2. The authors make no mention of any existing privacy-focused workspaces for data sharing and collaboration. There are multiple commercial tools, such as Google Workspace, that enable sharing of experimental raw data and meta data. Many of these tools provide privacy-preserving functionality and are marketed to be in-compliance with protected health data security policy. This reviewer felt only comparing MetaboSERV to public metabolomics data repositories misrepresented available options for secure collaborative research. The authors should mention the state of existing commercial and open-source tools for secure data sharing and collaboration, and what their platform offers relative to these tools to better represent the impact of their platform.Moderate Issues3. This reviewer observed some issues with metabolite metadata interactions when using MetaboSERV. The authors mention their tool will search for known synonyms of metabolites to match measures between studies that use different identifiers for the same molecule. However, this reviewer noted the metabolites in the `AKI Study` and `Biocrates Test` experiments could not be selectively queried together due to different synonym inputs. For example, `L-Isoleucine` in the AKI Study did not match the `Ile` metabolite abbreviation in the Biocrates Test experiment. This easily encountered conflict is highly concerning and suggests conflicting names will severely limit the cross-experiment selection functionality of MetaboSERV. Could the authors enforce uploads using chemical

identifiers, such as HMDB, CAS, InChiKey, to enhance integration of multiple independent data sources?4. The manuscript mentions the cyber security efforts for ensuring the privacy of user created accounts and passwords. This reviewer observed that newly registered accounts did not require email validation, thus it would be very easy to impersonate any researcher by creating an account using a known email address.Minor Issues5. It would be very helpful to provide example upload templates for the `Concentration data` and `Phenotype data` study sections on MetaboSERV to ease the new user experience of this platform.6. The liquid chromatography platform model is missing from the description of the Biocrates MxP Quant 500 kit experiment in the methods section.7. It appears that studies cannot be edited after creation when using the public instance. Is this the intended functionality or a bug? The manuscript alludes to that multiple researchers could upload the raw data, meta data, and phenotypes, but it is unclear how this could be accomplished if studies are not editable after creation.8. How are unique study IDs enforced? Would private studies require unique study IDs from other private studies? How would users know what IDs are unavailable for use?9. When a user's study uses multiple methods, there is nowhere to specify which multiple methods where used. For example, a typical Biocrates MxP Quant 500 experiment includes an LC and flow injection component, but the example data only notes the LC method. This issue is also observed in the Biospecimen category as well.10. Does MetaboSERV also record methodological meta data, such as chromatography, MS, or NMR parameters? These are critical components to existing public metabolomics data repositories mentioned throughout this manuscript but are undiscussed in the context of this manuscript.

Level of Interest

Please indicate how interesting you found the manuscript: Choose an item.

Quality of Written English

Please indicate the quality of language in the manuscript: Choose an item.

Declaration of Competing Interests

Please complete a declaration of competing interests, considering the following questions:

- Have you in the past five years received reimbursements, fees, funding, or salary from an organisation that may in any way gain or lose financially from the publication of this manuscript, either now or in the future?
- Do you hold any stocks or shares in an organisation that may in any way gain or lose financially from the publication of this manuscript, either now or in the future?
- Do you hold or are you currently applying for any patents relating to the content of the manuscript?
- Have you received reimbursements, fees, funding, or salary from an organization that holds or has applied for patents relating to the content of the manuscript?
- Do you have any other financial competing interests?

- Do you have any non-financial competing interests in relation to this paper?

If you can answer no to all of the above, write 'I declare that I have no competing interests' below. If your reply is yes to any, please give details below.

I declare that I have no competing interests

I agree to the open peer review policy of the journal. I understand that my name will be included on my report to the authors and, if the manuscript is accepted for publication, my named report including any attachments I upload will be posted on the website along with the authors' responses. I agree for my report to be made available under an Open Access Creative Commons CC-BY license (http://creativecommons.org/licenses/by/4.0/). I understand that any comments which I do not wish to be included in my named report can be included as confidential comments to the editors, which will not be published.

Choose an item.

To further support our reviewers, we have joined with Publons, where you can gain additional credit to further highlight your hard work (see: https://publons.com/journal/530/gigascience). On publication of this paper, your review will be automatically added to Publons, you can then choose whether or not to claim your Publons credit. I understand this statement.

Yes Choose an item.
