# Supplementary material for: MetaboSERV—a platform for selecting, exchanging, and visualizing metabolomics data with controlled data access
Source: Gigascience. 2025 Aug 1;14:giaf075. doi: 10.1093/gigascience/giaf075 (PMC12315529; doi:10.1093/gigascience/giaf075)
Supplement: giaf075_Reviewer_2_Report_Original_Submission — javascript:popupReviewerInfo(18892,%207763) -- 11/7/2024 [file giaf075_reviewer_2_report_original_submission.pdf]

Reviewer Report

Title: MetaboSERV - a platform for selecting, exchanging, and visualizing metabolomics data with controlled data access

Version: Original Submission **Date:** 11/7/2024

Reviewer name: TomÃ¡Å¡ ÄŒajka

Reviewer Comments to Author:

The manuscript presents MetaboSERV, an open-source platform for metabolomics data selection, integration, and controlled access sharing. The manuscript is well-structured and clearly explains the functionalities and use cases of MetaboSERV. However, a few areas where additional detail and clarification could enhance the manuscript's clarity and impact.Q1: The authors highlight MetaboSERV's potential in maintaining data privacy in compliance with GDPR and similar regulations. Can MetaboSERV detect and log unauthorized access attempts, and if so, how are these managed? A deeper discussion on MetaboSERV's robustness in data security would better inform readers of its suitability for handling sensitive biomedical data.Q2: How does MetaboSERV scale with larger datasets, such as those often encountered in multi-site studies or large biobanks? The examples provided as test data are relatively limited in size. Clinical metabolomics datasets can reach thousands of samples, so this aspect of scalability warrants further clarification.Q3: The manuscript mentions the availability of Docker images for self-managed instances of MetaboSERV. Are these images easy to configure for users with limited technical backgrounds? In my lab, I use multiple software tools for processing raw data (e.g., MZmine, MS-DIAL, XCMS), structure elucidation (e.g., MS-FINDER), and in-house Python-based programs for data curationâ€”all of which do not require extensive IT skills to set up. MetaboSERV seems more challenging to install, so a detailed manual would be beneficial.Q4: Is technical support available for installation and setup, or are there resources like a detailed user guide? Addressing this would reassure researchers of varying technical skill levels about the feasibility of independently setting up MetaboSERV.Q5: The authors limit the application of MetaboSERV to projects where metabolite concentrations are reported in molar units. This could exclude a substantial portion of metabolomics studies starting with a discovery cohort typically acquired using untargeted LC-MS or GC-MS-based metabolomics, often reported as peak areas or intensities in arbitrary units. Broadening this scope may enhance the platform's usability across a wider range of studies.Q6: How does MetaboSERV handle uploading raw LC-MS or GC-MS data? Here, raw data refer to the files generated directly from mass spectrometers (e.g., Agilent .d, Thermo .raw, Sciex .wiff, .wiff2). While MetaboSERV supports the upload and visualization of NMR spectra, extending this support to MS data would be beneficial, as LC-MS is a cornerstone in metabolomics.Q7: Using the https://metaboserv.ckdn.app/ website, I attempted to replicate Figure 4. However, no response was received after submitting, so I could not test the functionalities associated with Figures 5 and 6.

Level of Interest

Please indicate how interesting you found the manuscript: Choose an item.

Quality of Written English

Please indicate the quality of language in the manuscript: Choose an item.

Declaration of Competing Interests

Please complete a declaration of competing interests, considering the following questions:

- Have you in the past five years received reimbursements, fees, funding, or salary from an organisation that may in any way gain or lose financially from the publication of this manuscript, either now or in the future?
- Do you hold any stocks or shares in an organisation that may in any way gain or lose financially from the publication of this manuscript, either now or in the future?
- Do you hold or are you currently applying for any patents relating to the content of the manuscript?
- Have you received reimbursements, fees, funding, or salary from an organization that holds or has applied for patents relating to the content of the manuscript?
- Do you have any other financial competing interests?
- Do you have any non-financial competing interests in relation to this paper?

If you can answer no to all of the above, write 'I declare that I have no competing interests' below. If your reply is yes to any, please give details below.

I declare that I have no competing interests.

I agree to the open peer review policy of the journal. I understand that my name will be included on my report to the authors and, if the manuscript is accepted for publication, my named report including any attachments I upload will be posted on the website along with the authors' responses. I agree for my report to be made available under an Open Access Creative Commons CC-BY license (http://creativecommons.org/licenses/by/4.0/). I understand that any comments which I do not wish to be included in my named report can be included as confidential comments to the editors, which will not be published.

Choose an item.

To further support our reviewers, we have joined with Publons, where you can gain additional credit to further highlight your hard work (see: https://publons.com/journal/530/gigascience). On publication of this paper, your review will be automatically added to Publons, you can then choose whether or not to claim your Publons credit. I understand this statement.

Yes Choose an item.
